# Late‐Stage Amination of Drug‐Like Benzoic Acids: Access to Anilines and Drug Conjugates through Directed Iridium‐Catalyzed C−H Activation

**DOI:** 10.1002/chem.202103510

**Published:** 2021-11-17

**Authors:** Erik Weis, Magnus J. Johansson, Belén Martín‐Matute

**Affiliations:** ^1^ Department of Organic Chemistry Stockholm University 106 91 Stockholm Sweden; ^2^ Medicinal Chemistry Research and Early Development Cardiovascular Renal and Metabolism (CVRM) Biopharmaceuticals R&D AstraZeneca Gothenburg Pepparedsleden 1 431 50 Mölndal Sweden

**Keywords:** amination, C−H activation, conjugation, high-throughput experimentation, iridium

## Abstract

The functionalization of C−H bonds, ubiquitous in drugs and drug‐like molecules, represents an important synthetic strategy with the potential to streamline the drug‐discovery process. Late‐stage aromatic C−N bond–forming reactions are highly desirable, but despite their significance, accessing aminated analogues through direct and selective amination of C−H bonds remains a challenging goal. The method presented herein enables the amination of a wide array of benzoic acids with high selectivity. The robustness of the system is manifested by the large number of functional groups tolerated, which allowed the amination of a diverse array of marketed drugs and drug‐like molecules. Furthermore, the introduction of a synthetic handle enabled expeditious access to targeted drug‐delivery conjugates, PROTACs, and probes for chemical biology. This rapid access to valuable analogues, combined with operational simplicity and applicability to high‐throughput experimentation has the potential to aid and considerably accelerate drug discovery.

## Graphical abstract

**Added at the eleventh hour**: A method for the late‐stage directed amination of benzoic acids is presented. It allows rapid access to both small amino‐functionalized analogues and large conjugates, including PROTACs, peptide conjugates and probes for chemical biology. Selection of the right catalyst and reagent enabled the use of high‐throughput experimentation and automation.

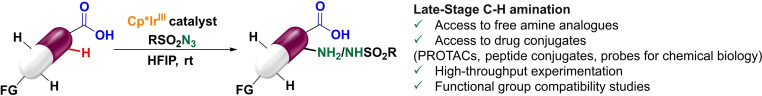

## Introduction

**Figure 1:**
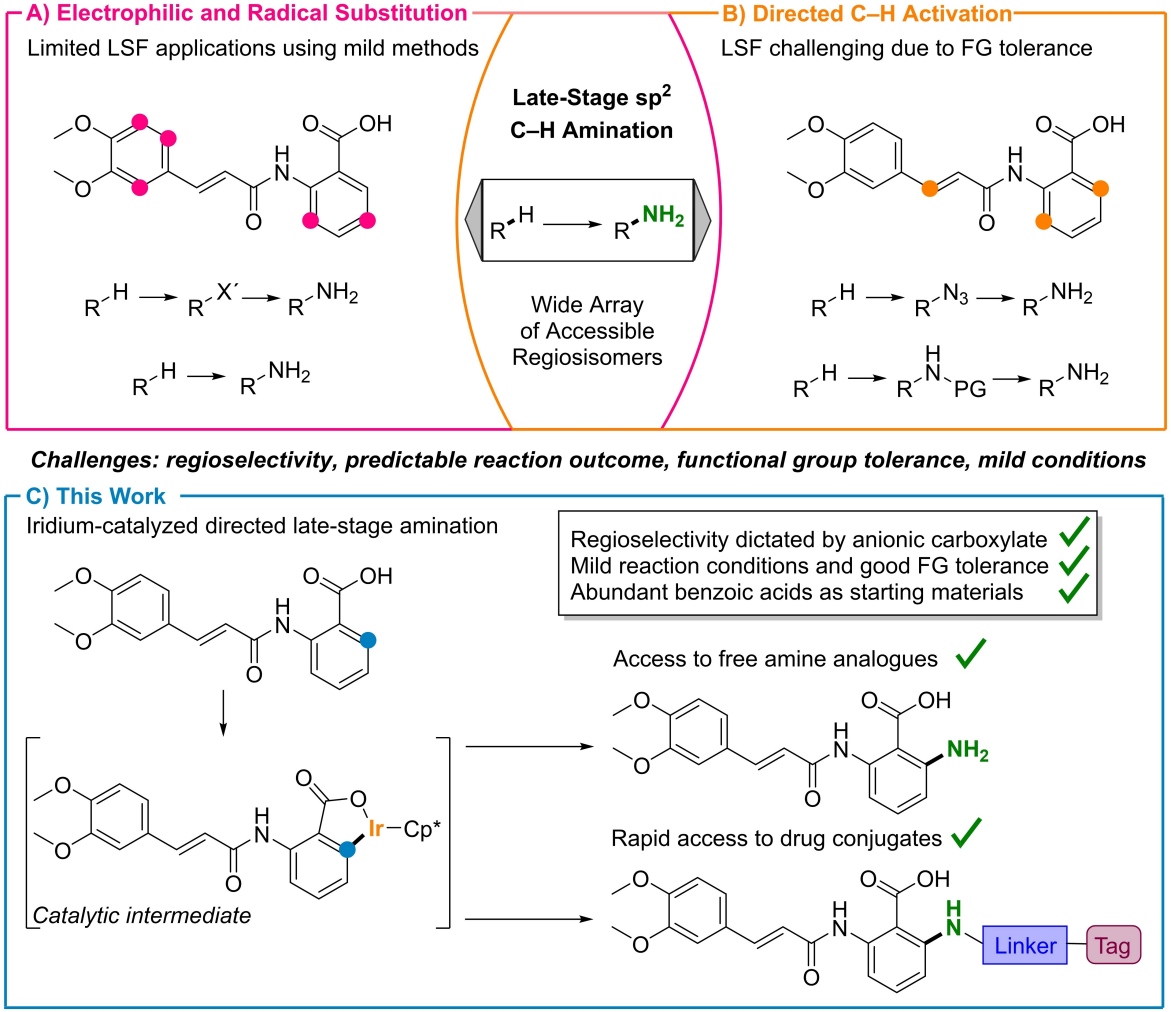
Approaches to late‐stage amination of the Tranilast model system. A) Electrophilic and radical substitutions. Regioselectivity is governed by the steric and electronic properties of the substrate. Pink dots show anticipated reaction sites. S_E_Ar: electrophilic aromatic substitution; X': nitro, halide. B) Directed C−H activation. Functionalization of C−H bonds in proximal to Lewis basic directing groups. Orange dots show anticipated reaction sites; FG: functional group. C) This work. Carboxylate‐directed regioselective C−H activation. The high regioselectivity is enabled by the transformations proceeding via an iridacycle as depicted.

Over the past years, late‐stage functionalization (LSF), the controlled chemoselective transformation of complex molecules to yield matched molecular pairs/drug‐like analogues, has emerged as a powerful strategy in medicinal chemistry.^[1]^ This approach allows to bypass the need for *de novo* synthesis of each desired analogue. In turn, this has the potential to vastly accelerate the drug‐discovery process, particularly the hit‐to‐lead optimization stage, and consequently increase the efficiency and reduce the cost and time required to access new candidate drugs.^[2]^ This sparked significant efforts from both academic and industrial groups, resulting in the development of numerous modern synthetic methods with LSF applications in mind.[^1a^, ^1b^, ^3^] Given the nature of the substrates, among the most desirable transformations are the introduction of functional groups such as methyl, amino, hydroxy, and fluoro, as well as other small groups as these would allow smaller steric perturbation of the bioactive compound.^[2a]^ On the other hand, the introduction of larger groups, in form of synthetic handles, can facilitate rapid access to conjugation chemistry. Although examples of such applications remain scarce,^[4]^ with the late‐stage C−H functionalization approach an already available hit compound can be rapidly transformed into conjugates for targeted drug deliver (small molecules, peptide and antibody drug conjugates),^[5]^ conjugates enabling novel mechanisms of action (proteolysis‐targeting chimeras (PROTACs))^[6]^ or even rapidly accessing probes for chemical biology (fluorescent and radiolabeled probes).^[7]^ This presents a significant advantage over common strategies for bioconjugation, which rely on prefunctionalization of the small‐molecule payload with reactive functional groups, thus again necessitating de‐ novo synthesis of analogues. In general, methodologies applicable for LSF must tolerate polar functional groups such as amines, alcohols, hydrogen bond donors and acceptors, heterocycles, basic as well as weakly acidic sites frequently present in drug‐like molecules. In terms of key disconnections, C−H bond functionalization is of special interest due to the abundance of C−H bonds in drug‐like molecules. The classical approach is represented by the historically well‐established electrophilic aromatic substitution (S_E_Ar) tactic (Figure 1A). The free amine would typically be introduced by nitration and subsequent reduction of the nitro analogue. While nitration is performed under strong acidic conditions,^[8]^ recent developments of mild nitration methodologies allow for the functionalization of complex drug‐like molecules.^[9]^ Alternatively, S_E_Ar halogenations followed by amination can also yield the desired free amino analogues. Similar to the nitration, mild halogenation reactions have been developed.^[10]^ Furthermore, transition‐metal catalyzed crosscoupling reactions of aryl halides to construct C−N bonds are well stablished.^[11]^ Finally, a Rh‐catalyzed electrophilic arene amination with similar regioselectivity has also been reported.^[12]^ Recent years have also seen developments in radical C−H aminations. Systems utilizing iron catalyst reported by Ritter^[13]^ and Morandi^[14]^ afford the free amine products directly from the corresponding C−H starting materials. A mechanistically distinct reactions utilizing photoredox catalyst were reported by Nicewicz,^[15]^ Ritter,^[16]^ Togni and Carreira.^[17]^ The regioselectivity in these electrophilic and radical substitution methodologies is governed by the electronic and steric properties of the substrates. As multiple suitable reaction sites are present in complex drug‐like molecules, this can result in the formation of regioisomeric mixtures and multiple functionalization products (Figure 1A). On one hand this can be desirable, as obtaining multiple analogues can prove beneficial for the drug discovery process. On the other hand, such mixtures, especially when only varying in the position of a small substituent, may be difficult or even impossible to purify. A complementary strategy in terms of regiochemical outcome lays within directed C−H activations (Figure 1B). The regioselectivity in this case is governed by Lewis basic coordinating groups, typically present in the drug‐like molecules, in combination with transition metal catalysts.^[18]^ Methodologies for transition metal‐catalyzed C−H aminations of small molecules and building blocks have been compiled in reviews.[^18b^, ^19^] Within directed C−H aminations, the wealth of work from Chang and co‐workers deserves a special mention. The group has over the years not only developed a large number of C−N bond‐forming reactions utilizing a number of different directing groups,^[20]^ but have also reported valuable insights into the mechanistic aspects of these transformations,[^20e^, ^21^] thus aiding further developments in the field. The directed C−H activation approach can however also result in the formation of regioisomeric mixtures, as multiple coordinating groups and C−H bonds suitable for activation are typically present, particularly in highly functionalized drug‐like molecules (Figure 1B). Although “designer” directing groups can provide the desired regioselectivity and conversion, the use of these is not ideal from an atom‐ and step‐economy point of view. In an ideal scenario the regioselectivity would be dictated by a single innate directing group already present in the molecule (Figure 1C). From this perspective, the carboxylic acid functional group is of special interest. Benzoic acids are an important structural motif frequently occurring not only in drugs, but also building blocks, fragment‐based lead discovery and hit discovery compounds.^[22]^ Our group has previously reported regioselective *ortho*‐directed iodinations,[^23^, ^24^] methylations and [D_3_]methylations of benzoic acids enabled by the use of Cp*Ir^III^ catalysts and HFIP as solvent,^[25]^ with the latter successfully applied to the late‐stage functionalization of a number of pharmaceuticals. The importance of the benzoic acid structural motif in medicinal chemistry and the potential high utility of its *ortho*‐aminated analogues prompted us to develop the herein presented method. We report a mild iridium‐catalyzed *ortho‐*selective C−H amination of benzoic acids. Late‐stage sulfonamidation of structurally diverse marketed drugs and drug‐like compounds has been achieved in a controlled and predictable fashion. The predictability of the reaction outcome in respect of functional group tolerance is further aided by a comprehensive functional group tolerance study. The method was further extended to grant access to valuable free amine analogues. Finally, the methodology was applied to previously unexplored diversifying conjugation, enabling rapid access to valuable new modalities compounds,^[26]^ as well as probes for chemical biology.

## Results and Discussion

### Optimization

We started our study with *m*‐toluic acid (**1 e**) as a model substrate, *p*‐toluenesulfonyl azide (TsN_3_) as the −NHTs source,^[20c]^ and Cp*Ir(H_2_O)_3_SO_4_ as the catalyst (Table 1A). The reagent selection was especially important, as among our goals was to develop a method applicable to high‐throughput experimentation (HTE) and automation.^[27]^ As the catalyst, we selected Cp*Ir(H_2_O)_3_SO_4_ instead of [Cp*IrCl_2_]_2_, used in our previous work,[^23^, ^25^] to omit the use of insoluble Ag^I^ salts. The use of insoluble reagents was identified as a limiting factor for HTE and miniaturization, as this limits the use of stock solutions and poses potential reproducibility issues in absence of stirring. In the initial stage, a screening of 24 solvents under two sets of conditions was performed, with and without 1 equiv. Et_3_N (Table 1A). The additive was chosen based on our previous research,^[23]^ where the presence of base in a similar system was necessary for achieving good conversions. Using 1,1,1,3,3,3‐Hexafluoroisopropanol (HFIP) as the reaction solvent proved to be crucial for achieving the desired transformation with good conversions.^[24]^ The vast majority of other solvents screened showed no conversion or only traces of product. The best result was obtained in HFIP as solvent with Et_3_N as additive (Table 1, entry F3), with 77 % conversion observed. In the next round of optimization, we investigated the catalyst loading (Table 1B). The goal was to keep the loading low, while having good conversion with various substrates, providing standard conditions applicable for a wider substrate scope. A screening of 16 reaction conditions was conducted, with four substrates tested against iridium loadings ranging from 0.5 mol % to 3 mol % (Table 1B). While fair to excellent conversions were observed already with only 0.5 mol %, 3 mol % loading was chosen for the reaction scope investigation, as under these conditions full conversion was observed for three out of four substrates tested.

**Table 1:**
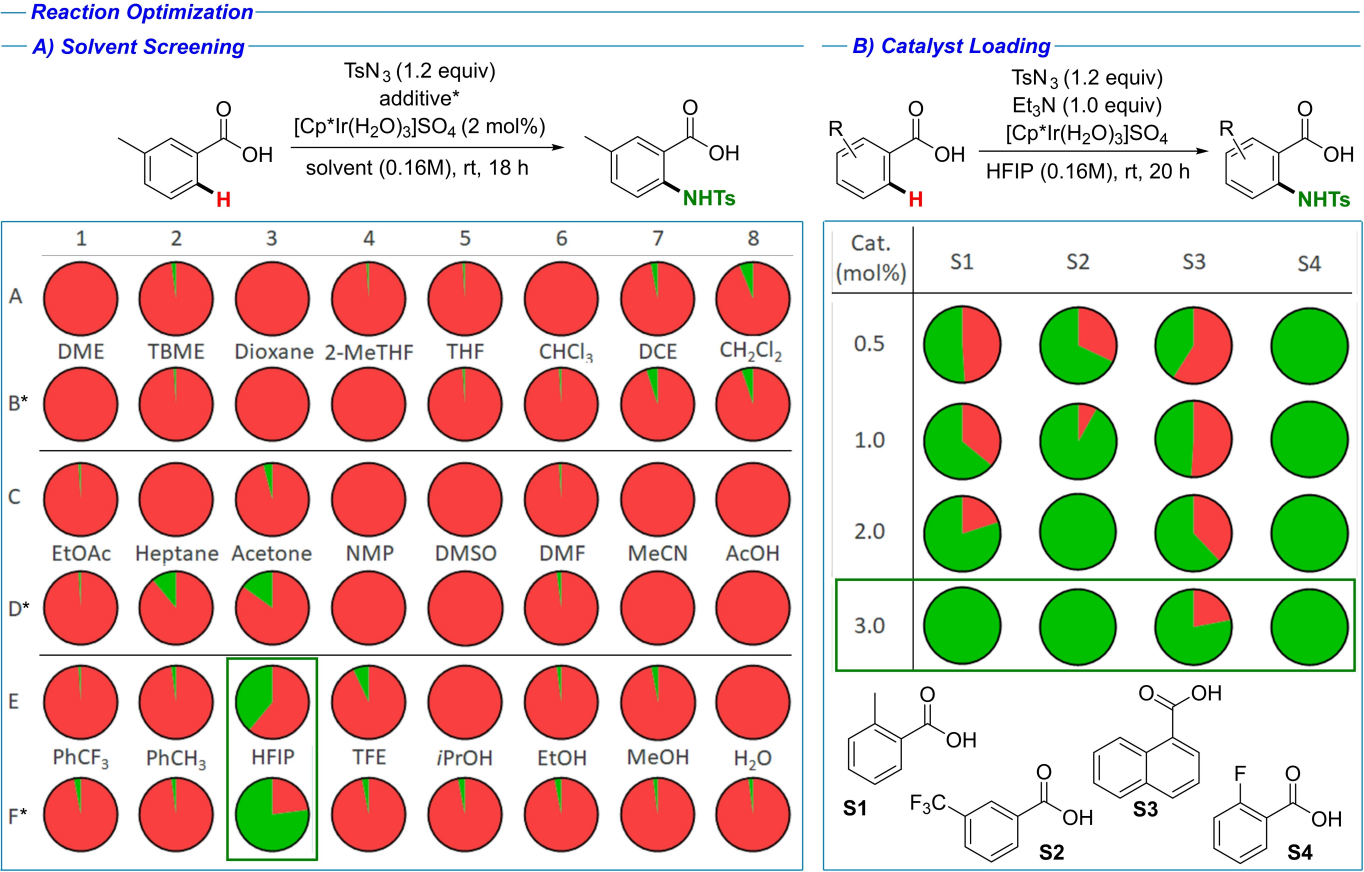
Reaction optimization. Red: % of unreacted starting material; Green: % product. Ratios established from UV trace by SFC‐MS. A) Solvent screening. In total 24 solvents were tested. * indicates reactions with Et_3_N (1.0 equiv.) B) Catalyst loading optimization. In total 4 substrates were tested against an increasing catalyst loading.

### Functional group compatibility

To support the late‐stage amination with a predictive tool for functional group and reagent compatibility, the effect of 53 different compounds on the reaction outcome was studied (Table 2).^[28]^ The goal was to identify functional groups that inhibit the C−H amination reaction, providing predictive power to evaluate the potential success of the reaction prior to being performed. Out of the 54 additives tested (H_2_O tested at two distinct loadings), conversion of >50 % was observed in 31 cases, 25–50 % in 7 cases and <25 % in 16 cases. Lewis basic groups commonly found in drug‐like molecules were in general well tolerated, including amides, ketones, esters, and even ureas. Protic functional groups and Brønsted acids, such as alcohols and carboxylic acids, were also compatible. A number of polar and/or protic solvents, including DMF, NMP, 2‐MeTHF and water, did not inhibit the reaction substantially. The presence of functional groups used as synthetic handles in cross coupling chemistry, that is, boron‐ or halide‐functionalized compounds, did not interfere with the reaction outcome. The limitations observed are as follows: 1) coordinating solvents such as DMSO and MeCN were not compatible with the reaction, possibly due to inhibition of the catalyst; 2) the presence of primary amines, both sp^2^ and sp^3^ is not tolerated, 3) nitrogen‐containing strongly coordinating heterocycles such as pyridine retard the reaction, however, introduction of steric bulk around the heteroatom and/or decreasing the electron density restores reactivity, 4) unhindered alkynes and alkenes are not tolerated, however, more sterically hindered alkenes such as norbornene or the Michael acceptor systems in progesterone were well tolerated.

**Table 2:**
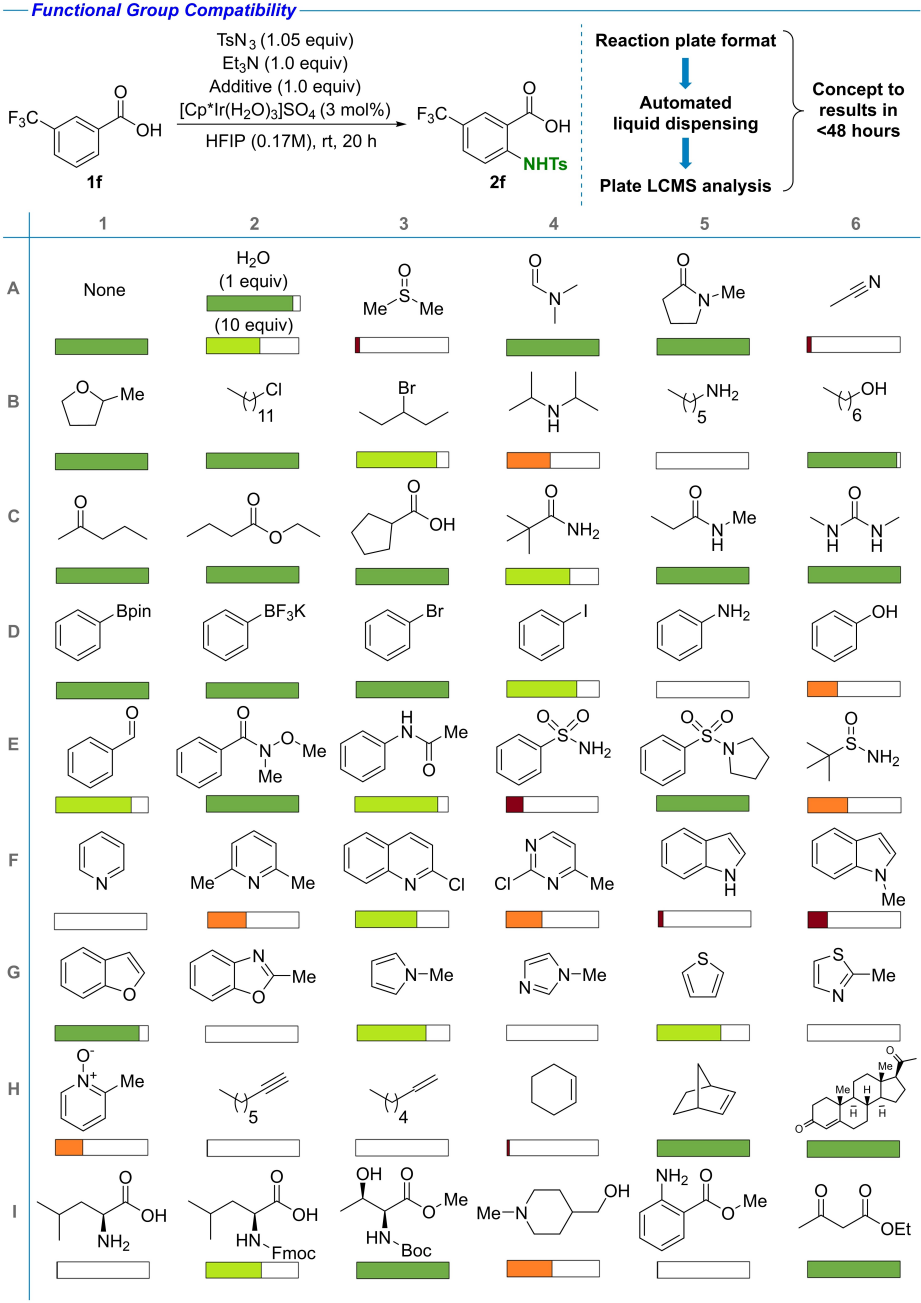
Functional group tolerance. The additive compatibility was quantified based on the conversion to the desired product, depicted by the colored bars (green >50 %; orange 25–50 %, red <25 %).

### Substrate scope and limitations

**Scheme 1:**
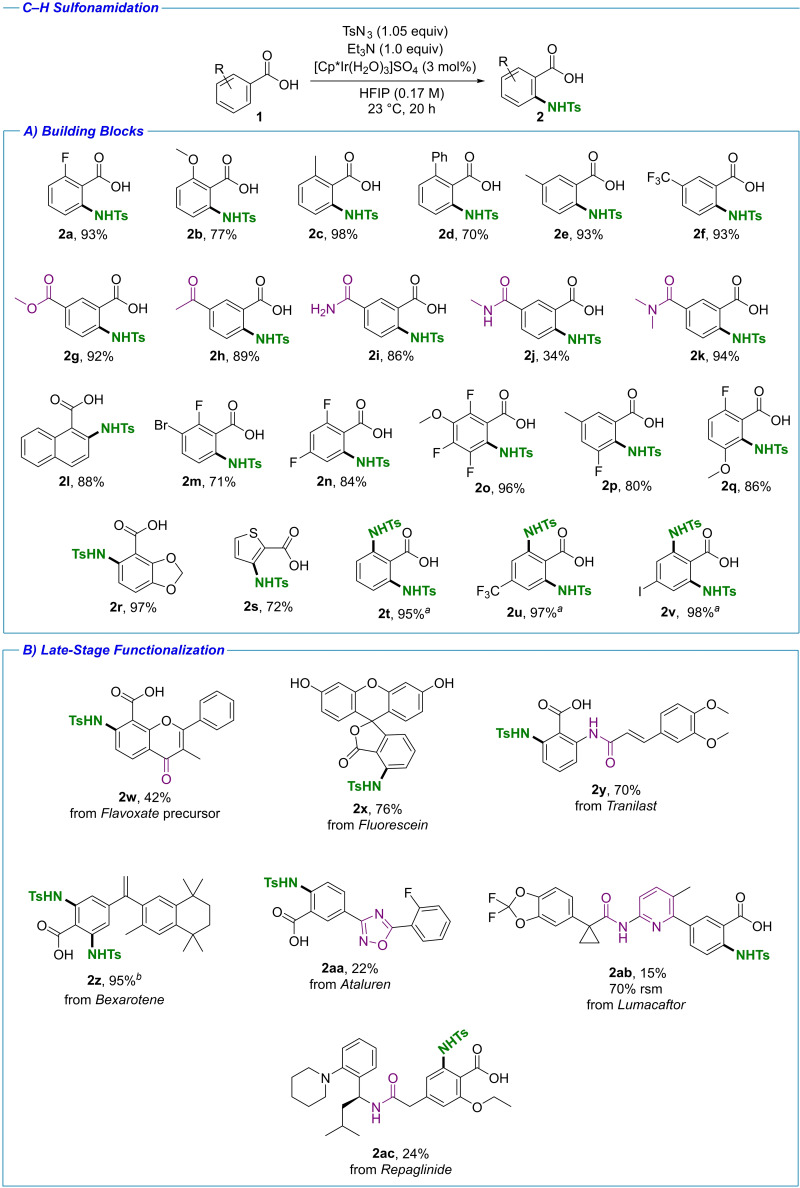
C−H sulfonamidation scope. Isolated yields shown. A) Scope of building blocks. [a] TsN_3_ (2.1 equiv.) was used. B) Scope of LSF. [b] TsN_3_ (2.1 equiv.), KOAc (1.0 equiv.), [Cp*Ir(H_2_O)_3_]SO_4_ (6 mol %). Other potential directing groups are highlighted in purple.

With the optimized reaction conditions in hand and a good understanding of the functional group compatibility, we embarked on investigating the substrate scope and limitations. In the first stage a series of diversely decorated benzoic acids were functionalized under standard conditions (Scheme 1A). *Ortho* substituents were well tolerated. Compounds **2 a**, **2 b** and **2 c** with small *ortho* substituents, and even compound **2 d** with the larger phenyl substituent were obtained in good to excellent yields. Substituents in the *meta* position were also tolerated, as shown in the preparation of **2 e** and **2 f**, demonstrating also applicability to electronically diverse substrates. In all cases the desired products were obtained with complete regioselectivity in a predictable manner: *ortho*‐ to the carboxylate directing group on the less sterically hindered site. We also investigated the reaction outcome with substrates bearing two directing groups.^[18c]^ No interference with the anticipated regioselectivity was observed in the presence of these groups; The ester functionality was tolerated, with **2 g** obtained as a single regioisomer in excellent yield. Similarly, the enolizable ketone in **2 h** was compatible with the reaction. In the amide series, all types of amides, primary, secondary and tertiary, were well compatible with the reaction conditions and provided the expected product as a single regioisomer. While a lower yield was observed with the secondary amide in **2 j**, the remaining mass balance consisted of unreacted starting material, and no other regioisomers were obtained. A series of more heavily decorated substrates were also tested, yielding compounds **2 l**–**2 q** in good to excellent yields. 1‐Naphthoic acid was functionalized at the 2‐position providing **2 l**, with complete selectivity over the 8‐position of the neighboring ring. Systems bearing multiple substituents were successfully functionalized, yielding products with a 1,2,3,4‐substitution pattern in **2 m** and a 1,2,3,5‐substitution pattern in **2 n**. We also investigated the possibility of introducing the sulfonamide moiety in the 2‐position, between the carboxylate directing group and another functional group in the 3‐position. We observed that this was only possible with substrates bearing small Lewis‐basic substituents in the 3‐position. A fully substituted benzene ring was successfully prepared as **2 o** in excellent yield, with a fluorine in the 3‐position. When two *ortho* positions were available, functionalization occurred with complete selectivity in the vicinity of the fluorine substituent, as demonstrated with compound **2 p**.^[25]^ The sulfonamide could also be introduced in the vicinity of a methoxy substituent in **2 q**. Functionalization of heterocycles is exemplified with the benzenedioxole **2 r**, and the five‐membered thiophene **2 s**. Functionalization of *para‐*substituted benzoic acids was complicated by the formation of inseparable mixtures of mono‐ and difunctionalized products under standard conditions. The reactions could however reach complete difunctionalization by simply increasing the azide loading to 2.1 equiv., as shown in the synthesis of **2 t**–**2 v**. In all the presented cases complete regioselectivity for challenging functionalization *ortho* to the carboxylic acid functional group was achieved, thus presenting a method complementary to electrophilic aromatic substitutions, where *meta*‐C−N bonds are formed with benzoic acids. In the next stage we turned our attention to late‐stage functionalizations of drug‐like molecules. A series of compounds depicted in Scheme 1B was subjected to the standard reaction conditions. It is important to note that no prefunctionalization or protecting group introduction was needed. The Flavoxate precursor was functionalized in the anticipated position yielding **2 w** in a synthetically useful yield. While the ketone functionality is in plain with the neighboring C−H functionality and could theoretically serve as a good directing group, no functionalization directed by this group was observed. Fluorescein, a widely used fluorescent tracer,^[29]^ was functionalized in the anticipated position with complete regioselectivity. While the product **2 x** is depicted in its ring‐closed lactone form as observed by ^1^H NMR spectroscopy, the corresponding starting material would be present in an equilibrium with a ring‐opened form containing a free carboxylate facilitating the directed C−H activation. Noteworthy is the presence of phenolic hydroxy groups and quinone moiety in the ring open form, both of which were well tolerated under the presented conditions. Although phenol was shown to significantly decrease conversion in our functional group compatibility study (Table 2), the phenolic OH groups were better tolerated with this particular substrate, possibly owing to electronic differences between the two compounds, and **2 x** was obtained in a very good 76 % yield. Tranilast, an anti‐allergic drug used also for the treatment of a variety of other indications,^[30]^ was functionalized with complete regioselectivity at the *ortho* position, yielding compound **2 y** in 70 % yield. The acylanilide functionality present did not serve as a competing directing group in the reaction. The Michael acceptor vinylic system also posed no issues in terms of reaction compatibility. The functionalization of Bexarotene, an antineoplastic agent used for the treatment of cutaneous T‐cell lymphoma (CTCL),^[31]^ under standard conditions resulted in a formation of a mixture of mono‐ and di‐functionalized products and unreacted starting material, which we were unable to separate. Under slightly modified conditions, increased catalyst and azide loadings, and adding KOAc (Scheme 1B), conversion to the difunctionalized **2 z** was achieved in excellent yield (95 %). The alkene functionality, flagged as potentially detrimental to the reaction (Table 2), was well tolerated in this case, most likely owing to the alkene substitution pattern. We were especially pleased with the functionalization of heterocycle‐containing Ataluren, a therapeutic agent developed for the treatment of Duchenne muscular dystrophy.^[32]^ While compound **2 aa** was obtained with a modest yield, this example was important as it showcased not only compatibility with the oxadiazole heterocycle, but also demonstrated complete regioselectivity for the predicted C−H bond over the other three C−H bonds suitable for directed activation. Similarly Lumacaftor, a therapeutic agent used in the treatment of cystic fibrosis,^[33]^ was functionalized, tolerating the phenylpyridine and anilide Lewis basic moieties, yielding the anticipated product **2 ab** with complete regioselectivity. The results obtained for the heterocycle‐containing compounds are in line with our predictions based on the functional group compatibility study (Table 2), as sterically hindered heterocycles are anticipated to be tolerated, albeit with expected negative impact on yield. Finally Repaglinide, used for blood glucose reduction in type 2 diabetes mellitus,^[34]^ was selectively functionalized at the anticipated position as compound **2 ac**, with the tertiary amine and secondary amide functionalities well tolerated.

**Scheme 2:**
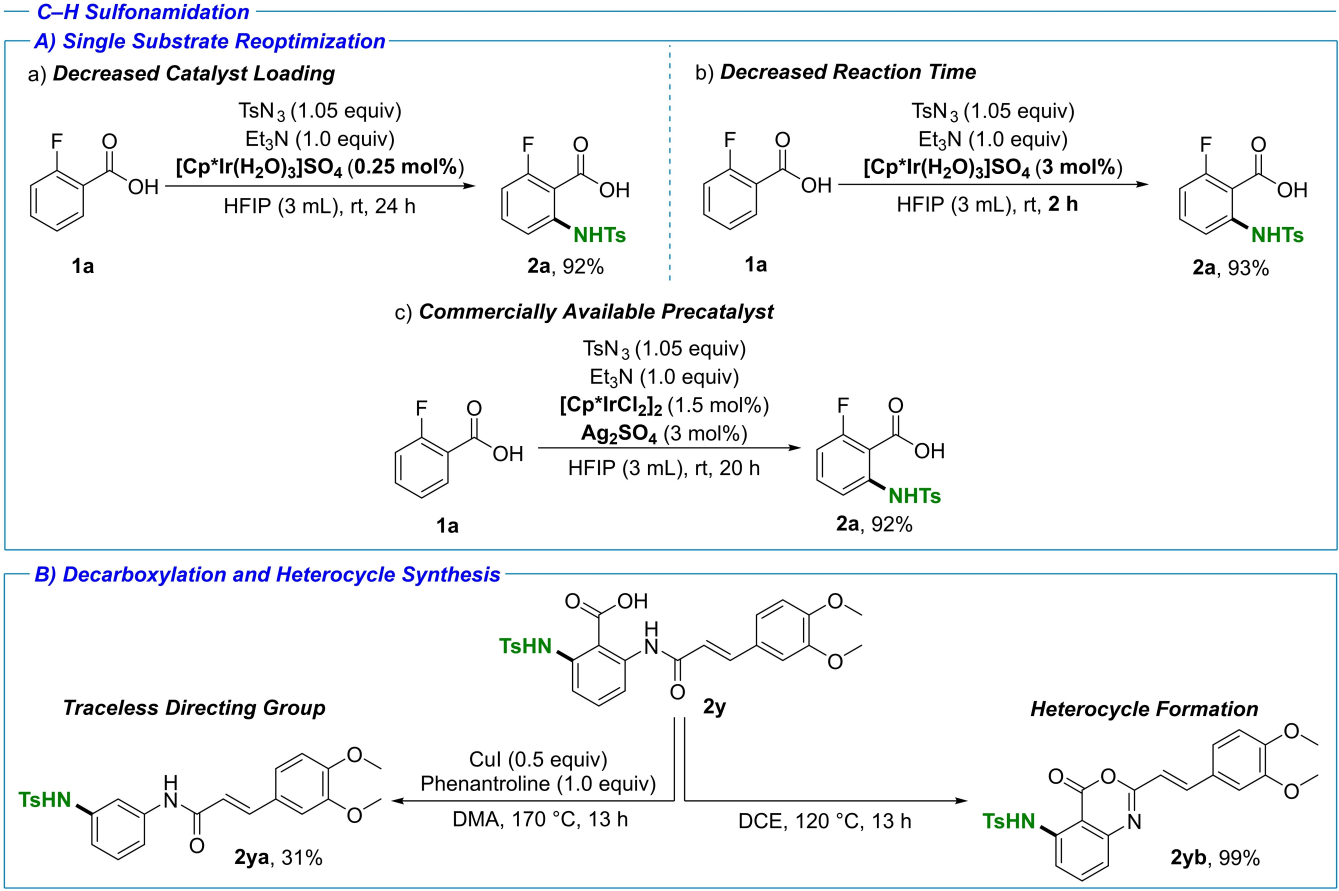
Additional investigations; isolated yields are shown. A) Single substrate reoptimization. a) Lowest catalyst loading at 0.25 mol %. b) Decreased reaction time (2 h) at 3.0 mol % catalyst loading. c) Use of commercially available [Cp*IrCl_2_]_2_ catalyst. B) Decarboxylation studies. Anticipated decarboxylation was achieved for copper‐mediated decarboxylation (left), whereas an unexpected heterocycle was formed when the mixture was heated in 1,2‐dichloroethane (DCE).

While the development of a single set of reaction conditions applicable for a variety of substrates was our initial goal, we also investigated the possibility of fine‐tuning the reaction conditions for a single substrate (Scheme 2A). With the 2‐fluorobenzoic acid model system, full conversion was observed with a catalyst loading as low as 0.25 mol %. On the other hand, the reaction time required for full conversion could be reduced to 2 h with a 3 mol % catalyst loading. We also demonstrated the applicability of the commercially available [Cp*IrCl_2_]_2_ for the transformation, with addition of Ag_2_SO_4_ for in‐situ catalyst activation. Under these conditions stirring proved crucial for consistently obtaining full conversion (92 % yield).

The use of carboxylates as directing groups presents another attractive synthetic strategy namely utilization of the acid as a traceless directing group. The use of benzoic acids for obtaining 1,3‐ and 1,4‐substituted anilines has been pioneered by the Chang and co‐workers,^[20a]^ however, no such application to complex drug‐like molecules has been reported to the best of our knowledge. Using Tranilast as the model system, under copper‐catalyzed conditions the formation of the envisioned decarboxylated compound **2 ya** was observed (31 % yield, Scheme 2B). Interestingly, we also observed quantitative formation of the 1,3‐benzoxazin‐4‐one core in **2 yb** resulting from a dehydration of the starting material upon heating in DCE. The demonstrated rapid access to three distinct analogues of a single compound further strengthens the utility of the presented C−H sulfonamidation in terms of late‐stage diversification. Such approach has the potential to greatly aid structure–activity relationship (SAR) studies by modification and/or complete removal of binding structural motifs.

### Synthesis of free amines

**Scheme 3:**
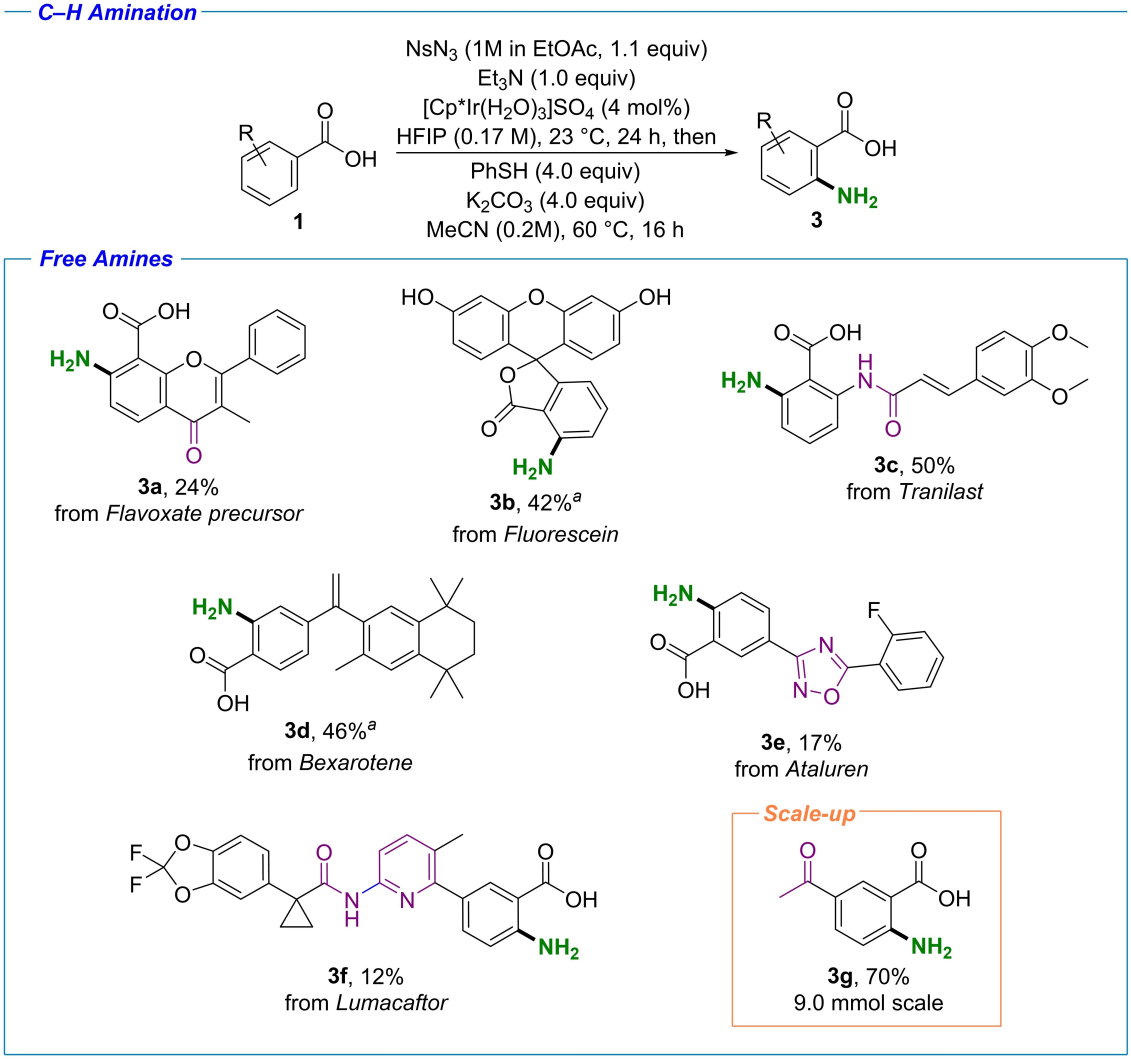
C−H amination; isolated yields are shown. [a] Reaction time in first step 44 h. Other potential directing groups are highlighted in purple.

While the synthesis of tosylamide analogues gave valuable insights into reactivity and selectivity trends of the methodology, we were especially interested in the formation of free amines, as this transformation is highly desirable in the LSF context. For this transformation to be successful, the formation of a protected amine which would be possible to deprotect under conditions compatible with a wide variety of functional groups present in drug‐like molecules was sought after. Unfortunately, the tosylamide functionality was deemed unsuitable for this purpose, as the methods for tosyl cleavage were considered too harsh for complex molecules. We decided to switch to the corresponding nosylamides, which can be conveniently deprotected with PhSH and a weak base (Scheme 3). As nosylazide (NsN_3_) required for this transformation was flagged as a high energy compound after literature search,^[35]^ we wanted to exercise caution while using the reagent. Thus, a protocol for the preparation of the ethyl acetate solution of the compound was used, avoiding the formation of the crystalline material. The amination protocol developed involves a sequential C−H functionalization, followed by a solvent swap and nosyl deprotection (Scheme 3). While slightly lower conversions to the nosylamide were observed compared to the corresponding tosylamide analogues, we were pleased to see that the nosyl protecting group was possible to cleave under standard deprotection conditions without compound decomposition throughout the substrate scope. The Flavoxate precursor was successfully converted into the corresponding free amine analogue **3 a** (Scheme 3). The Fluorescein amino analogue **3 b** was also obtained, with the deprotection methodology tolerated by the potentially reactive quinone ring‐opened form of Fluorescein. The anticipated amine analogue of Tranilast **3 c** was obtained in a fair yield, once again with complete regioselectivity. Interestingly, while the conversion of Bexarotene to the corresponding nosylamide was modest, high selectivity for the monofunctionalized product was observed, yielding compound **3 d** in 46 % yield. The heterocycle‐containing Ataluren **3 e** and Lumacaftor **3 f** were successfully aminated at their respective anticipated positions. Finally, the possibility of scaling up the reaction was demonstrated by synthesizing the previously unreported anthranilic acid **3 g**, which was obtained with a 70 % isolated yield at a 9.0 mmol scale. At this point it is important to mention that in all presented cases the regiochemistry obtained with our method grants access to aminated analogues not accessible by means of electrophilic aromatic substitutions or radical aminations.[^9^, ^13^, ^14^, ^17^, ^36^]

### Synthesis of conjugates

**Scheme 4:**
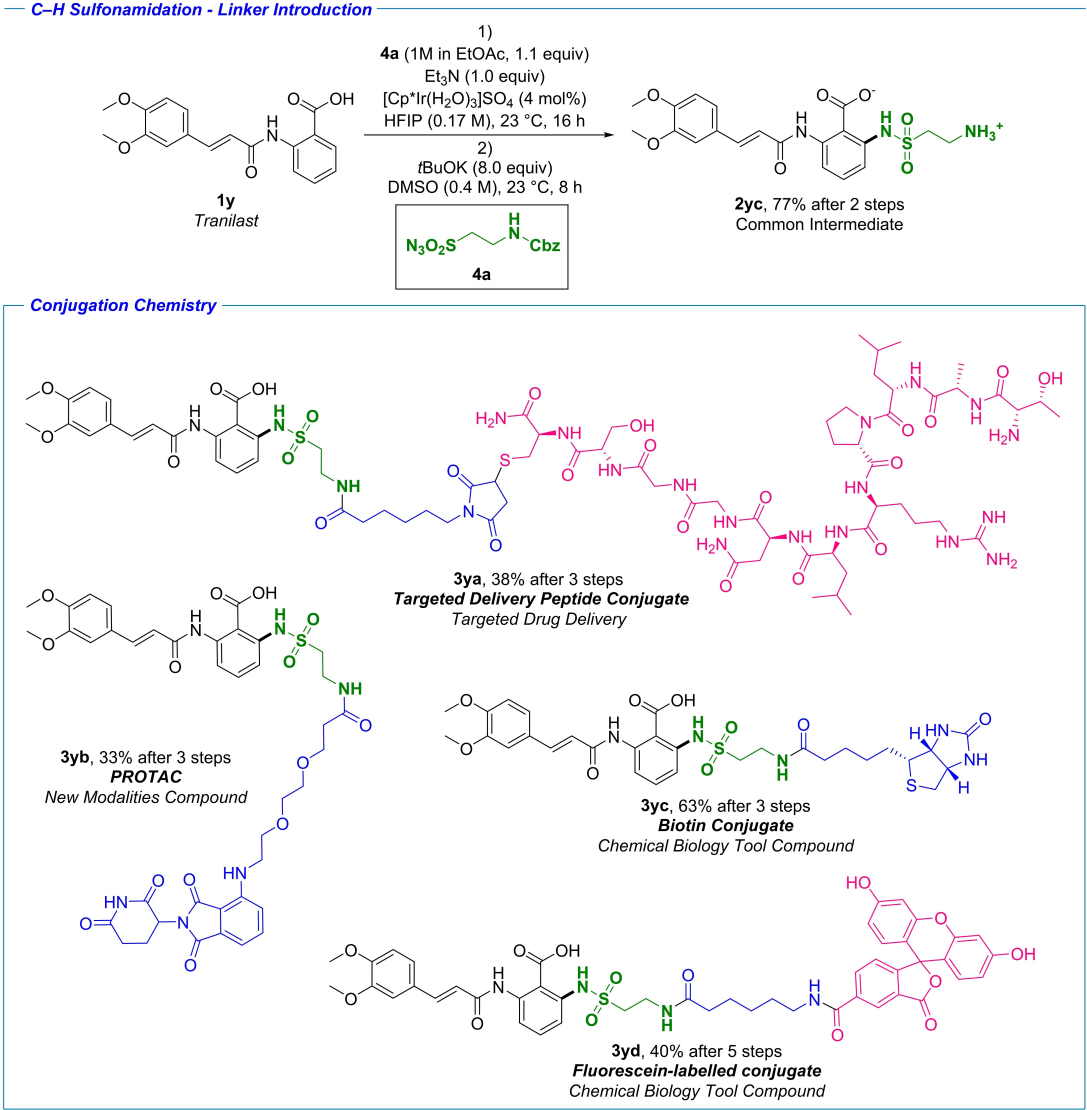
Synthesis of drug conjugates. The key intermediate was prepared in a two‐step protocol in 77 % overall yield; isolated yields are shown. The yields and step count depicted are from commercially available Tranilast. Each color represents a structural motif added in a new transformation. For detailed reaction conditions, see the Supporting Information.

Our final goal in terms of late‐stage C−N bond formation was the introduction of synthetic handles for conjugation chemistry, directly applicable for the development of so‐called new modalities. New modalities have over the past decades become a viable and important class of therapeutics, complementing small‐molecule drugs and biologicals.[^26^, ^37^] Modalities containing small‐molecule drug moieties such as peptide– and antibody–drug conjugates (PDCs and ADCs), small‐molecule oligonucleotide conjugates, and PROTACs, have attracted considerable interest from both industry and academia, which has already yielded therapeutics in clinical use.^[26]^ These modalities hold great potential not only for targeted delivery of therapeutics to specific cells or tissues, but can also widen the therapeutic window for molecules with otherwise unsuitable safety profile. Moreover, they could potentially unlock therapeutical targets deemed undruggable by more traditional approaches. In their 2017 review, Valeur et al. called for new, creative approaches to branch out from traditional strategies to discover new chemical space within new modalities.^[26]^ C−H functionalization presents a logical, yet relatively unexplored approach in this respect. A bifunctional azide reagent was designed and prepared for the envisioned C−H sulfonamidation. Azide **4 a** (Scheme 4) was prepared from the corresponding commercially available sulfonyl chloride (see the Supporting Information). The choice of the amine group for linker functionalization was deliberate,^[38]^ allowing for a plethora of further derivatizations, such as robust urea, thiourea and amide formations, reductive aminations, and transition metal‐catalyzed cross‐couplings. The Cbz protecting group was chosen as it allows for a number of different deprotection methodologies, which was proven crucial for our model system. Tranilast was chosen as the model system for this investigation. The Cbz‐protected amine was well tolerated under the reaction conditions, and similar to the previous results, the desired product was obtained with complete regioselectivity. Flexibility in deprotection was crucial, as we did not succeed to cleave the Cbz group under standard hydrogenation conditions due to extremely low solubility of the starting material. The alternative base‐mediated deprotection provided the common intermediate with 77 % yield after two steps (Scheme 4). With this intermediate in hand we proceeded to demonstrate the applicability of our methodology in conjugation chemistry. In the first example, the peptide conjugate **3 ya** was prepared in a one pot sequential procedure from the common intermediate **2 yc**. First the amide bond formation with the NHS ester of the maleimide linker was set, followed by Michael addition of the cysteine side chain of the fully unprotected peptide to the maleimide synthetic handle. The desired peptide conjugate was obtained in a 38 % overall yield starting from commercially available Tranilast. The peptide tag chosen for this compound was previously used for targeted delivery of liposomes in the context of myocardial infarction.^[39]^ The late‐stage formation of a targeted‐delivery conjugate exemplified here presents an attractive strategy for rapid preparation and evaluation of the feasibility of targeted delivery of hit and lead compounds. PROTACs have attracted considerable attention within both academic and industrial communities over the past years.^[40]^ The PROTAC compounds themselves are bifunctional small molecules consisting of two active domains and a linker. Tranilast was successfully converted into PROTAC compound **3 yb** in only three steps with an overall 33 % isolated yield. This example demonstrates the possibility for rapidly accessing complex molecular conjugates with different modes of action from hit and lead compounds, further aiding the drug discovery process. Elucidation of the mode of action of biologically active small molecules is another key objective within drug discovery and chemical biology.^[41]^ Among widely utilized methods for target validation is affinity chromatography, through attachment of a small‐molecule probe to biotin.^[41]^ Regioselective C−H activation presents an attractive approach in this context, as the biotinylated handle can be attached in the predetermined position with minimal impact on the drug's binding affinity, and without the risk of functionalizing the polar functional groups necessary for binding. A biotinylated Tranilast analogue was prepared in a three‐step protocol, providing compound **3 yc** in a 63 % overall yield. Small‐molecule‐based fluorophore–drug conjugates represent a valuable class of tool compounds for real‐time monitoring of drug delivery and distribution.^[7a]^ The synthesis of molecular imaging tool compounds was exemplified with the Fluorescein‐tagged compound **3 yd**. With this particular synthetic pathway, we also sought to demonstrate the modular approach to building up linkers, with the two amide bonds in the final linker being set in consecutive steps. This application further demonstrates the versatility of the common intermediate **2 yc** in the preparation of small‐molecule drug conjugates by late stage diversification.

### Mechanistic studies

**Scheme 5:**
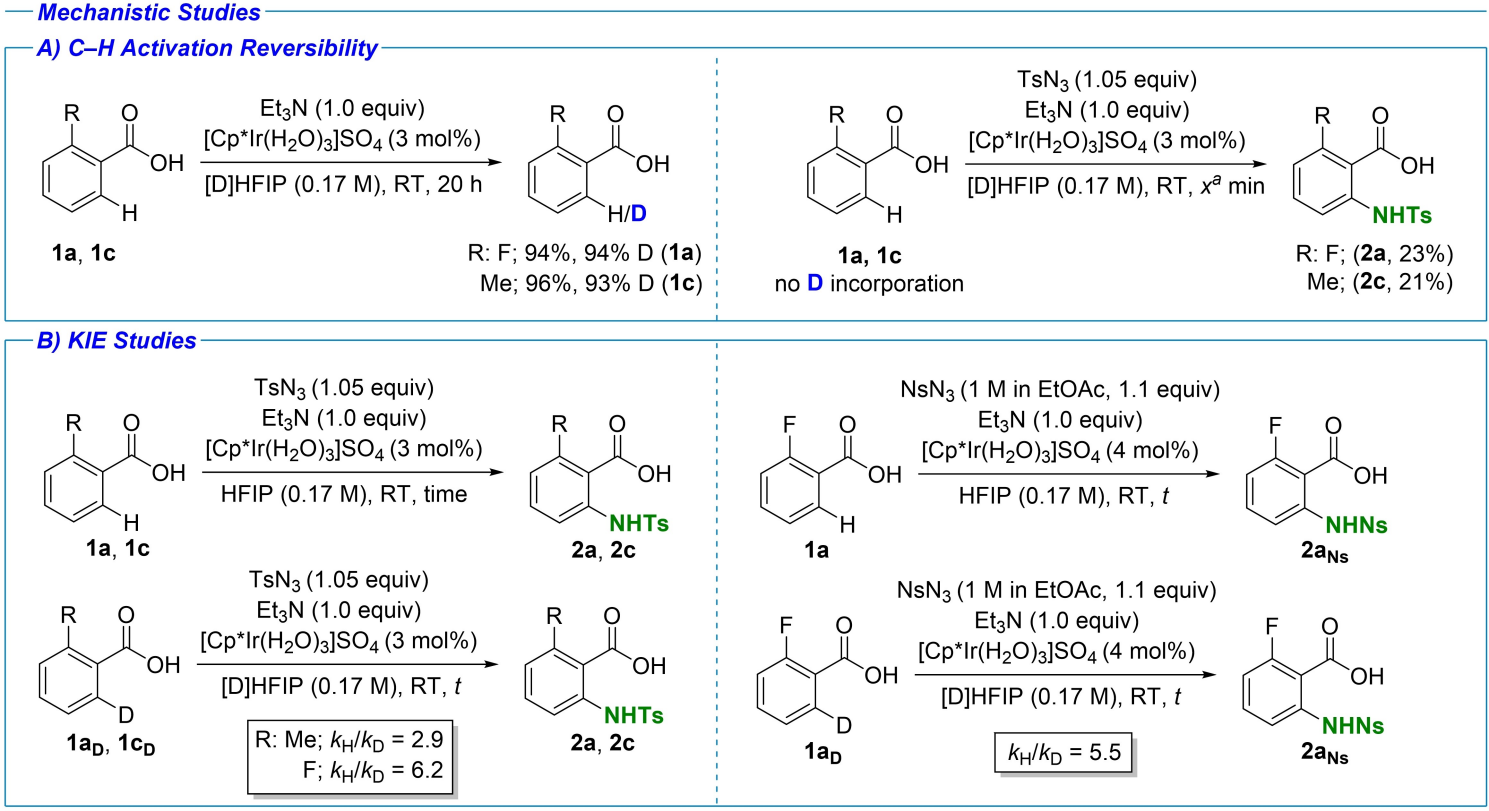
Mechanistic studies. A) C−H activation reversibility. Left: reversibility of C−H activation confirmed in the absence of azide source. Right: C−H activation shown to be irreversible in the presence of an azide source. No deuterium incorporation into the starting material was observed. [a] R=Me, 15‐min reaction time; R=F, 8‐min reaction time. B) KIE studies. Left: Parallel experiments with H and D compounds, initial rates measured (see the Supporting Information), TsN_3_ as azide source. R=Me k_H_/k_D_ of 2.9 measured. R=F k_H_/k_D_ of 6.2 measured. Right: NsN_3_ as azide source. k_H_/k_D_ of 5.5 measured.

A mechanistic study was conducted in order to gain deeper understanding of the underlying processes in the presented methodology. The reversibility of the C−H activation was studied using two *ortho*‐substituted substrates (Scheme 5, **1 a** and **1 c**) in [D]HFIP as solvent.^[23]^ In the absence of the azide source, high levels of deuterium incorporation were observed with both substrates. However, when the azide source was added and the reaction stopped after a short time, no deuterium incorporation was observed in the remaining starting materials. This result suggests that the C−H activation step is reversible in the absence of the azide reagent, but not under the amination conditions. A kinetic isotope effect (KIE) investigation was also undertaken with three sets of conditions. First, the KIE for two *ortho*‐substituted substrates was studied with TsN_3_ as azide source. The reactions of non‐deuterated (**1 a** and **1 c**) and deuterated (**1a_D_** and **1c_D_**) starting materials were run in a parallel set‐up. For *o*‐toluic acid a *k*_H_/*k*_D_ value of 2.9 was measured, while for the *o*‐fluorobenzoic acid the *k*_H_/*k*_D_ value of 6.2 was recorded. Similar outcome was observed when NsN_3_ was used as the azide source. A *k*_H_/*k*_D_ value of 5.5 was recorded for *o*‐fluorobenzoic acid under these conditions. The *k*_H_/*k*_D_>1 observed in all cases suggests a turnover‐limiting C−H activation step.^[42]^

## Conclusion

For the first time, we have developed a robust and predictable iridium‐catalyzed C−H *ortho*‐amination for the late‐stage sulfonamidation and amination of complex benzoic acid derivatives. The reaction demonstrates high selectivity for carboxylate‐directed functionalization, even in presence of multiple strongly coordinating directing groups. Importantly, the use of soluble reagents and catalyst, tolerance to air and moisture, and no requirement for heating and stirring makes the method ideal for chemistry automation and applications in high‐throughput experimentation. In terms of regioselectivity, the method presents a valuable addition to established electrophilic and radical aromatic substitutions. The demonstrated high functional group tolerance, in combination with the functional group compatibility study, allows good predictability of reaction success, which is especially important for LSF applications. This was showcased by the preparation of a series of anthranilic acid analogues from marketed drugs. Furthermore, the unique features of the catalytic system enabled the rapid synthesis of drug conjugates through C−H activation, a mostly unexplored synthetic approach in this respect, facilitated by the introduction of a sulfonamide synthetic handle designed for the purpose. Although the concise synthesis of a number of exciting, highly complex new modalities compounds and tool compounds for chemical biology enabled by the directed C−H sulfonamidation tactic was demonstrated in this report, this by no means exhausts the possible applications of late‐stage conjugation strategies within these continuously growing fields. It is our firm belief that this practical approach to C−H amination, sulfonamidation, conjugation and bioconjugation chemistry will be of immediate use to medicinal chemists, and will aid and streamline the drug‐discovery process when applied.

## Experimental Section

**General synthesis of −NHTs analogues**: [Cp*Ir(H_2_O)_3_]SO_4_ (7.3 mg, 0.015 mmol) was weighed in a 16 mL glass scintillation vial. In a separate vial substrate (0.50 mmol) was added, followed by addition of HFIP (3 mL) and Et_3_N (69.7 μL, 0.50 mmol). The mixture was briefly sonicated until a solution was formed, which was then added to the weighed in catalyst. To this mixture TsN_3_ (80.5 μL, 0.53 mmol) was added, and the mixture was briefly shaken. The reaction mixture was let to stand at room temperature for 20 h. The mixture was then transferred to a phase separator containing 1 M aq. HCl (50 mL) and EtOAc (50 mL). The organic phase was collected and the aqueous phase was extracted with EtOAc (3×40 mL). The combined organic fractions were washed with brine (40 mL), dried over MgSO_4_ and concentrated under reduced pressure. The purification of the products is described in each experiment entry separately.

**General synthesis of −NH_2_ analogues**: [Cp*Ir(H_2_O)_3_]SO_4_ (9.7 mg, 0.02 mmol) was weighed in a 16 mL glass scintillation vial. In a separate vial substrate (0.50 mmol) was added, followed by addition of HFIP (2.5 mL) and Et_3_N (69.7 μL, 0.50 mmol). The mixture was briefly sonicated until a solution was formed, which was then added to the weighed in catalyst. To this mixture NsN_3_ (1 M in EtOAc, 0.55 mL, 0.55 mmol) was added, and the mixture was briefly shaken. The reaction mixture was let to stand at room temperature for 24 h. K_2_CO_3_ (138 mg, 1.0 mmol) was added to the reaction mixture, followed by slow addition of PhSH (102 μL, 1.0 mmol) upon which gas evolution was observed (decomposition of unreacted NsN_3_). The reaction mixture was then concentrated under reduced pressure. The residue was redissolved in MeCN (2.5 mL) and K_2_CO_3_ (138 mg, 1.0 mmol) and PhSH (102 μL, 1.0 mmol) were added. The reaction vial was closed and heated at 60 °C for 16 h. The mixture was then transferred to a phase separator containing 1 M aq. HCl (50 mL) and EtOAc (50 mL). The organic phase was collected and the aqueous phase was extracted with EtOAc (3×40 mL). The combined organic fractions were washed with brine (40 mL), dried over MgSO_4_ and concentrated under reduced pressure. The residue was redissolved in DMSO (1 mL) and purified by acidic HPLC (gradient specifications described in each experiment).

**Synthesis of common intermediate 2 yc for conjugates**: [Cp*Ir(H_2_O)_3_]SO_4_ (7.3 mg, 0.015 mmol) was weighed in a 16 mL glass scintillation vial. In a separate vial Tranilast (163.7 mg, 0.50 mmol) was added, followed by addition of HFIP (2.5 mL) and Et_3_N (69.7 μL, 0.50 mmol). The mixture was briefly sonicated until a solution as formed, which was then added to the weighed in catalyst. To this mixture benzyl (2‐(azidosulfonyl)ethyl)carbamate (**4 a**, 1 M in EtOAc, 0.55 mL, 0.55 mmol) was added, and the mixture was briefly shaken. The reaction mixture was let to stand at room temperature for 16 h. After this AcOH (143 μL, 2.5 mmol) was added and the total volume reduced to circa 50 % under reduced pressure, upon which the reaction product precipitated. The mixture was partitioned between EtOAc (40 mL) and aqueous HCl (1 M, 60 mL), in which the product remained insoluble. The product was then collected by vacuum filtration and used in the next step without further purification. Dried in vacuo overnight. The residue was suspended in DMSO (3 mL) and *t*BuOK (448 mg, 4.0 mmol) was added. The reaction mixture was stirred at room temperature for 8 h, after which LCMS analysis showed complete Cbz deprotection. Formic acid (300 μL) was added to the reaction mixture and stirred for 5 min. The mixture was then directly injected on HPLC and the product purified (acidic method, gradient 22–62 %). The product **2 yc** (173 mg, 77 %) was obtained as a white solid.

## Conflict of interest

The authors declare no conflict of interest.

## Supporting information

As a service to our authors and readers, this journal provides supporting information supplied by the authors. Such materials are peer reviewed and may be re‐organized for online delivery, but are not copy‐edited or typeset. Technical support issues arising from supporting information (other than missing files) should be addressed to the authors.

Supporting Information
