# Supplementary material for: Late‐Stage Amination of Drug‐Like Benzoic Acids: Access to Anilines and Drug Conjugates through Directed Iridium‐Catalyzed C−H Activation
Source: Chemistry. 2021 Nov 17;27(72):18188–200. doi: 10.1002/chem.202103510 (PMC9299223; doi:10.1002/chem.202103510)
Supplement: Supplementary file 1 — Supporting Information [file CHEM-27-18188-s001.pdf]

Chemistry–A European Journal

Supporting Information

Late-Stage Amination of Drug-Like Benzoic Acids: Access to Anilines and Drug Conjugates through Directed Iridium-Catalyzed C–H Activation

Erik Weis, Magnus J. Johansson,* and Belén Martín-Matute*

Contents

General information S2

General reagent and purification information S2

General analytical information S2

General safety considerations S2

General screening and HTE information S2

Catalyst preparation S2

Reaction optimization S3

Initial hit S3

Solvent screening S3

Catalyst loading screening S5

Functional Group Tolerance study S6

Mechanistic studies S8

Deuterium incorporation studies S8

Kinetic isotope effect (KIE) investigations S9

System 1: 2-Fluorobenzoic acid, TsN_3 .... S9

System 2: *o*-Toluic acid, TsN_3 .... S10

System 3: 2-Fluorobenzoic acid, NsN_3 .... S11

Kinetic profile: 2-Fluorobenzoic acid, TsN_3 .... S12

Synthesis of starting materials S13

General experimental procedures S15

Experimental and Analytical Data for Products S16

NMR spectra and HPLC chromatograms S33

References S101

General information

General reagent and purification information

1,1,1,3,3,3-hexafluoroisopropanol (HFIP) was purchased from Chem-Impex and used directly without drying or degassing. Unless otherwise noted, all reagents were used as received. The $[Cp^*IrCl_2]_2$ catalyst precursor for the preparation of $[Cp^*Ir(H_2O)_3]SO_4$ was purchased from Strem Chemicals. Et_3N was purchased from Sigma Aldrich. TsN_3 was purchased from Sigma Aldrich as a solution in toluene and concentrated under reduced pressure to the pure reagent. Organic phases (after aqueous workup) were dried over anhydrous $MgSO_4$, filtered, and evaporated to dryness under reduced pressure. Flash column chromatography purifications were performed on Biotage ISOLUTE SI 25 g pre-loaded columns, unless otherwise stated. For HPLC purification preparative reverse-phase HPLC on a Kromasil C8 column (10 µm, 250x50 ID mm), using gradient elution (A: H2O/MeCN/FA = 95/5/0.2, B: MeCN) with a flow rate of 100 mL/min over 20 minutes. Gilson GX-281 liquid handler/autosampler was used. UV detector Gilson UV/VIS-155 was used for UV-triggered collection of fractions at 254 nm wavelength. For SFC-MS analysis a Waters Acquity UPC2 SFC-MS system with a BEH column was used.

General analytical information

LCMS analysis was carried out on a Waters Acquity UPLC system, on a HSS C18 column. Nuclear magnetic resonance spectra (^1H, ^{13}C, ^{19}F, COSY, HSQC, HMBC) were recorded on Bruker ULTRASHIELD 500 and 600 MHz spectrometer with a Bruker CRYO PLATFORM. ^1H NMR spectra were referenced to CD_3OD (3.31 ppm), DMSO-d_6 (2.50) and acetone-d_6 (2.05 ppm). ^{13}C NMR spectra were recorded at 126 and 151 MHz, referenced to in CD_3OD (49.00 ppm), DMSO-d_6 (39.52) and acetone-d_6 (206.26 ppm). Coupling constant (J) values were measured in Hertz (Hz) and chemical shift (δ) values in parts per million (ppm). High resolution mass spectra (HRMS) were recorded on a Bruker microTOF ESI-TOF mass spectrometer.

General safety considerations

Organic azides are known high energy compounds and special precautions need to be taken when used. The reagents were at all time used at the temperature of 23 °C or room temperature, approximately a 100 °C from decomposition initiation temperatures as a safety precaution. The decomposition initiation temperatures of 120-128 °C for TsN_3 and 118 °C have been reported.[1] Temperature restrictions were also included for in-vacuo concentrations and reactions themselves. During work-ups plastic separation funnels were used. As N_2 gas is formed as a side-product in the reaction, measures to mitigate the pressure build-up should be taken. Keeping a 1:3 reaction volume to head space is recommended.

General screening and HTE information

Solids were weighed in manually. For liquid dispensing syringes, manual pipettes and the TECAN freedom evo liquid handling platform was used. For solvent removal the SP Scientific Genevac HT-6 evaporation system was used. The reactions were set-up in a Para-Dox® 24- or 96-position parallel synthesis plate using 1 mL vials.

Catalyst preparation

The $[Cp^*Ir(H_2O)_3]SO_4$ catalyst was prepared with a modified literature procedure.[2]

$AgSO_4$ (0.754 mmol, 235 mg) was added to a suspension of $[Cp^*IrCl_2]_2$ (0.377 mmol, 300 mg) in H_2O (2.5 mL). The mixture was stirred at room temperature for 16 h. The contents were then transferred into a Corning® 15 mL centrifuge tube and centrifuged at 2000rpm for 1 minute to sediment the

silver salts. The supernatant was removed and kept. The cake was washed with water (8 mL), centrifuged and filtrate collected two times. The combined aqueous phases were concentrated in vacuo to yield the product [Cp*Ir(H_2O)$_3$]SO_4 as a yellow solid (341mg, 93%). Catalyst appearance depicted in Figure S1. The catalyst is stored under air atmosphere at room temperature in our group, with no observed diminishing of catalytic activity (batches up to 2 years of age used).

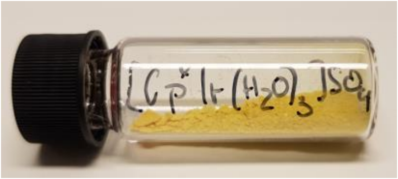

*Figure S1. Sample of [Cp*Ir(H_2O)$_3$]SO_4.*

Reaction optimization

Initial hit

The initial hit was obtained using potassium benzoate as starting material (Scheme S1).

Potassium benzoate (**1a**$_{\mathbf{K\text{-}salt}}$, 16.0 mg, 0.1 mmol), [Cp*Ir(H_2O)$_3$]SO_4 (0.5 mg, 1 µmol) and KOAc (14.7 mg, 0.15 mmol) were charged in a 1.5 mL scintillation vial. HFIP (1 mL) was added, followed by addition of TsN_3 (18.4 µL, 0.12 mmol). The vial was sealed and the mixture stirred at room temperature for 22 h. The reaction outcome was analyzed by SFC-MS. Formation of a mixture of **2t´** and **2t** was observed, however, this could not be quantified due to bad separation.

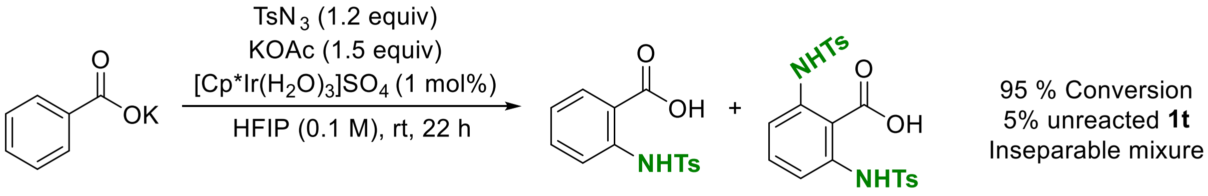

1t$_{\mathbf{K\text{-}salt}}$ **2t´** **2t**

*Scheme S1. Initial C-H sulfonamidation hit. The exact **2t´** to **2t** ratio was not determined. 95% conversion observed by SFC-MS (based on UV trace)*

Solvent screening

In the next step (solvent screening), we decided to increase the catalyst loading in order to increase the chances of obtaining a hit. We changed the substrate to *m*-toluic acid in order to suppress mono/di-functionalized product mixture formation. Furthermore, we chose the commercially available acid as starting material instead of the potassium salt. The KOAc additive used in the previous stage was removed, and instead Et_3N was tested as a base additive (Table S1).

The reactions were set up using a 96-well Para-Dox® plate with 1 mL vials. Reaction scale 0.025 mmol.

Experimental set-up:

Stock solution SS1: [Cp*Ir(H_2O)$_3$]SO_4 (14.6 mg, 0.03 mmol) in deionized water (1.2 mL total volume).

Stock solution SS2: *m*-toluic acid (204 mg, 1.5 mmol) in MeOH (4.5 mL total volume).

Stock solution SS1 (20 µL per vial) was added to the reaction plate via the TECAN liquid handling platform (Figure S2). The plate was transferred into the Genevac HT-6 evaporation system (Figure S2), and evaporated to dryness. Stock solution SS2 (75 µL per vial) was added to the plate via the

TECAN liquid handling platform and evaporated to dryness with the Genevac HT-6 evaporation system. Wedge-shaped magnetic stirrer bars were added to each reaction vial. The reaction solvents (160 µL per well) were added to each individual vial by manual pipetting. To vials in columns 4-6 Et_3N (3.5 µL, 0.025 mmol, per vial) was added manually with a multichannel pipette. The vials were sealed and the plate was stirred at room temperature for 18 h. After this DMSO (150 µL per vial) was added to the plate via the TECAN liquid handling system and the reactions were stirred for additional 5 minutes.

An analytical plate was prepared by manual pipetting with multichannel pipettes. MeOH (150 µL per well) was added into an analytical 96-well plate. To this samples from each vials were added (3 µL per vial, collection from the bottom of the vial). The plate was sealed and analyzed by analytical SFC-MS.

Table S1: Solvent screening

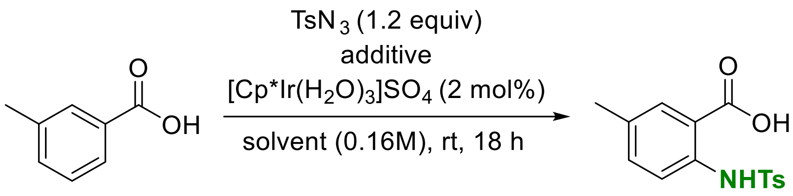

	No Additive			Et₃N (1 equiv)		
Entry	1	2	3	4	5	6
A	0	0	6	2	0	5
B	3	0	3	2	0	5
C	2	1	0	3	2	1
D	0	0	1	3	0	1
E	7	0	1	3	0	0
F	39	4	0	77	15	0
G	2	0	2	2	11	1
H	1	1	0	3	1	0

Entry	1, 4	2, 5	3, 6
A	Water	AcOH	CH_2Cl_2
B	MeOH	MeCN	DCE
C	EtOH	DMF	$CHCl_3$
D	*i*PrOH	DMSO	THF
E	TFE	NMP	2-MeTHF
F	HFIP	acetone	dioxane
G	$PhCH_3$	heptane	TBME
H	$PhCF_3$	EtOAc	DME

Conversions to product in % shown in table. Color gradient from red to green results from worst to best. Best result in F4.

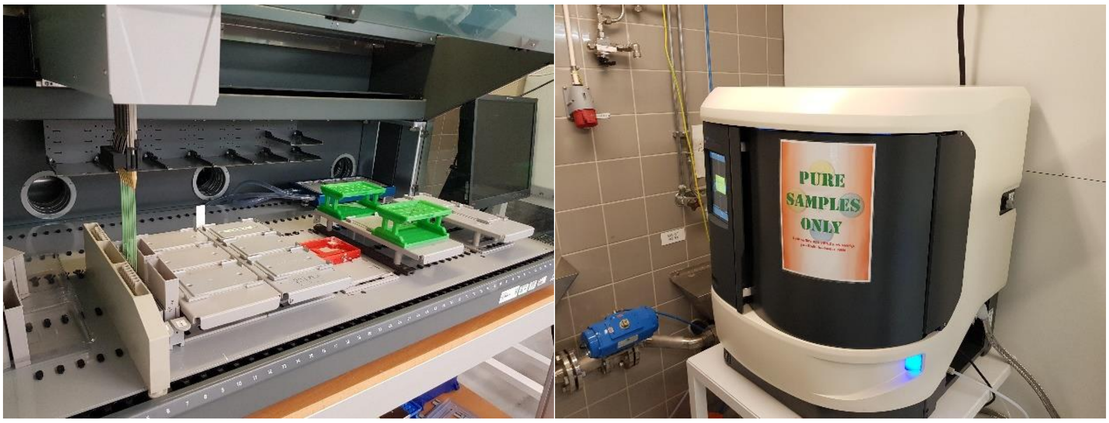

Figure S2. Left: TECAN liquid handling platform used throughout the screening campaign. Right: Genevac HT-6 evaporation system used for solvent removal.

Catalyst loading screening

The reactions were set up using a 24-well Para-Dox® plate with 1 mL vials. Reaction scale 0.025 mmol (Table S2).

Experimental set-up:

$[Cp^*Ir(H_2O)_3]SO_4$ was weighed in individually to each vial. Stock solutions of substrates and Et_3N were prepared and 155 µL (0.025 mmol of reagents) pipetted to each vial. TsN_3 (4.6 µL, 0.03 mmol) was added to each vial. The vials were sealed and shaked on a plate shaker for 10 minutes. After this the reaction plate was let to stand at room temperature for 20 h. Samples for SFC-MS analysis were prepared (150 µL MeOH + 2 µL reaction mixture). The results are plotted in Table S2.

Table S2: Catalyst loading screening

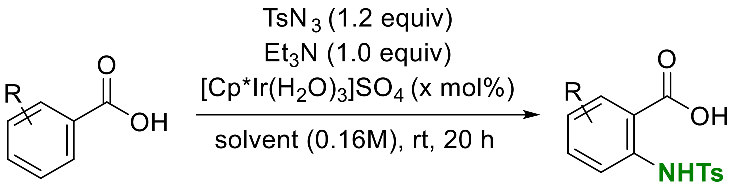

Substrate	$[Cp^*Ir(H_2O)_3]SO_4$ (mol%)	Conv. (%)	Substrate	$[Cp^*Ir(H_2O)_3]SO_4$ (mol%)	Conv. (%)
2Me	0.5	51	1napth	0.5	41
2Me	1	64	1napth	1	49
2Me	2	80	1napth	2	62
2Me	3	100	1napth	3	78
3CF3	0.5	68	2F	0.5	100
3CF3	1	92	2F	1	100
3CF3	2	100	2F	2	100
3CF3	3	100	2F	3	100

Conversions to product in % shown in table. Color gradient from red to green results from worst to best. 3 mol% chosen for further reactions.

Functional Group Tolerance study

The reactions were set up using a 96-well Para-Dox® plate with 1 mL vials. Reaction scale 0.025 mmol. The reactions were carried out without exclusion of air. The reagents were used as received from vendors. Reactions run in duplicates (55 reactions per plate, Table S3).

Stock solutions:

SS1: 3-(trifluoromethyl) toluic acid (570 mg, 3.0 mmol), was weighed in a 20 mL scintillation vial, followed by addition of HFIP (8.2 mL) and Et_3N (418 µL, 3.0 mmol). The contents were briefly sonicated and a clear solution was obtained. To this TsN_3(483 µL, 3.15 mmol) was added and the mixture briefly shaked. The resulting solution was partitioned in equal portions into 4 mL high recovery vials.

SS2: $[Cp^*Ir(H_2O)_3]SO_4$ (43.9 mg, 0.09 mmol) was charged in a 4 mL high recovery vial, followed by addition of HFIP (3mL). The mixture was briefly sonicated, upon which a yellow solution was obtained.

Experimental procedure:

1) Solid additives (1.0 equiv, 0.025 mmol) were manually weighed in in the corresponding vials.

Step time: 48m 34s

2) HFIP (50 µL) was added to all vials in the plate using the TECAN liquid dispensing system (Figure S3).

Step time: 3m 46s

3) Liquid additives (1.0 equiv, 0.025 mmol) were added to the corresponding vials by manual pipetting (Figure S3). Addition into the HFIP volume, mixed with pipette.

Step time: 29m 8s

4) The stock solution SS1 (75 µL per vial) was pipetted using the TECAN liquid dispensing system, followed by addition of stock solution SS2 (25 µL per vial) using the same system.

Step time: 5m 7s

5) The plate was sealed and shaked on a plate shaker at room temperature for 20 h (Figure S3).

Step time: 20 h

6) SiliaMetS® imidazole metal scavenger was added in DMSO (150 µL per vial) with a multichannel pipette and the reactions were stirred at room temperature for 2 h.

Step time: 2 h

7) The plate was then centrifuged for 10 minutes in order to deposit the solid-supported scavenger and other solids.

Step time: 12 minutes

8) An analytical plate was prepared by manual pipetting with multichannel pipettes (Figure S3). DMSO (150 µL per well) was added into an analytical 96-well plate. To this samples from each vials were added (3 µL per vial, collection from the top/middle of the vial). The plate was sealed and analyzed by analytical LCMS. Analysis time 5 h 30 minutes.

Step time: 6 h

Overall step time: 29 h 39 minutes. The <48 h time depiction shown in Table 2 accounts also for synthesis planning and collection of compounds from storage and stock solution preparation.

Table S3: Functional group compatibility screening

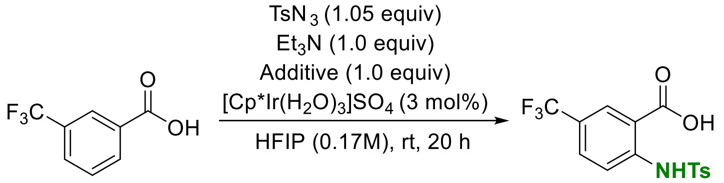

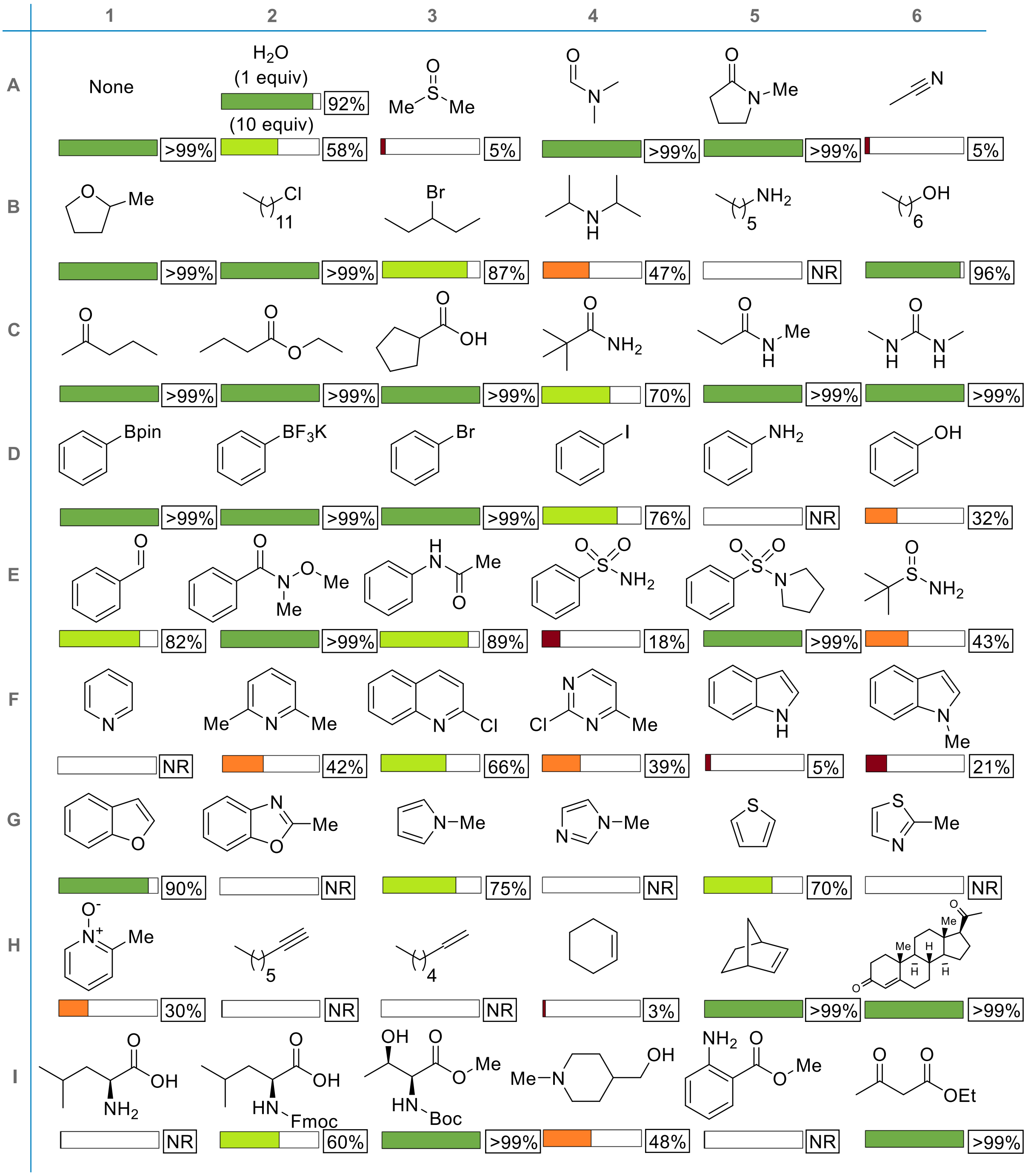

Conversion percentage values shown in sqares. Tolerance to additive represented by color scheme: >50% conversion green, 25-50% orange, <25% red.

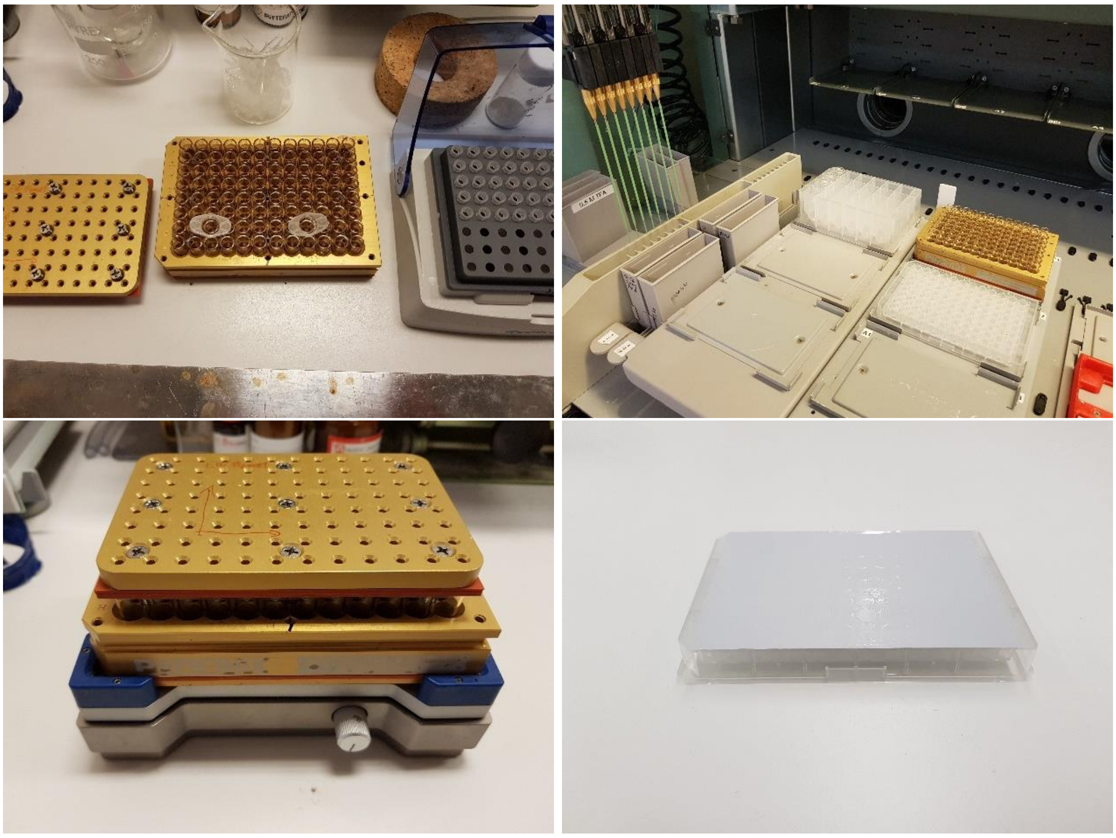

Figure S3. Top left: Manual addition of reagents into the reaction block. Plastic tabs (cut off 3 mL syringes) were used to aid the dispension progress and mitigate human error. Top right: Dispensing of stock solutions using the TECAN liquid handling platform. Bottom left: Reaction set-up after completion of the plate. Orbital shaker used for agitating the reaction mixtures. Bottom right: Analytical 96-well plate used for reaction evaluation via plate LCMS.

Mechanistic studies

Deuterium incorporation studies

The deuterium incorporation in two distinct compounds (1a and 1c) was first investigated in absence of azide in HFIP-*d* under otherwise standard reaction conditions (Scheme S2):

$[Cp^*Ir(H_2O)_3]SO_4$ (1.5 mg, 3.0 µmol) was weighed in a 1.5 mL glass scintillation vial. In a separate vial substrate (0.10 mmol) was added, followed by addition of HFIP-*d* (0.5 mL) and Et_3N (13.9 µL, 0.10 mmol). The mixture was briefly sonicated until a solution as formed, which was then added to the weighed in catalyst. The reactions were let to stir at room temperature for 20 h.

The mixture was then transferred to a phase separator containing 1M aq. HCl (10 mL) and EtOAc (10 mL). The organic phase was collected and the aqueous phase was extracted with EtOAc (3 x 10 mL). The combined organic fractions were washed with brine (40 mL), dried over $MgSO_4$ and concentrated under reduced pressure. The purification of the products is described in each experiment entry separately. The products were purified by flash column chromatography (10-100 % EtOAc with 2% AcOH in heptane, 10 g SiO_2). Deuterium incorporation was assessed by ^1H NMR.

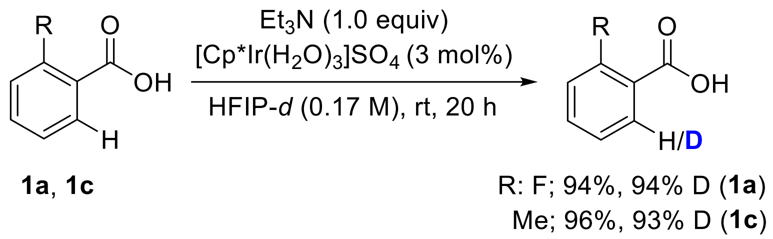

Scheme S2. Deuterium incorporation study under modified conditions (no azide). C-H activation was shown to be reversible.

The deuterium incorporation in two distinct compounds (1a and 1c) was then investigated in presence of TsN_3 in HFIP-*d* under otherwise standard reaction conditions (Scheme S3):

[Cp*Ir(H_2O)$_3$]SO_4 (1.5 mg, 3.0 µmol) was weighed in a 1.5 mL glass scintillation vial. In a separate vial substrate (0.10 mmol) was added, followed by addition of HFIP-*d* (0.5 mL) and Et_3N (13.9 µL, 0.10 mmol). The mixture was briefly sonicated until a solution as formed, which was then added to the weighed in catalyst. TsN_3 (16.1, 0.11 mmol) was added. The reaction with **1a** was stirred for 8 min, while the reaction of **1c** for 15 min.

The mixture was then transferred to a phase separator containing 1M aq. HCl (10 mL) and EtOAc (10 mL). The organic phase was collected and the aqueous phase was extracted with EtOAc (3 x 10 mL). The combined organic fractions were washed with brine (40 mL), dried over $MgSO_4$ and concentrated under reduced pressure. Conversion was established by qNMR (^{1}H), directly from the obtained material (CD_3OD, 1,1,2,2-tetrachloroethane as standard). No deuterium incorporation was observed.

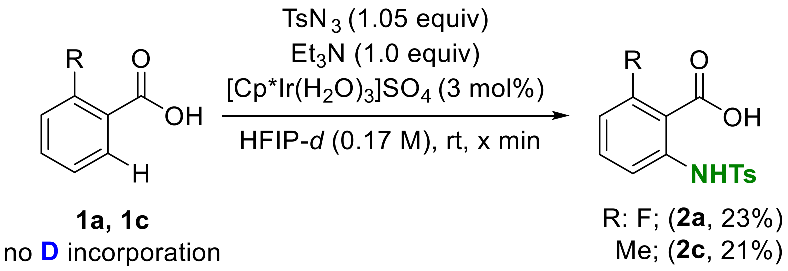

Scheme S3. Deuterium incorporation under disrupted reaction conditions. No D incorporation in starting material was observed. C–H activation not reversible under these conditions.

Kinetic isotope effect (KIE) investigations

A KIE investigation was conducted with three distinct systems. In all cases the parallel experiment set-up was chosen in order to reliably determine whether the C–H activation step is turnover-determining in the reaction.[3]

System 1: 2-Fluorobenzoic acid, TsN_3

[Cp*Ir(H_2O)$_3$]SO_4 (1.5 mg, 3.0 µmol) was weighed in a 1.5 mL glass scintillation vial. In a separate vial substrate **1a** (14.0 mg, 0.10 mmol) was added, followed by addition of HFIP (0.5 mL) and Et_3N (13.9 µL, 0.10 mmol). The mixture was briefly sonicated until a solution as formed, which was then added to the weighed in catalyst. TsN_3 (16.1, 0.11 mmol) was added. The reaction was stirred at room temperature. Aliquots of 2 µL were taken every 20 seconds and added to MeOH (0.1 mL). These samples were used for analysis by SFC-MS. Reactions carried out in triplicates.

The same reactions were set up with deuterated **1a$_D$**, while HFIP-*d* was used as solvent. Aliquots were taken every minute.

The k_H/k_D value of 6.2 obtained in this study points to the C–H activation step being turnover determining under the described conditions (Scheme S4).

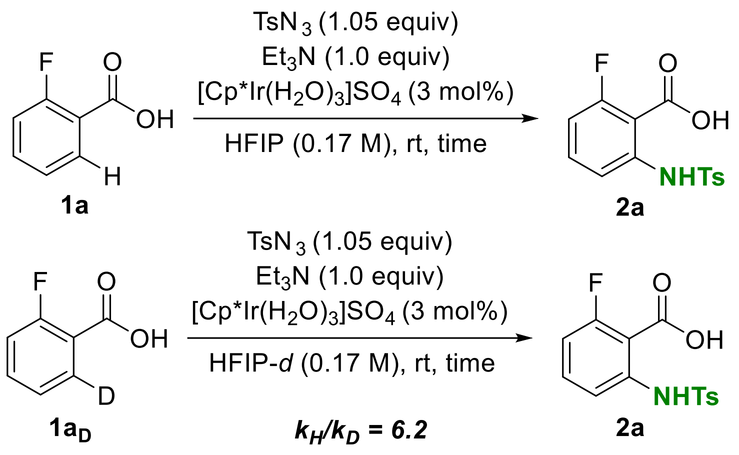

Scheme S4. KIE study using 2-fluorobenzoic acid and TsN_3. The $k_H/k_D > 1$ suggests C–H activation is the turnover determining step under these conditions.

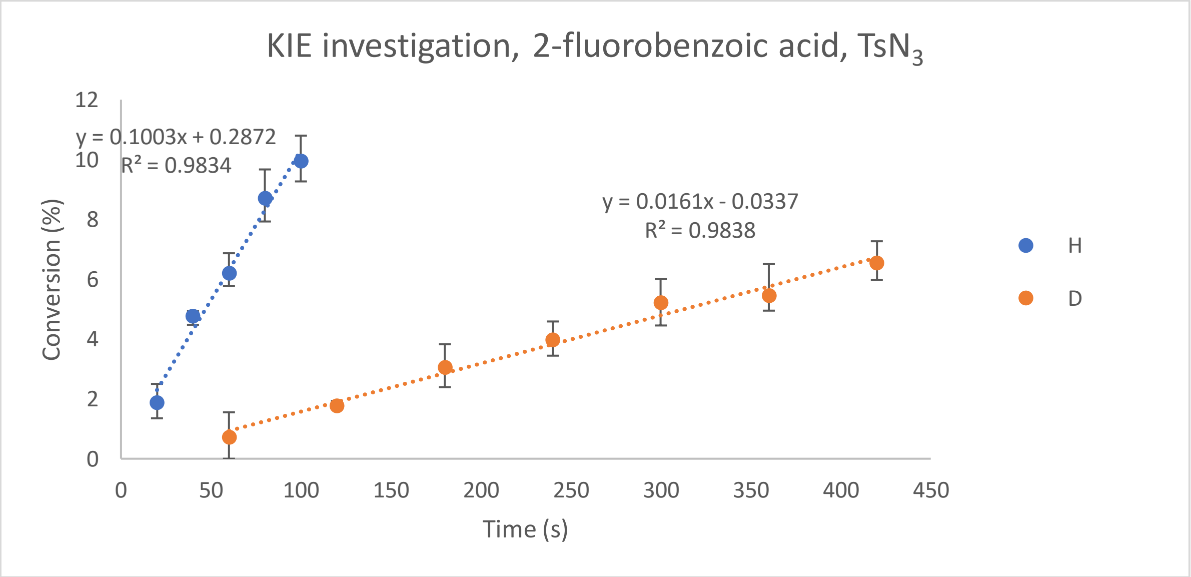

System 2: *o*-Toluic acid, TsN_3

$[Cp^*Ir(H_2O)_3]SO_4$ (1.5 mg, 3.0 µmol) was weighed in a 1.5 mL glass scintillation vial. In a separate vial substrate **1c** (13.6 mg, 0.10 mmol) was added, followed by addition of HFIP (0.5 mL) and Et_3N (13.9 µL, 0.10 mmol). The mixture was briefly sonicated until a solution as formed, which was then added to the weighed in catalyst. TsN_3 (16.1, 0.11 mmol) was added. The reaction was stirred at room temperature. Aliquots of 2 µL were taken every minute and added to MeOH (0.1 mL). These samples were used for analysis by SFC-MS. Reactions carried out in triplicates.

The same reactions were set up with deuterated **1c**$_D$, while HFIP-*d* was used as solvent. Aliquots were taken every minute.

The k_H/k_D value of 2.9 obtained in this study points to the C–H activation step being turnover determining under the described conditions (Scheme S5).

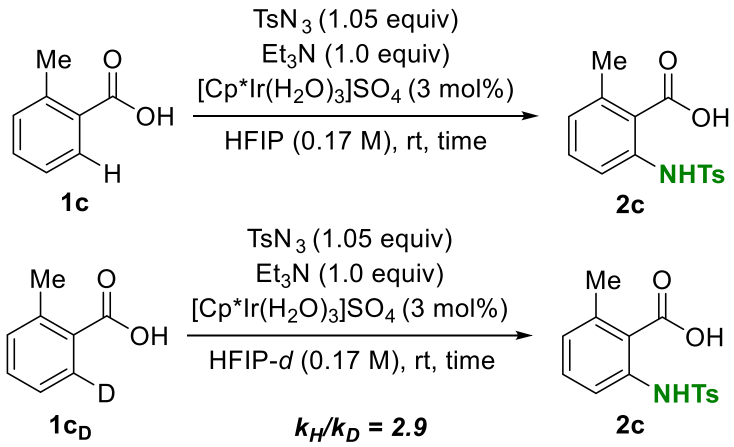

Scheme S5. KIE study using o-*toluic acid and* TsN_3*. The* $k_H/k_D > 1$ *suggests C–H activation is the turnover determining step under these conditions.*

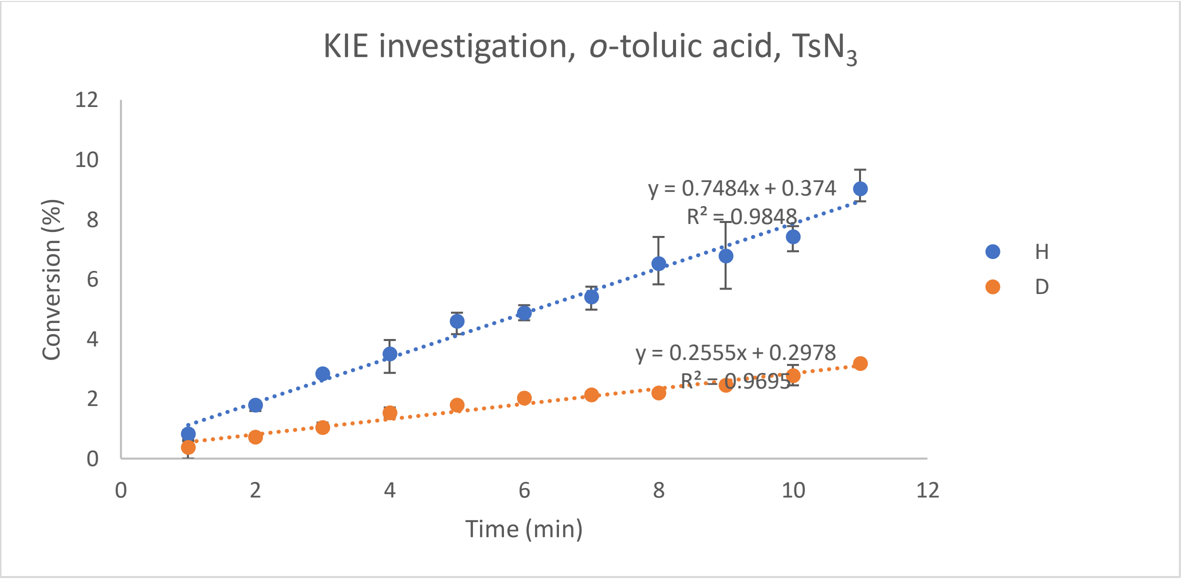

System 3: 2-Fluorobenzoic acid, NsN_3

$[Cp^*Ir(H_2O)_3]SO_4$ (1.9 mg, 4.0 µmol) was weighed in a 1.5 mL glass scintillation vial. In a separate vial substrate **1a** (14.0 mg, 0.10 mmol) was added, followed by addition of HFIP (0.5 mL) and Et_3N (13.9 µL, 0.10 mmol). The mixture was briefly sonicated until a solution as formed, which was then added to the weighed in catalyst. TsN_3 (16.1, 0.11 mmol) was added. The reaction was stirred at room temperature. Aliquots of 2 µL were taken every minute and added to MeOH (0.1 mL). These samples were used for analysis by SFC-MS. Reactions carried out in triplicates.

The same reactions were set up with deuterated **1a**$_D$, while HFIP-*d* was used as solvent. Aliquots were taken every five minutes.

The k_H/k_D value of 5.5 obtained in this study points to the C–H activation step being turnover determining under the described conditions (Scheme S6).

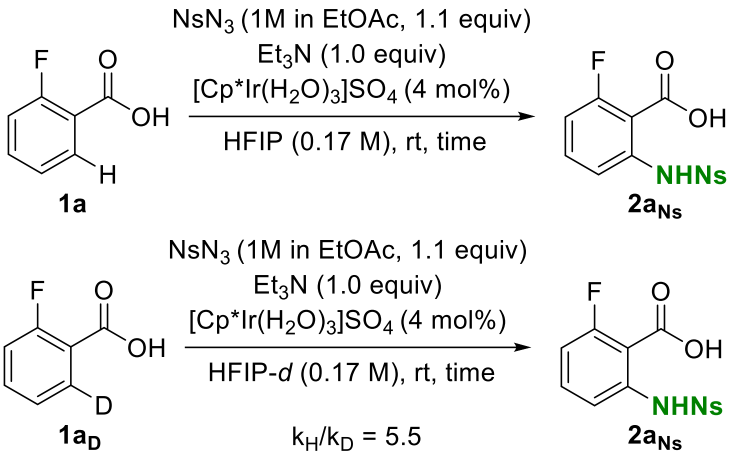

Scheme S6. KIE study using 2-fluorobenzoic acid and NsN_3. The $k_H/k_D > 1$ suggests C–H activation is the turnover determining step under these conditions.

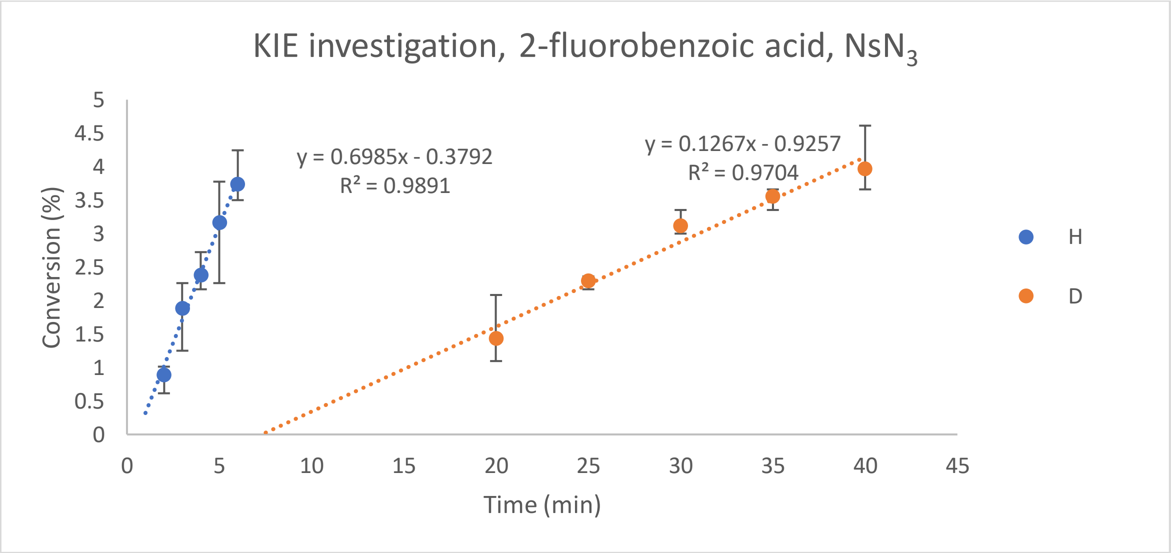

Kinetic profile: 2-Fluorobenzoic acid, TsN_3

$[Cp^*Ir(H_2O)_3]SO_4$ (1.5 mg, 3.0 µmol) was weighed in a 1.5 mL glass scintillation vial. In a separate vial substrate **1a** (14.0 mg, 0.10 mmol) was added, followed by addition of HFIP (0.5 mL) and Et_3N (13.9 µL, 0.10 mmol). The mixture was briefly sonicated until a solution as formed, which was then added to the weighed in catalyst. TsN_3 (16.1, 0.11 mmol) was added. The reaction was stirred at room temperature. Aliquots of 2 µL were taken every 20 seconds and added to MeOH (0.1 mL). These samples were used for analysis by SFC-MS.

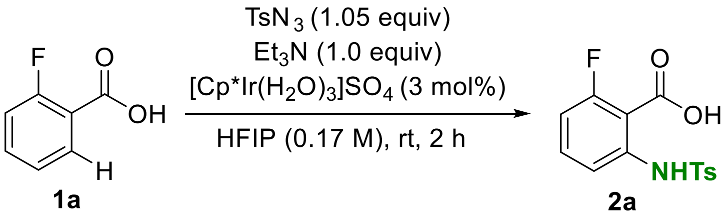

Scheme S7. Kinetic profile of sulfonamidation of 2-fluorobenzoic acid with TsN_3.

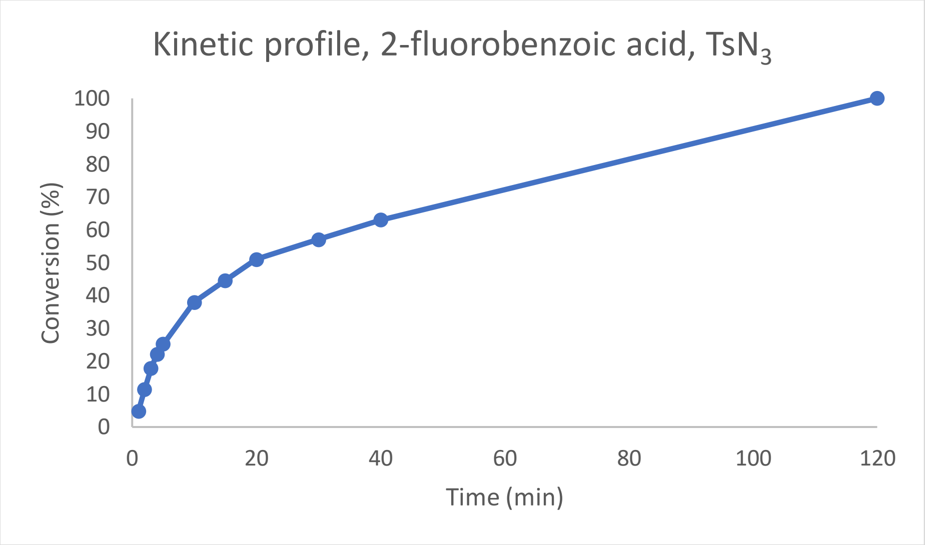

Time (min)	Conversion (%)
1	5
2	11
3	18
4	22
5	25
10	38
15	45
20	51
30	57
40	63
120	100

The reaction proceeds to completion under 2 hours. No induction period observed. The initial data points consistent with the initial rate studies from the KIE investigations.

Synthesis of starting materials

Synthesis of **2-nitrobenzenesulfonyl azide** (**NsN_3**)

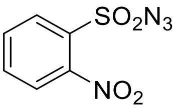

Sodium azide (645 mg, 9.9 mmol) was suspended in water (4 mL) and acetone (4 mL) in a microwave vial (20 mL). 2-nitrobenzenesulfonyl chloride (2 g, 9.0 mmol) was added as a solution in acetone (5 mL) over 1 minute at 0 °C while stirred. The reaction mixture was let to heat up to room temperature and stirred at 1000 RPM for 16 h. After this the contents were transferred to a separator funnel (plastic), partitioned between EtOAc (50 mL) and 1M NaOH (50 mL). The organic phase was collected and the aqueous extracted with EtOAc (2 x 50 mL). The combined organic phases were dried with $MgSO_4$, filtered and concentrated to circa 5 mL total volume. The total volume was adjusted to 9 mL to obtain the 1M stock solution, which was used directly without further purification. Light orange solution.

^{1}H NMR (500 MHz, DMSO-d_6) δ 8.27 (dd, J = 8.0, 1.3 Hz, 1H), 8.22 (dd, J = 8.0, 1.2 Hz, 1H), 8.11 (td, J = 7.8, 1.4 Hz, 1H), 8.02 (td, J = 7.8, 1.3 Hz, 1H). **^{13}C NMR** (126 MHz, DMSO-d_6) δ 147.2, 137.1, 133.9, 131.3, 130.7, 125.8.

Compound previously prepared and reported.[1]

Synthesis of **benzyl (2-(azidosulfonyl)ethyl)carbamate** (**4a**)

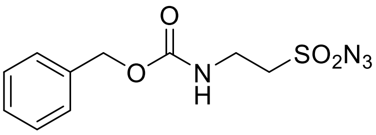

Sodium azide (193 mg, 3.0 mmol) was suspended in water (2 mL) and acetone (2 mL) in a microwave vial (20 mL). Benzyl (2-(chlorosulfonyl)ethyl)carbamate (750 mg, 2.7 mmol) was added as a solution in acetone (2 mL) over 1 minute at 0 °C while stirred. The reaction mixture was let to heat up to room

temperature and stirred at 1000 RPM for 16 h. After this the contents were transfered to a separator funnel (plastic), partitioned between EtOAc (25 mL) and 1M NaOH (25 mL). The organic phase was collected and the aqueous extracted with EtOAc (2 x 25 mL). The combined organic phases were dried with $MgSO_4$, filtered and concentrated to circa 2 mL total volume. The total volume was adjusted to 2.7 mL to obtain the 1M stock solution, which was used directly without further purification. Colorless, slightly opaque solution.

^{1}H NMR (500 MHz, DMSO-d_6) δ 7.60 (t, *J* = 5.1 Hz, 1H), 7.3 – 7.39 (m, 5H), 5.05 (s, 2H), 3.87 (t, *J* = 6.4 Hz, 2H), 3.50 (q, *J* = 6.2 Hz, 2H). **^{13}C NMR** (126 MHz, DMSO-d_6) δ 156.06, 136.92, 128.37, 127.86, 127.78, 65.59, 54.26, 35.25. **HRMS** $C_{10}H_{12}N_4O_4S$; calcd. for (M+H$^+$): 285.0662, found: 285.0660.

Compound previously prepared and reported.[4]

Synthesis of **2-fluorobenzoic-6-d acid (1a$_D$)**

The synthesis was based on published procedure.(*62*)

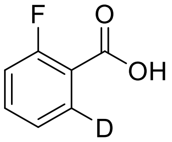

2-fluorobenzoic acid (140 mg, 1.0 mmol) was added to a microwave vial, followed by addition of [Cp*Rh(MeCN)$_3$](SbF$_6$)$_2$ (8.4 mg, 10 µmol) and NaOAc (90.3 mg, 1.1 mmol). D_2O (4 mL) was added, the vial capped and stirred at 90 °C for 24 h. The mixture was then transferred to a phase separator containing 1M aq. HCl (50 mL) and EtOAc (50 mL). The organic phase was collected and the aqueous phase was extracted with EtOAc (3 x 40 mL). The combined organic fractions were washed with brine (40 mL), dried over $MgSO_4$ and concentrated under reduced pressure. The product was purified by flash column chromatography (10-100 % EtOAc with 2% AcOH in heptane, 25 g SiO_2). Compound **1a$_D$** was obtained as a white solid (115.6 mg, 82%).

^{1}H NMR (500 MHz, CD_3OD) δ 7.59 (ddd, *J* = 8.3, 7.4, 4.9 Hz, 1H), 7.25 (d, *J* = 7.4 Hz, 1H), 7.19 (ddd, *J* = 11.1, 8.3, 1.1 Hz, 1H). **^{13}C NMR** (126 MHz, CD_3OD) δ 167.4 (d, J_{C-F} = 3.2 Hz, 1C), 163.4 (d, J_{C-F} = 258.3 Hz, 1C), 135.8 (d, J_{C-F} = 9.1 Hz, 1C), 133.0 (t, J_{C-D} = 25.2 Hz, 1C), 125.1 (d, J_{C-F} = 3.9 Hz, 1C), 120.3 (d, J_{C-F} = 9.8 Hz, 1C), 117.9 (d, J_{C-F} = 22.7 Hz, 1C). **^{19}F NMR** (471 MHz, CD_3OD) δ – 111.97. **HRMS** $C_7H_4DFO_2$; calcd. for (M-H$^+$): 140.0263, found: 140.0264.

Synthesis of **2-methylbenzoic-6-d acid (1c$_D$)**

Synthesis according to published procedure,[5] with modified purification procedure.

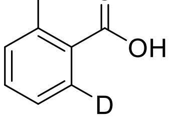

2-methylbenzoic acid (136 mg, 1.0 mmol) was added to a microwave vial, followed by addition of [Cp*Rh(MeCN)$_3$](SbF$_6$)$_2$ (8.4 mg, 10 µmol) and NaOAc (90.3 mg, 1.1 mmol). D_2O (4 mL) was added, the vial capped and stirred at 90 °C for 24 h. The mixture was then transferred to a phase separator containing 1M aq. HCl (50 mL) and EtOAc (50 mL). The organic phase was collected and the aqueous phase was extracted with EtOAc (3 x 40 mL). The combined organic fractions were washed with brine (40 mL), dried over $MgSO_4$ and concentrated under reduced pressure. The product was purified by

flash column chromatography (10-100 % EtOAc with 2% AcOH in heptane, 25 g SiO_2). Compound **1c$_D$** was obtained as a white solid (115.5 mg, 85%).

^{1}H NMR (500 MHz, CD_3OD) δ 7.41 (t, *J* = 7.5 Hz, 1H), 7.24 – 7.29 (m, 2H), 2.58 (s, 3H). **^{13}C NMR** (126 MHz, CD_3OD) δ 171.1, 141.1, 133.0, 132.6, 131.5 (t, $J_{C\text{-}D}$ = 24.6 Hz, 1C), 131.23, 126.6, 21.9. **HRMS** $C_8H_8O_2$; calcd. for (M-H$^+$): 135.0451, found: 135.0451.

Compound previously prepared and reported.[5]

General experimental procedures

General procedure A

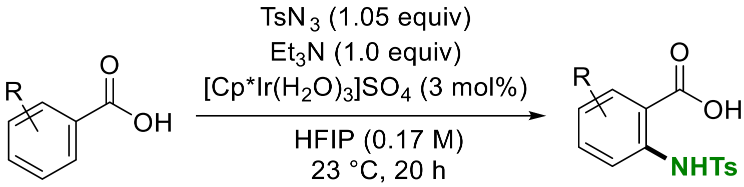

$[Cp^*Ir(H_2O)_3]SO_4$ (7.3 mg, 0.015 mmol) was weighed in a 16 mL glass scintillation vial. In a separate vial substrate (0.50 mmol) was added, followed by addition of HFIP (3 mL) and Et_3N (69.7 µL, 0.50 mmol). The mixture was briefly sonicated until a solution was formed, which was then added to the weighed in catalyst. To this mixture TsN_3 (80.5 µL, 0.53 mmol) was added and the mixture briefly shaked. The reaction mixture was let to stand at room temperature for 20 h.

The mixture was then transferred to a phase separator containing 1M aq. HCl (50 mL) and EtOAc (50 mL). The organic phase was collected and the aqueous phase was extracted with EtOAc (3 x 40 mL). The combined organic fractions were washed with brine (40 mL), dried over $MgSO_4$ and concentrated under reduced pressure. The purification of the products is described in each experiment entry separately.

General procedure B

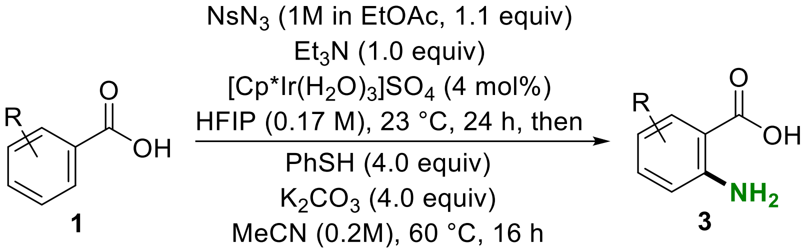

$[Cp^*Ir(H_2O)_3]SO_4$ (9.7 mg, 0.02 mmol) was weighed in a 16 mL glass scintillation vial. In a separate vial substrate (0.50 mmol) was added, followed by addition of HFIP (2.5 mL) and Et_3N (69.7 µL, 0.50 mmol). The mixture was briefly sonicated until a solutionw as formed, which was then added to the weighed in catalyst. To this mixture NsN_3 (1M in EtOAc, 0.55 mL, 0.55 mmol) was added and the mixture briefly shaked. The reaction mixture was let to stand at room temperature for 24 h.

K_2CO_3 (138 mg, 1.0 mmol) was added to the reaction mixture, followed by slow addition of PhSH (102 µL, 1.0 mmol) upon which gas evolution was observed (decomposition of unreacted NsN3). The reaction mixture was then concentrated under reduced pressure. The residue was redisolved in MeCN (2.5 mL) and K_2CO_3 (138 mg, 1.0 mmol) and PhSH (102 µL, 1.0 mmol) were added. The reaction vial was closed and heated at 60 °C for 16 h.

The mixture was then transferred to a phase separator containing 1M aq. HCl (50 mL) and EtOAc (50 mL). The organic phase was collected and the aqueous phase was extracted with EtOAc (3 x 40 mL). The combined organic fractions were washed with brine (40 mL), dried over $MgSO_4$ and concentrated under reduced pressure. The residue was redissolved in DMSO (1 mL) and purified by acidic HPLC (gradient specifications described in each experiment).

Synthesis of **(E)-2-((2-ammonioethyl)sulfonamido)-6-(3-(3,4-dimethoxyphenyl)acrylamido)benzoate (2yc)**

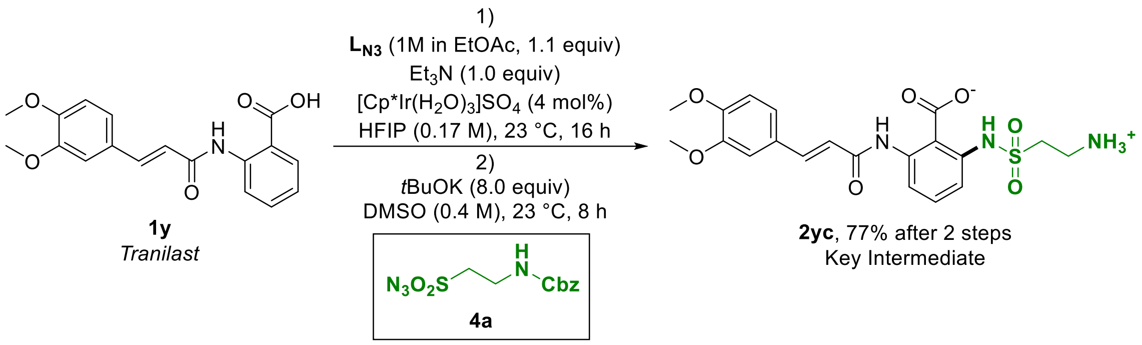

$[Cp^*Ir(H_2O)_3]SO_4$ (7.3 mg, 0.015 mmol) was weighed in a 16 mL glass scintillation vial. In a separate vial Tranilast (163.7 mg, 0.50 mmol) was added, followed by addition of HFIP (2.5 mL) and Et_3N (69.7 µL, 0.50 mmol). The mixture was briefly sonicated until a solution as formed, which was then added to the weighed in catalyst. To this mixture **4a** (1M in EtOAc, 0.55 mL, 0.55 mmol) was added and the mixture briefly shaked. The reaction mixture was let to stand at room temperature for 16 h. After this AcOH (143 µL, 2.5 mmol) was added and the total volume reduced to circa 50% under reduced pressure, upon which the reaction product precipitated. The mixture was partitioned between EtOAc (40 mL) and aqueous HCl (1M, 60 mL), in which the product remained insoluble. The product was then collected by vacuum filtration and used in the next step without further purification. Dried in vacuo overnight.

The residue was suspended in DMSO (3 mL) and *t*BuOK (448 mg, 4.0 mmol) was added. The reaction mixture was stirred at room temperature for 8 h, after which LCMS analysis showed complete Cbz deprotection. Formic acid (300 µL) was added to the reaction mixture and stirred for 5 minutes. The mixture was then directly injected on HPLC and the product purified (acidic method, gradient 22-62%). The product **2yc** (173 mg, 77%) was obtained as a white solid.

^{1}H NMR (500 MHz, DMSO-d_6) δ 8.41 (d, *J* = 8.3 Hz, 1H), 7.69 (s, 3H), 7.50 (d, *J* = 15.6 Hz, 1H), 7.28 – 7.32 (m, 2H), 7.21 (dd, *J* = 8.3, 1.6 Hz, 1H), 7.17 (d, *J* = 8.2 Hz, 1H), 6.99 (d, *J* = 8.3 Hz, 1H), 6.59 (d, *J* = 15.6 Hz, 1H), 3.83 (s, 3H), 3.80 (s, 3H), 3.30-3.33 (m, 2H), 3.06 – 3.1 (m, 2H). **^{13}C NMR** (126 MHz, DMSO-d_6) δ 171.5, 163.9, 150.4, 149.0, 142.7, 142.4, 140.4, 130.8, 127.4, 122.3, 121.0, 113.6, 112.7, 111.6, 110.2, 110.0, 55.61, 55.56, 47.4, 34.0. **HRMS** $C_{20}H_{23}N_3O_7S$; calcd. for $(M-H^+)$: 448.1184, found 448.1184.

Experimental and Analytical Data for Products

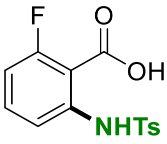

2-Fluoro-6-((4-methylphenyl)sulfonamido)benzoic acid (**2a**) was prepared according to General Procedure A. The product was purified by flash column chromatography (10-100 % EtOAc with 2% AcOH in heptane, 25 g SiO_2). The product **2a** was isolated as a beige solid (143.9 mg, 93%).

^{1}H NMR (500 MHz, Acetone-d_6) δ 10.59 (s, 1H), 7.73 (d, J = 8.3 Hz, 2H), 7.56 (td, J = 8.3, 5.7 Hz, 1H), 7.51 (d, J = 8.3 Hz, 1H), 7.36 (d, J = 8.0 Hz, 2H), 6.94 (ddd, J = 10.9, 8.2, 1.1 Hz, 1H), 2.37 (s, 3H). **^{13}C NMR** (126 MHz, Acetone-d_6) δ 168.4 (d, J_{C-F} = 3.3 Hz, 1C), 163.3 (d, J_{C-F} = 258.3 Hz, 1C), 145.3, 142.3 (d, J_{C-F} = 3.3 Hz, 1C), 137.2, 135.7 (d, J_{C-F} = 11.3 Hz, 1C), 130.7, 128.1, 116.1 (d, J_{C-F} = 3.5 Hz, 1C), 112.4 (d, J_{C-F} = 24.0 Hz, 1C), 108.1 (d, J_{C-F} = 14.5 Hz, 1C), 21.4. **^{19}F NMR** (471 MHz, Acetone-d_6) δ -104.66. **HRMS** $C_{14}H_{12}FNO_4S$; calcd. for $(M-H^+)$: 308.0398, found 308.0396.

Compound previously prepared and reported.[6]

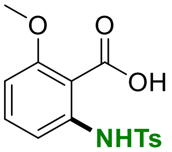

2-Methoxy-6-((4-methylphenyl)sulfonamido)benzoic acid (**2b**) was prepared according to General Procedure A. The product was purified by flash column chromatography (10-100 % EtOAc with 2% AcOH in heptane, 25 g SiO_2). The product **2b** was isolated as a beige solid (123.8 mg, 77%).

^{1}H NMR (500 MHz, Acetone-d_6) δ 11.68 (s, 1H), 11.04 (s, 1H), 7.74 (d, J = 8.3 Hz, 2H), 7.49 (t, J = 8.4 Hz, 1H), 7.33 – 7.38 (m, 3H), 6.92 (d, J = 8.4 Hz, 1H), 4.01 (s, 3H), 2.37 (s, 3H). **^{13}C NMR** (126 MHz, Acetone-d_6) δ 169.3, 160.4, 145.1, 142.4, 137.5, 135.3, 130.7, 128.1, 113.0, 107.8, 107.5, 57.5, 21.4. **HRMS** $C_{15}H_{15}NO_5S$; calcd. for $(M-H^+)$: 320.0598, found 320.0602.

Compound previously prepared and reported.[6]

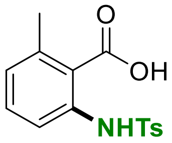

2-Methyl-6-((4-methylphenyl)sulfonamido)benzoic acid (**2c**) was prepared according to General Procedure A. The product was purified by flash column chromatography (10-100 % EtOAc with 2% AcOH in heptane, 25 g SiO_2). The product **2c** was isolated as a beige solid (149.5 mg, 98%).

^{1}H NMR (500 MHz, Acetone-d_6) δ 9.85 (s, 1H), 7.67 (d, J = 8.3 Hz, 2H), 7.49 (d, J = 8.2 Hz, 1H), 7.31-7.37 (m, 3H), 7.03 (d, J = 7.6 Hz, 1H), 2.44 (s, 3H), 2.36 (s, 3H). **^{13}C NMR** (126 MHz, Acetone-d_6) δ 170.6, 144.9, 141.1, 139.6, 137.5, 132.8, 130.6, 128.2, 128.1, 121.6, 119.5, 22.8, 21.4. **HRMS** $C_{15}H_{15}NO_4S$; calcd. for $(M-H^+)$: 304.0649, found 304.0649.

Compound previously prepared and reported.[6]

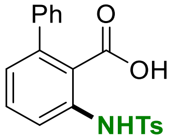

3-((4-Methylphenyl)sulfonamido)-[1,1'-biphenyl]-2-carboxylic acid (**2d**) was prepared according to General Procedure A. The product was purified by HPLC (40-80% MeCN in acidic buffer). The product **2d** was isolated as a beige solid (128.7 mg, 70%).

^{1}H NMR (500 MHz, Acetone-d_6) δ 9.34 (s, 1H), 7.71 (d, J = 8.3 Hz, 2H), 7.65 (dd, J = 8.3, 0.9 Hz, 1H), 7.51 (t, J = 8.0 Hz, 1H), 7.32 – 7.38 (m, 5H), 7.26-7.29 (m, 2H), 7.14 (dd, J = 7.6, 0.9 Hz, 1H), 2.34 (s, 3H). **^{13}C NMR** (126 MHz, Acetone-d_6) δ 170.5, 145.0, 144.3, 142.3, 138.2, 137.5, 132.3, 130.6, 129.0, 129.0, 128.2, 128.0, 127.6, 122.8, 120.9, 21.4. **HRMS** $C_{20}H_{17}NO_4S$; calcd. for (M-H$^+$): 366.0805, found 366.0802.

Compound previously prepared and reported.[6]

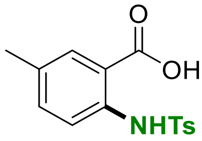

5-Methyl-2-((4-methylphenyl)sulfonamido)benzoic acid (**2e**) was prepared according to General Procedure A. The product was purified by flash column chromatography (10-100 % EtOAc with 2% AcOH in heptane, 25 g SiO_2). The product **2e** was isolated as a beige solid (141.8 mg, 93%).

^{1}H NMR (500 MHz, Acetone-d_6) δ 10.82 (s, 1H), 7.79 – 7.82 (m, 1H), 7.72 (d, J = 8.3 Hz, 2H), 7.61 (d, J = 8.5 Hz, 1H), 7.39 (dd, J = 8.5, 1.9 Hz, 1H), 7.33 (d, J = 8.1 Hz, 2H), 2.35 (s, 3H), 2.28 (s, 3H). **^{13}C NMR** (126 MHz, Acetone-d_6) δ 170.4, 145.0, 139.5, 137.5, 136.3, 133.6, 132.6, 130.6, 128.1, 119.7, 116.6, 21.4, 20.4. **HRMS** $C_{15}H_{15}NO_4S$; calcd. for (M-H$^+$): 304.0649, found 304.0652.

Compound previously prepared and reported.[6]

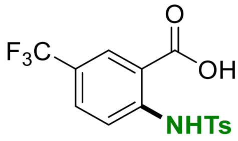

2-((4-Methylphenyl)sulfonamido)-5-(trifluoromethyl)benzoic acid (**2f**) was prepared according to General Procedure A. The product was purified by flash column chromatography (10-100 % EtOAc with 2% AcOH in heptane, 25 g SiO_2). The product **2f** was isolated as a beige solid (167.0 mg, 93%).

^{1}H NMR (500 MHz, Acetone-d_6) δ 11.30 (s, 1H), 8.29 (s, 1H), 7.89 (s, 2H), 7.85 (d, J = 8.4 Hz, 2H), 7.40 (d, J = 8.0 Hz, 2H), 2.38 (s, 3H). **^{13}C NMR** (126 MHz, Acetone-d_6) δ 169.4, 145.7, 145.0, 137.1, 132.1 (q, $J_{C\text{-}F}$ = 3.9 Hz, 1C), 130.9, 129.7 (q, $J_{C\text{-}F}$ = 3.9 Hz, 1C), 128.2, 124.7 (q, $J_{C\text{-}F}$ = 272.2 Hz, 1C), 124.6 (q, $J_{C\text{-}F}$ = 33.4 Hz, 1C), 119.1, 116.3, 21.4. **^{19}F NMR** (471 MHz, Acetone-d_6) δ -62.94. **HRMS** $C_{15}H_{12}F_3NO_4S$; calcd. for (M-H$^+$): 358.0366, found 358.0361.

Compound previously prepared and reported.[6]

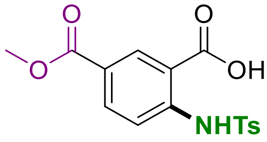

5-(Methoxycarbonyl)-2-((4-methylphenyl)sulfonamido)benzoic acid (**2g**) was prepared according to General Procedure A. The product was purified by flash column chromatography (10-100 % EtOAc with 2% AcOH in heptane, 25 g SiO_2). The product **2g** was isolated as a light brown solid (160.7 mg, 92%).

^{1}H NMR (500 MHz, Acetone-d_6) δ 8.64 (d, J = 2.0 Hz, 1H), 8.13 (dd, J = 8.8, 2.1 Hz, 1H), 7.84 (d, J = 8.3 Hz, 2H), 7.79 (d, J = 8.8 Hz, 1H), 7.38 (d, J = 8.1 Hz, 2H), 3.86 (s, 3H), 2.37 (s, 3H). **^{13}C NMR** (126 MHz,

Acetone-d_6) δ 170.0, 165.9, 145.6, 145.6, 137.2, 136.1, 134.0, 130.8, 128.2, 125.1, 118.4, 116.0, 52.5, 21.4. **HRMS** $C_{16}H_{15}NO_6S$; calcd. for (M-H$^+$): 348.0547, found 348.0550.

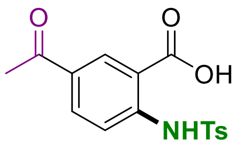

5-Acetyl-2-((4-methylphenyl)sulfonamido)benzoic acid (**2h**) was prepared according to General Procedure A. The product was purified by flash column chromatography (10-100 % EtOAc with 2% AcOH in heptane, 25 g SiO_2). The product **2h** was isolated as a light brown solid (150.1 mg, 90%).

^{1}**H NMR** (500 MHz, Acetone-d_6) δ 11.39 (s, 1H), 8.62 (d, J = 2.1 Hz, 1H), 8.15 (dd, J = 8.8, 2.1 Hz, 1H), 7.85 (d, J = 8.3 Hz, 2H), 7.77 (d, J = 8.8 Hz, 1H), 7.39 (d, J = 8.2 Hz, 2H), 2.55 (s, 3H), 2.38 (s, 3H). ^{13}**C NMR** (126 MHz, Acetone-d_6) δ 195.9, 170.1, 145.6, 145.4, 137.2, 135.2, 133.0, 132.3, 130.9, 128.2, 118.1, 115.7, 26.4, 21.4. **HRMS** $C_{16}H_{15}NO_5S$; calcd. for (M-H$^+$): 332.0598, found 332.0598.

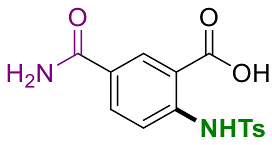

5-Carbamoyl-2-((4-methylphenyl)sulfonamido)benzoic acid (**2i**) was prepared according to General Procedure A. The product was purified by HPLC (17-57% MeCN in acidic buffer). The product **2i** was isolated as an off-white solid (143.7 mg, 86%).

^{1}**H NMR** (500 MHz, CD_3OD) δ 8.52 (d, J = 2.2 Hz, 1H), 7.96 (dd, J = 8.7, 2.2 Hz, 1H), 7.7 – 7.76 (m, 3H), 7.31 (d, J = 8.0 Hz, 2H), 2.35 (s, 3H). ^{13}**C NMR** (126 MHz, CD_3OD) δ 170.9, 170.5, 146.0, 144.7, 137.4, 134.3, 132.7, 130.9, 129.4, 128.4, 119.2, 117.3, 21.5. **HRMS** $C_{15}H_{14}N_2O_5S$; calcd. for (M-H$^+$): 333.0550, found 333.0546.

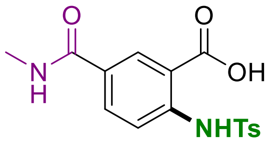

5-(Methylcarbamoyl)-2-((4-methylphenyl)sulfonamido)benzoic acid (**2j**) was prepared according to General Procedure A. The product was purified by HPLC (38-78% MeCN in acidic buffer). The product **2j** was isolated as a colorless solid (59.1 mg, 34%).

^{1}**H NMR** (500 MHz, CD_3OD) δ 8.45 (s, 1H), 7.89 (d, J = 8.7 Hz, 1H), 7.68 – 7.75 (m, 3H), 7.30 (d, J = 8.0 Hz, 2H), 2.87 (s, 3H), 2.34 (s, 3H). ^{13}**C NMR** (126 MHz, CD_3OD) δ 170.9, 168.9, 146.0, 144.4, 137.4, 133.8, 132.1, 130.9, 130.0, 128.4, 119.3, 117.3, 26.9, 21.4. **HRMS** $C_{16}H_{16}N_2O_5S$; calcd. for (M-H$^+$): 347.0707, found 347.0710.

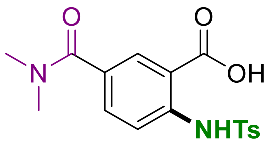

5-(Dimethylcarbamoyl)-2-((4-methylphenyl)sulfonamido)benzoic acid (**2k**) was prepared according to General Procedure A. The product was purified by HPLC (24-64% MeCN in acidic buffer). The product **2k** was isolated as an off-white solid (170.2 mg, 94%).

^{1}H NMR (500 MHz, DMSO-d_6) δ 11.23 (s, 1H), 7.90 (s, 1H), 7.75 (d, J = 8.0 Hz, 2H), 7.60 (d, J = 8.5 Hz, 1H), 7.53 (d, J = 8.5 Hz, 1H), 7.38 (d, J = 8.0 Hz, 2H), 2.93 (s, 3H), 2.87 (s, 3H), 2.34 (s, 3H). **^{13}C NMR** (126 MHz, DMSO-d_6) δ 169.2, 168.5, 144.4, 140.7, 140.5, 135.6, 130.8, 130.5, 130.1, 127.0, 117.6, 116.0, 39.5, 34.9, 21.0. **HRMS** $C_{17}H_{18}N_2O_5S$; calcd. for (M-H$^+$): 361.0863, found 361.0865.

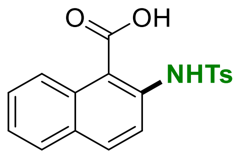

2-((4-Methylphenyl)sulfonamido)-1-naphthoic acid (**2l**) was prepared according to General Procedure A. The product was purified by HPLC (37-77% MeCN in acidic buffer). The product **2l** was isolated as a gray solid (150.1 mg, 88%).

^{1}H NMR (500 MHz, Acetone-d_6) δ 10.12 (s, 1H), 8.48 (d, J = 8.7 Hz, 1H), 8.07 (d, J = 9.0 Hz, 1H), 7.91 (d, J = 8.1 Hz, 1H), 7.88 (d, J = 9.0 Hz, 1H), 7.70 (d, J = 8.3 Hz, 2H), 7.57 (ddd, J = 8.5, 6.9, 1.3 Hz, 1H), 7.47 – 7.51 (m, 1H), 7.30 (d, J = 8.1 Hz, 2H), 2.32 (s, 3H). **^{13}C NMR** (126 MHz, Acetone-d_6) δ 170.3, 145.08, 138.8, 137.4, 134.5, 132.1, 131.7, 130.7, 129.4, 128.9, 128.0, 126.9, 126.4, 120.7, 117.2, 21.4. **HRMS** $C_{18}H_{15}NO_4S$; calcd. for (M-H$^+$): 340.0649, found 340.0647.

Compound previously prepared and reported.[6]

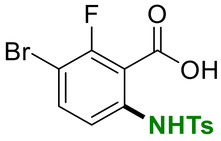

3-Bromo-2-fluoro-6-((4-methylphenyl)sulfonamido)benzoic acid (**2m**) was prepared according to General Procedure A. The product was purified by flash column chromatography (10-100 % EtOAc with 2% AcOH in heptane, 25 g SiO_2). The product **2m** was isolated as a light brown solid (137.9 mg, 71%).

^{1}H NMR (500 MHz, Acetone-d_6) δ 10.43 (s, 1H), 7.82 (dd, J = 9.0, 7.4 Hz, 1H), 7.74 (d, J = 8.3 Hz, 2H), 7.48 (dd, J = 9.0, 1.4 Hz, 1H), 7.37 (d, J = 8.1 Hz, 2H), 2.38 (s, 3H). **^{13}C NMR** (126 MHz, Acetone-d_6) δ 167.3 (d, J_{C-F} =3.5 Hz, 1C), 159.2 (d, J_{C-F} = 259.6 Hz, 1C), 145.5, 141.4, 138.4 (d, J_{C-F} = 2.5 Hz, 1C), 136.9, 130.8, 128.1, 117.5 (d, J_{C-F} = 4.1 Hz, 1C), 110.1 (d, J_{C-F} = 15.1 Hz, 1C), 104.7 (d, J_{C-F} = 23.3 Hz, 1C), 21.4. **^{19}F NMR** (471 MHz, Acetone-d_6) δ -97.79. **HRMS** $C_{14}H_{11}BrFNO_4S$; calcd. for (M-H$^+$): 385.9503, found 385.9510.

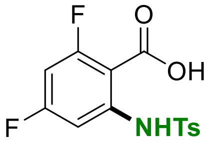

2,4-Difluoro-6-((4-methylphenyl)sulfonamido)benzoic acid (**2n**) was prepared according to General Procedure A. The product was purified by flash column chromatography (10-100 % EtOAc with 2% AcOH in heptane, 25 g SiO_2). The product **2n** was isolated as a light yellow solid (137.5 mg, 84%).

^{1}**H NMR** (500 MHz, CD_3OD) δ 7.70 (d, *J* = 8.4 Hz, 2H), 7.35 (d, *J* = 8.0 Hz, 2H), 7.2 – 7.25 (m, 1H), 6.73 (ddd, *J* = 11.3, 8.9, 2.5 Hz, 1H), 2.39 (s, 3H). ^{13}**C NMR** (126 MHz, CD_3OD) δ 168.7 (d, J_{C-F} = 3.2 Hz, 1C), 166.4 (dd, J_{C-F} = 253.3, 15.8 Hz, 1C), 165.1 (dd, J_{C-F} = 262.1, 15.1 Hz, 1C), 146.2, 144.3 (dd, J_{C-F} = 14.5, 5.5 Hz, 1C), 137.0, 131.0, 128.4, 105.6 (dd, J_{C-F} = 13.9, 3.8 Hz, 1C), 103.7 (dd, J_{C-F} = 27.7, 3.7 Hz, 1C), 101.1 (dd, J_{C-F} = 29.0, 26.5 Hz, 1C), 21.5. ^{19}**F NMR** (471 MHz, CD_3OD) δ -100.28 (d, *J* = 13.4 Hz), -102.90 (d, *J* = 13.4 Hz). **HRMS** $C_{14}H_{11}F_2NO_4S$; calcd. for ($M-H^+$): 326.0304, found 326.0304.

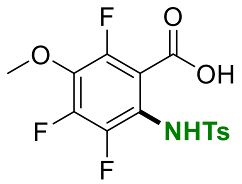

2,4,5-Trifluoro-3-methoxy-6-((4-methylphenyl)sulfonamido)benzoic acid (**2o**) was prepared according to General Procedure A. The product was purified by flash column chromatography (10-100 % EtOAc with 2% AcOH in heptane, 25 g SiO_2). The product **2o** was isolated as a light brown solid (180.3 mg, 96%).

^{1}**H NMR** (500 MHz, CD_3OD) δ 7.59 (d, *J* = 8.3 Hz, 2H), 7.33 (d, *J* = 8.4 Hz, 2H), 4.02 (s, 3H), 2.43 (s, 3H). ^{13}**C NMR** (126 MHz, CD_3OD) δ 164.9, 150.8 (dq, J_{C-F} = 252.0, 5.5, 3.5 Hz, 1C), 147.2 (ddd, J_{C-F} = 253.3, 13.9, 6.9 Hz, 1C), 146.2 (ddd, J_{C-F} = 205.7, 12.0, 3.8 Hz, 1C), 145.5, (ddd, J_{C-F} = 16.4, 11.3, 1.4 Hz, 1C), 138.1, 130.7, 128.4, 120.0 (m, 1C), 118.7 (dd, J_{C-F} = 15.1, 3.8 Hz, 1C), 62.9, 21.5. ^{19}**F NMR** (471 MHz, CD_3OD) δ -134.11 (dd, *J* = 10.4, 7.5 Hz), -146.29 (dd, *J* = 20.2, 10.7 Hz), -149.80 (dd, *J* = 20.2, 7.4 Hz). **HRMS** $C_{15}H_{12}F_3NO_5S$; calcd. for ($M-H^+$): 374.0315, found 374.0316.

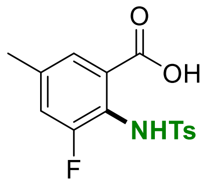

3-Fluoro-5-methyl-2-((4-methylphenyl)sulfonamido)benzoic acid (**2p**) was prepared according to General Procedure A. The product was purified by flash column chromatography (10-100 % EtOAc with 2% AcOH in heptane, 25 g SiO_2). The product **2p** was isolated as a beige solid (129.4 mg, 80%).

^{1}**H NMR** (500 MHz, Acetone-d_6) δ 7.63 (d, *J* = 8.3 Hz, 2H), 7.58 (s, 1H), 7.34 (d, *J* = 8.1 Hz, 2H), 7.28 (d, *J* = 11.3 Hz, 1H), 2.40 (s, 3H), 2.37 (s, 3H). ^{13}**C NMR** (126 MHz, Acetone-d_6) δ 168.5 (d, J_{C-F} = 3.8 Hz, 1C), 157.6 (d, J_{C-F} = 250.7 Hz, 1C), 144.6, 138.1 (d, J_{C-F} = 1.6 Hz, 1C), 138.0 (d, J_{C-F} = 7.8 Hz, 1C), 130.2, 128.1 (d, J_{C-F} = 0.6 Hz, 1C), 127.8 (d, J_{C-F} = 3.0 Hz, 1C), 125.7 (d, J_{C-F} = 13.9 Hz, 1C), 125.4 (d, J_{C-F} = 2.1 Hz, 1C), 122.4 (d, J_{C-F} = 20.8 Hz, 1C), 21.4, 20.7 (d, J_{C-F} = 1.3 Hz, 1C). ^{19}**F NMR** (471 MHz, Acetone-d_6) δ -118.26. **HRMS** $C_{15}H_{14}FNO_4S$; calcd. for ($M-H^+$): 322.0555, found 322.0553.

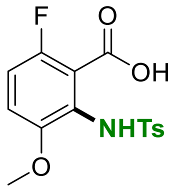

6-Fluoro-3-methoxy-2-((4-methylphenyl)sulfonamido)benzoic acid (**2q**) was prepared according to General Procedure A. The product was purified by flash column chromatography (10-100 % EtOAc with 2% AcOH in heptane, 25 g SiO_2). The product **2q** was isolated as a beige solid (145.8 mg, 86%).

^{1}H NMR (500 MHz, Acetone-d_6) δ 8.44 (s, 1H), 7.61 (d, J = 8.3 Hz, 2H), 7.34 (d, J = 8.0 Hz, 2H), 7.09 (t, J = 9.3 Hz, 1H), 7.02 (dd, J = 9.2, 4.8 Hz, 1H), 3.35 (s, 3H), 2.40 (s, 3H). **^{13}C NMR** (126 MHz, Acetone-d_6) δ 164.8, 154.8 (d, $J_{C\text{-}F}$ = 245.7 Hz, 1C), 151.9 (d, $J_{C\text{-}F}$ = 2.5 Hz, 1C), 144.0, 139.2, 129.8, 128.0, 125.8 (d, $J_{C\text{-}F}$ = 5.0 Hz, 1C), 121.6 (d, $J_{C\text{-}F}$ = 18.3 Hz, 1C), 115.2 (d, $J_{C\text{-}F}$ = 23.9 Hz, 1C), 115.0 (d, $J_{C\text{-}F}$ = 9.5 Hz, 1C), 56.2, 21.4. **^{19}F NMR** (471 MHz, Acetone-d_6) δ -123.24. **HRMS** $C_{15}H_{14}FNO_5S$; calcd. for (M-H$^+$): 338.0504, found 338.0503.

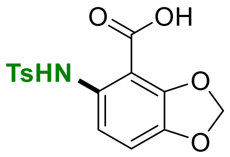

5-((4-Methylphenyl)sulfonamido)benzo[d][1,3]dioxole-4-carboxylic acid (**2r**) was prepared according to General Procedure A. The product was purified by flash column chromatography (10-100 % EtOAc with 2% AcOH in heptane, 25 g SiO_2). The product **2r** was isolated as a brown solid (162.7 mg, 97%).

^{1}H NMR (500 MHz, Acetone-d_6) δ 10.38 (s, 1H), 7.63 (d, J = 8.3 Hz, 2H), 7.31 (d, J = 8.2 Hz, 2H), 7.15 (d, J = 8.6 Hz, 1H), 7.02 (d, J = 8.6 Hz, 1H), 6.07 (s, 2H), 2.36 (s, 3H). **^{13}C NMR** (126 MHz, Acetone-d_6) δ 168.5, 150.6, 145.9, 144.8, 137.2, 134.3, 130.5, 128.1, 113.8, 113.1, 103.8, 103.4, 21.4. **HRMS** $C_{15}H_{13}NO_6S$; calcd. for (M-H$^+$): 334.0391, found 334.0391.

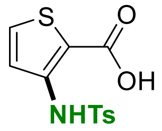

3-((4-Methylphenyl)sulfonamido)thiophene-2-carboxylic acid (**2s**) was prepared according to General Procedure A. The product was purified by HPLC (15-55% MeCN in acidic buffer). The product **2s** was isolated as a brown solid (106.9 mg, 72%).

^{1}H NMR (500 MHz, Acetone-d_6) δ 7.75 – 7.8 (m, 3H), 7.36 – 7.41 (m, 3H), 2.38 (s, 3H). **^{13}C NMR** (126 MHz, Acetone-d_6) δ 165.7, 145.4, 144.7, 137.5, 133.5, 130.8, 127.9, 121.2, 111.9, 21.4. **HRMS** $C_{12}H_{11}NO_4S_2$; calcd. for (M-H$^+$): 296.0056, found 296.0051.

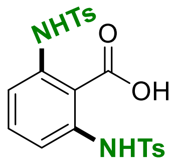

2,6-Bis((4-methylphenyl)sulfonamido)benzoic acid (**2t**) was prepared according to modified General Procedure A. TsN3 (161 µL, 1.05 mmol) was used. The product was purified by flash column chromatography (10-100 % EtOAc with 2% AcOH in heptane, 25 g SiO_2). The product **2t** was isolated as an off-white solid (218.9 mg, 95%).

^{1}H NMR (500 MHz, CD_3OD) δ 7.48 (d, J = 8.2 Hz, 4H), 7.19 – 7.23 (m, 2H), 7.14 (dd, J = 9.3, 7.1 Hz, 1H), 6.69 (d, J = 8.1 Hz, 4H), 1.96 (s, 6H). **^{13}C NMR** (126 MHz, CD_3OD) δ 177.0, 144.7, 143.2, 137.5, 132.8, 130.3, 128.2, 113.4, 110.1, 21.3. **HRMS** $C_{21}H_{20}N_2O_6S_2$; calcd. for (M-H$^+$): 459.0690, found 459.0686.

Compound previously prepared and reported.[7]

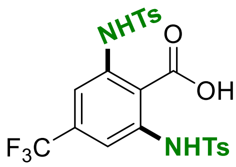

2,6-Bis((4-methylphenyl)sulfonamido)-4-(trifluoromethyl)benzoic acid (**2u**) was prepared according to modified General Procedure A. TsN3 (161 µL, 1.05 mmol) was used. The product was purified by flash column chromatography (10-100 % EtOAc with 2% AcOH in heptane, 25 g SiO_2). The product **2u** was isolated as a beige solid (256.2 mg, 97%).

^{1}H NMR (500 MHz, CD_3OD) δ 7.51 – 7.57 (m, 6H), 7.28 (d, *J* = 8.1 Hz, 4H), 2.38 (s, 6H). **^{13}C NMR** (126 MHz, CD_3OD) δ 168.7, 146.1, 141.8, 136.9, 135.0 (q, J_{C-F} = 33.4 Hz, 1C), 130.9, 128.3, 125.3, 123.1, 114.5 (q, J_{C-F} = 3.7 Hz, 1C), 21.5. **^{19}F NMR** (471 MHz, CD_3OD) δ -65.77 **HRMS** $C_{22}H_{19}F_3N_2O_6S_2$; calcd. for $(M-H^+)$: 527.0564, found 527.0567.

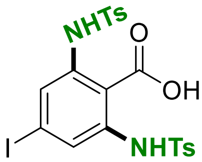

4-Iodo-2,6-bis((4-methylphenyl)sulfonamido)benzoic acid (**2v**) was prepared according to modified General Procedure A. TsN3 (161 µL, 1.05 mmol) was used. The reaction mixture was stirred at 800 rpm. The product was purified by flash column chromatography (10-100 % EtOAc with 2% AcOH in heptane, 25 g SiO_2). The product **2v** was isolated as a beige solid (287.0 mg, 98%).

^{1}H NMR (500 MHz, DMSO-d_6) δ 7.57 (d, *J* = 8.3 Hz, 4H), 7.33 (d, *J* = 8.0 Hz, 4H), 7.29 (s, 2H), 2.33 (s, 6H). **^{13}C NMR** (126 MHz, DMSO-d_6) δ 170.6, 143.4, 142.5, 136.6, 129.8, 126.5, 120.4, 109.2, 98.3, 21.0. **HRMS** $C_{21}H_{19}IN_2O_6S_2$; calcd. for $(M-H^+)$: 584.9656, found 584.9649.

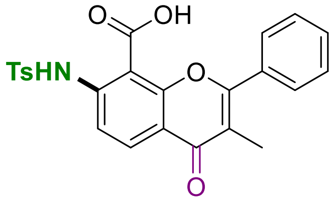

3-Methyl-7-((4-methylphenyl)sulfonamido)-4-oxo-2-phenyl-4*H*-chromene-8-carboxylic acid (**2w**) was prepared according to General Procedure A. The product was purified by HPLC (38-78% MeCN in acidic buffer). The product **2w** was isolated as a colorless solid (94.5 mg, 42%).

^{1}H NMR (500 MHz, DMSO-d_6) δ 8.14 (d, *J* = 8.7 Hz, 1H), 7.82 – 7.87 (m, 4H), 7.56 – 7.61 (m, 3H), 7.42 (t, *J* = 7.8 Hz, 3H), 2.35 (s, 3H), 2.11 (s, 3H). **^{13}C NMR** (126 MHz, DMSO-d_6) δ 181.6, 164.6, 160.9, 155.1, 144.6, 142.5, 137.5, 135.4, 131.8, 131.1, 130.2, 129.4, 128.5, 127.1, 116.9, 109.8, 109.1, 21.0, 11.1. **HRMS** $C_{24}H_{19}NO_6S$; calcd. for $(M-H^+)$: 448.0860, found 448.0864.

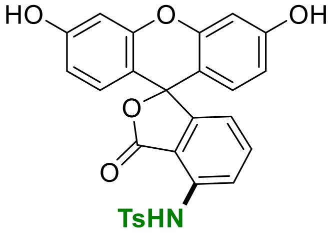

***N*-(3',6'-Dihydroxy-3-oxo-3*H*-spiro[isobenzofuran-1,9'-xanthen]-4-yl)-4-methylbenzenesulfonamide** (**2x**) was prepared according to General Procedure A. The product was purified by HPLC (43-83% MeCN in acidic buffer). The product **2x** was isolated as a dark yellow solid (190.5 mg, 76%).

^{1}H NMR (500 MHz, CD_3OD) δ 7.77 (d, *J* = 8.3 Hz, 2H), 7.69 (d, *J* = 7.9 Hz, 1H), 7.64 (t, *J* = 7.8 Hz, 1H), 7.37 (d, *J* = 8.1 Hz, 2H), 6.81 (d, *J* = 7.3 Hz, 1H), 6.64 (d, *J* = 2.4 Hz, 2H), 6.47 (dd, *J* = 8.7, 2.4 Hz, 2H), 6.30 (d, *J* = 8.7 Hz, 2H), 2.43 (s, 3H). **^{13}C NMR** (126 MHz, CD_3OD) δ 170.9, 161.2, 155.0, 153.9, 146.0, 137.9, 137.8, 137.2, 130.8, 130.0, 128.7, 121.4, 120.7, 116.8, 113.4, 110.6, 103.5, 21.6. **HRMS** $C_{27}H_{19}NO_7S$; calcd. for (M-H$^+$): 500.0809, found 500.0805.

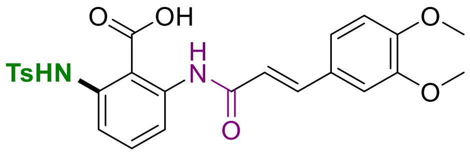

(*E*)-2-(3-(3,4-Dimethoxyphenyl)acrylamido)-6-((4-methylphenyl)sulfonamido)benzoic acid (**2y**) was prepared according to General Procedure A. The product was purified by HPLC (33-73% MeCN in acidic buffer). The product **2y** was isolated as an orange solid (173.7 mg, 70%).

^{1}H NMR (500 MHz, DMSO-*d*$_6$) δ 10.74 (s, 1H), 10.59 (s, 1H), 7.67 (d, *J* = 8.1 Hz, 1H), 7.63 (d, *J* = 8.3 Hz, 2H), 7.49 (d, *J* = 15.6 Hz, 1H), 7.33 – 7.4 (m, 3H), 7.26 (d, *J* = 1.8 Hz, 1H), 7.18 (dd, *J* = 8.3, 1.8 Hz, 1H), 7.09 (dd, *J* = 8.2, 0.9 Hz, 1H), 7.00 (d, *J* = 8.4 Hz, 1H), 6.69 (d, *J* = 15.6 Hz, 1H), 3.81 (s, 3H), 3.79 (s, 3H), 2.34 (s, 3H). **^{13}C NMR** (126 MHz, DMSO-*d*$_6$) δ 168.7, 164.0, 150.5, 148.9, 143.6, 141.0, 138.8, 137.7, 136.3, 131.7, 129.8, 127.3, 126.9, 122.2, 119.7, 118.9, 116.5, 115.3, 111.7, 110.1, 55.6, 55.5, 21.0. **HRMS** $C_{25}H_{24}N_2O_7S$; calcd. for (M-H$^+$): 495.1231, found 495.1231.

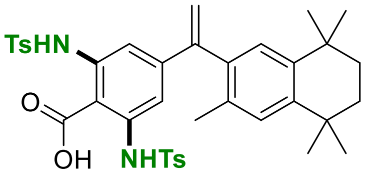

2,6-Bis((4-methylphenyl)sulfonamido)-4-(1-(3,5,5,8,8-pentamethyl-5,6,7,8-tetrahydronaphthalen-2-yl)vinyl)benzoic acid (**2z**) was prepared according to modified General Procedure A. TsN3 (161 µL, 1.05 mmol). KOAc (49 mg, 0.5 mmol), [Cp*Ir(H2O)3]SO4 (14.6 mg, 0.03 mmol) was used. The product was purified by flash column chromatography (10-100 % EtOAc with 2% AcOH in heptane, 25 g SiO_2). The product **2z** was isolated as a light brown solid (326.2 mg, 95%).

^{1}H NMR (500 MHz, CD_3OD) δ 7.28 (d, *J* = 8.2 Hz, 4H), 7.19 (s, 1H), 7.08 (s, 2H), 7.04 (s, 1H), 6.70 (d, *J* = 8.0 Hz, 4H), 5.65 – 5.68 (m, 1H), 5.16 – 5.19 (m, 1H), 2.01 (s, 6H), 1.75 (s, 4H), 1.69 (s, 3H), 1.35 (s, 6H), 1.31 (s, 6H). **^{13}C NMR** (126 MHz, CD_3OD) δ 176.5, 175.2, 150.8, 145.5, 145.1, 144.6, 143.4, 143.1, 139.1, 137.3, 133.8, 130.3, 129.1, 129.0, 128.3, 116.8, 111.2, 40.4, 36.40, 36.36, 35.0, 34.9, 32.44, 32.40, 21.4, 20.0. **HRMS** $C_{38}H_{42}N_2O_6S_2$; calcd. for (M-H$^+$): 685.2411, found 685.2416.

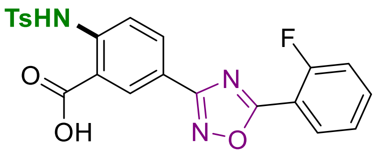

5-(5-(2-Fluorophenyl)-1,2,4-oxadiazol-3-yl)-2-((4-methylphenyl)sulfonamido)benzoic acid (**2aa**) was prepared according to General Procedure A. The product was purified by HPLC (43-83% MeCN in acidic buffer). The product **2aa** was isolated as an off-white solid (49.9 mg, 22%).

^{1}**H NMR** (500 MHz, DMSO-d_6) δ 8.59 (d, J = 2.1 Hz, 1H), 8.19 – 8.24 (m, 2H), 7.80 (d, J = 8.3 Hz, 3H), 7.73 (d, J = 8.7 Hz, 1H), 7.54 (dd, J = 10.4, 8.5 Hz, 1H), 7.46 – 7.5 (m, 1H), 7.39 (d, J = 8.1 Hz, 2H), 2.34 (s, 3H). ^{13}**C NMR** (126 MHz, DMSO-d_6) δ 172.6 (d, J_{C-F} = 4.4 Hz, 1C), 169.2, 166.9, 160.0 (d, J_{C-F} = 258.3 Hz, 1C), 144.4, 142.8, 135.9 (d, J_{C-F} = 9.1 Hz, 1C), 135.7, 132.7, 131.0, 130.4, 130.2, 127.1, 125.6 (d, J_{C-F} = 3.5 Hz, 1C), 120.24, 118.6, 117.5, 117.3, 117.0, 111.8, 111.7, 21.1. ^{19}**F NMR** (471 MHz, DMSO-d_6) δ -109.47. **HRMS** $C_{22}H_{16}FN_3O_5S$; calcd. for (M-H$^+$): 452.0722, found 452.0725.

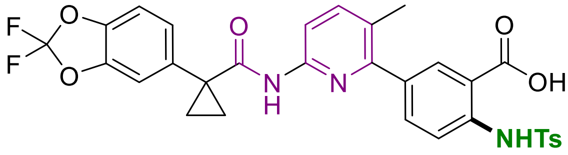

5-(6-(1-(2,2-Difluorobenzo[d][1,3]dioxol-5-yl)cyclopropane-1-carboxamido)-3-methylpyridin-2-yl)-2-((4-methylphenyl)sulfonamido)benzoic acid (**2ab**) was prepared according to General Procedure A. The product was purified by HPLC (20-60% MeCN in acidic buffer). The product **2ab** was isolated as an off-white solid (49.5 mg, 15%). Unreacted starting material (158.2 mg, 70%) was also recovered.

^{1}**H NMR** (500 MHz, DMSO-d_6) δ 11.17 (s, 1H), 9.14 (s, 1H), 7.97 (d, J = 2.2 Hz, 1H), 7.89 (d, J = 8.4 Hz, 1H), 7.75 (d, J = 8.3 Hz, 3H), 7.67 (dd, J = 8.6, 2.2 Hz, 1H), 7.55 (d, J = 8.6 Hz, 1H), 7.52 (d, J = 1.7 Hz, 1H), 7.37 (dd, J = 8.2, 6.1 Hz, 3H), 7.30 (dd, J = 8.3, 1.7 Hz, 1H), 2.34 (s, 3H), 2.18 (s, 3H), 1.46 – 1.52 (m, 2H), 1.13 – 1.18 (m, 2H). ^{13}**C NMR** (126 MHz, DMSO-d_6) δ 171.4, 169.6, 153.1, 148.8, 144.3, 142.8, 142.1, 141.6, 139.7, 136.1, 135.7, 134.9, 133.3, 132.0, 131.2, 130.1, 129.2, 127.0, 126.6, 126.5, 117.6, 116.1, 113.6, 112.3, 110.1, 31.4, 21.0, 18.7, 15.8. ^{19}**F NMR** (471 MHz, DMSO-d_6) δ -48.78. **HRMS** $C_{31}H_{25}F_2N_3O_7S$; calcd. for (M-H$^+$): 620.1308, found 620.1305.

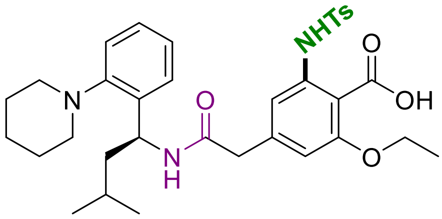

(*S*)-2-Ethoxy-4-(2-((3-methyl-1-(2-(piperidin-1-yl)phenyl)butyl)amino)-2-oxoethyl)-6-((4-methylphenyl)sulfonamido)benzoic acid (**2ac**) was prepared according to General Procedure A. The product was purified by HPLC (60-100% MeCN in acidic buffer). The product **2ac** was isolated as a light brown solid (74.6 mg, 24%).

^{1}**H NMR** (500 MHz, CD_3OD) δ 7.56 (d, J = 8.2 Hz, 2H), 7.33 (d, J = 7.5 Hz, 1H), 7.21 (s, 3H), 7.17 (d, J = 8.1 Hz, 2H), 7.07 – 7.14 (m, 1H), 6.69 (s, 1H), 5.55 (s, 1H), 3.97 (q, J = 7.0 Hz, 2H), 3.51 (s, 2H), 3.12 (s, 2H), 2.73 (s, 2H), 2.32 (s, 3H), 1.81 (s, 2H), 1.45 – 1.74 (m, 7H), 1.31 (t, J = 7.0 Hz, 3H), 0.97 (dd, J = 6.4, 2.7 Hz, 6H). ^{13}**C NMR** (126 MHz, CD_3OD) δ 172.0, 170.3, 159.7, 145.3, 142.9, 140.4, 137.4, 130.7, 129.0, 128.4, 127.5, 126.3, 122.2, 115.5, 111.6, 110.9, 66.3, 56.2, 48.2, 43.9, 27.6, 26.6, 25.1, 23.6, 22.6, 21.5, 14.8. **HRMS** $C_{34}H_{43}N_3O_6S$; calcd. for (M-H$^+$): 620.2800, found 620.2796.

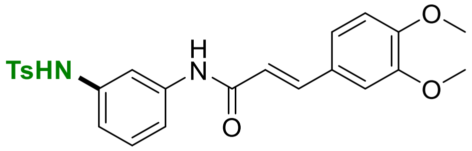

(*E*)-3-(3,4-Dimethoxyphenyl)-N-(3-((4-methylphenyl)sulfonamido)phenyl)acrylamide (**2ya**).

(*E*)-2-(3-(3,4-Dimethoxyphenyl)acrylamido)-6-((4-methylphenyl)sulfonamido)benzoic acid (**2y**, 36.0 mg, 0.07 mmol), Cu2O (5.2 mg, 0.04 mmol) and 1,10-phenantroline (13.1 mg, 0.07 mmol) were added to a microwave vial. DMA (0.25 mL) was added and the sealed reaction mixture stirred at 170 °C for 13 h. The reaction mixture was partitioned between aqueous HCL (1M, 30 mL) and EtOAc (30 mL). The aqeous phase was extracted with EtOAc (2x30 mL) and the combined organic phases dried over MgSO4. The product was purified by flash column chromatography (EtOAc in heptane, 20-80%, 25 g SiO_2) and **2ya** (10.2 mg, 31%) obtained as a colorless solid.

^{1}H NMR (500 MHz, DMSO-d_6) δ 10.26 (s, 1H), 10.11 (s, 1H), 7.68 (d, *J* = 8.3 Hz, 2H), 7.55 (s, 1H), 7.50 (d, *J* = 15.6 Hz, 1H), 7.39 (d, *J* = 8.4 Hz, 1H), 7.34 (d, *J* = 8.0 Hz, 2H), 7.19 (s, 1H), 7.16 – 7.19 (m, 1H), 7.13 (t, *J* = 8.1 Hz, 1H), 7.02 (d, *J* = 8.3 Hz, 1H), 6.73 – 6.78 (m, 1H), 6.67 (d, *J* = 15.6 Hz, 1H), 3.82 (s, 3H), 3.80 (s, 3H), 2.33 (s, 3H). **^{13}C NMR** (126 MHz, DMSO-d_6) δ 163.9, 150.4, 148.9, 143.3, 140.4, 140.1, 138.3, 136.7, 129.7, 129.4, 127.4, 126.8, 121.9, 119.7, 114.6, 114.4, 111.8, 110.2, 109.9, 55.6, 55.4, 21.0. **HRMS** $C_{24}H_{24}N_2O_5S$; calcd. for (M+H$^+$): 453.1479, found 453.1478.

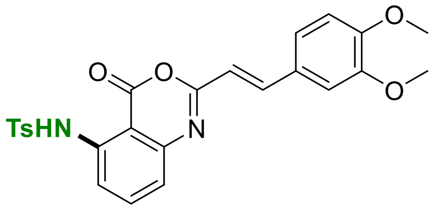

(*E*)-*N*-(2-(3,4-Dimethoxystyryl)-4-oxo-4H-benzo[d][1,3]oxazin-5-yl)-4-methylbenzenesulfonamide (**2yb**).

(*E*)-2-(3-(3,4-Dimethoxyphenyl)acrylamido)-6-((4-methylphenyl)sulfonamido)benzoic acid (**2y**, 28.9 mg, 0.06 mmol) was added to a microwave vial. DCE (0.25 mL) was added and the sealed reaction mixture stirred at 130 °C for 13 h. The volatiles were removed, and the residue co-evaporated with toluene (1 mL). The obtained solids were dried *in vacuo* for 3 days. The product was obtained pure as an yellow solid **2yb** (27.6 mg, 99%).

^{1}H NMR (500 MHz, DMSO-d_6) δ 10.66 (s, 1H), 7.86 (d, *J* = 8.4 Hz, 2H), 7.76 (t, *J* = 8.2 Hz, 1H), 7.71 (d, *J* = 16.0 Hz, 1H), 7.47 (d, *J* = 1.9 Hz, 1H), 7.39 – 7.44 (m, 3H), 7.35 (dd, *J* = 8.4, 1.9 Hz, 1H), 7.19 (dd, *J* = 8.0, 0.7 Hz, 1H), 7.01 (d, *J* = 8.4 Hz, 1H), 6.92 (d, *J* = 16.1 Hz, 1H), 3.83 (s, 3H), 3.81 (s, 3H), 2.35 (s, 3H). **^{13}C NMR** (126 MHz, DMSO-d_6) δ 161.2, 156.9, 151.2, 149.1, 147.9, 144.6, 142.2, 139.4, 137.6, 135.4, 130.2, 127.28, 127.25, 123.2, 120.6, 116.0, 114.6, 111.6, 110.4, 104.5, 55.7, 55.6, 21.0. **HRMS** $C_{25}H_{22}N_2O_6S$; calcd. for (M+H$^+$): 479.1272, found 479.1274.

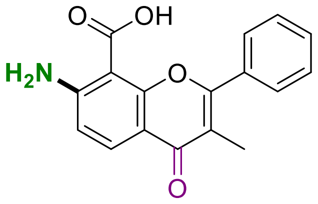

7-Amino-3-methyl-4-oxo-2-phenyl-4*H*-chromene-8-carboxylic acid (**3a**) was prepared according to General Procedure B. The product was purified by HPLC (26-66% MeCN in acidic buffer). The product **3a** was isolated as a colorless solid (35.5 mg, 24%).

^{1}**H NMR** (500 MHz, DMSO-d_6) δ 7.85 (d, *J* = 9.0 Hz, 1H), 7.77 (dd, *J* = 6.7, 3.0 Hz, 2H), 7.54 (dq, *J* = 6.2, 3.5 Hz, 3H), 7.35 (s, 2H), 6.84 (d, *J* = 9.0 Hz, 1H), 2.04 (s, 3H). ^{13}**C NMR** (126 MHz, DMSO-d_6) δ 175.9, 167.7, 158.8, 156.9, 155.2, 133.0, 130.2, 129.1, 128.7, 128.4, 116.0, 115.0, 111.5, 98.8, 11.3. **HRMS** $C_{17}H_{13}NO_4$; calcd. for (M-H$^+$): 294.0772, found 294.0772.

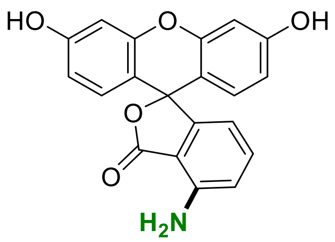

4-Amino-3',6'-dihydroxy-3*H*-spiro[isobenzofuran-1,9'-xanthen]-3-one (**3b**) was prepared according to General Procedure B. Reaction time in the C–H amination step increased to 44 h. The product was purified by HPLC (27-67% MeCN in acidic buffer). The product **3b** was isolated as an orange solid (72.6 mg, 42%).

^{1}**H NMR** (500 MHz, CD_3OD) δ 7.35 – 7.39 (m, 1H), 6.77 (d, *J* = 8.2 Hz, 1H), 6.68 (d, *J* = 8.7 Hz, 2H), 6.64 (d, *J* = 2.4 Hz, 2H), 6.53 (dd, *J* = 8.7, 2.4 Hz, 2H), 6.23 (d, *J* = 7.3 Hz, 1H). ^{13}**C NMR** (126 MHz, CD_3OD) δ 173.0, 160.8, 155.5, 153.8, 149.2, 137.8, 130.2, 115.2, 113.2, 112.1, 111.6, 109.7, 103.3, 85.1. **HRMS** $C_{20}H_{13}NO_5$; calcd. for (M-H$^+$): 346.0721, found 346.0717.

Compound previously prepared and reported.[8]

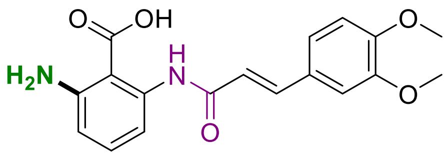

(*E*)-2-Amino-6-(3-(3,4-dimethoxyphenyl)acrylamido)benzoic acid (**3c**) was prepared according to General Procedure B. The product was purified by HPLC (23-63% MeCN in acidic buffer). The product **3c** was isolated as a beige solid (85.7 mg, 50%).

^{1}**H NMR** (500 MHz, DMSO-d_6) δ 11.00 (s, 1H), 7.58 (d, *J* = 7.8 Hz, 1H), 7.49 (d, *J* = 15.6 Hz, 1H), 7.31 (d, *J* = 1.7 Hz, 1H), 7.21 (dd, *J* = 8.3, 1.7 Hz, 1H), 7.17 (t, *J* = 8.2 Hz, 1H), 6.99 (d, *J* = 8.4 Hz, 1H), 6.69 (d, *J* = 15.6 Hz, 1H), 6.56 (d, *J* = 8.2 Hz, 1H), 3.82 (s, 3H), 3.79 (s, 3H). ^{13}**C NMR** (126 MHz, DMSO-d_6) δ 170.09, 163.74, 151.10, 150.46, 148.95, 140.77, 140.65, 132.79, 127.40, 122.25, 120.51, 111.99, 111.61, 110.24, 108.36, 101.80, 55.57, 55.56. **HRMS** $C_{18}H_{18}N_2O_5$; calcd. for (M-H$^+$): 341.1143, found 341.1140.

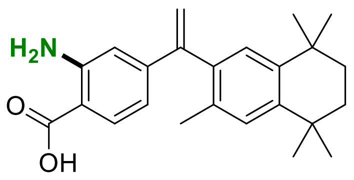

2-Amino-4-(1-(3,5,5,8,8-pentamethyl-5,6,7,8-tetrahydronaphthalen-2-yl)vinyl)benzoic acid (**3d**) was prepared according to General Procedure B. Reaction time in the C–H amination step increased to 44

h. The product was purified by HPLC (60-100% MeCN in acidic buffer). The product **3d** was isolated as a light brown solid (83.5 mg, 46%).

^{1}**H NMR** (500 MHz, CD_3OD) δ 7.86 – 7.82 (m, 1H), 7.12 – 7.08 (m, 2H), 6.82 – 6.78 (m, 1H), 6.71-6.67 (m, 1H), 5.84 – 5.81 (m, 1H), 5.22 – 5.19 (m, 1H), 1.98 – 1.95 (m, 3H), 1.72 – 1.70 (m, 4H), 1.30 – 1.26 (m, 12H). ^{13}**C NMR** (126 MHz, CD_3OD) δ 170.8, 151.0, 149.1, 147.8, 145.4, 143.3, 139.7, 134.0, 133.0, 129.1, 129.0, 117.52, 117.47, 116.9, 113.2, 36.32, 36.30, 34.89, 34.78, 32.33, 32.27, 20.01. **HRMS** $C_{24}H_{29}NO_2$; calcd. for (M-H$^+$): 362.2125, found 362.2124.

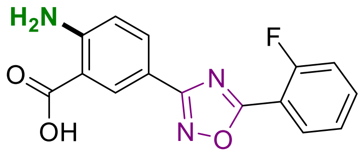

2-Amino-5-(5-(2-fluorophenyl)-1,2,4-oxadiazol-3-yl)benzoic acid (**3e**) was prepared according to General Procedure B. The product was purified by HPLC (38-78% MeCN in acidic buffer). The product **3e** was isolated as a beige solid (19.6 mg, 17%).

^{1}**H NMR** (500 MHz, DMSO-d_6) 8.48 (d, J = 1.7 Hz, 1H), 8.22 (t, J = 7.0 Hz, 1H), 7.87 – 7.94 (m, 1H), 7.78 (q, J = 6.5 Hz, 1H), 7.52 – 7.58 (m, 1H), 7.48 (t, J = 7.6 Hz, 1H), 6.92 (d, J = 8.7 Hz, 1H). ^{13}**C NMR** (126 MHz, DMSO-d_6) δ 171.9 (d, J_{C-F} = 4.1 Hz, 1C), 169.0, 167.7, 159.9 (d, J_{C-F} = 258.3 Hz, 1C), 153.8, 135.5 (d, J_{C-F} = 8.8 Hz, 1C), 131.9, 131.2, 130.9, 125.5 (d, J_{C-F} = 3.5 Hz, 1C), 117.3 (d, J_{C-F} = 20.2 Hz, 1C), 117.1, 112.0 (d, J_{C-F} = 11.3 Hz, 1C), 111.8, 109.5. ^{19}**F NMR** (471 MHz, DMSO-d_6) δ -109.68. **HRMS** $C_{15}H_{10}FN_3O_3$; calcd. for (M-H$^+$): 298.0633, found 298.0636.

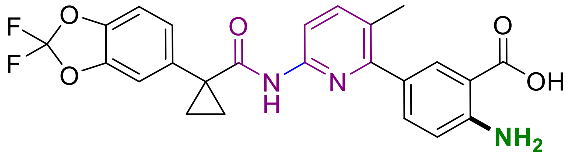

2-Amino-5-(6-(1-(2,2-difluorobenzo[d][1,3]dioxol-5-yl)cyclopropane-1-carboxamido)-3-methylpyridin-2-yl)benzoic acid (**3f**) was prepared according to General Procedure B. The product was purified by HPLC (28-68% MeCN in basic buffer). The isolated material was acidified (1M HCl, 10 mL) and extracted with EtOAc (3x10 mL). The combined extracts were dried with MgSO4 and concentrated. The product **3f** was isolated as a yellow solid (28.1 mg, 12%).

^{1}**H NMR** (500 MHz, DMSO-d_6) δ 9.76 (s, 1H), 7.99 (s, 2H), 7.89 (d, J = 1.8 Hz, 1H), 7.55 (s, 1H), 7.45 (dd, J = 8.6, 1.9 Hz, 1H), 7.39 (d, J = 8.3 Hz, 1H), 7.33 (d, J = 8.3 Hz, 1H), 6.86 (d, J = 8.6 Hz, 1H), 2.28 (s, 3H), 1.54 – 1.64 (m, 2H), 1.23 – 1.27 (m, 2H). ^{13}**C NMR** (126 MHz, DMSO-d_6) δ 172.0, 169.1, 152.0, 147.2, 142.9, 142.4, 135.3, 134.4, 133.3, 132.6, 131.3, 129.3, 127.1, 126.9, 116.3, 113.1, 112.6, 110.2, 109.2, 31.4, 18.6, 16.6. ^{19}**F NMR** (471 MHz, DMSO-d_6) δ -48.72. **HRMS** $C_{24}H_{19}F_2N_3O_5$; calcd. for (M-H$^+$): 466.1220, found 466.1218.

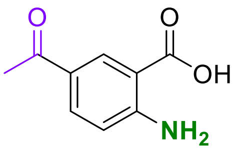

5-acetyl-2-aminobenzoic acid (**3g**) was prepared according to General Procedure B on a 9.0 mmol scale. Modification: The reaction mixture was cooled in an ice bath. The azide solution was slowly added over 5 minutes to the cooled reaction mixture and let to stir with cooling for further 5 minutes

before letting to hear up to room temperature. The product was purified by HPLC (20-60% MeCN in acidic buffer). The product **3g** was isolated as a colorless solid (1129 mg, 70%).

^{1}**H NMR** (500 MHz, MeOD) δ 8.54 (d, *J* = 2.2 Hz, 1H), 7.85 (dd, *J* = 8.8, 2.2 Hz, 1H), 6.76 (d, *J* = 8.8 Hz, 1H), 2.49 (s, 3H). ^{13}**C NMR** (126 MHz, MeOD) δ 198.5, 170.9, 157.0, 135.7, 134.6, 125.7, 117.2, 110.1, 25.9. **HRMS** $C_9H_9NO_3$; calcd. For (M-H$^+$): 178.0504, found 178.0504

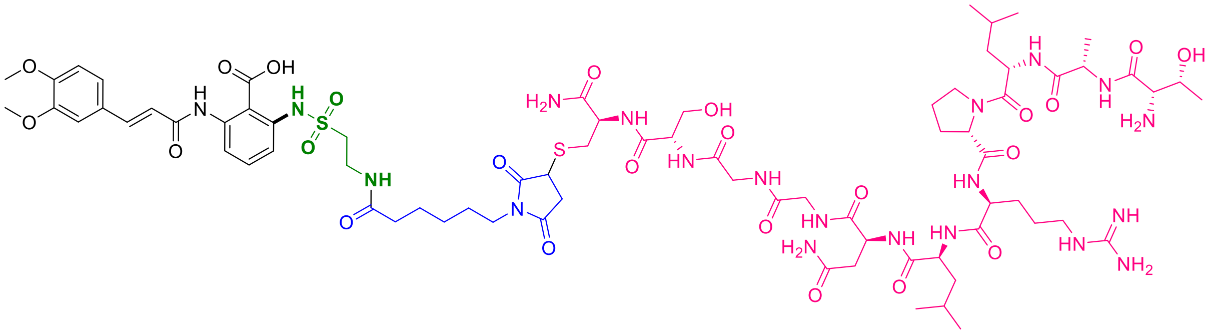

2-((2-(6-(3-(((6*S*,9*S*,12*S*,21*S*,24*R*)-6-((*S*)-1-(L-Threonyl-L-alanyl-L-leucyl)pyrrolidine-2-carboxamido)-1-amino-12-(2-amino-2-oxoethyl)-24-carbamoyl-21-(hydroxymethyl)-1-imino-9-isobutyl-7,10,13,16,19,22-hexaoxo-2,8,11,14,17,20,23-heptaazapentacosan-25-yl)thio)-2,5-dioxopyrrolidin-1-yl)hexanamido)ethyl)sulfonamido)-6-((*E*)-3-(3,4-dimethoxyphenyl)acrylamido)benzoic acid (**3ya**)

2,5-Dioxopyrrolidin-1-yl 6-(2,5-dioxo-2,5-dihydro-1H-pyrrol-1-yl)hexanoate (8.1 mg, 0.03 mmol) and (*E*)-2-((2-ammonioethyl)sulfonamido)-6-(3-(3,4-dimethoxyphenyl)acrylamido)benzoate (**2yc**, 12.6 mg, 0.03 mmol) were charged in a 4mL scitilation vial, followed by addition of DMF (0.3 mL) and N-ethyl-N-isopropylpropan-2-amine (18.31 µl, 0.11 mmol). The mixture was stirred at room temperature for 1 hour, upon which the peptide (*S*)-2-((*S*)-2-((*S*)-2-((*S*)-1-(L-threonyl-L-alanyl-L-leucyl)pyrrolidine-2-carboxamido)-5-guanidinopentanamido)-4-methylpentanamido)-*N*1-(2-((2-(((*S*)-1-(((*R*)-1-amino-1-oxo-3-(l6-sulfaneyl)propan-2-yl)amino)-3-hydroxy-1-oxopropan-2-yl)amino)-2-oxoethyl)amino)-2-oxoethyl)succinamide (24.0 mg, 0.022 mmol) was added. The RM was stirred for further 3 h.

The product was purified by HPLC*,* 10-60% B in 6 min, flow 8.3 ml/min. Collection on double charged ion.
A: 3% MeCN, 97% H20, 0.1 M FA
B: 95% MeCN, 5% H20, 0.1 M FA
Column Waters SunFire 5µm 10x100mm
System: Waters Autopurification LC-MS using MassLynx and FractionLynx.
Compound **3ya** (14.4 mg, 38%) was obtained as a colorless solid.

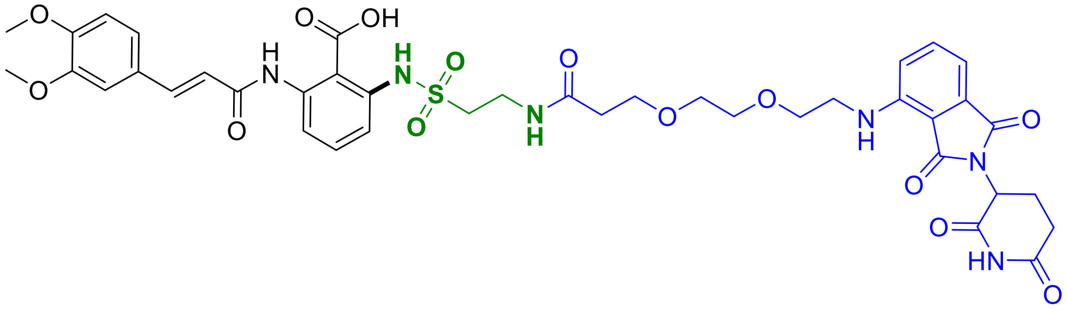

(*E*)-2-(3-(3,4-Dimethoxyphenyl)acrylamido)-6-((2-(3-(2-(2-((2-(2,6-dioxopiperidin-3-yl)-1,3-dioxoisoindolin-4-yl)amino)ethoxy)ethoxy)propanamido)ethyl)sulfonamido)benzoic acid (**3yb**).

3-(2-(2-((2-(2,6-Dioxopiperidin-3-yl)-1,3-dioxoisoindolin-4-yl)amino)ethoxy)ethoxy)propanoic acid (13.9 mg, 0.03 mmol), 1-hydroxypyrrolidine-2,5-dione (6.3 mg, 0.05 mmol) and 3-(((ethylimino)methylene)amino)-N,N-dimethylpropan-1-amine hydrochloride (8.9 mg, 0.05 mmol) were charged in a 1.5mL scintillation vial, followed by addition of DMF (0.2 mL) and triethylamine (9 µl, 0.06 mmol). The RM was stirred at rt for 16 h. After this LCMS (pH3) suggested high conversion to the desired NHS ester. (E)-2-((2-ammonioethyl)sulfonamido)-6-(3-(3,4-dimethoxyphenyl)acrylamido)benzoate (**2yc**, 14.4 mg, 0.03 mmol) was added in one portion, followed by the addition of DMSO (0.2 mL) and triethylamine (8.93 µl, 0.06 mmol). The reaction mixture was stirred at room temperature overnight.

The product was purified by HPLC*,* 0-50% B in 6 min, flow 8.3 ml/min.
A: 3% MeCN, 97% H20, 0.1 M FA
B: 95% MeCN, 5% H20, 0.1 M FA
Column Waters SunFire 5µm 10x100mm
System: Waters Autopurification LC-MS using MassLynx and FractionLynx.
Compound **3ab** (11.9 mg, 43%) was obtained as a yellow solid.

^{1}H NMR (600 MHz, DMSO-*d*$_6$) δ 8.37 (dd, *J* = 8.2, 1.0 Hz, 1H), 7.92 (t, *J* = 5.6 Hz, 1H), 7.56 (dd, *J* = 8.4, 7.2 Hz, 1H), 7.49 (d, *J* = 15.6 Hz, 1H), 7.30 (d, *J* = 1.8 Hz, 1H), 7.25 (t, *J* = 8.2 Hz, 1H), 7.19 (dd, *J* = 8.3, 1.8 Hz, 1H), 7.14 (dd, *J* = 8.2, 1.1 Hz, 1H), 7.12 (d, *J* = 8.6 Hz, 1H), 7.02 (d, *J* = 7.0 Hz, 1H), 6.98 (d, *J* = 8.4 Hz, 1H), 6.54 – 6.59 (m, 2H), 5.05 (dd, *J* = 12.9, 5.5 Hz, 1H), 3.83 (s, 3H), 3.79 (s, 3H), 3.57 (t, *J* = 5.5 Hz, 2H), 3.49 – 3.54 (m, 4H), 3.41 – 3.46 (m, 4H), 3.35 (dd, *J* = 8.9, 5.8 Hz, 2H), 3.12 – 3.16 (m, 2H), 2.88 (ddd, *J* = 17.1, 13.9, 5.4 Hz, 1H), 2.51 – 2.63 (m, 2H), 2.22 (t, *J* = 6.5 Hz, 2H), 2.02 (dq, *J* = 10.4, 3.1 Hz, 1H). **^{13}C NMR** (151 MHz, DMSO-*d*$_6$) δ 172.8, 171.4, 170.3, 170.1, 168.9, 167.3, 163.8, 150.4, 149.0, 146.4, 142.6, 142.3, 140.1, 136.2, 132.1, 130.4, 127.4, 122.1, 121.2, 117.4, 113.2, 111.6, 111.1, 110.6, 110.2, 110.1, 109.2, 69.6, 69.5, 68.8, 66.6, 55.6, 55.5, 49.5, 48.5, 41.7, 36.0, 33.5, 30.7, 22.1. **HRMS** $C_{40}H_{44}N_6O_{14}S$; calcd. for (M-H$^+$): 863.2563, found 863.2555.

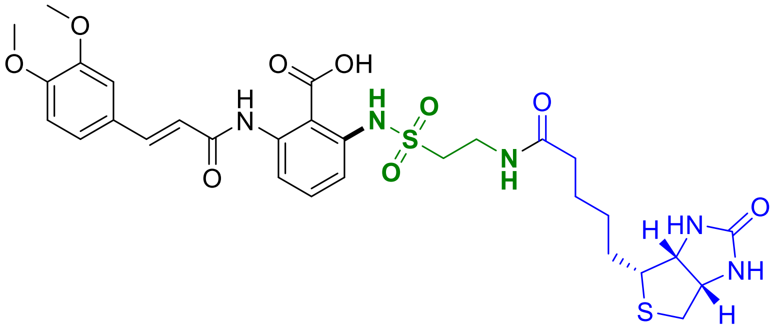

2-((*E*)-3-(3,4-Dimethoxyphenyl)acrylamido)-6-((2-(6-((3aR,4R,6aS)-2-oxohexahydro-1H-thieno[3,4-d]imidazol-4-yl)hexanamido)ethyl)sulfonamido)benzoic acid (**3yc**).

(E)-2-((2-Ammonioethyl)sulfonamido)-6-(3-(3,4-dimethoxyphenyl)acrylamido)benzoate (**2yc**, 24.0 mg, 0.05 mmol) and 2,5-dioxopyrrolidin-1-yl 5-((3aS,4S,6aR)-2-oxohexahydro-1H-thieno[3,4-d]imidazol-4-yl)pentanoate were charged in a 1.5mL scintillation vial, followed by addition of DMF (0.3 mL) and N,N-diisopropylethylamine (46.5 µl, 0.27 mmol). The reaction mixture was stirred at room temperature overnight.

The product was purified by HPLC*,* 0-50% B in 6 min, flow 8.3 ml/min .

A: 3% MeCN, 97% H20, 0.2% NH3
B: 95% MeCN, 5% H20, 0.2% NH3
Column Waters XBridge 5µm 10x100mm
System: Waters Autopurification LC-MS using MassLynx and FractionLynx.
Compound **3yc** (22.7 mg, 63%) was obtained as a colorless solid.

^{1}H NMR (600 MHz, DMSO-d_6) δ 8.38 (dd, *J* = 8.3, 0.9 Hz, 1H), 7.84 (t, *J* = 5.7 Hz, 1H), 7.50 (d, *J* = 15.6 Hz, 1H), 7.31 (d, *J* = 1.7 Hz, 1H), 7.27 (t, *J* = 8.2 Hz, 2H), 7.21 (dd, *J* = 8.3, 1.7 Hz, 2H), 7.15 (dd, *J* = 8.2, 1.0 Hz, 1H), 6.99 (d, *J* = 8.4 Hz, 1H), 6.58 (d, *J* = 15.6 Hz, 1H), 6.40 (s, 1H), 6.33 (s, 1H), 4.23 – 4.26 (m, 1H), 4.07 – 4.1 (m, 1H), 3.84 (s, 3H), 3.80 (s, 3H), 3.34 (p, *J* = 7.4, 6.8 Hz, 3H), 3.17 (t, *J* = 7.1 Hz, 2H), 3.02 – 3.06 (m, 1H), 2.76 (dd, *J* = 12.4, 5.1 Hz, 1H), 2.54 (d, *J* = 12.4 Hz, 1H), 1.96 (tt, *J* = 9.2, 4.9 Hz, 2H), 1.53 – 1.59 (m, 1H), 1.40 (dq, *J* = 13.6, 7.1, 5.9 Hz, 3H), 1.23 (tt, *J* = 15.1, 8.1 Hz, 2H). **^{13}C NMR** (151 MHz, DMSO-d_6) δ 172.2, 171.5, 163.8, 162.7, 150.4, 149.0, 142.6, 142.2, 140.2, 130.5, 127.5, 122.2, 121.2, 113.3, 111.6, 111.1, 110.2, 110.0, 61.0, 59.2, 55.61, 55.56, 55.4, 49.5, 35.1, 33.5, 28.2, 28.0, 25.0, 22.5. **HRMS** $C_{30}H_{37}N_5O_9S_2$; calcd. for (M-H$^+$): 674.1960, found 674.1957.

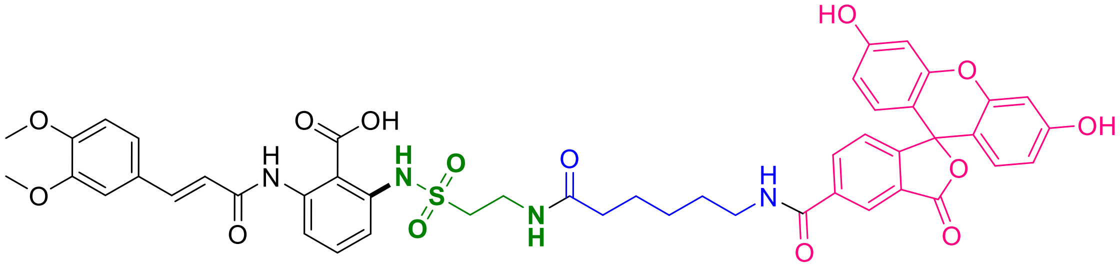

(*E*)-2-((2-(6-(3',6'-Dihydroxy-3-oxo-3H-spiro[isobenzofuran-1,9'-xanthene]-5-carboxamido)hexanamido)ethyl)sulfonamido)-6-(3-(3,4-dimethoxyphenyl)acrylamido)benzoic acid (**3yd**).

Step 1: Synthesis of **(*E*)-2-((2-(6-((*tert*-Butoxycarbonyl)amino)hexanamido)ethyl)sulfonamido)-6-(3-(3,4-dimethoxyphenyl)acrylamido)benzoic acid** (**3yd´**)

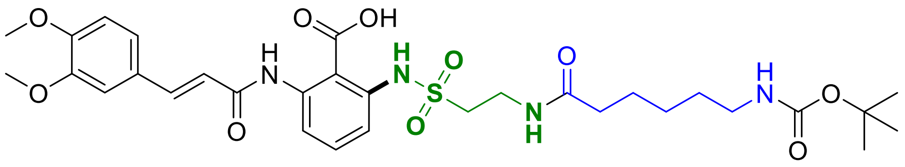

(E)-2-((2-Ammonioethyl)sulfonamido)-6-(3-(3,4-dimethoxyphenyl)acrylamido)benzoate (**2yc**, 31.1 mg, 0.07 mmol) and 2,5-dioxopyrrolidin-1-yl 6-((tert-butoxycarbonyl)amino)hexanoate (25.0 mg, 0.08 mmol) were charged in a 1.5mL scintillation vial, followed by addition of DMF (0.3 mL), DMSO (0.2 mL) and N,N-diisopropylethylamine (48.2 µl, 0.28 mmol). The reaction mixture was stirred at room temperature overnight. The product was purified by HPLC (23-63% MeCN in basic buffer) to yield compound impure **3yd´**. The material was taken for the next step without further purification.

Step 2: **Synthesis of (*E*)-2-((2-(6-Ammoniohexanamido)ethyl)sulfonamido)-6-(3-(3,4-dimethoxyphenyl)acrylamido)benzoate** (**3yd´´**)

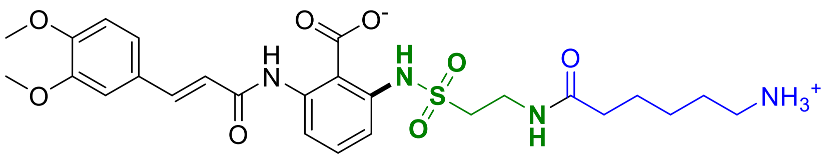

The material obtained in the previous step was dissolved in a mixture of DCM:TFA (1:1, 1 mL). The volatiles were removed in vacuo to yield an orange residue. The material was partitioned between EtOAc (10 mL) and aqueous HCl (1M, 10 mL). The aqueous phase was extracted with EtOAc (4x10 mL) and the combined organic phases dried with MgSO4. The material was used in the next step without further purification.

Step 3:

To the previously obtained material, now transfered into a 1.5 mL scintillation vial, 5-(((2,5-dioxopyrrolidin-1-yl)oxy)carbonyl)-2-(6-hydroxy-3-oxo-3H-xanthen-9-yl)benzoic acid (49.3 mg, 0.1 mmol) was added, followed by addition of DMF (0.8 mL), DMSO (0.2 mL) and N,N-diisopropylethylamine (63.9 µl, 0.37 mmol). The reaction mixture was stirred at room temperature overnight. The product was purified by HPLC (30-70% MeCN in acidic buffer). The product **3yd** was isolated as a yellow solid (25.4 mg, 40%).

^{1}H NMR (600 MHz, DMSO-*d₆*) δ 11.17 (s, 2H), 10.14 (s, 2H), 8.78 (t, *J* = 5.5 Hz, 1H), 8.44 (s, 1H), 8.23 (dd, *J* = 8.0, 1.5 Hz, 1H), 7.96 (t, *J* = 5.7 Hz, 1H), 7.87 (d, *J* = 8.2 Hz, 1H), 7.51 (d, *J* = 15.6 Hz, 1H), 7.44 (t, *J* = 8.2 Hz, 1H), 7.35 (d, *J* = 8.0 Hz, 1H), 7.28 (d, *J* = 1.8 Hz, 1H), 7.23 (dd, *J* = 8.2, 0.8 Hz, 1H), 7.19 (dd, *J* = 8.3, 1.8 Hz, 1H), 6.99 (d, *J* = 8.4 Hz, 1H), 6.72 (d, *J* = 15.6 Hz, 1H), 6.68 (d, *J* = 2.3 Hz, 2H), 6.58 (d, *J* = 8.7 Hz, 2H), 6.54 (dd, *J* = 8.7, 2.3 Hz, 2H), 3.82 (s, 3H), 3.79 (s, 3H), 3.39 – 3.43 (m, 3H), 3.25 – 3.32 (m, 5H), 2.03 (t, *J* = 7.5 Hz, 2H), 1.50 (dp, *J* = 22.9, 7.4 Hz, 4H), 1.27 (p, *J* = 7.7 Hz, 2H). **^{13}C NMR** (151 MHz, DMSO-*d₆*) δ 172.4, 168.9, 168.2, 164.5, 164.0, 159.6, 154.6, 151.8, 150.5, 149.0, 140.9, 139.4, 138.4, 136.4, 134.7, 131.6, 129.2, 127.4, 126.5, 124.2, 123.2, 122.2, 120.0, 118.0, 115.8, 114.8, 112.7, 111.7, 110.2, 109.1, 102.3, 55.6, 55.5, 50.6, 40.4, 35.2, 33.5, 28.7, 26.1, 24.8. **HRMS** $C_{47}H_{44}N_4O_{14}S$; calcd. for $(M-H^+)$: 919.2502, found 919.2512.

NMR spectra and HPLC chromatograms

2-Nitrobenzenesulfonyl azide (**NsN_3**), 1M in EtOAc

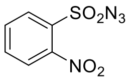

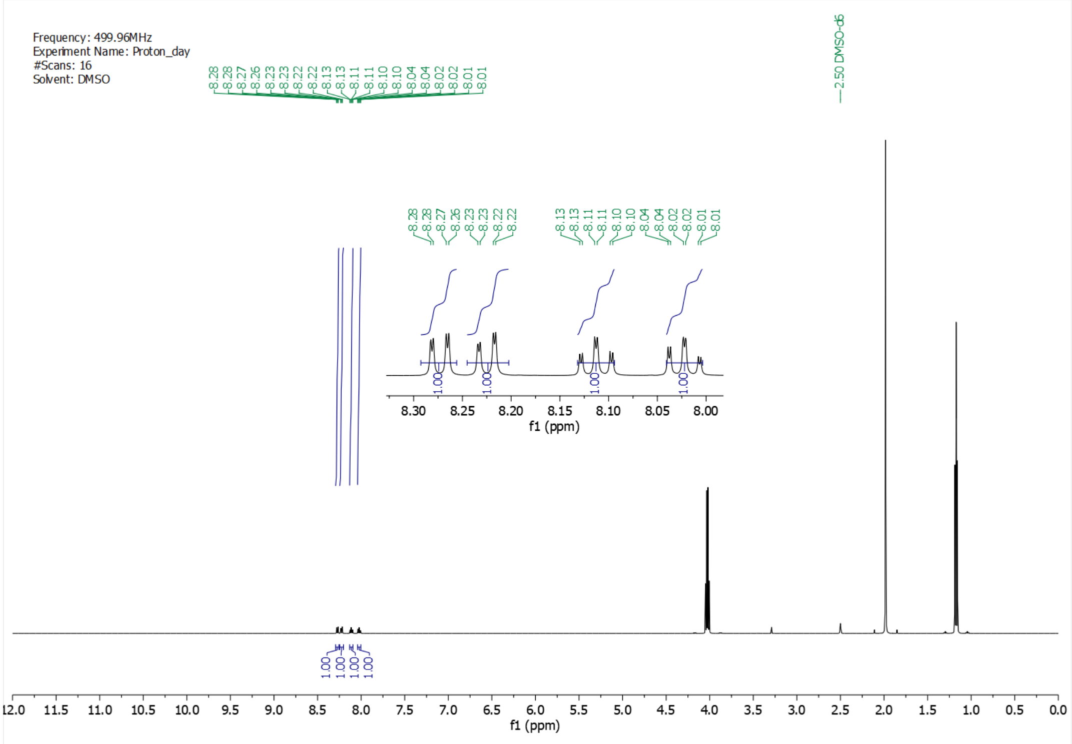

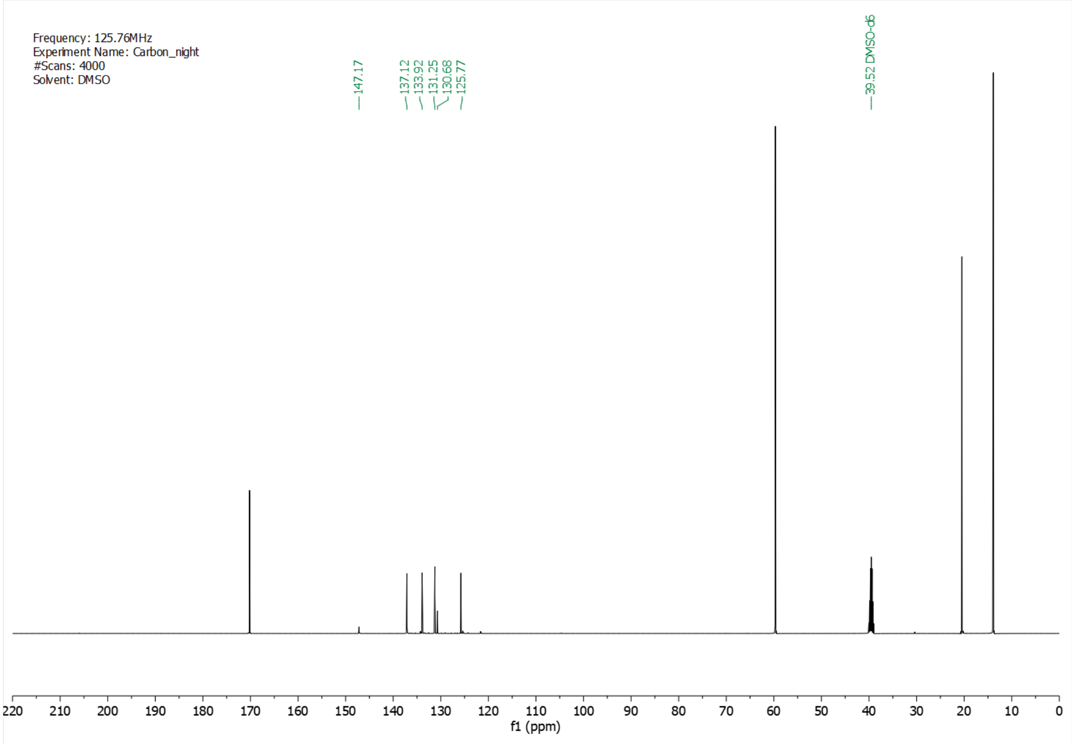

Note: Carboxylic acid C not observed (low signal intensity)

Benzyl (2-(azidosulfonyl)ethyl)carbamate (4a), 1M in EtOAc

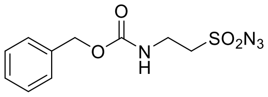

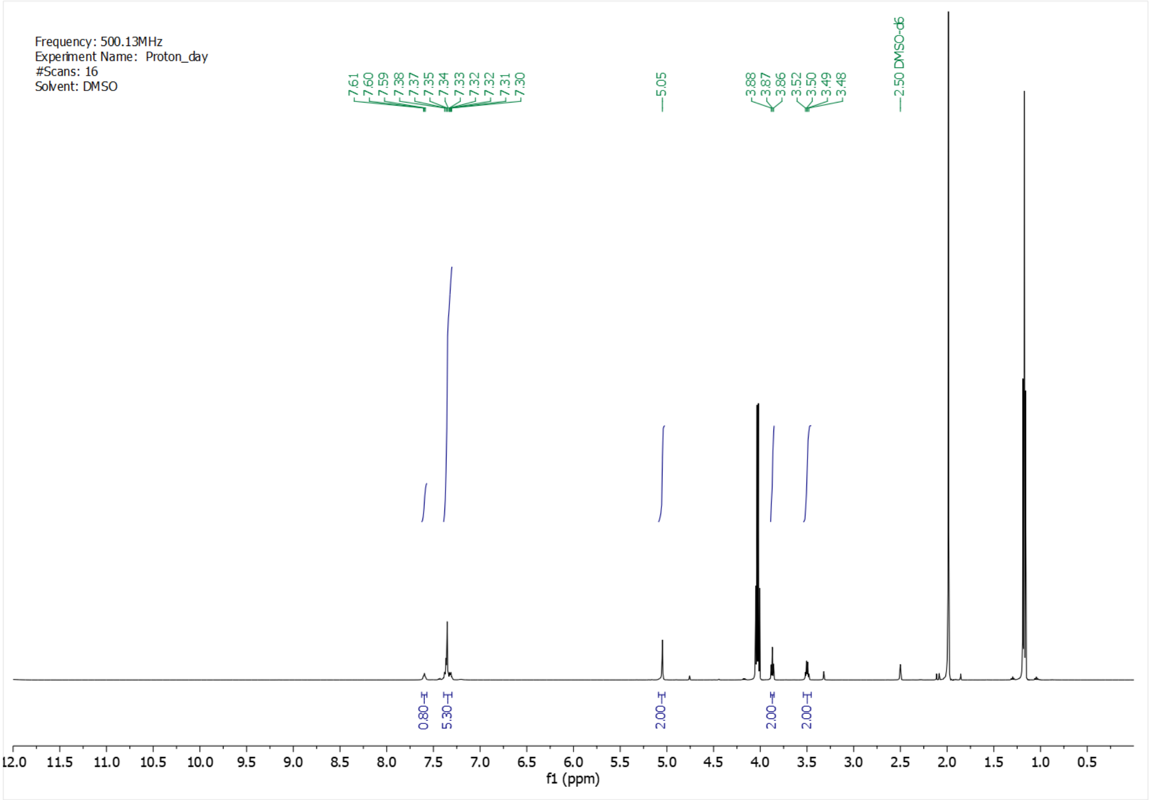

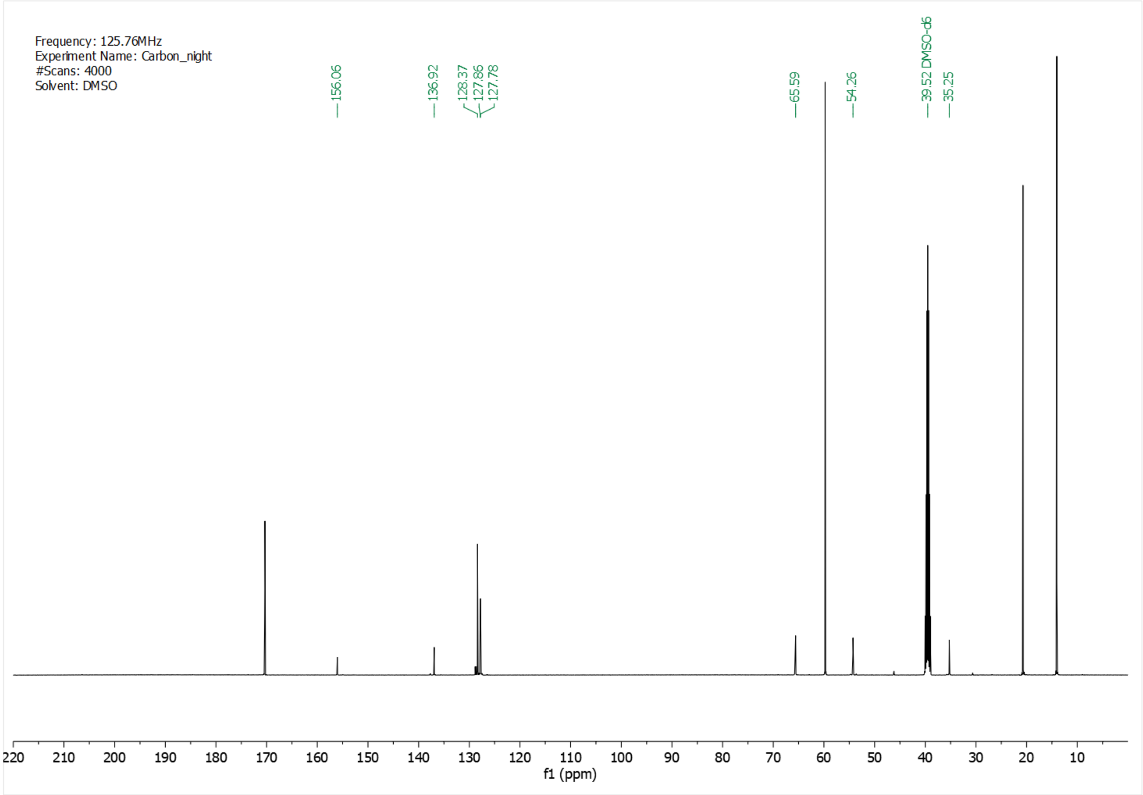

(*E*)-2-((2-Ammonioethyl)sulfonamido)-6-(3-(3,4-dimethoxyphenyl)acrylamido)benzoate (2yc)

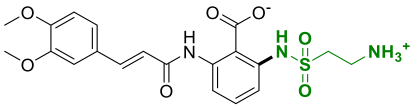

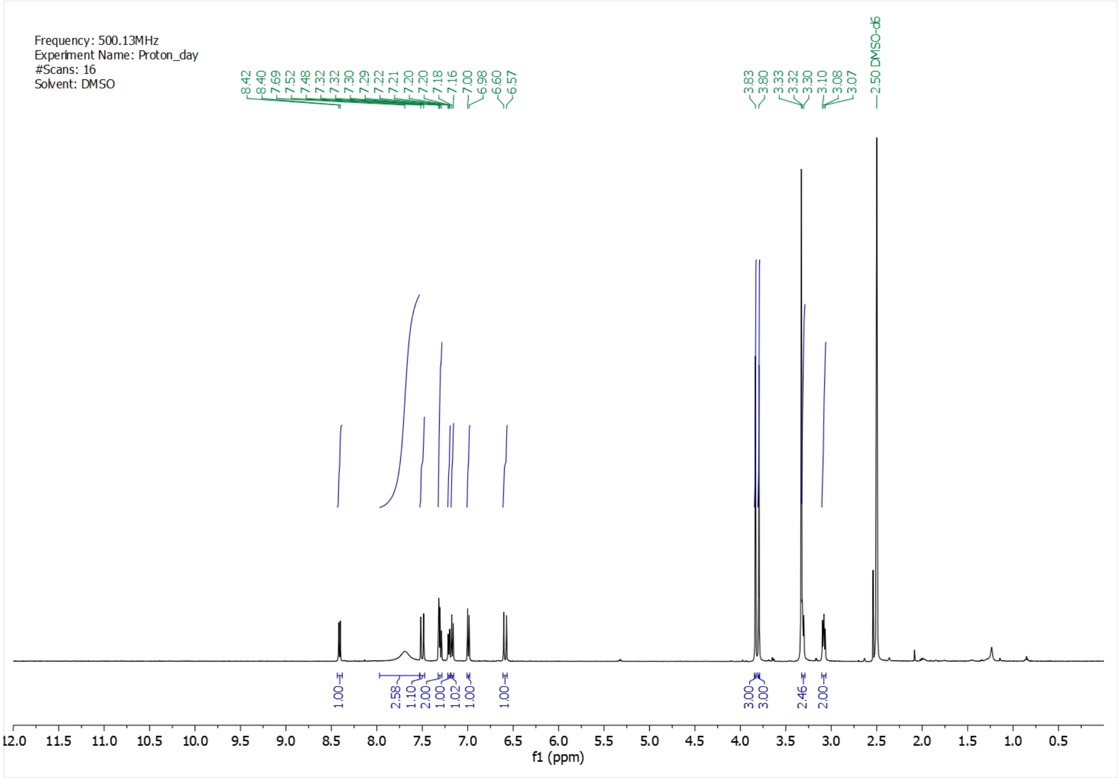

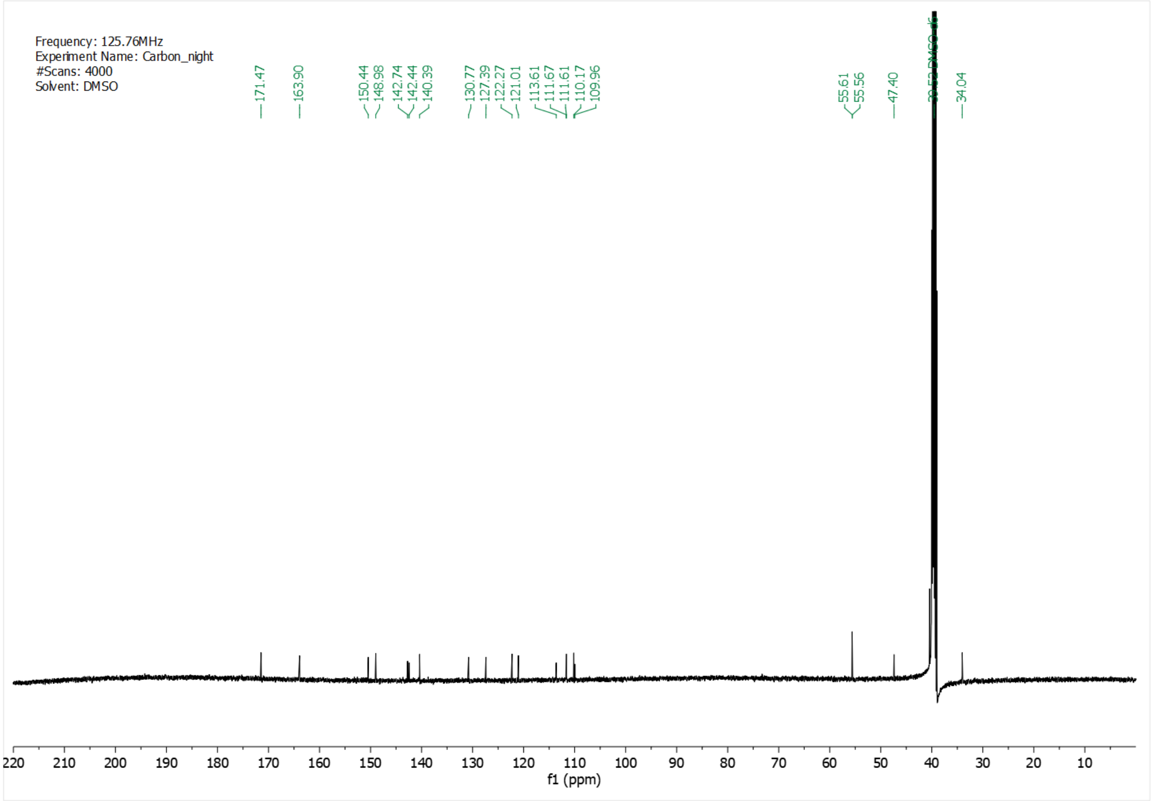

2-Fluoro-6-((4-methylphenyl)sulfonamido)benzoic acid (2a)

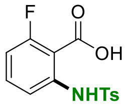

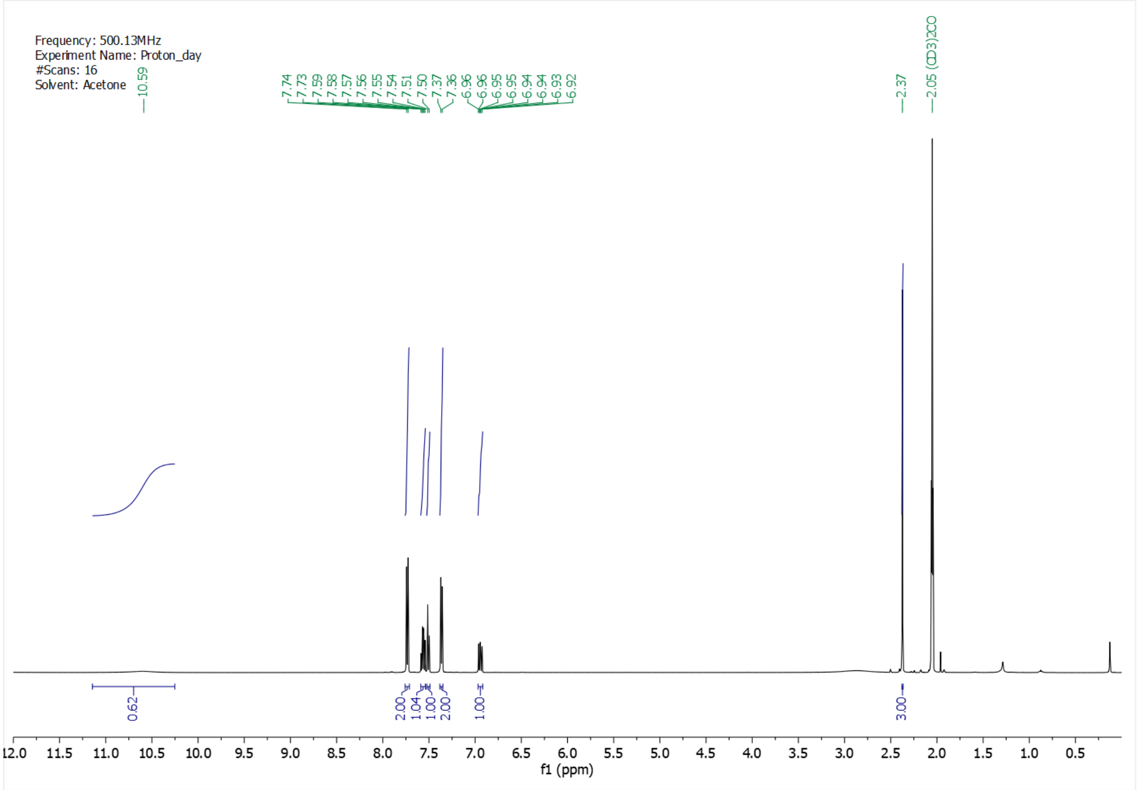

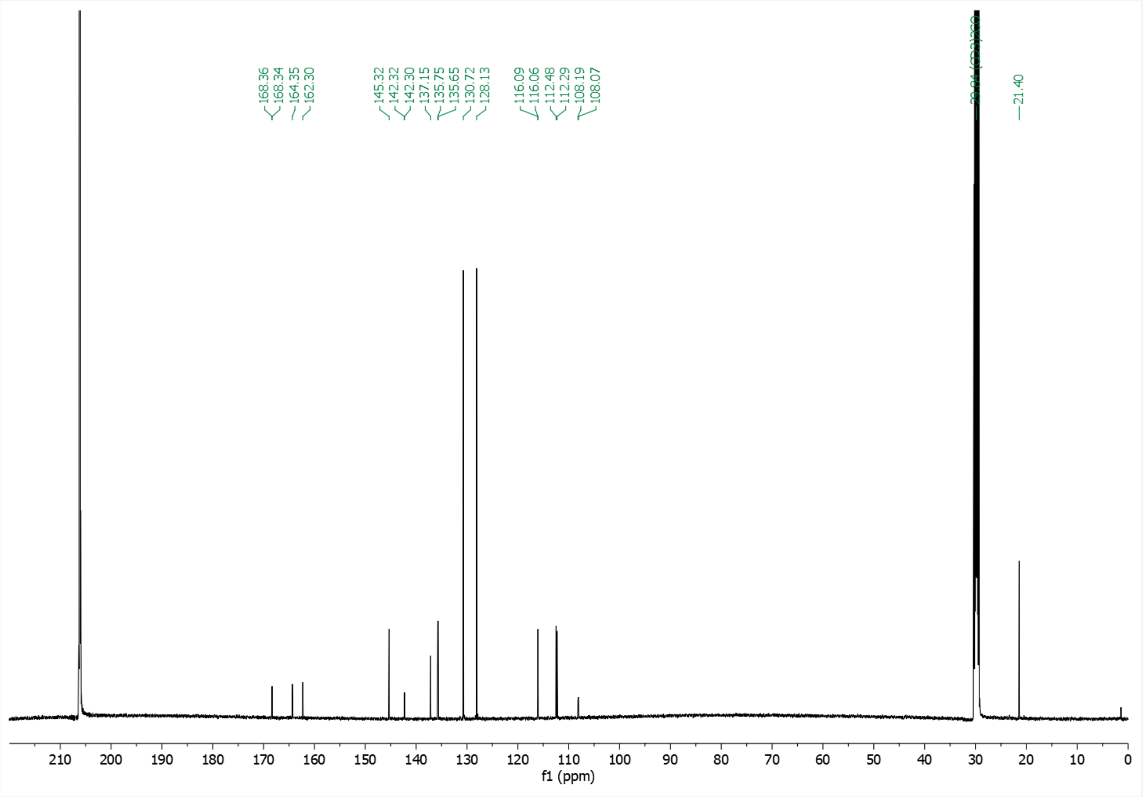

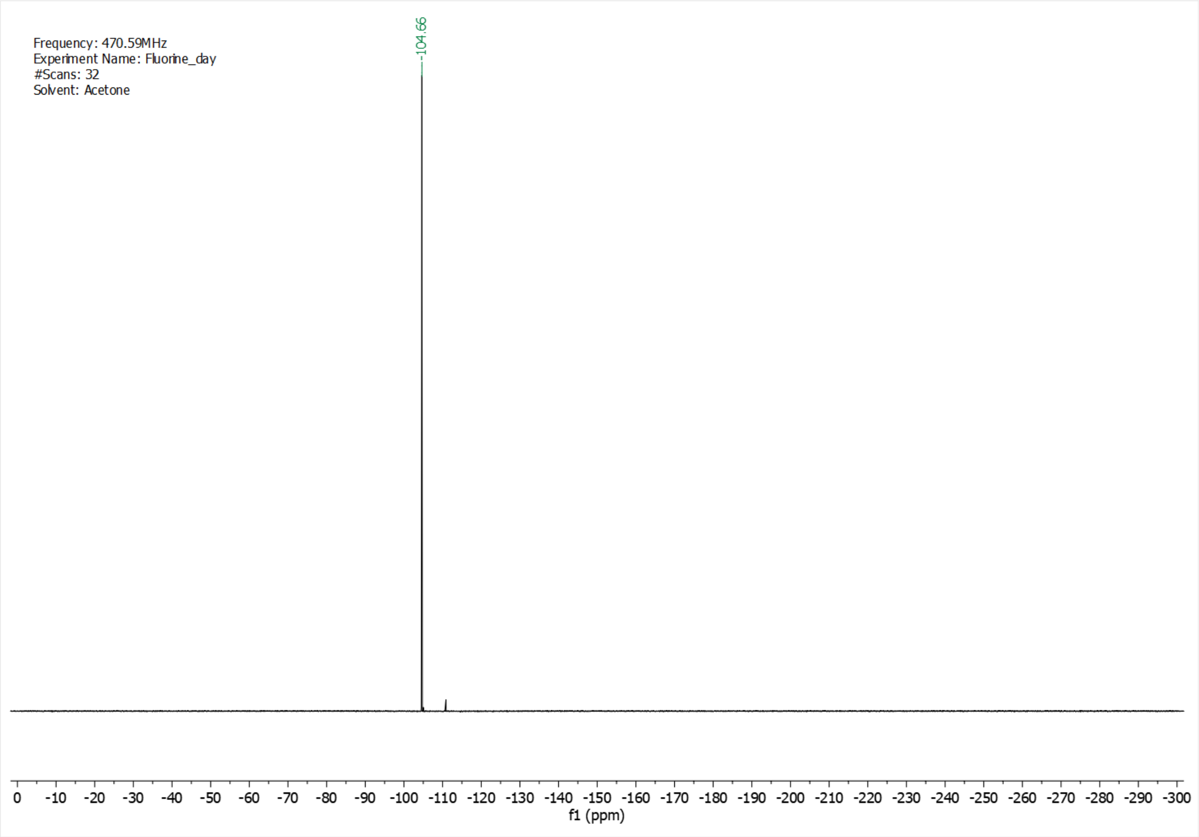
Frequency: 470.59MHz
Experiment Name: Fluorine_day
#Scans: 32
Solvent: Acetone
-104.66
0 -10 -20 -30 -40 -50 -60 -70 -80 -90 -100 -110 -120 -130 -140 -150 -160 -170 -180 -190 -200 -210 -220 -230 -240 -250 -260 -270 -280 -290 -300
f1 (ppm)

2-Methoxy-6-((4-methylphenyl)sulfonamido)benzoic acid (2b)

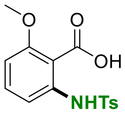

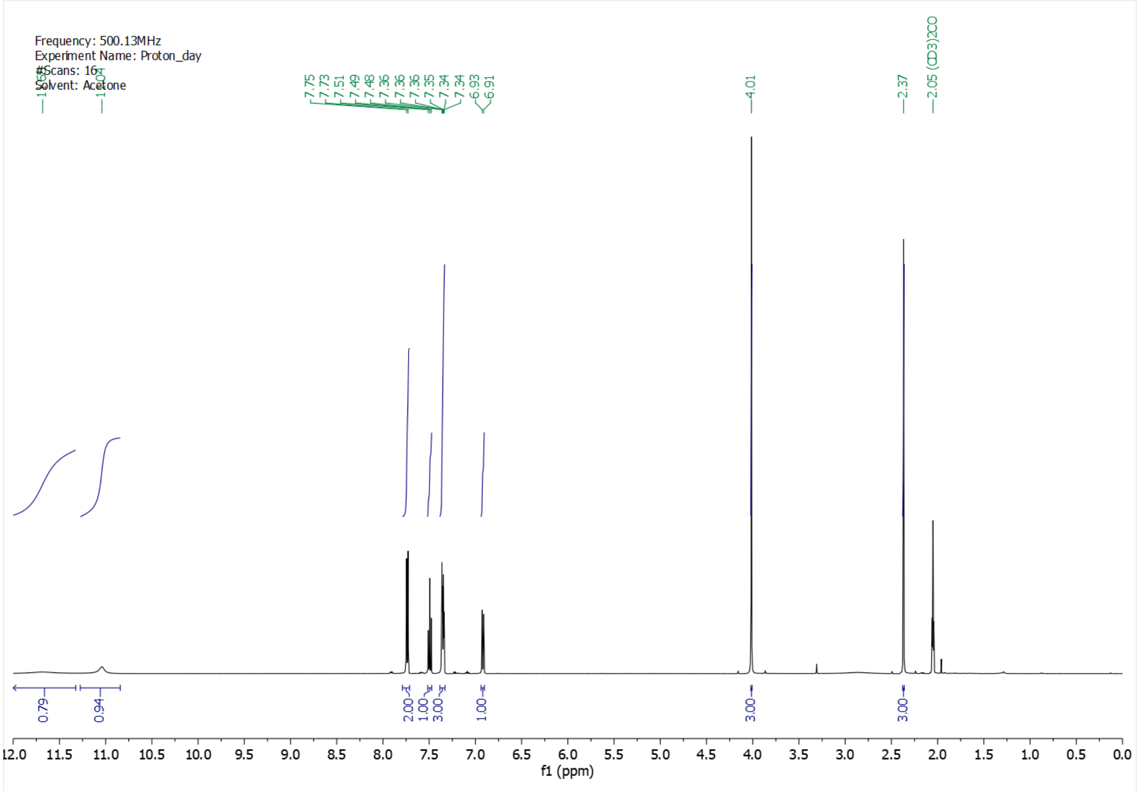

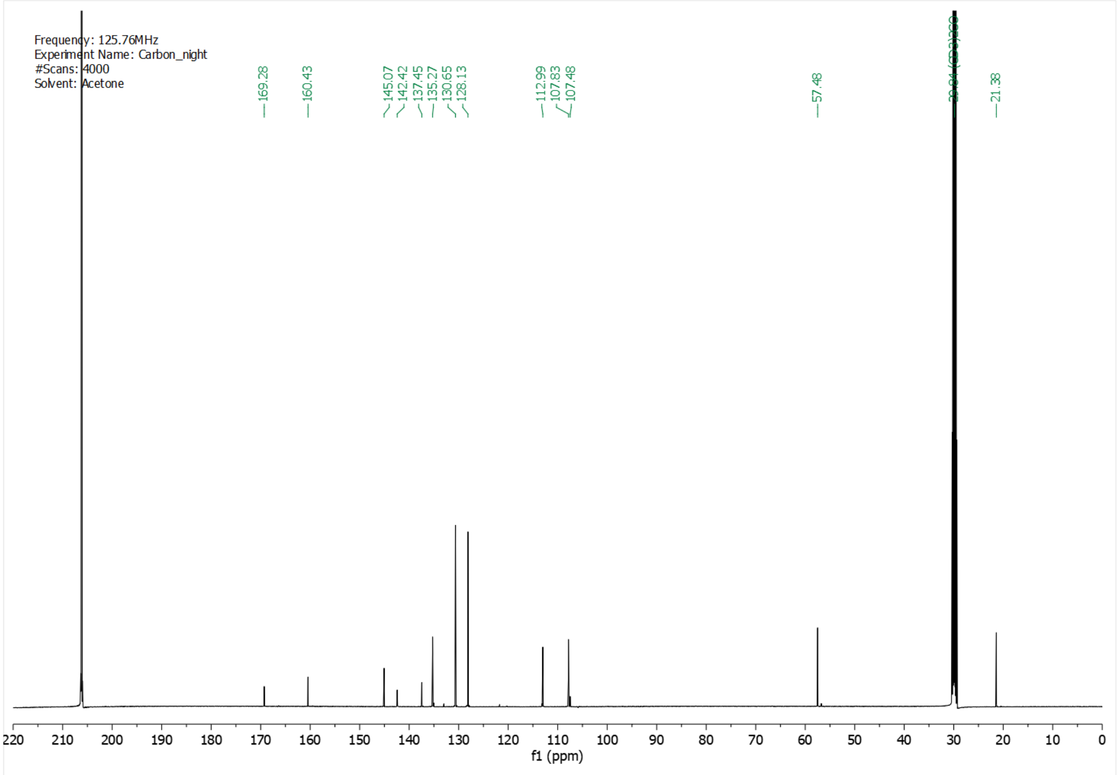

2-Methyl-6-((4-methylphenyl)sulfonamido)benzoic acid (2c)

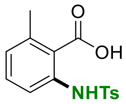

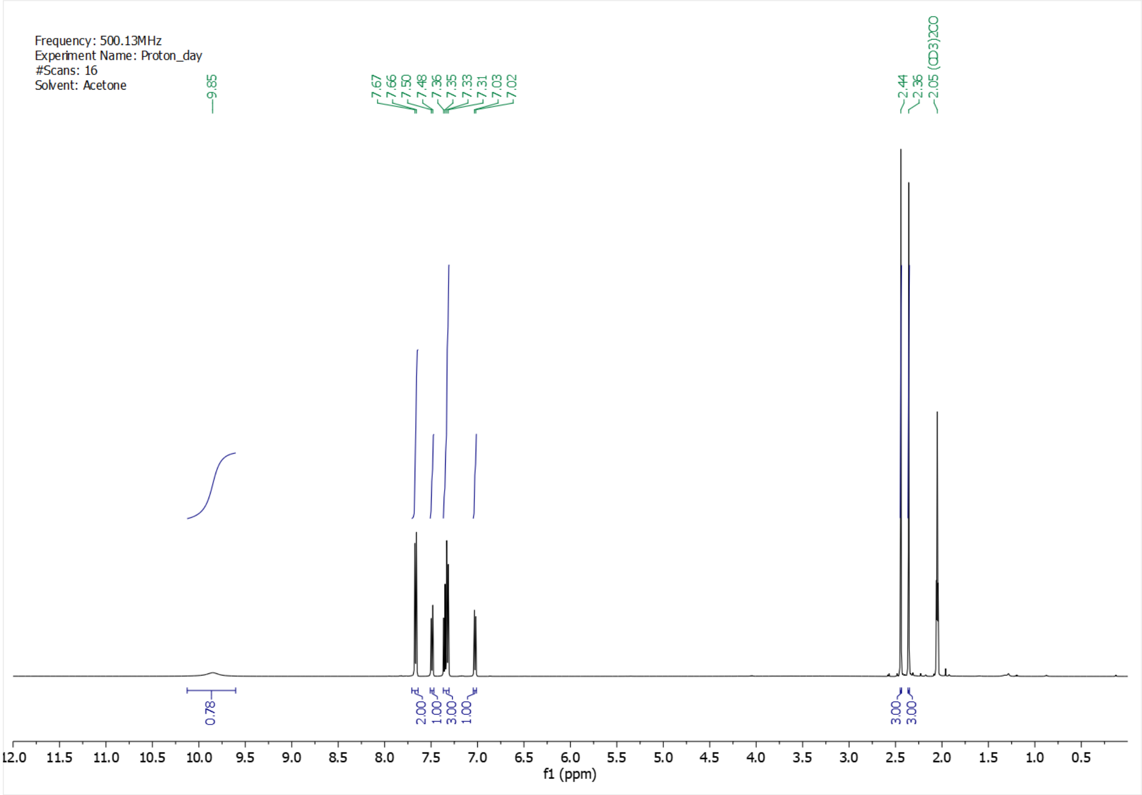

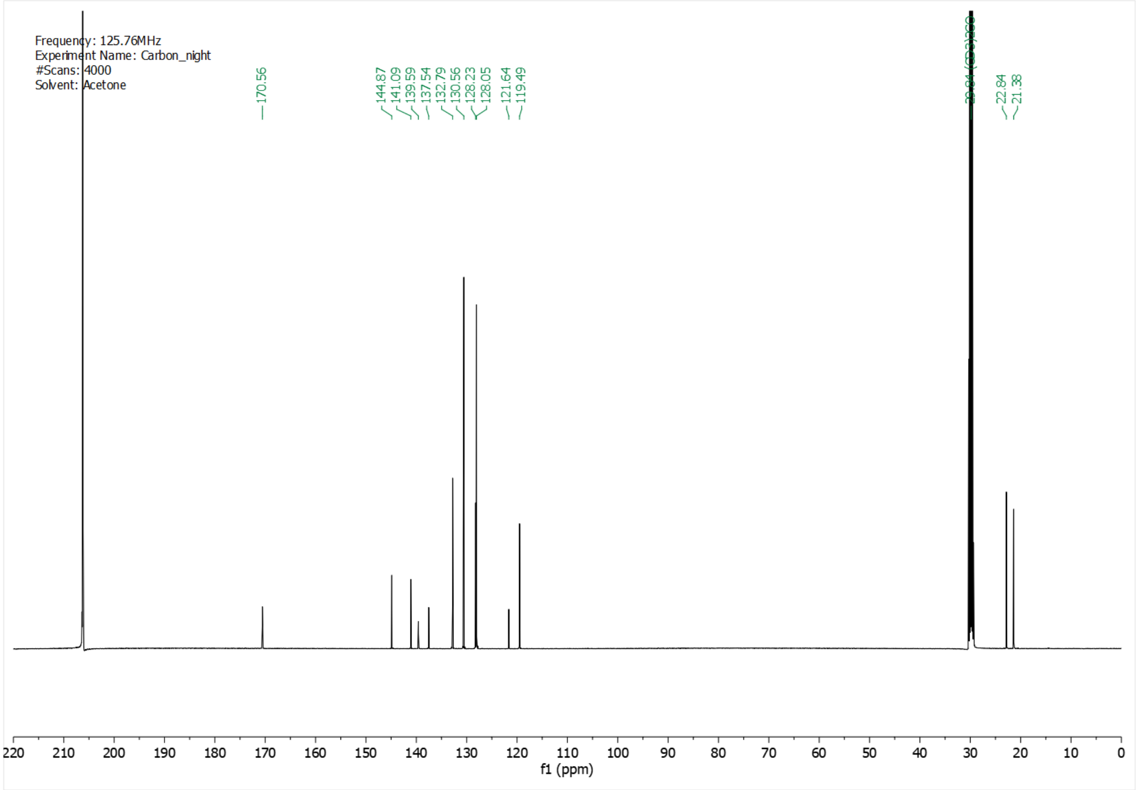

3-((4-Methylphenyl)sulfonamido)-[1,1'-biphenyl]-2-carboxylic acid (2d)

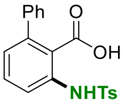

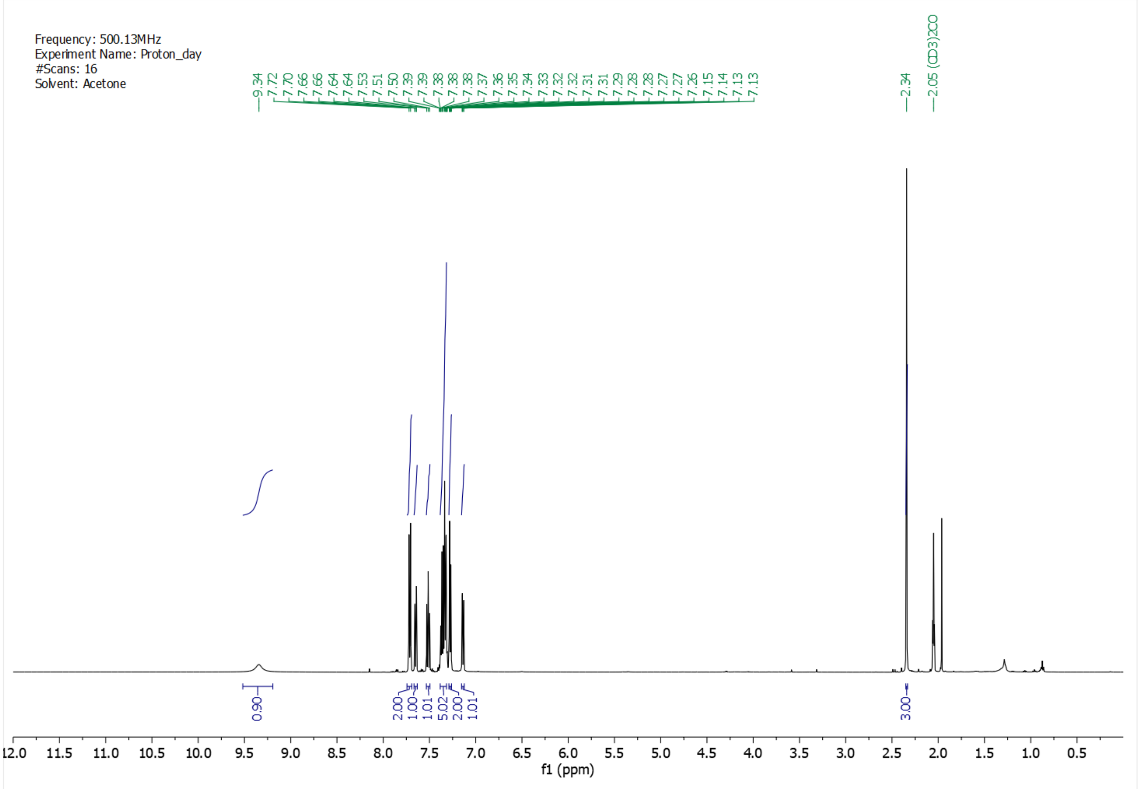

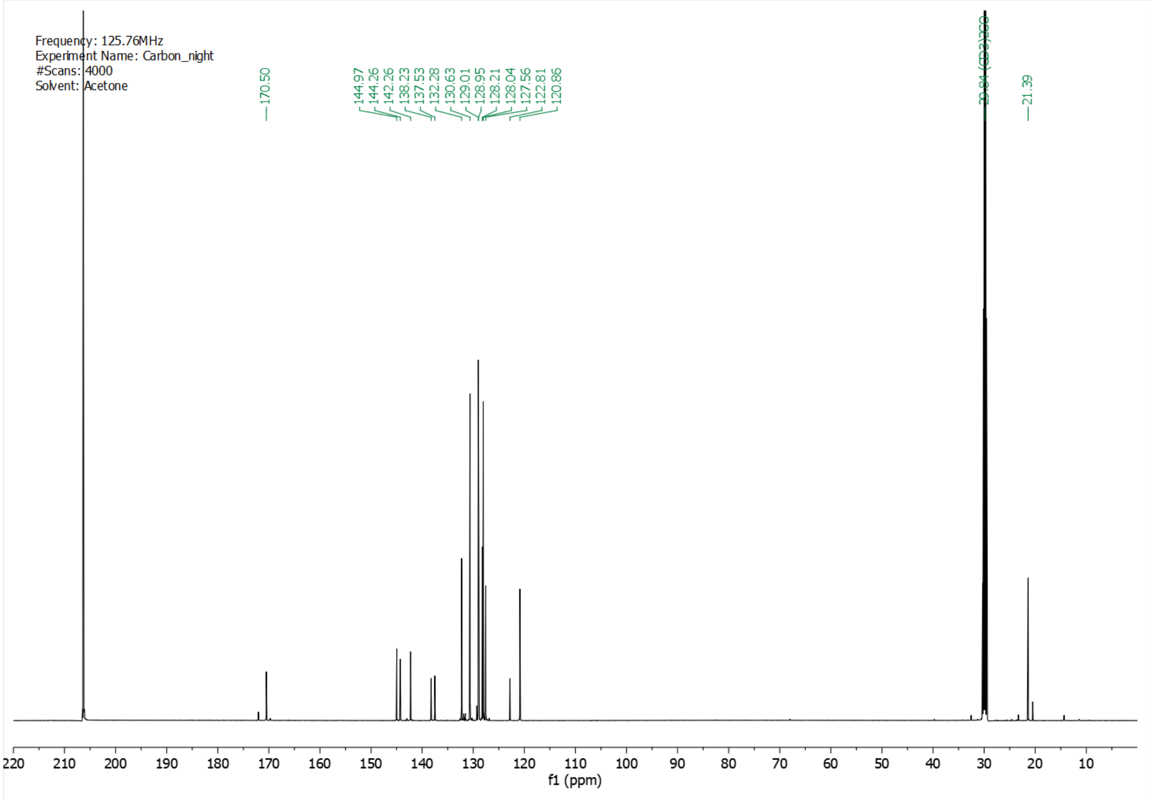

5-Methyl-2-((4-methylphenyl)sulfonamido)benzoic acid (**2e**)

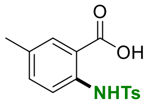

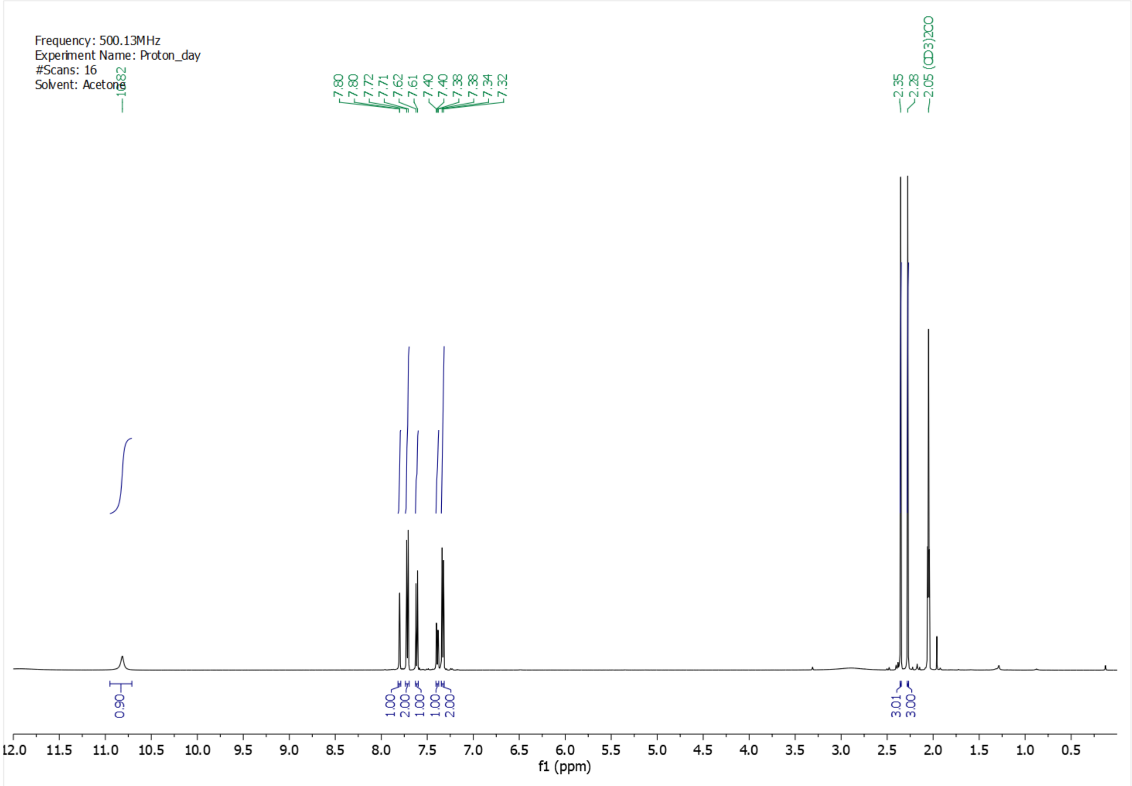

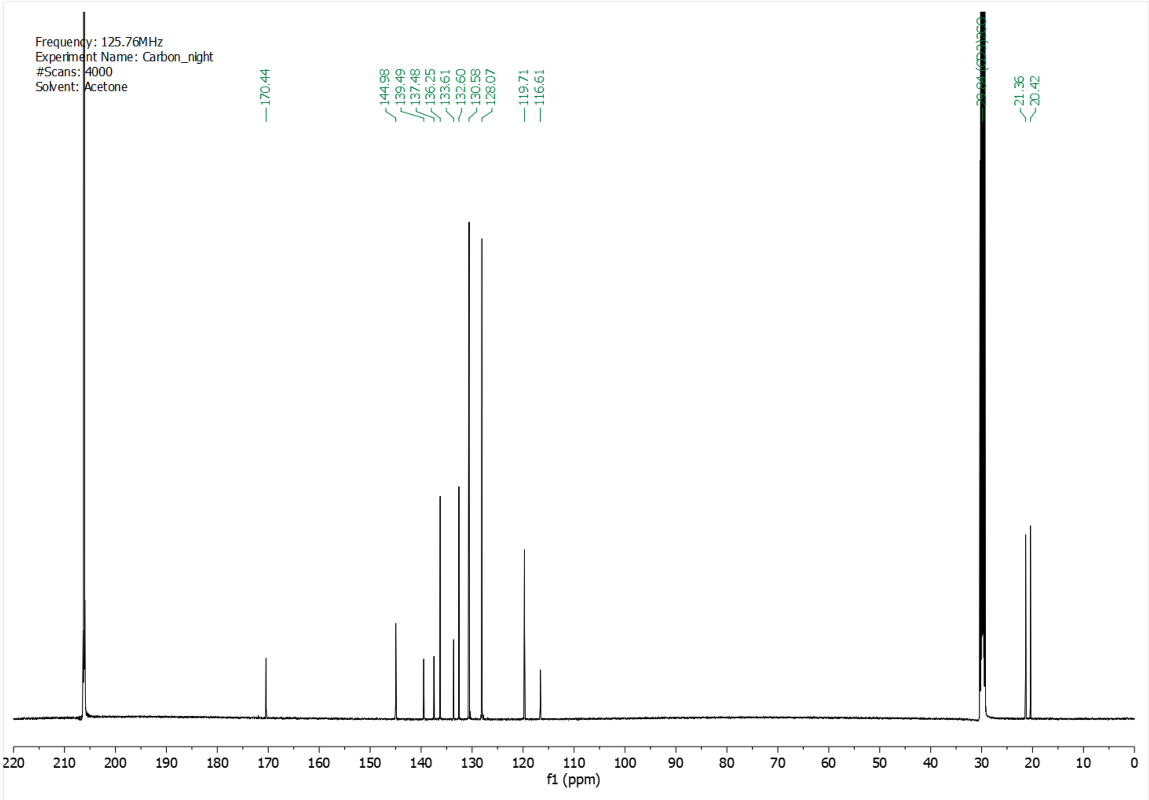

2-((4-Methylphenyl)sulfonamido)-5-(trifluoromethyl)benzoic acid (2f)

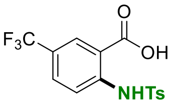

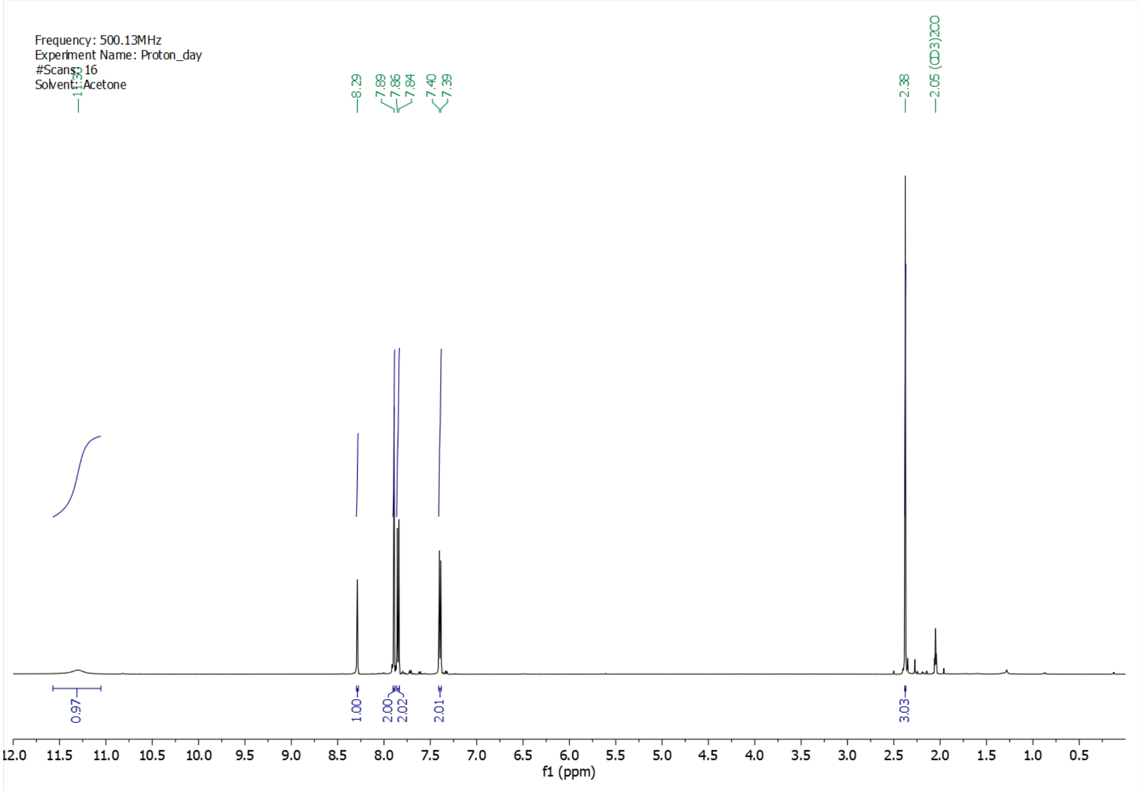

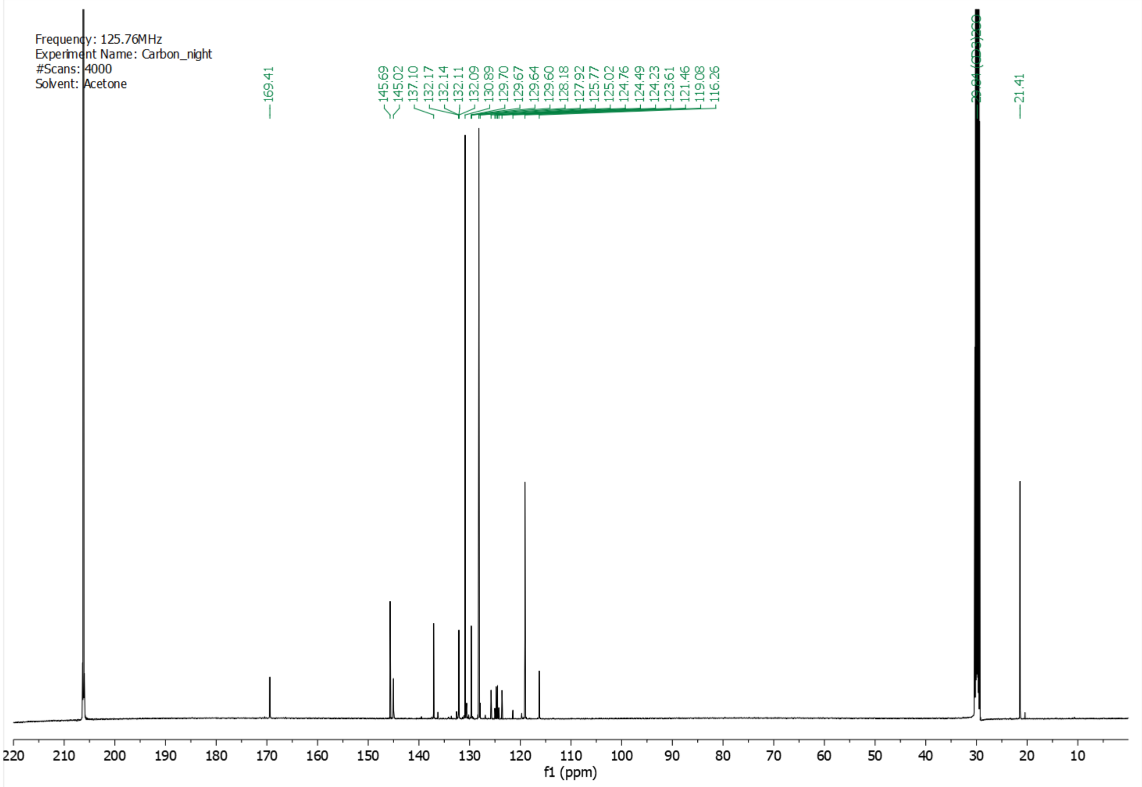

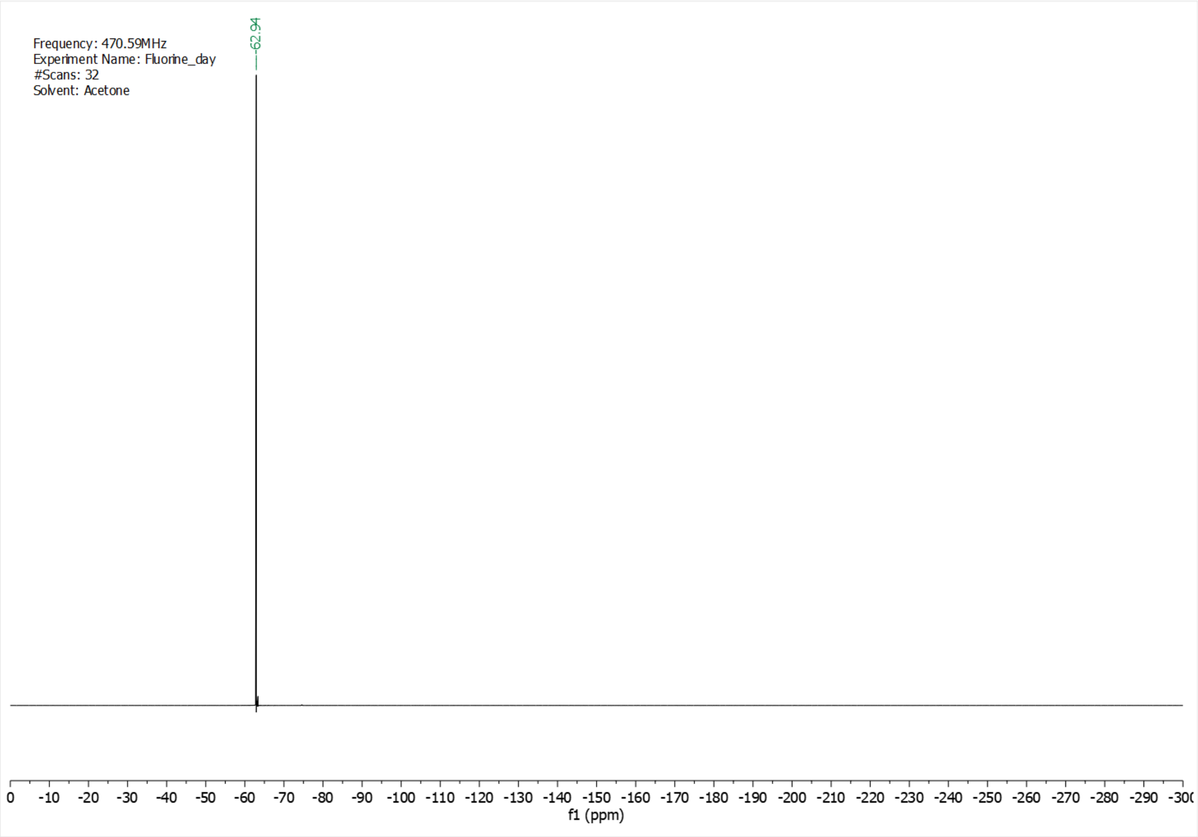

Frequency: 470.59MHz
Experiment Name: Fluorine_day
#Scans: 32
Solvent: Acetone
-62.94
0 -10 -20 -30 -40 -50 -60 -70 -80 -90 -100 -110 -120 -130 -140 -150 -160 -170 -180 -190 -200 -210 -220 -230 -240 -250 -260 -270 -280 -290 -300
f1 (ppm)

5-(Methoxycarbonyl)-2-((4-methylphenyl)sulfonamido)benzoic acid (2g)

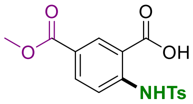

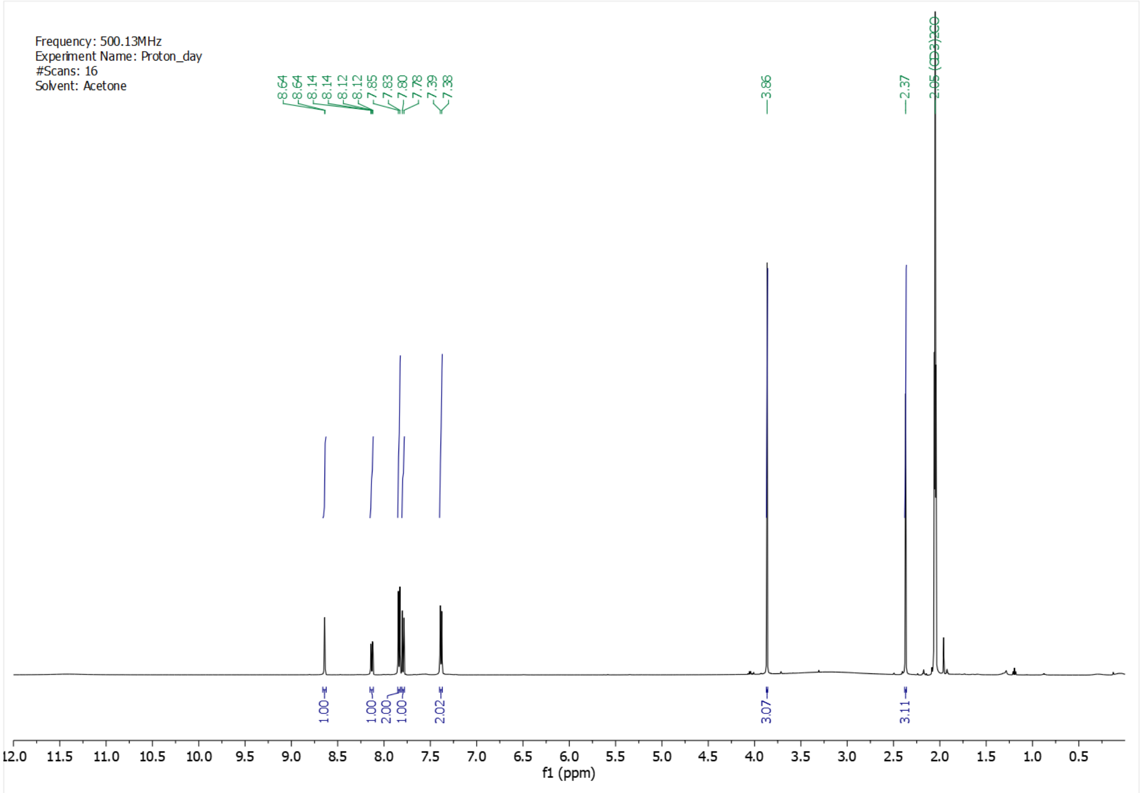

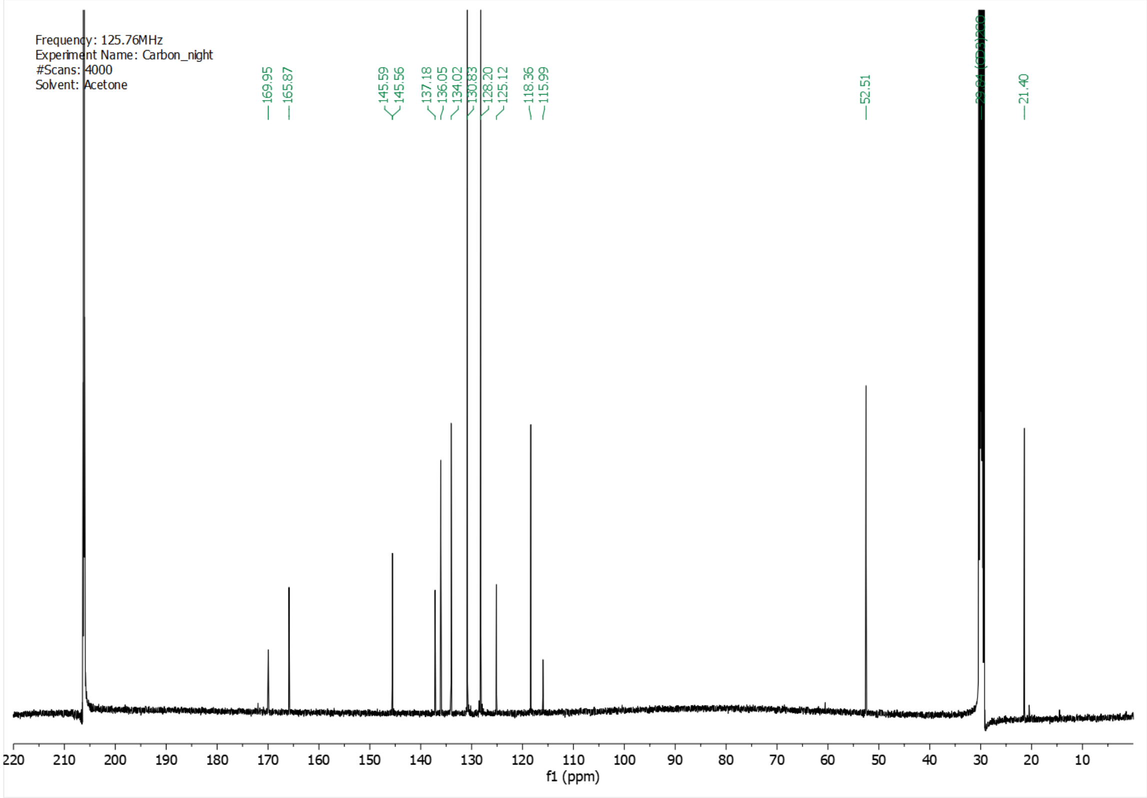

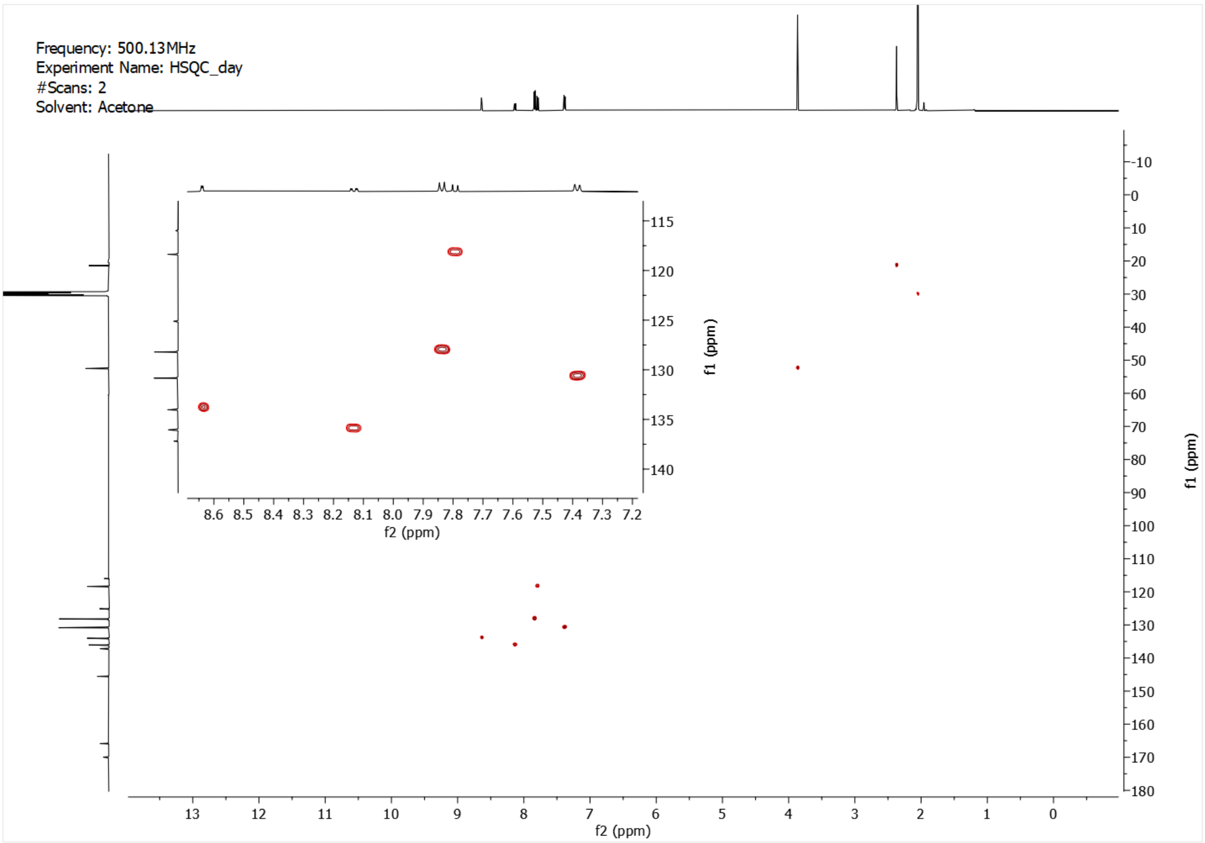
Frequency: 500.13MHz
Experiment Name: HSQC_day
#Scans: 2
Solvent: Acetone
f1 (ppm)
f2 (ppm)

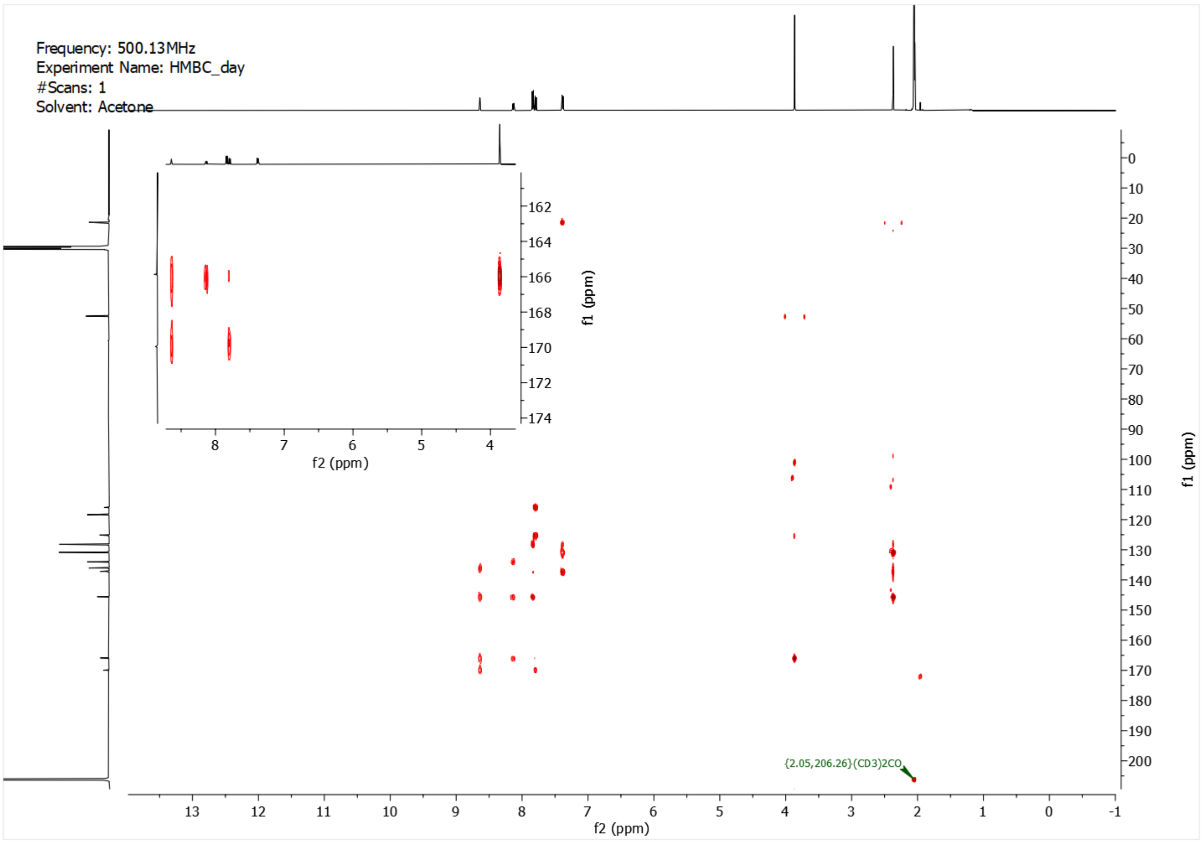
Frequency: 500.13MHz
Experiment Name: HMBC_day
#Scans: 1
Solvent: Acetone
{2.05,206.26}(CD3)2CO
f1 (ppm)
f2 (ppm)

5-Acetyl-2-((4-methylphenyl)sulfonamido)benzoic acid (2h)

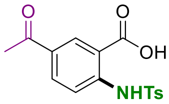

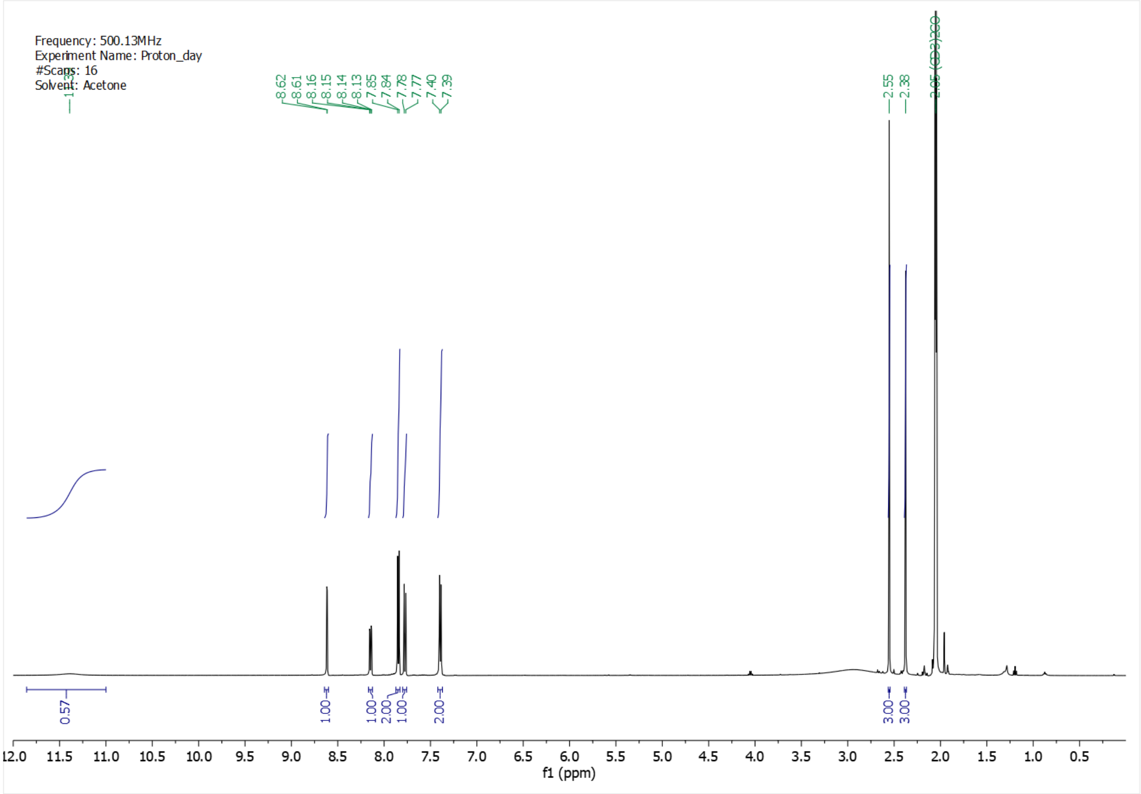

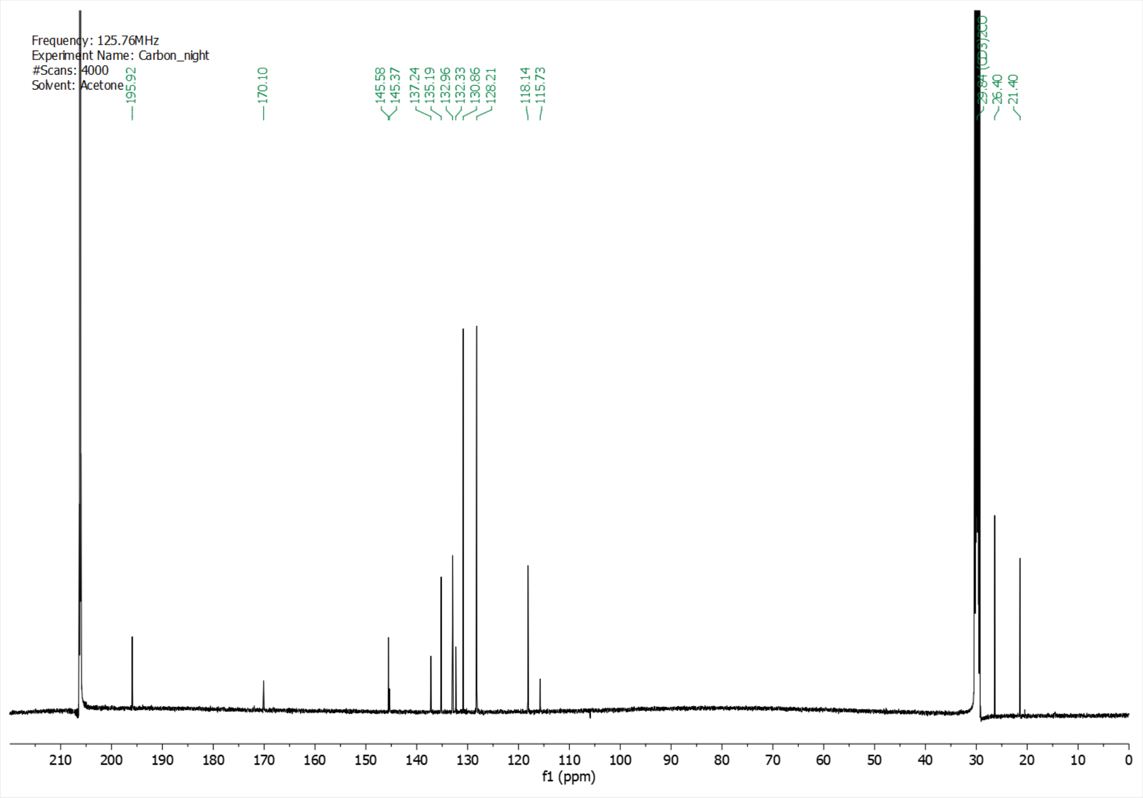

5-Carbamoyl-2-((4-methylphenyl)sulfonamido)benzoic acid (2i)

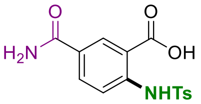

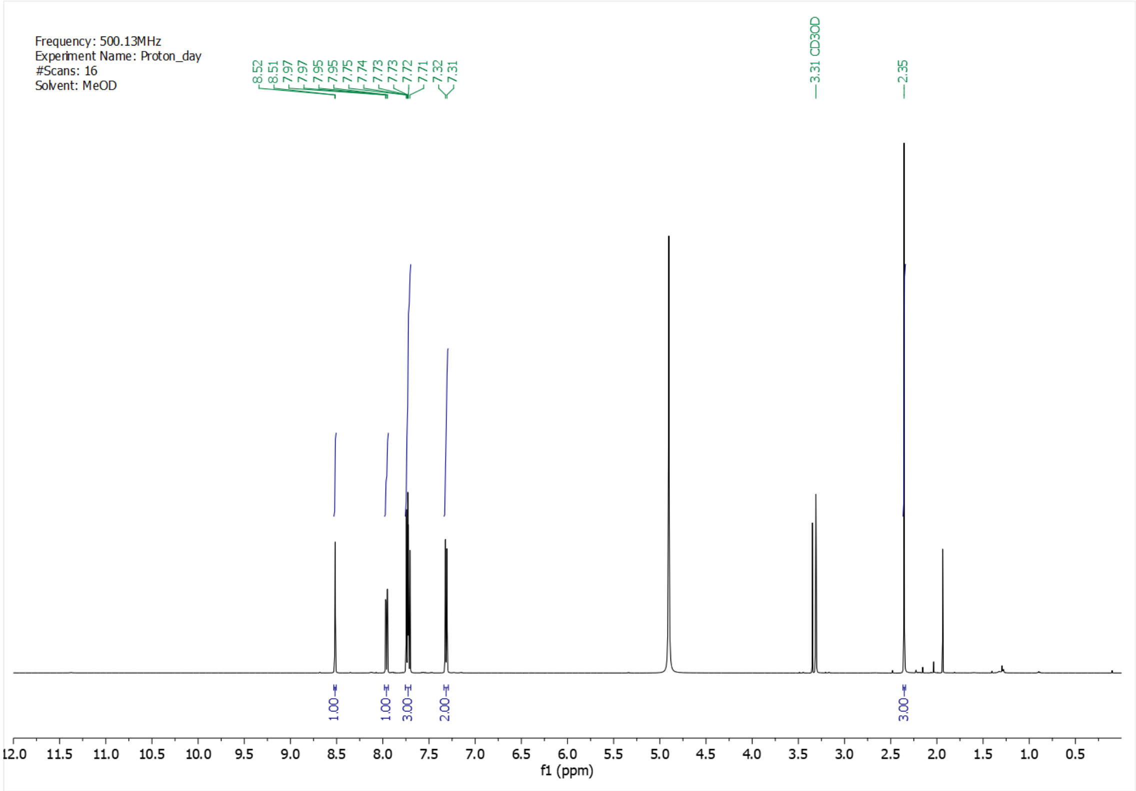

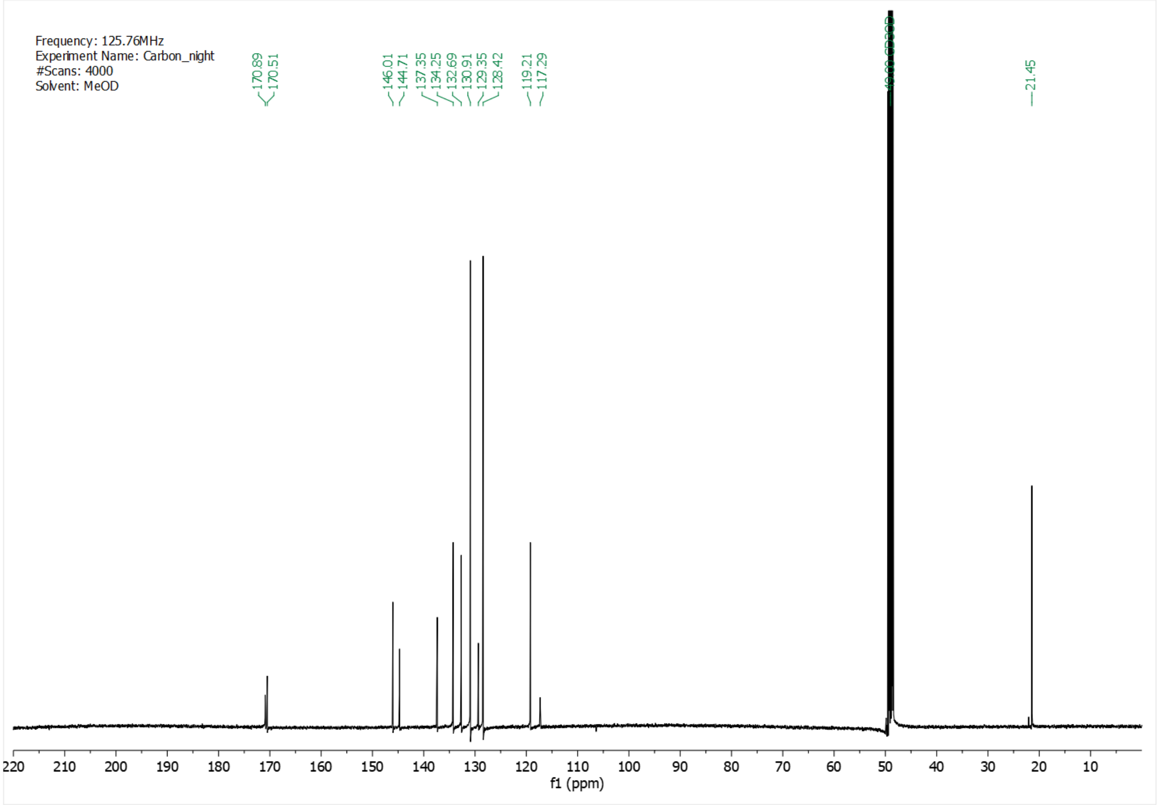

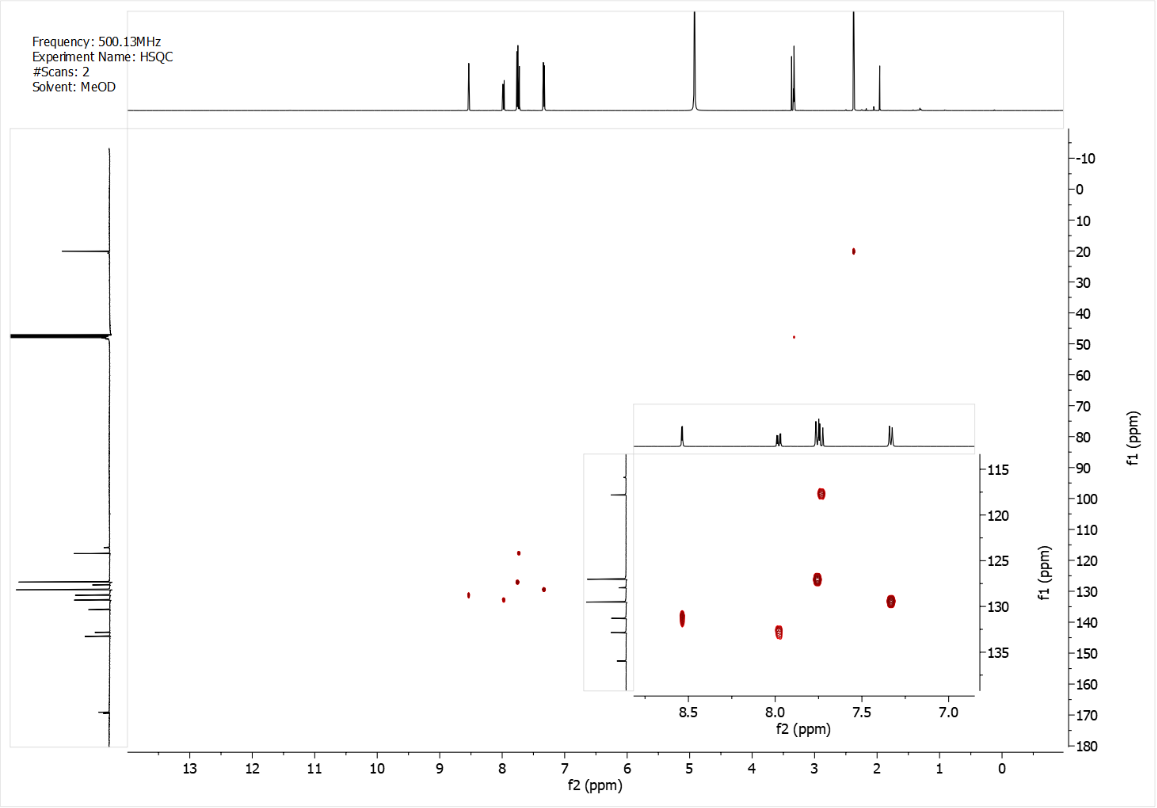

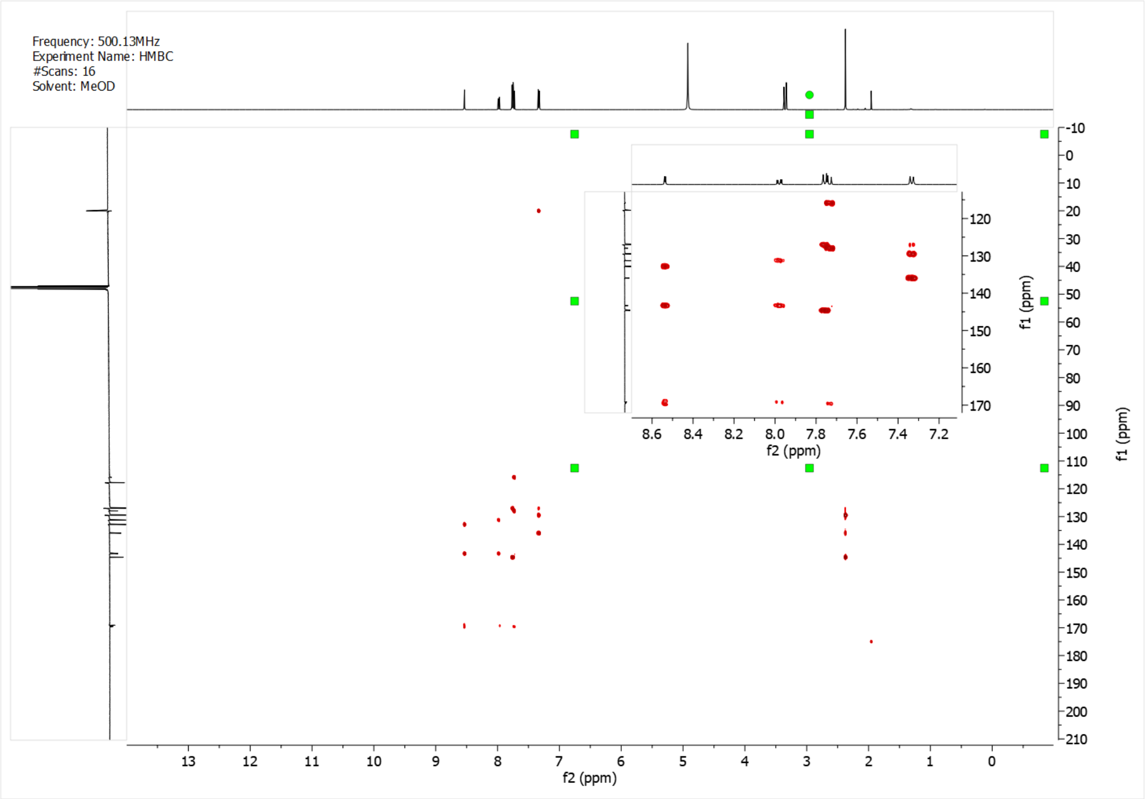

Note: Regiochemistry confirmed by HMBC. Cross-peaks between 6-H and both carbonyl carbons observed in HMBC. Cross-peak between 4-H and amide carbon observed in HMBC. Cross-peak between H-3 and carboxylic acid carbon.

5-(Methylcarbamoyl)-2-((4-methylphenyl)sulfonamido)benzoic acid (2j)

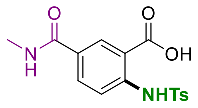

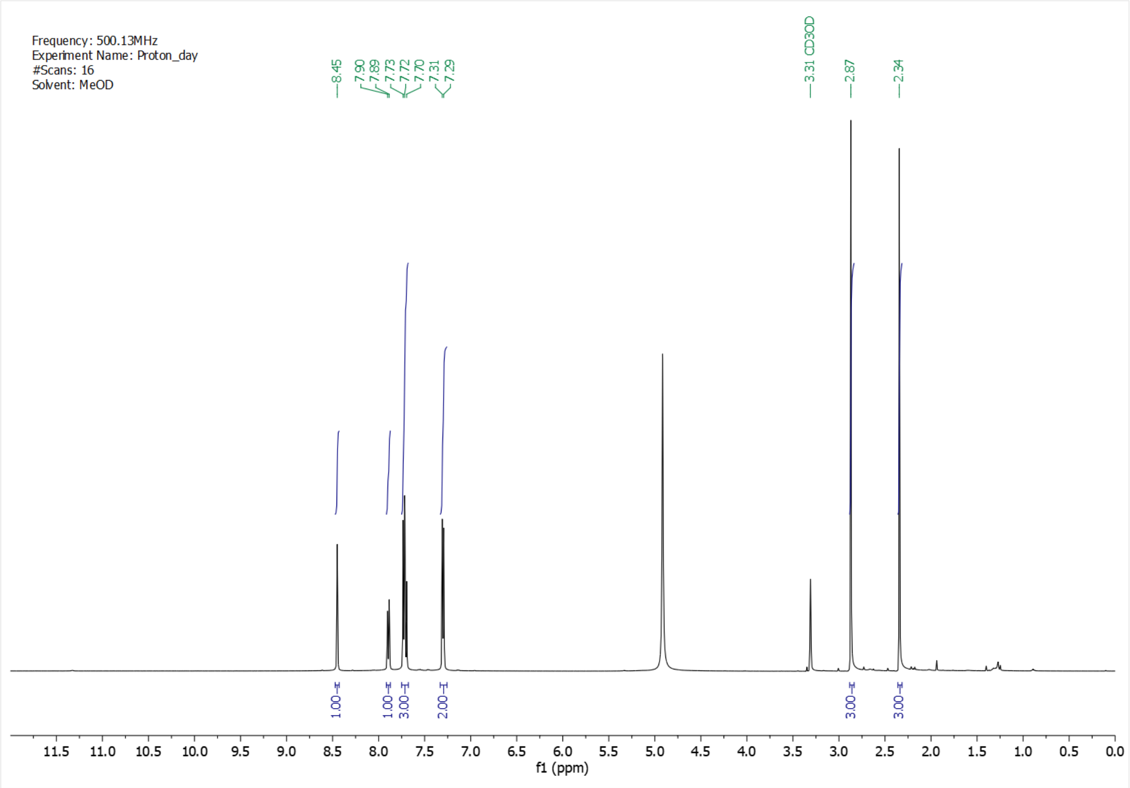

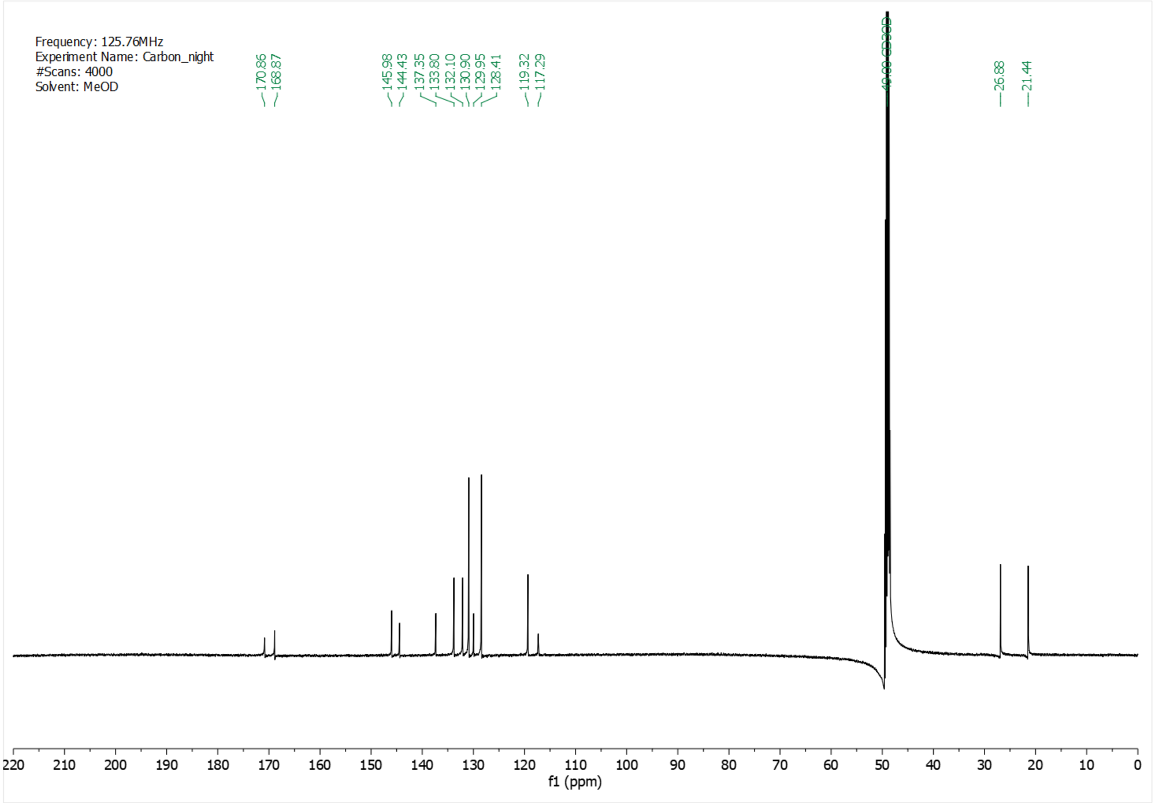

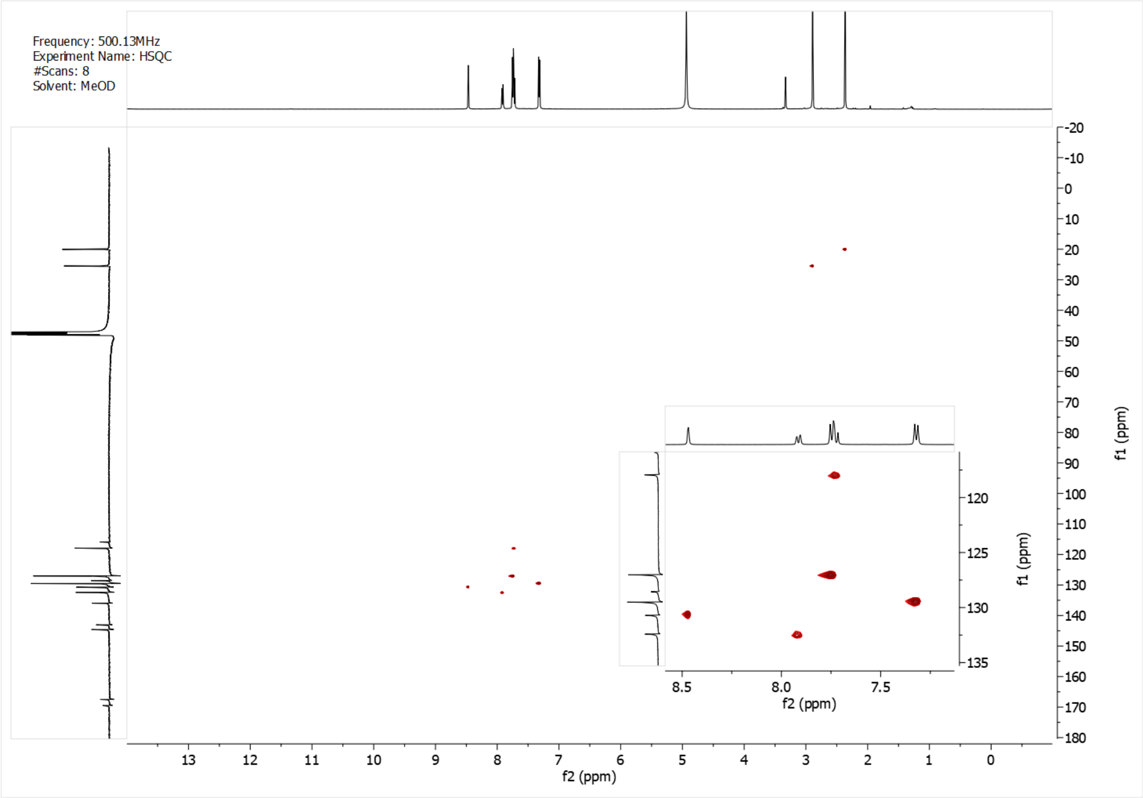

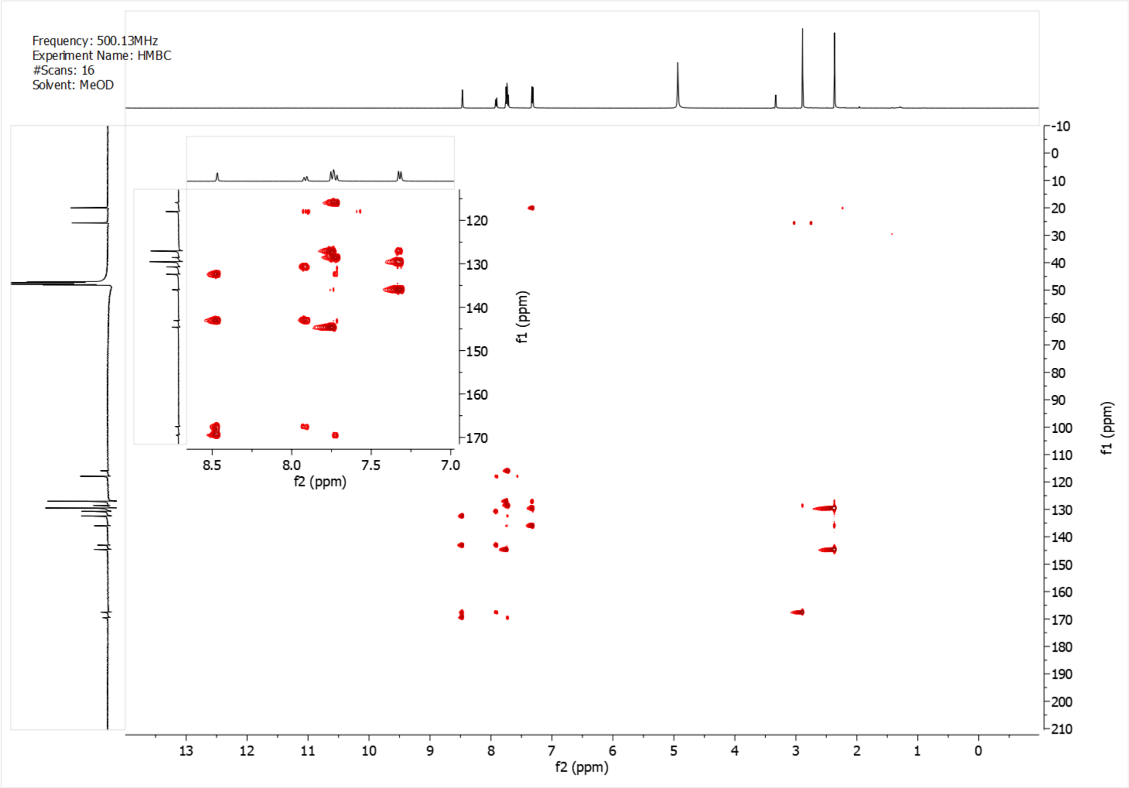

Note: Regiochemistry confirmed by HMBC. Cross-peaks between 6-H and both carbonyl carbons observed in HMBC. Cross-peak between 4-H and amide carbon observed in HMBC. Cross-peak between H-3 and carboxylic acid carbon, cross peak with amide carbon only at high magnification (low signal-to-noise ratio).

5-(Dimethylcarbamoyl)-2-((4-methylphenyl)sulfonamido)benzoic acid (2k)

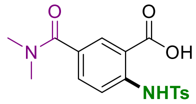

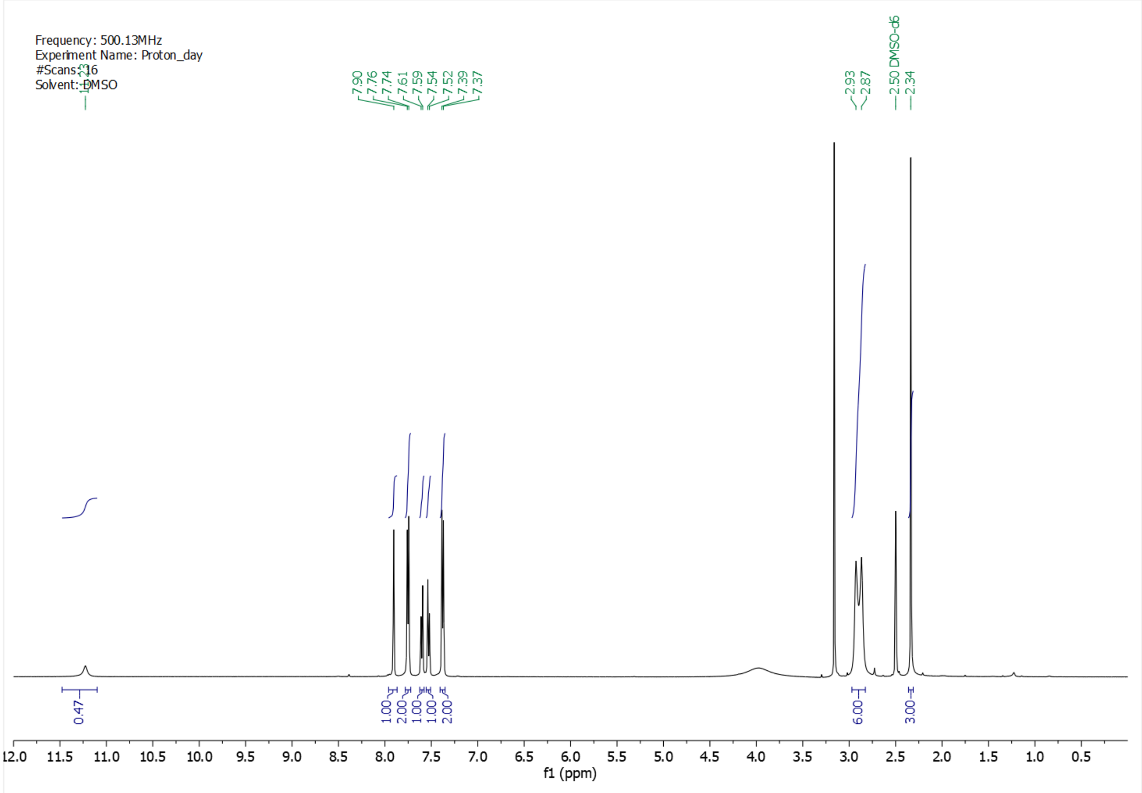

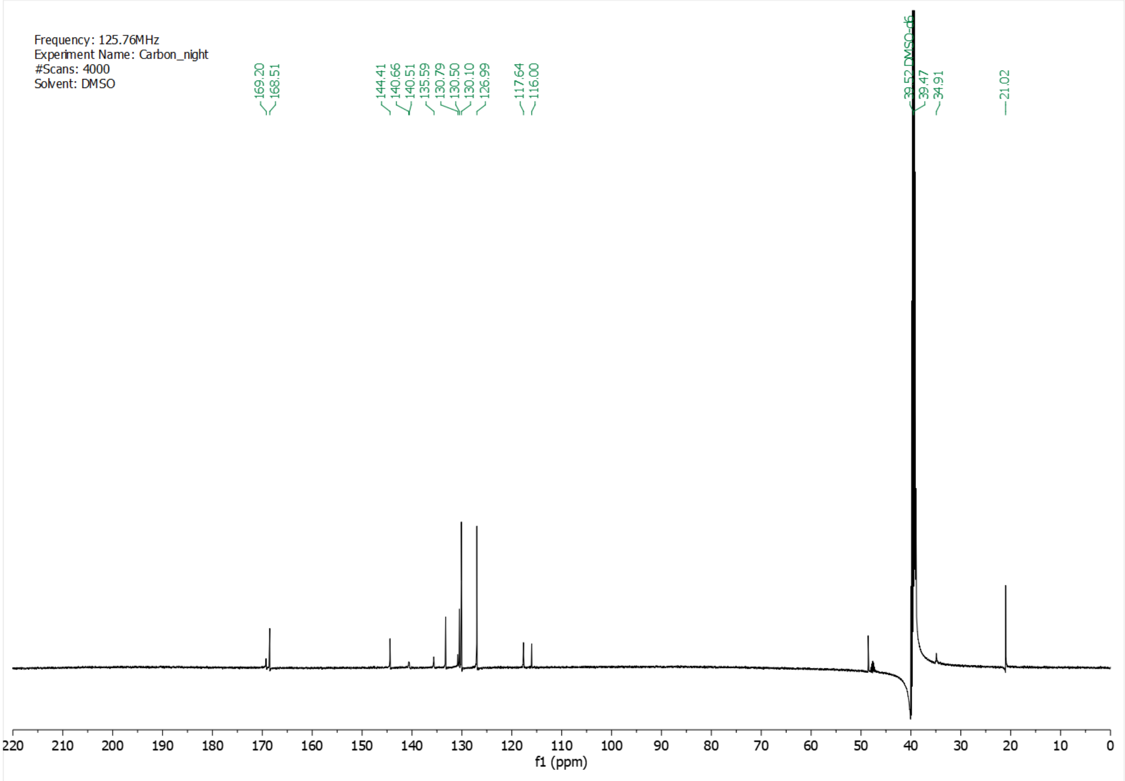

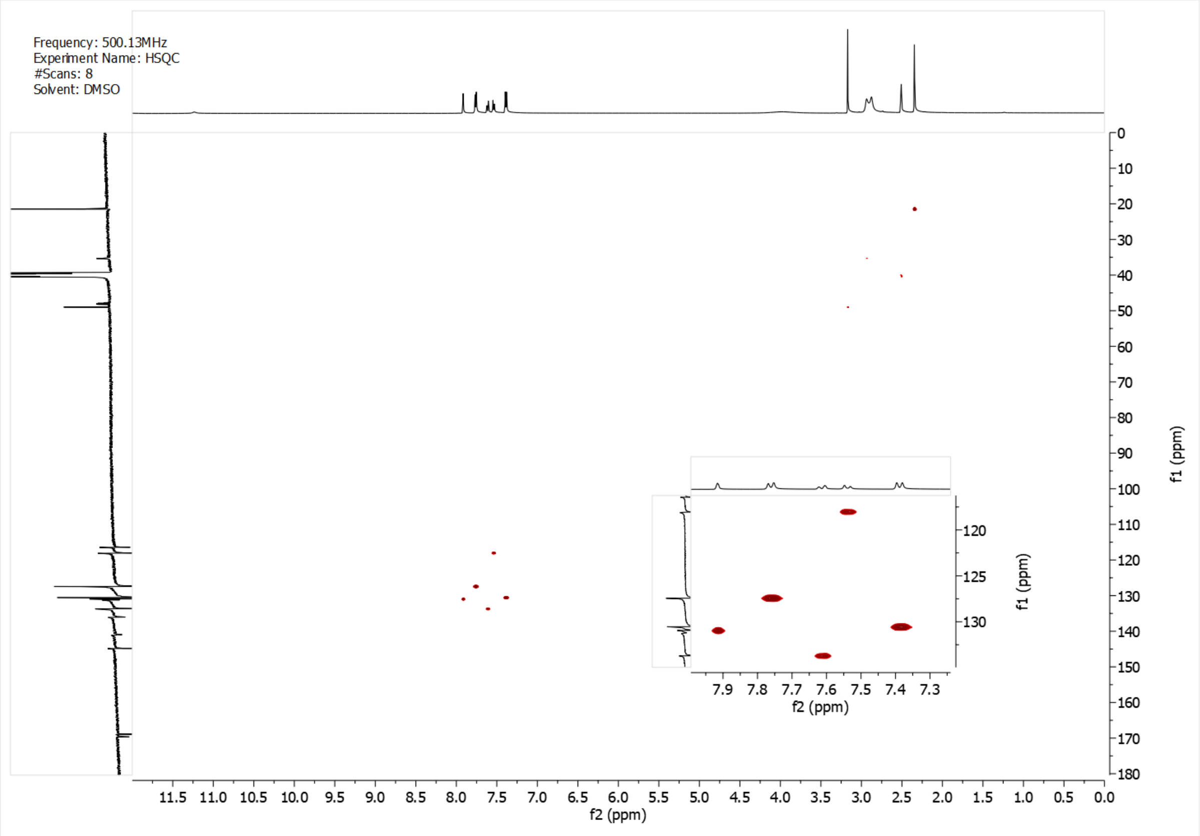

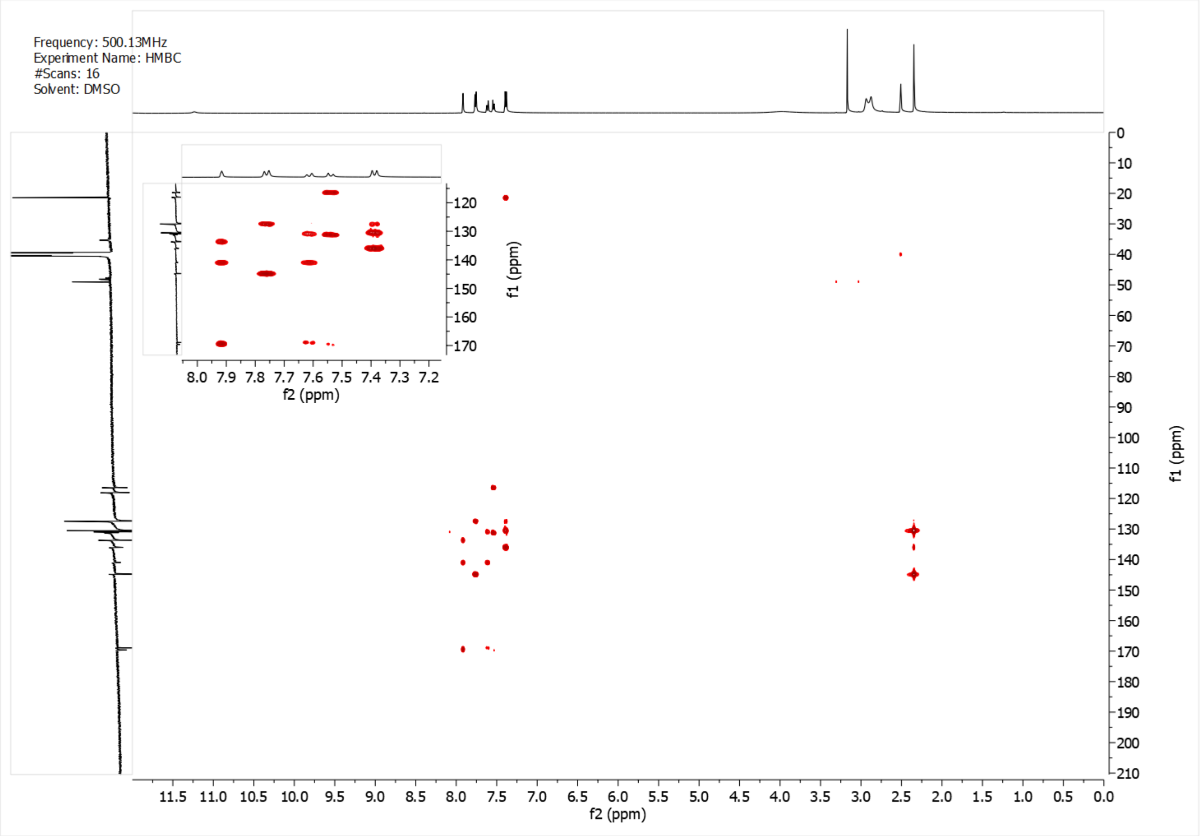

Note: Regiochemistry confirmed by HMBC. Cross-peaks between 6-H and both carbonyl carbons observed in HMBC. Cross-peak between 4-H and amide carbon observed in HMBC. Cross-peak between 3-H and carboxylic acid carbon.

2-((4-Methylphenyl)sulfonamido)-1-naphthoic acid (2l)

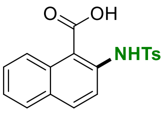

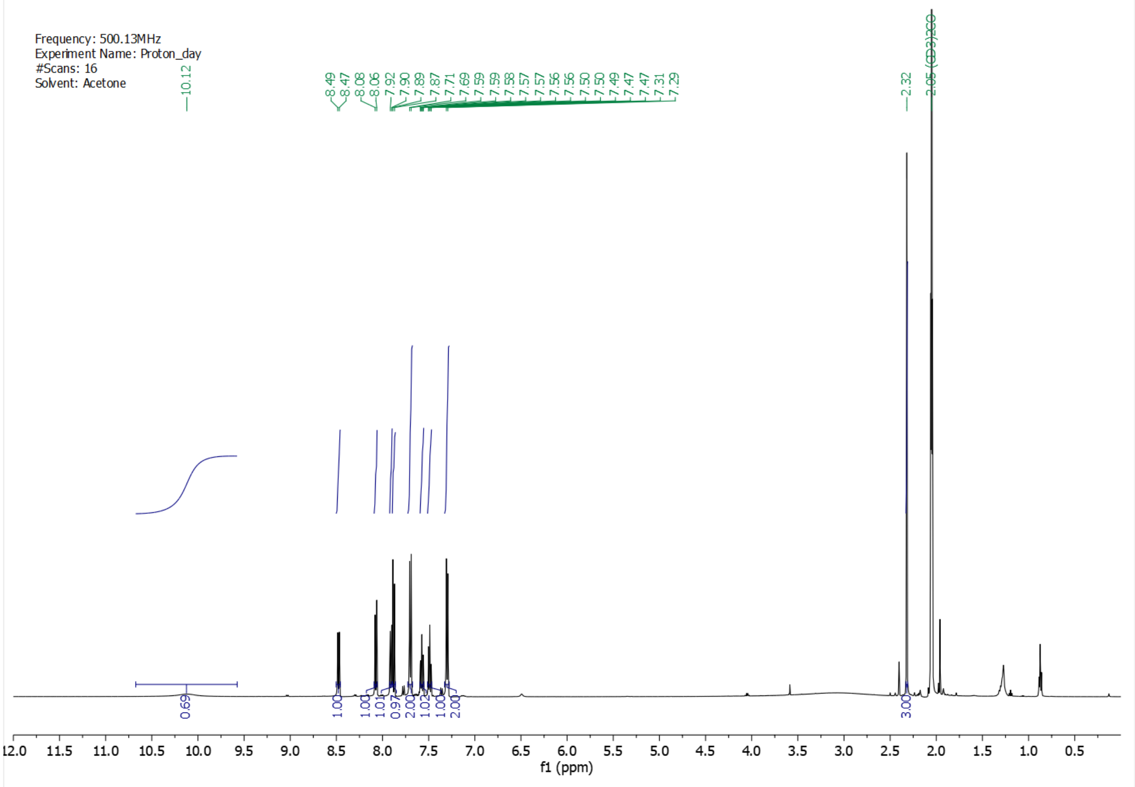

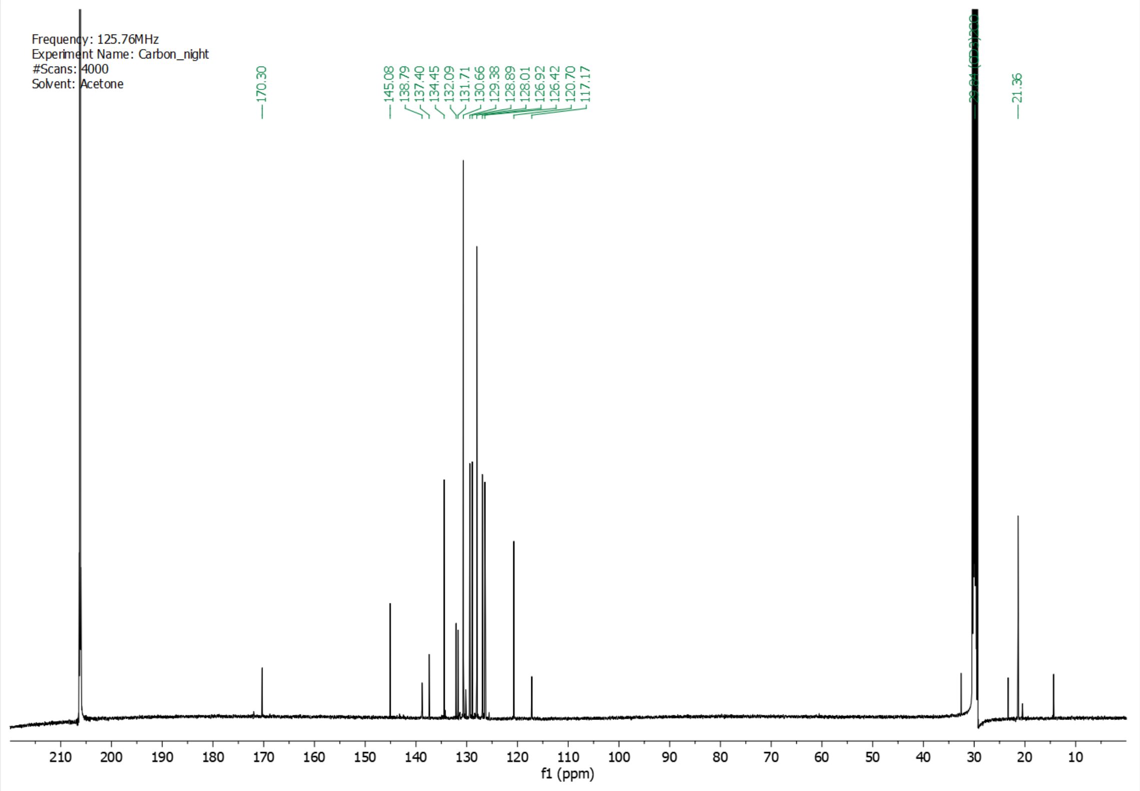

3-Bromo-2-fluoro-6-((4-methylphenyl)sulfonamido)benzoic acid (2m)

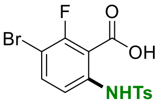

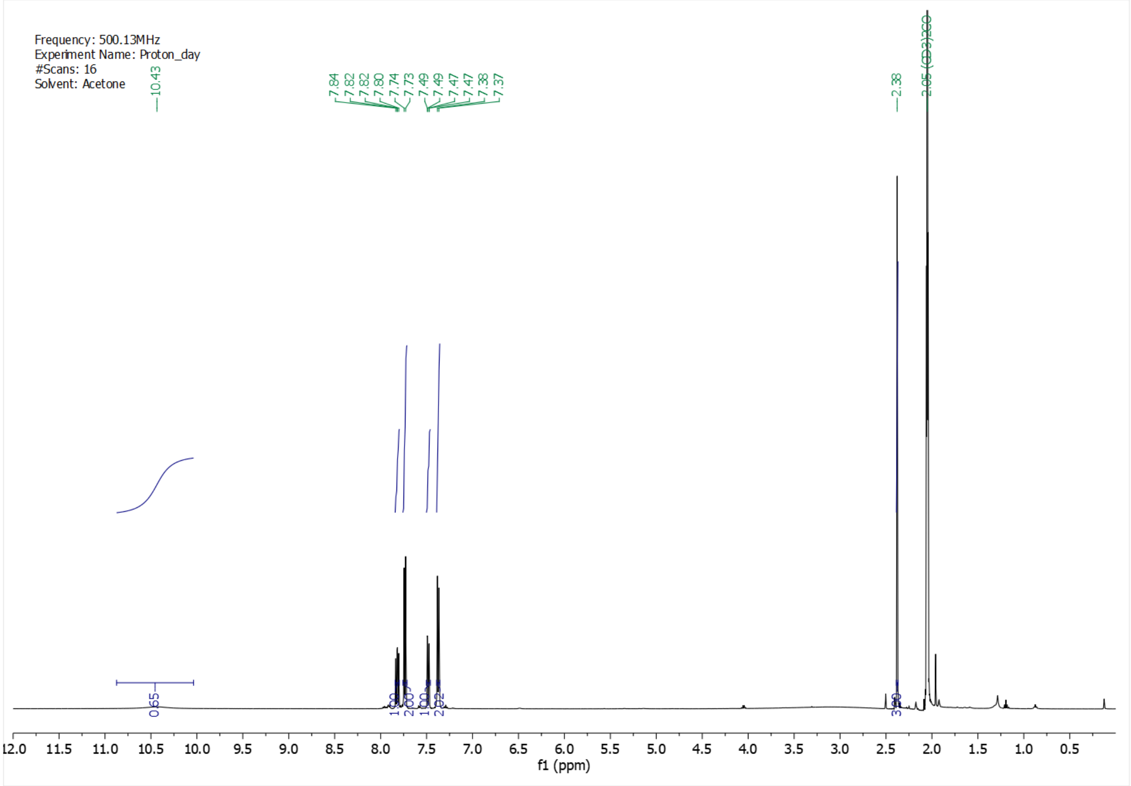

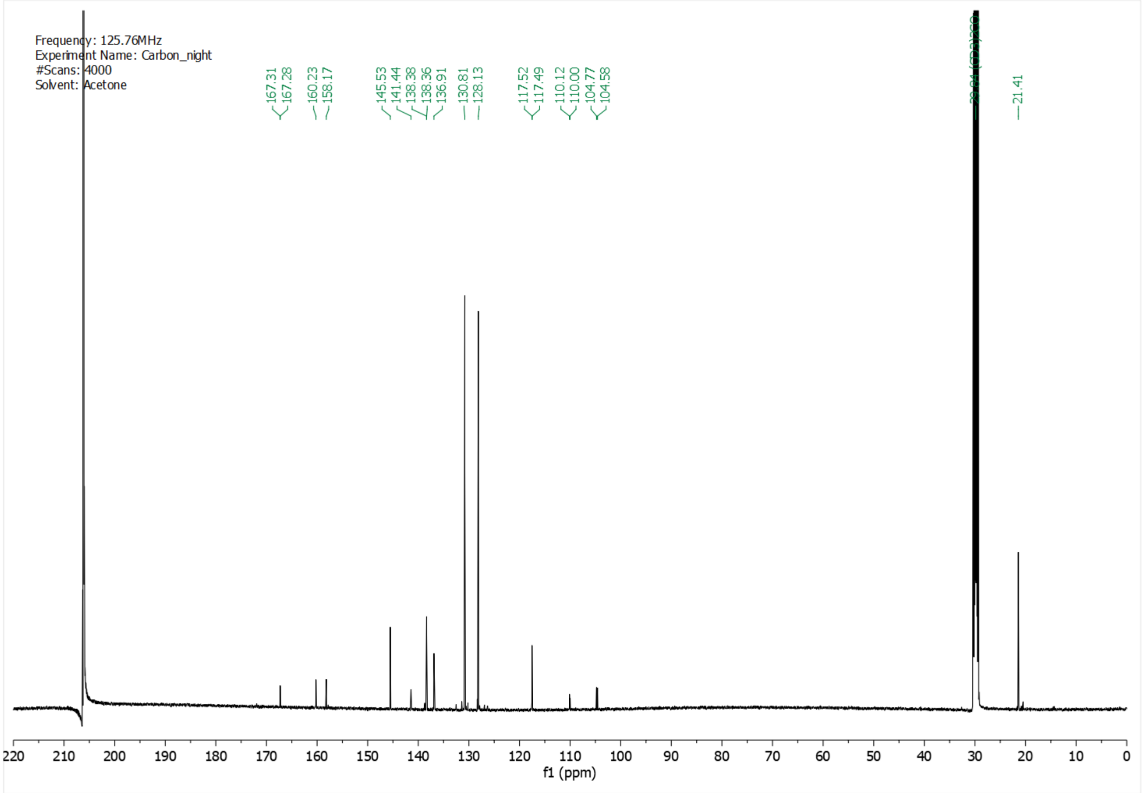

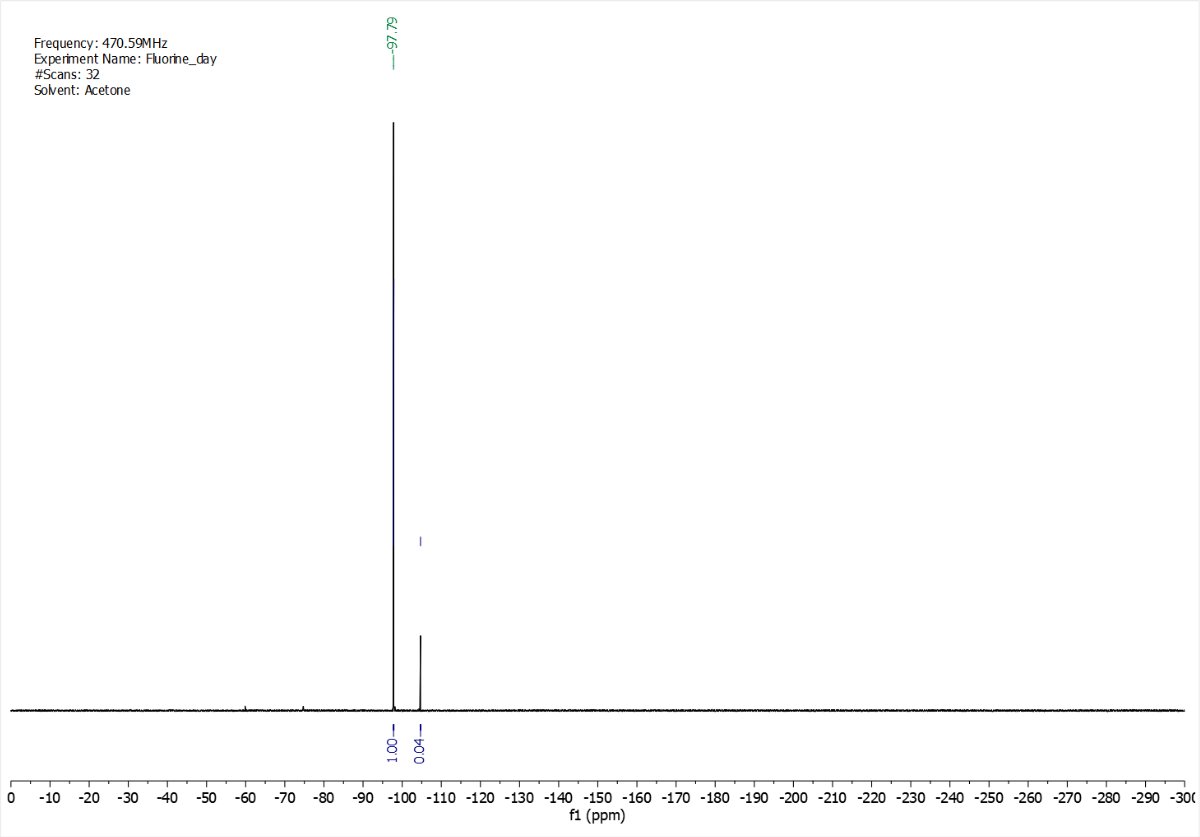

Note: Small peak (integrated as 0.04) corresponds to remaining unreacted starting material.

2,4-Difluoro-6-((4-methylphenyl)sulfonamido)benzoic acid (2n)

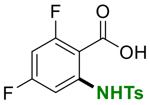

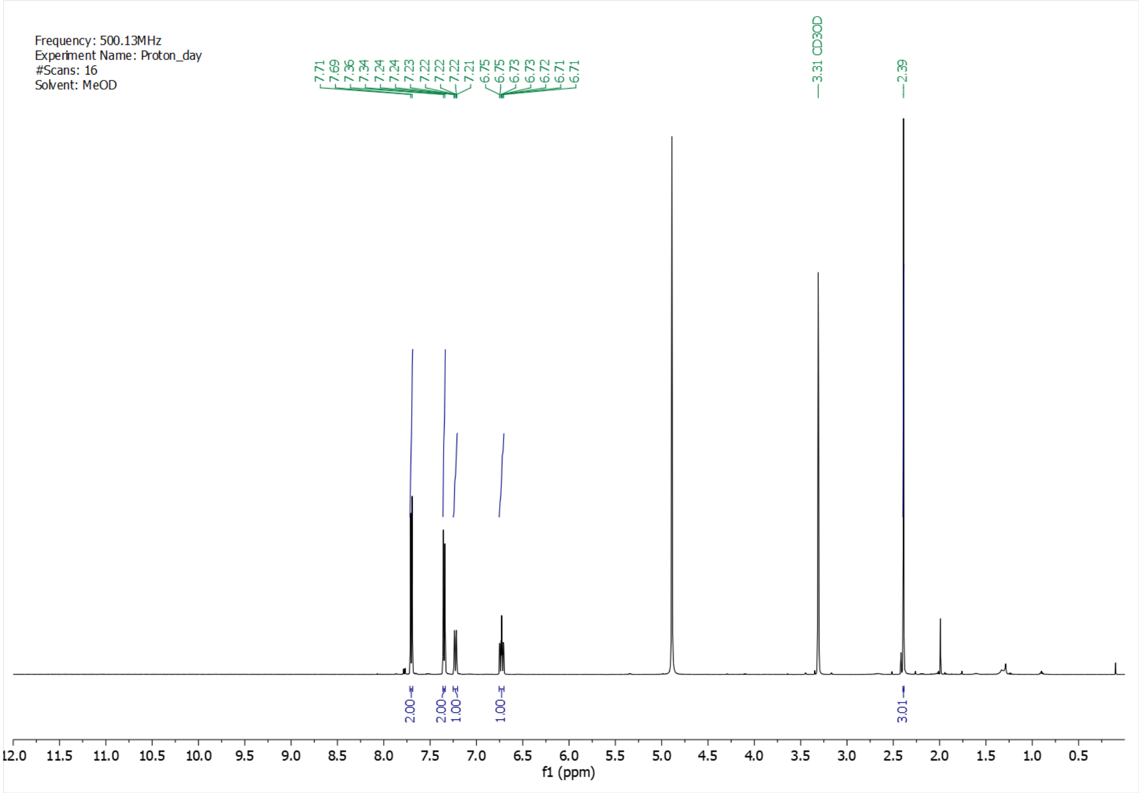

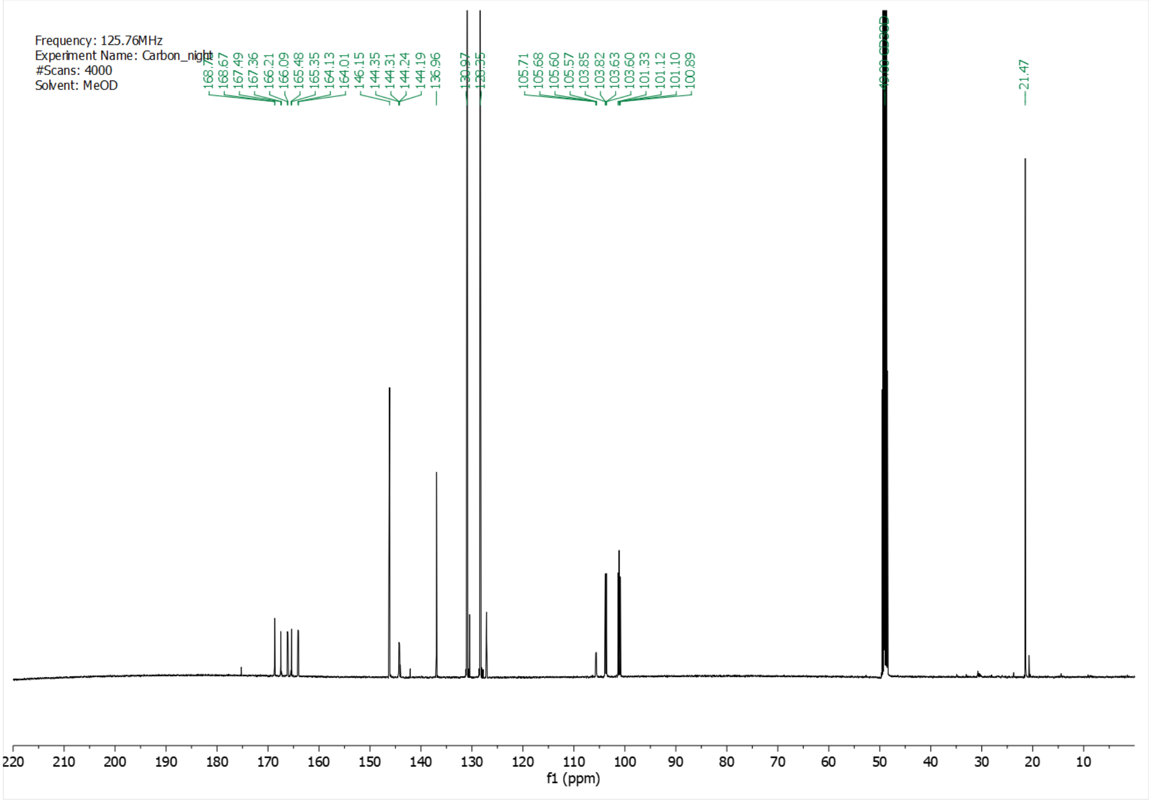

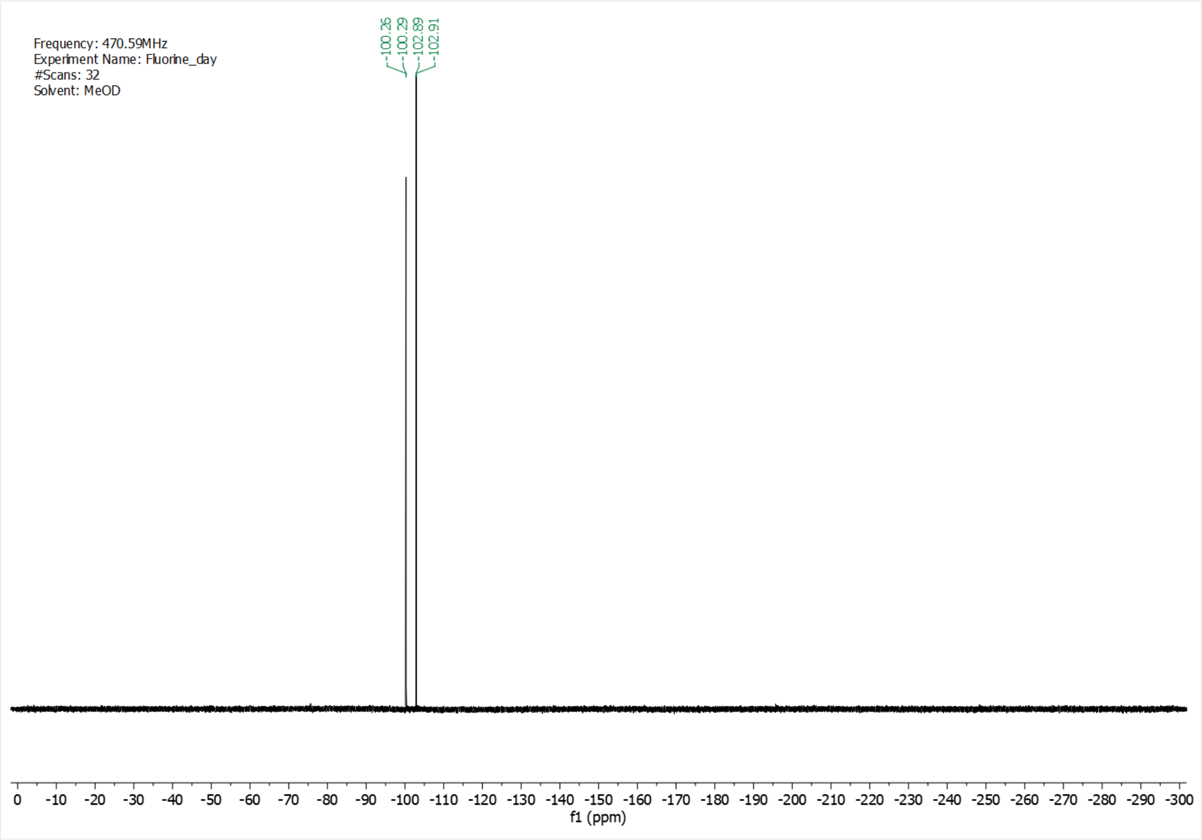
Frequency: 470.59MHz
Experiment Name: Fluorine_day
#Scans: 32
Solvent: MeOD
-100.26
-100.29
-102.89
-102.91
0 -10 -20 -30 -40 -50 -60 -70 -80 -90 -100 -110 -120 -130 -140 -150 -160 -170 -180 -190 -200 -210 -220 -230 -240 -250 -260 -270 -280 -290 -300
f1 (ppm)

2,4,5-Trifluoro-3-methoxy-6-((4-methylphenyl)sulfonamido)benzoic acid (2o)

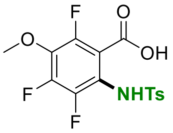

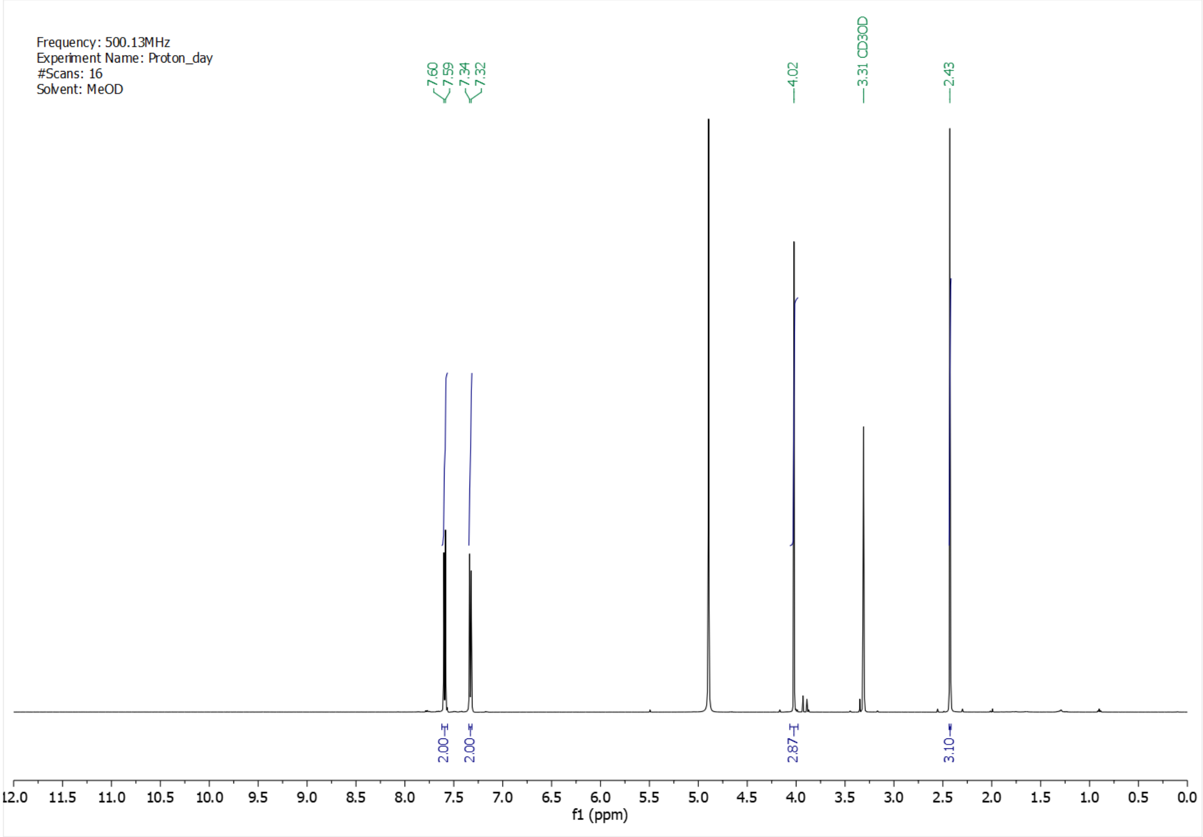

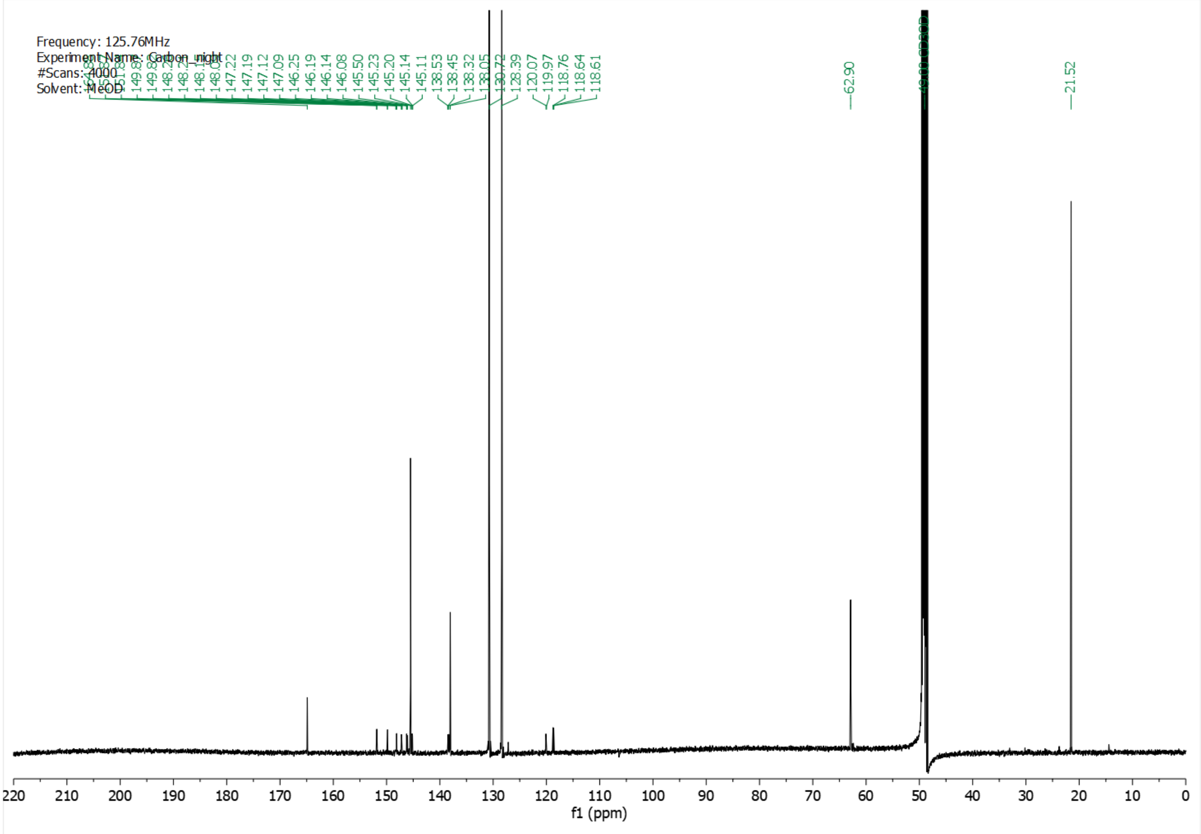
Frequency: 125.76MHz
Experiment Name: Carbon_night
#Scans: 4000
Solvent: MeOD
147.22
147.19
147.12
147.09
146.25
146.19
146.14
146.08
145.50
145.23
145.20
145.14
145.11
138.53
138.45
138.32
138.05
130.72
128.39
120.07
119.97
118.76
118.64
118.61
62.90
21.52
f1 (ppm)

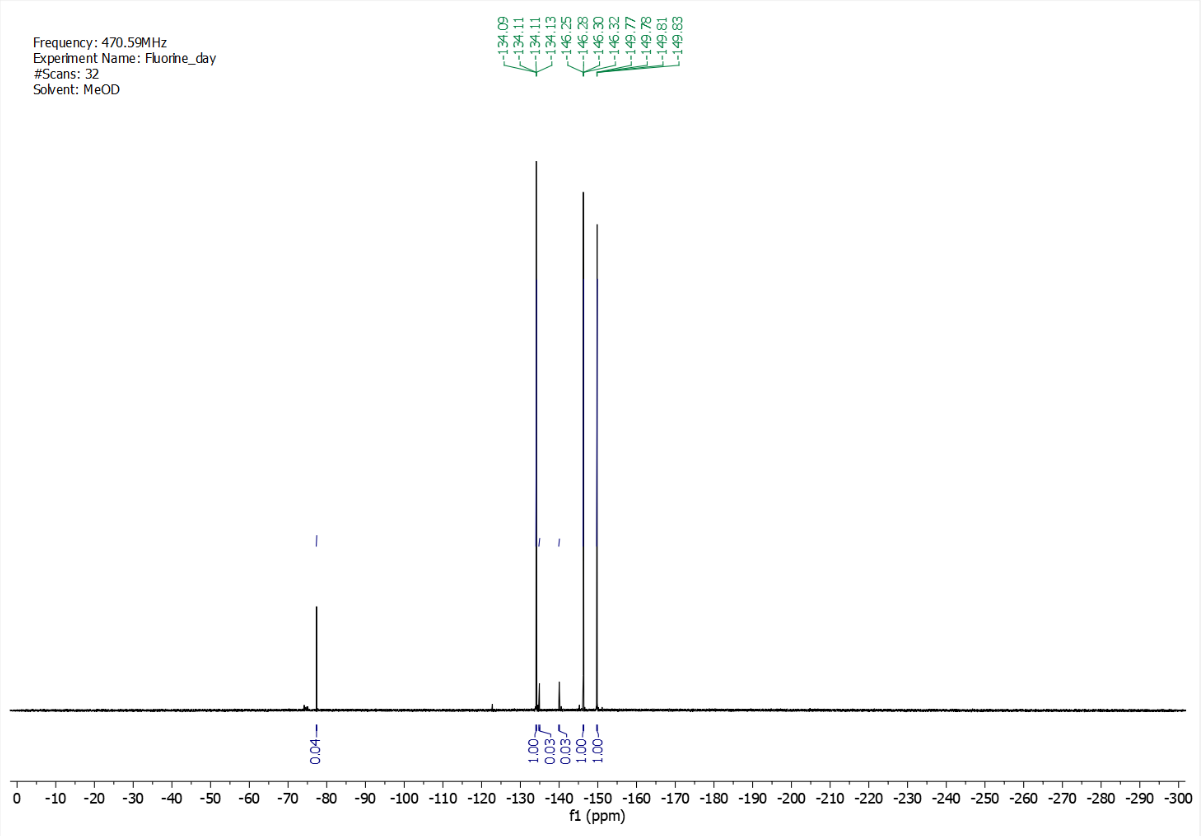
Frequency: 470.59MHz
Experiment Name: Fluorine_day
#Scans: 32
Solvent: MeOD
-134.09
-134.11
-134.11
-134.13
-146.25
-146.28
-146.30
-146.32
-149.77
-149.78
-149.81
-149.83
0.04
1.00
0.03
0.03
1.00
1.00
f1 (ppm)

3-Fluoro-5-methyl-2-((4-methylphenyl)sulfonamido)benzoic acid (2p)

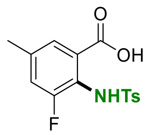

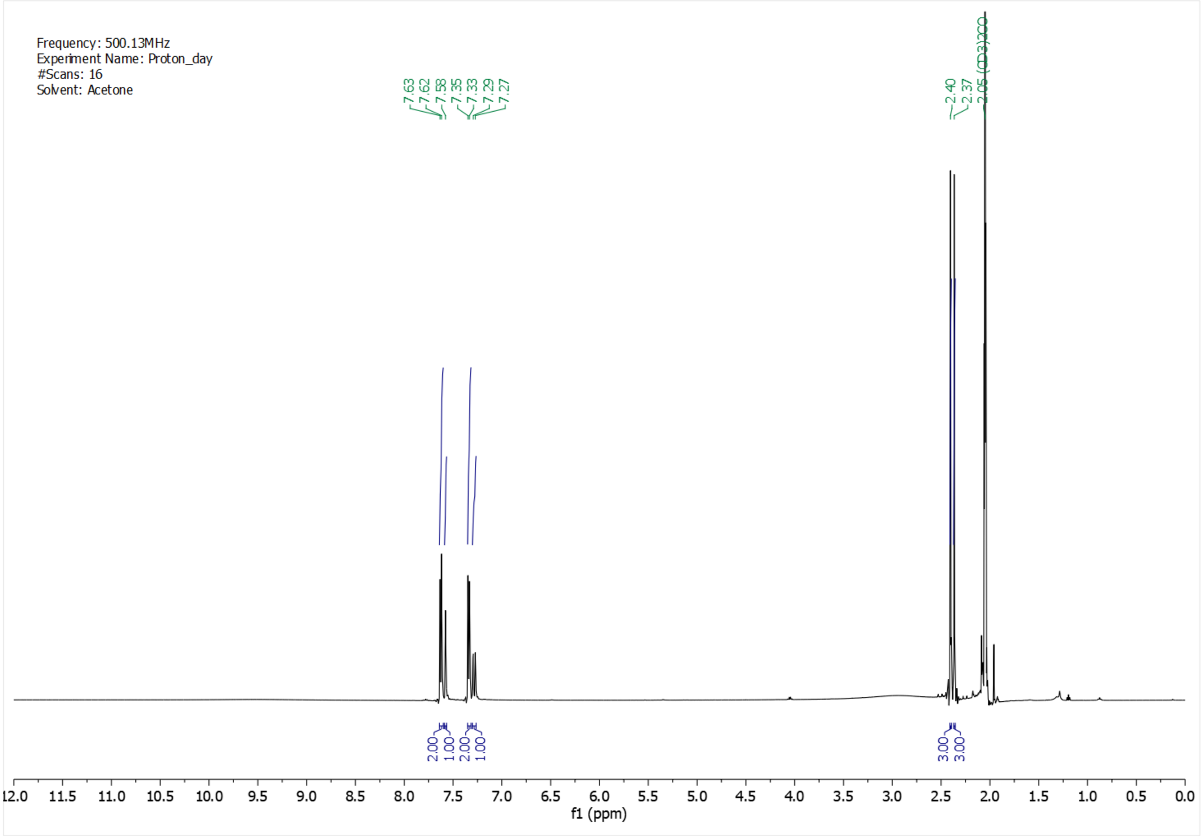

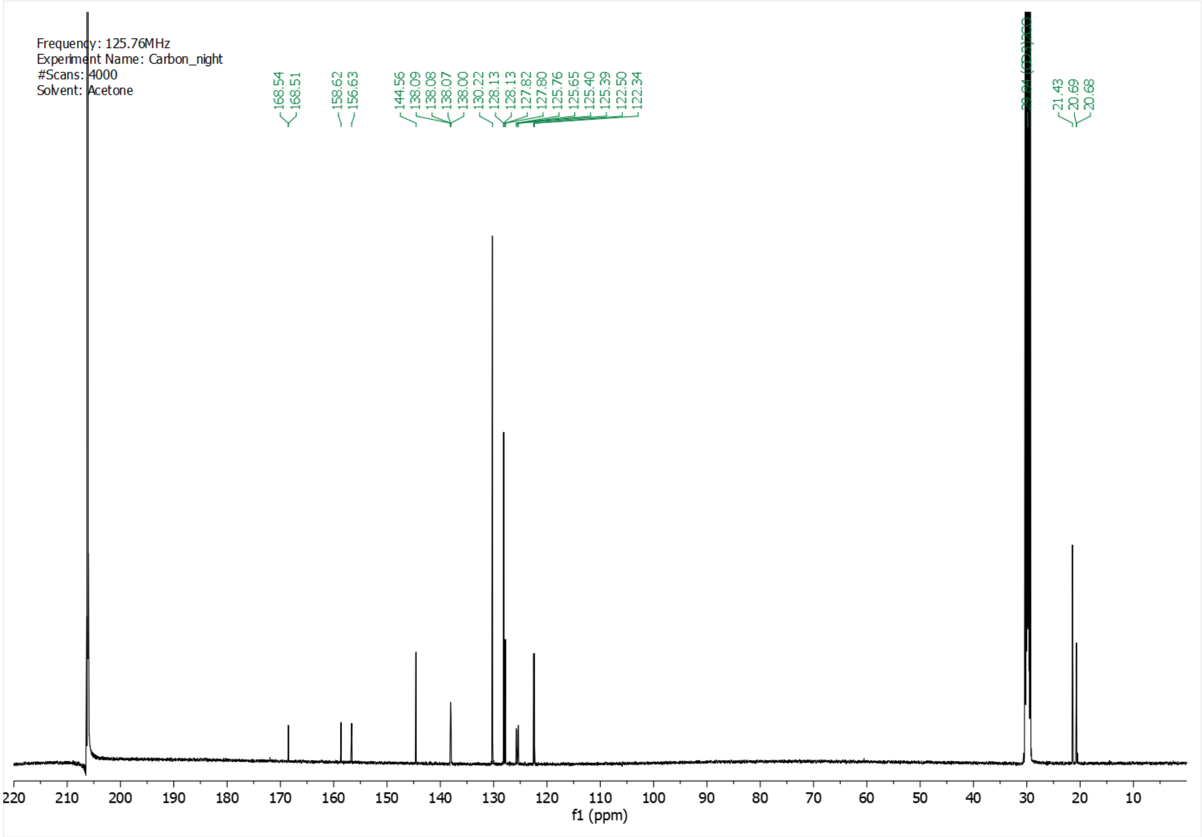
Frequency: 125.76MHz
Experiment Name: Carbon_night
#Scans: 4000
Solvent: Acetone
168.54
168.51
158.62
156.63
144.56
138.09
138.08
138.07
138.00
130.22
128.13
128.13
127.82
127.80
125.76
125.65
125.40
125.39
122.50
122.34
21.43
20.69
20.68
f1 (ppm)

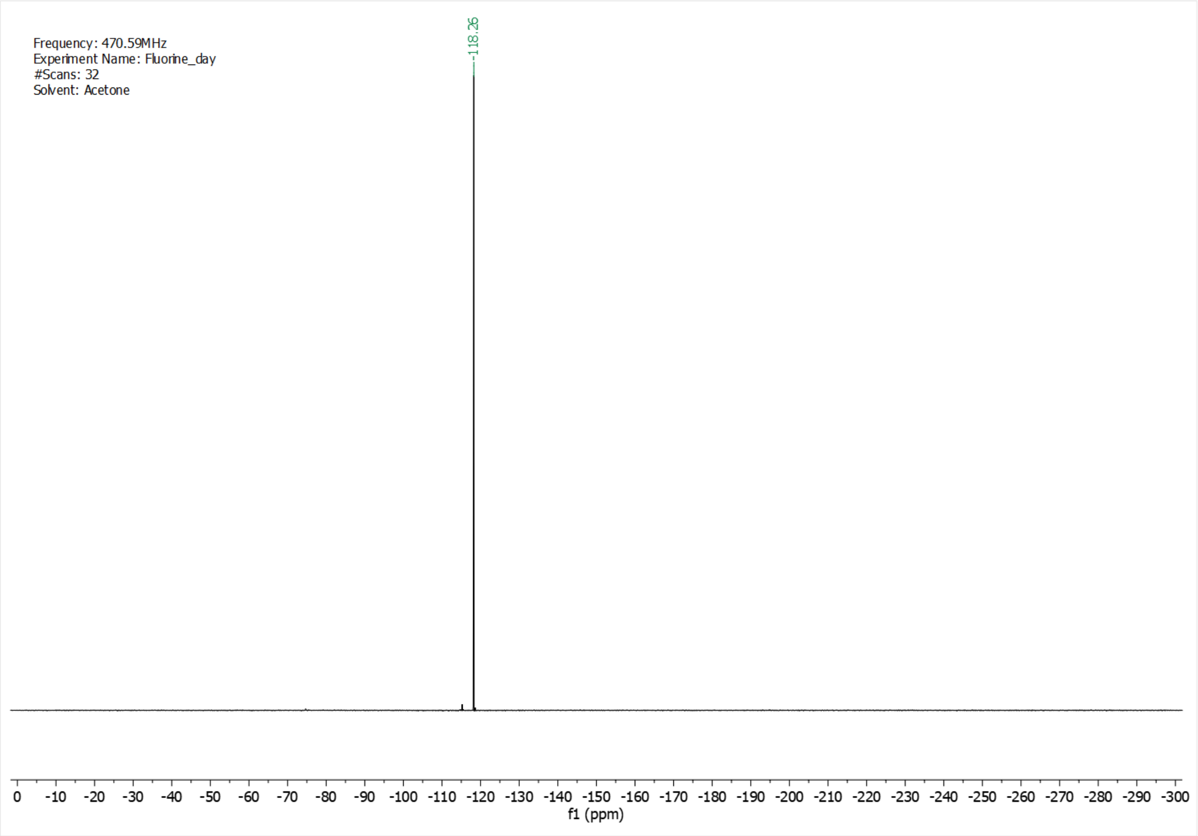
Frequency: 470.59MHz
Experiment Name: Fluorine_day
#Scans: 32
Solvent: Acetone
-118.26
f1 (ppm)

6-Fluoro-3-methoxy-2-((4-methylphenyl)sulfonamido)benzoic acid (2q)

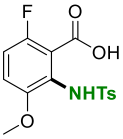

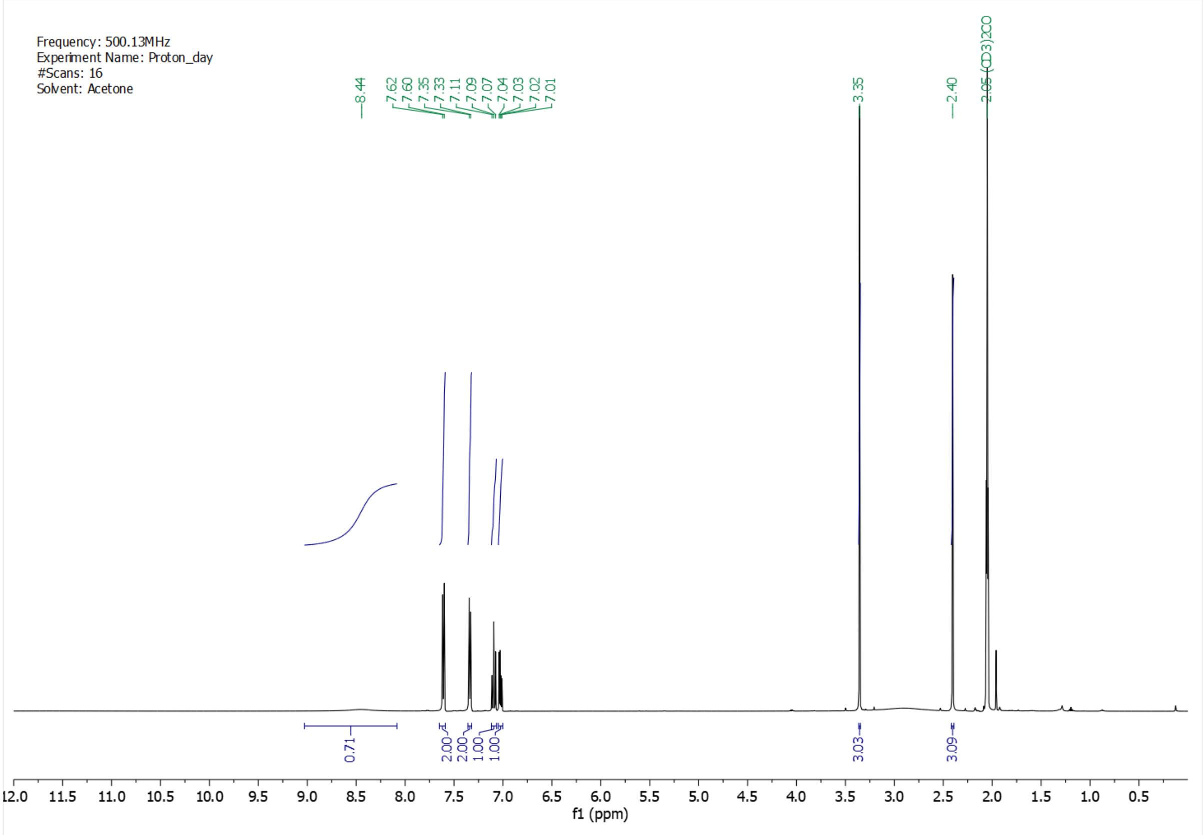

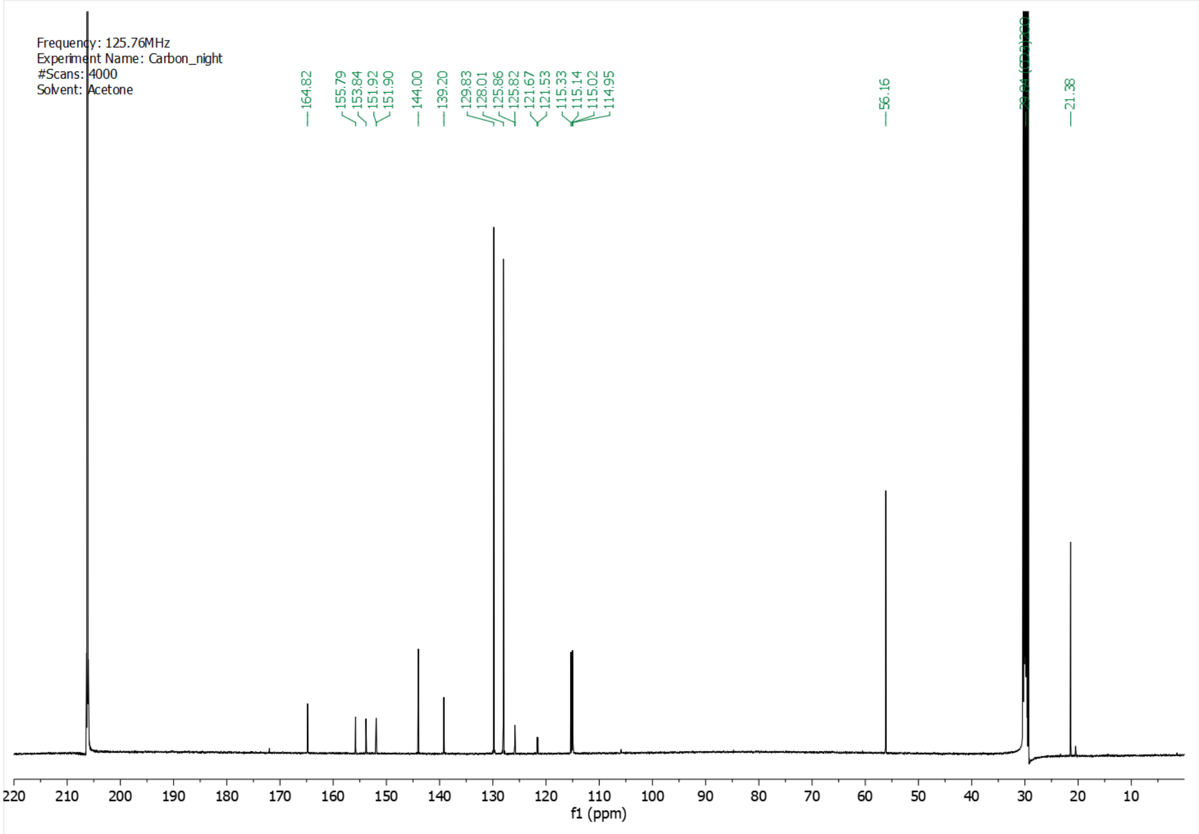
Frequency: 125.76MHz
Experiment Name: Carbon_night
#Scans: 4000
Solvent: Acetone
164.82
155.79
153.84
151.92
151.90
144.00
139.20
129.83
128.01
125.86
125.82
121.67
121.53
115.33
115.14
115.02
114.95
56.16
21.38
f1 (ppm)

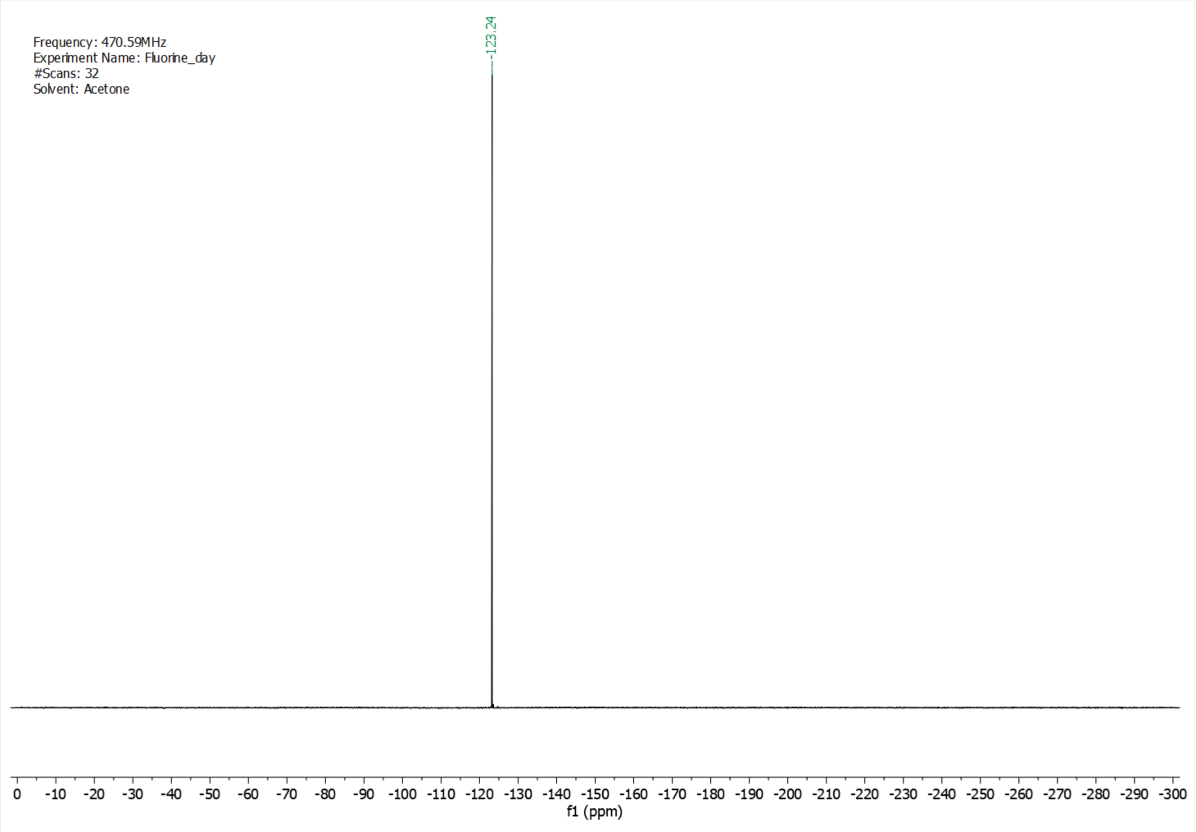
Frequency: 470.59MHz
Experiment Name: Fluorine_day
#Scans: 32
Solvent: Acetone
-123.24
f1 (ppm)

5-((4-Methylphenyl)sulfonamido)benzo[d][1,3]dioxole-4-carboxylic acid (2r)

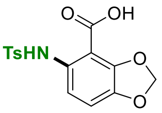

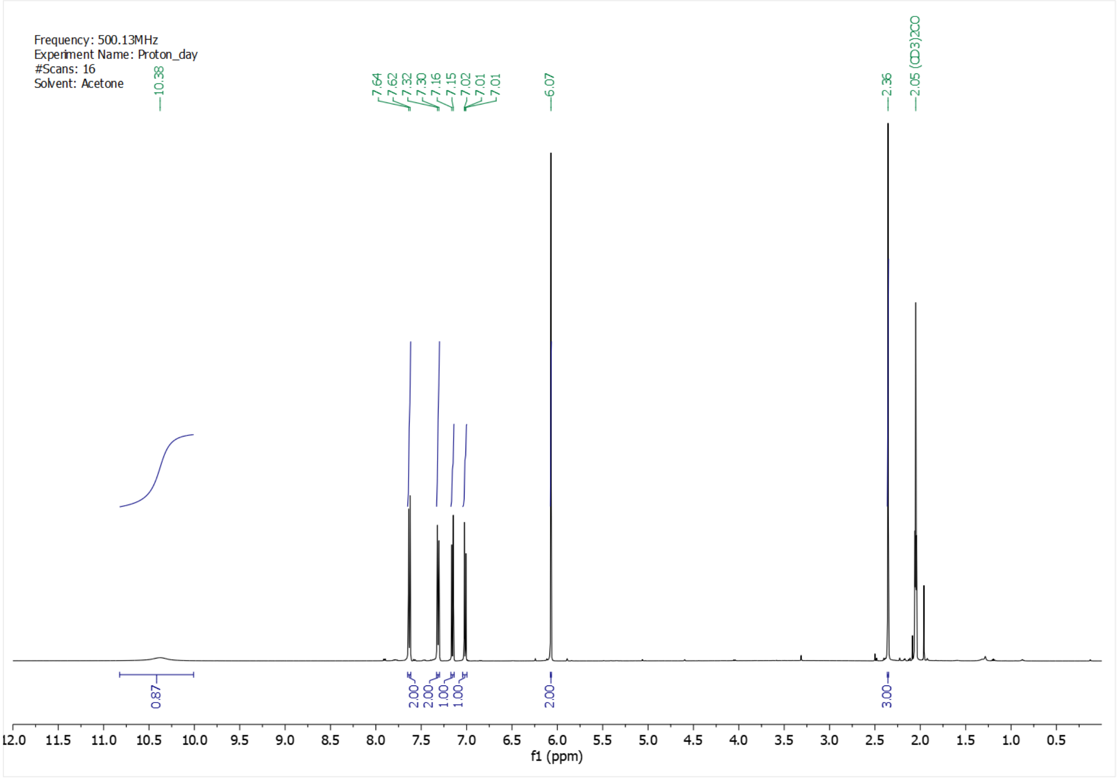

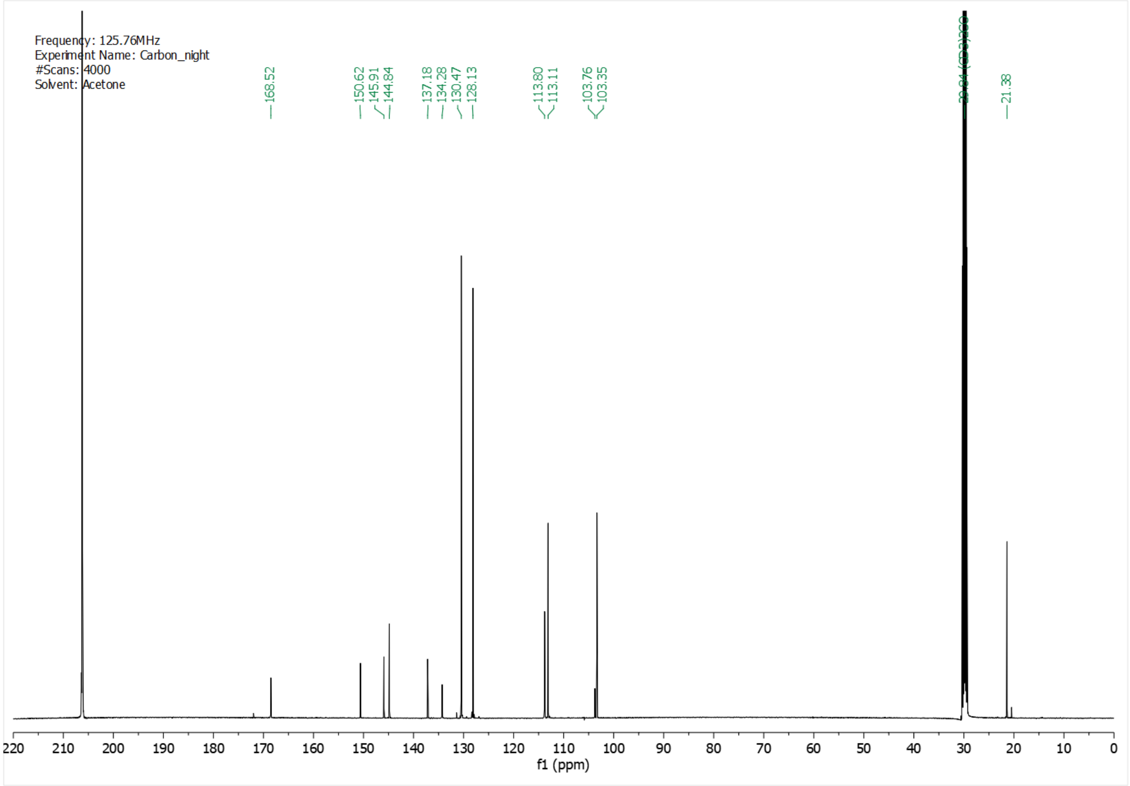

3-((4-Methylphenyl)sulfonamido)thiophene-2-carboxylic acid (2s)

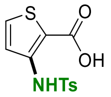

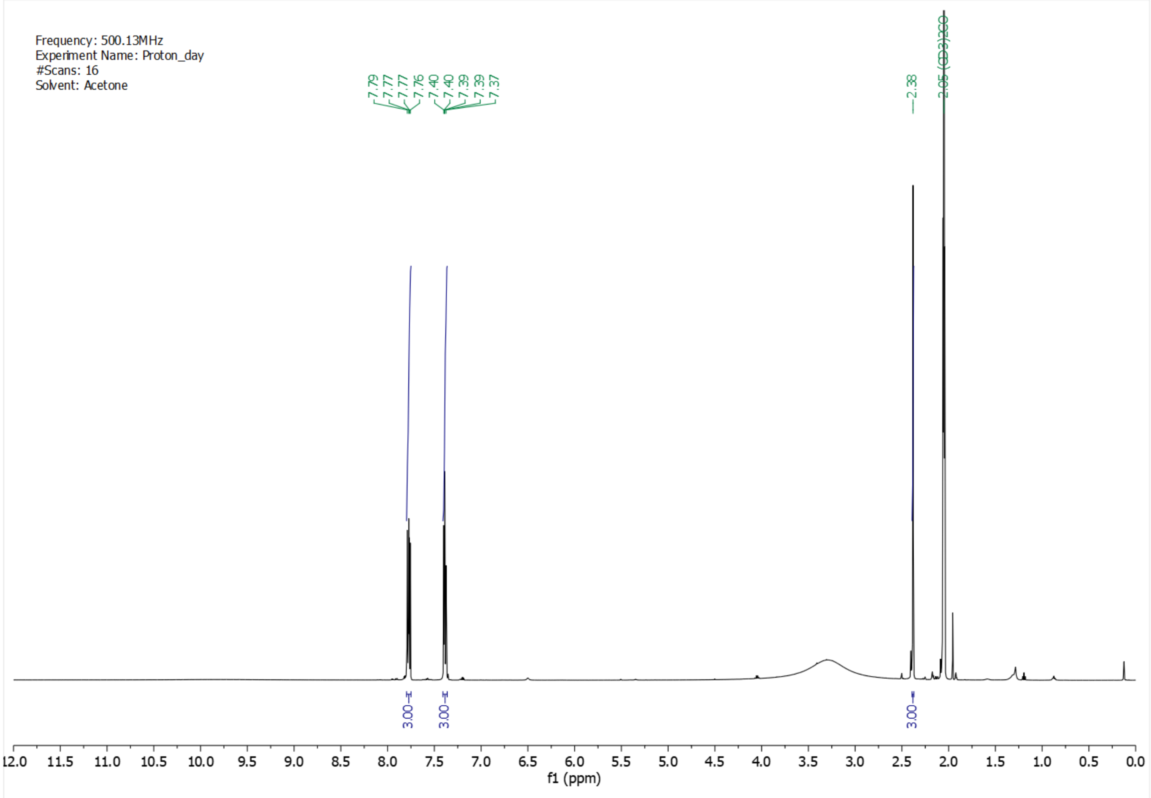

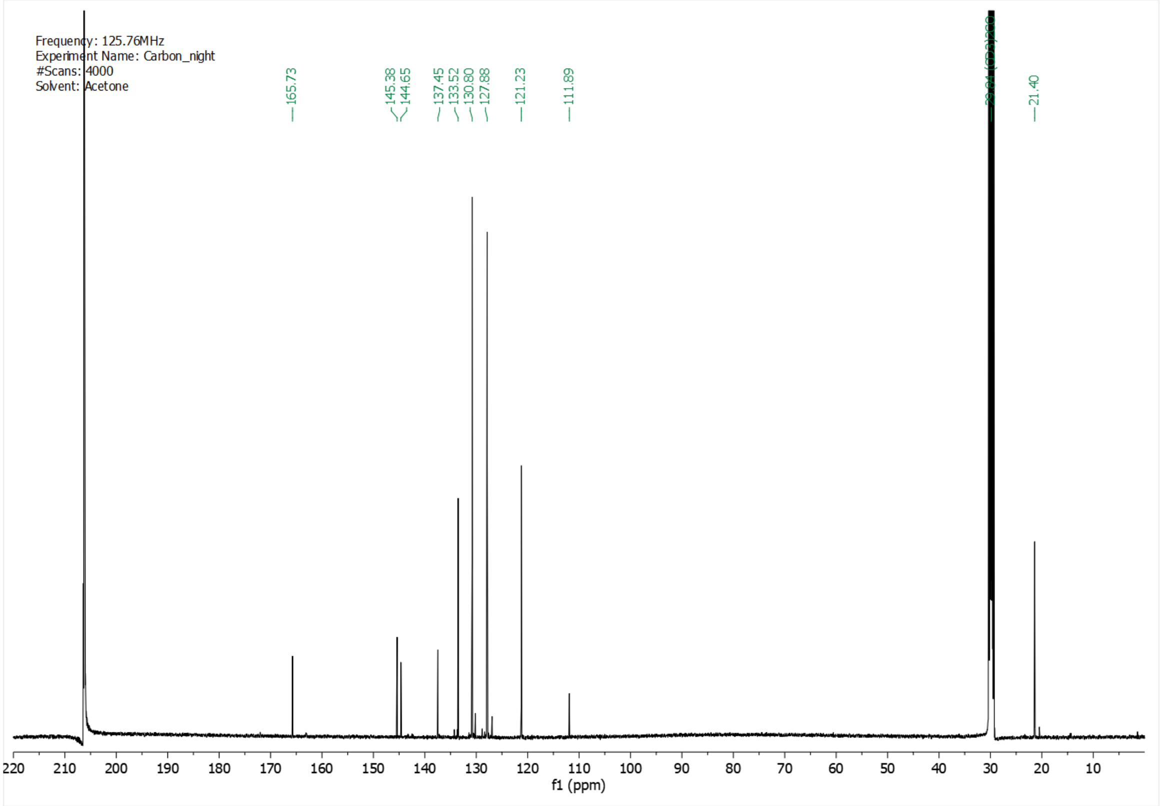

2,6-Bis((4-methylphenyl)sulfonamido)benzoic acid (2t)

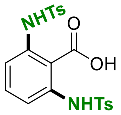

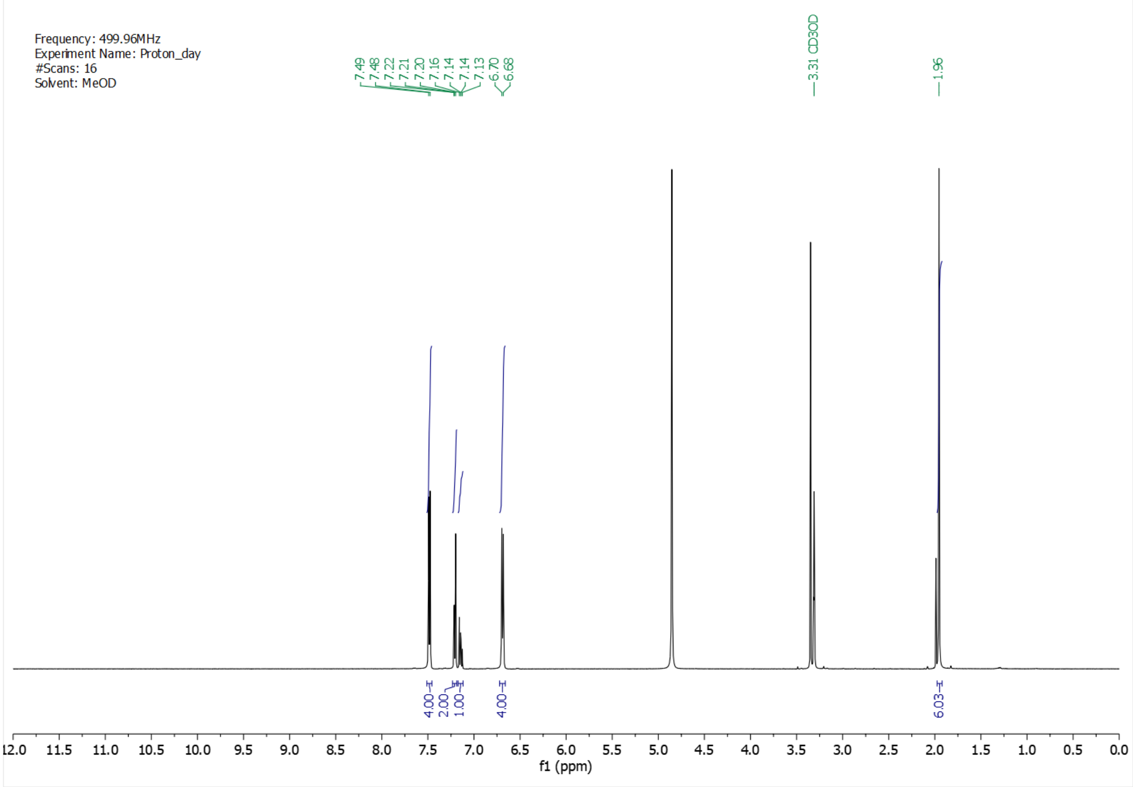

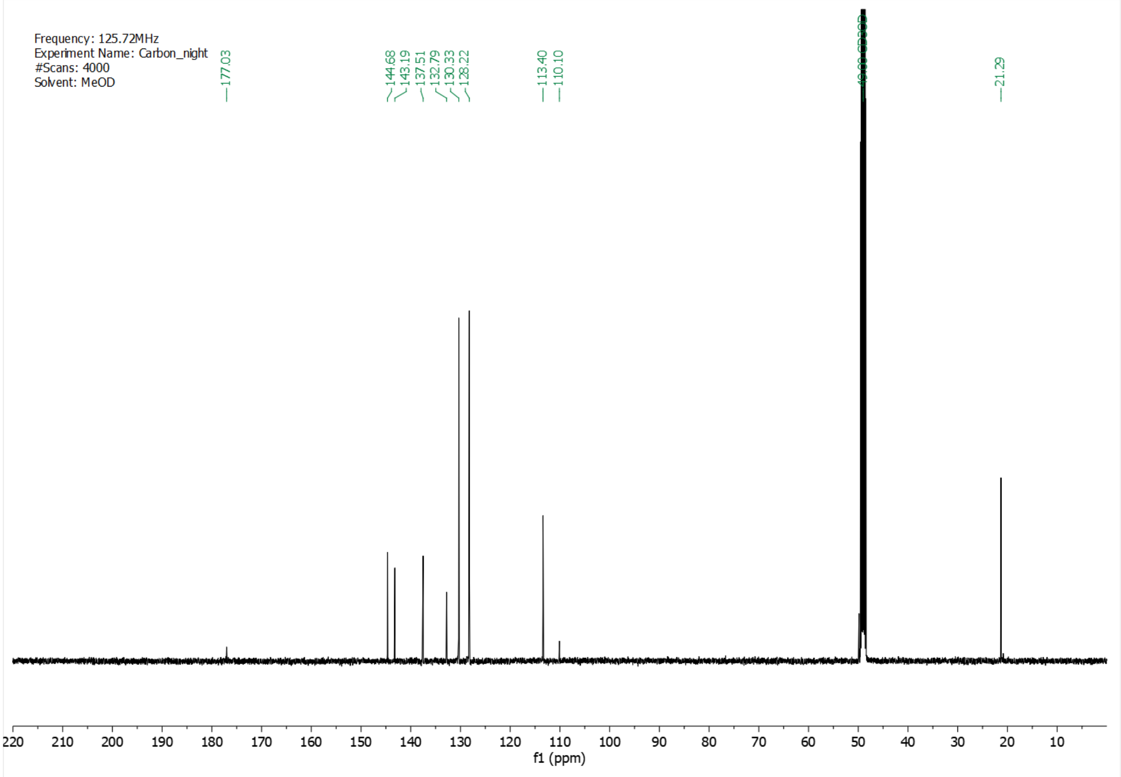

2,6-Bis((4-methylphenyl)sulfonamido)-4-(trifluoromethyl)benzoic acid (**2u**)

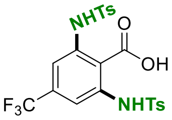

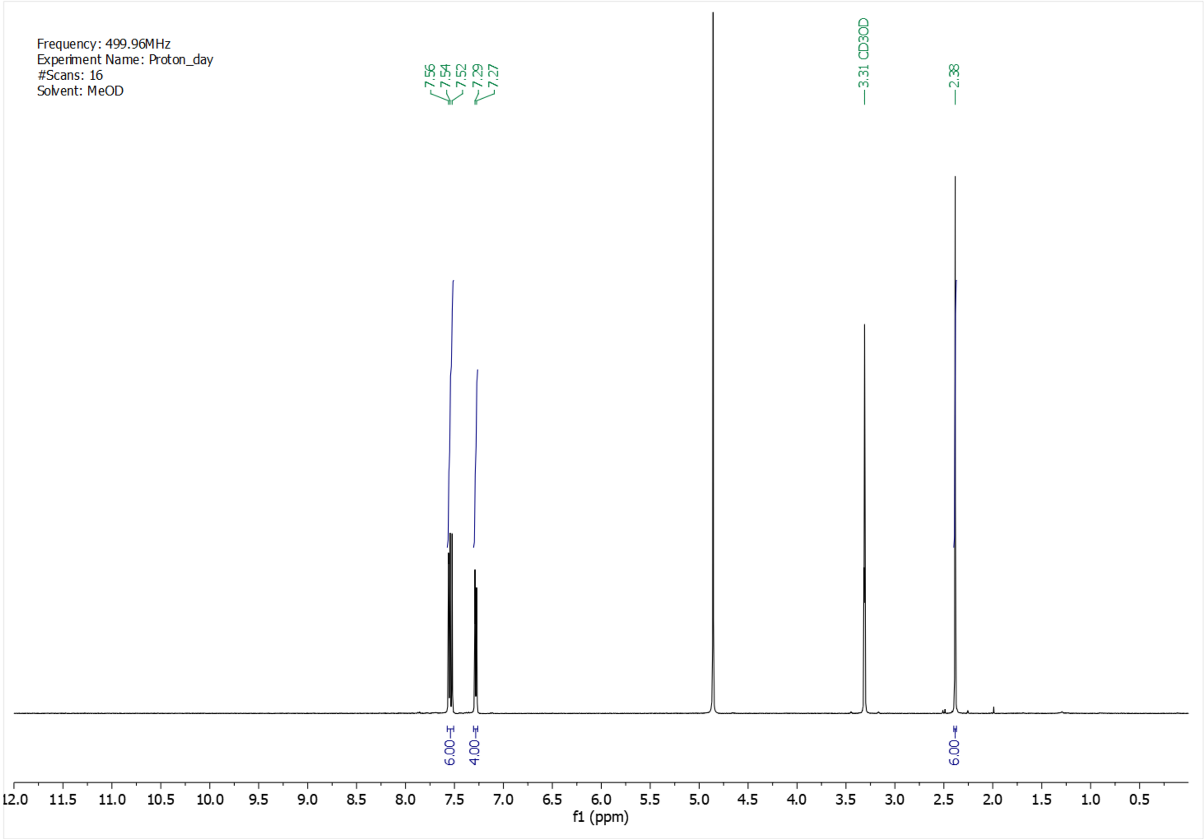

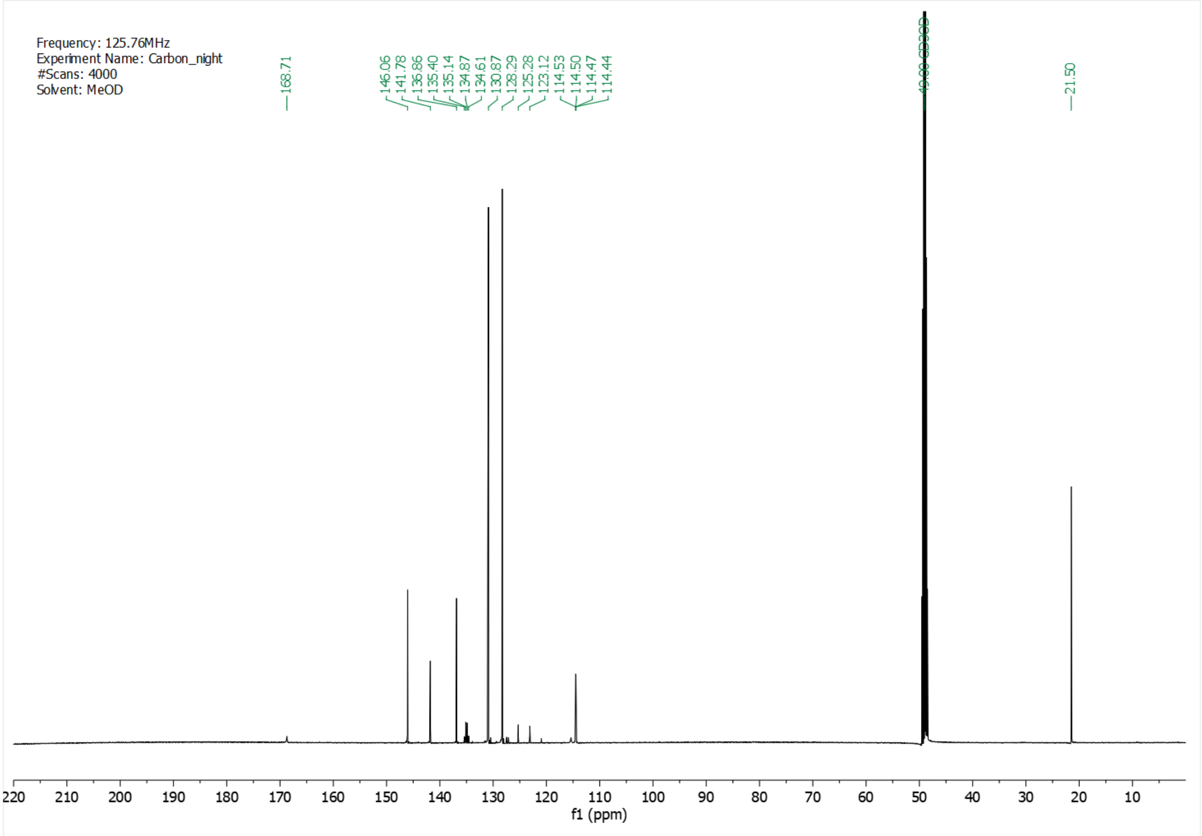
Frequency: 125.76MHz
Experiment Name: Carbon_night
#Scans: 4000
Solvent: MeOD
168.71
146.06
141.78
136.86
135.40
135.14
134.87
134.61
130.87
128.29
125.28
123.12
114.53
114.50
114.47
114.44
21.50
f1 (ppm)

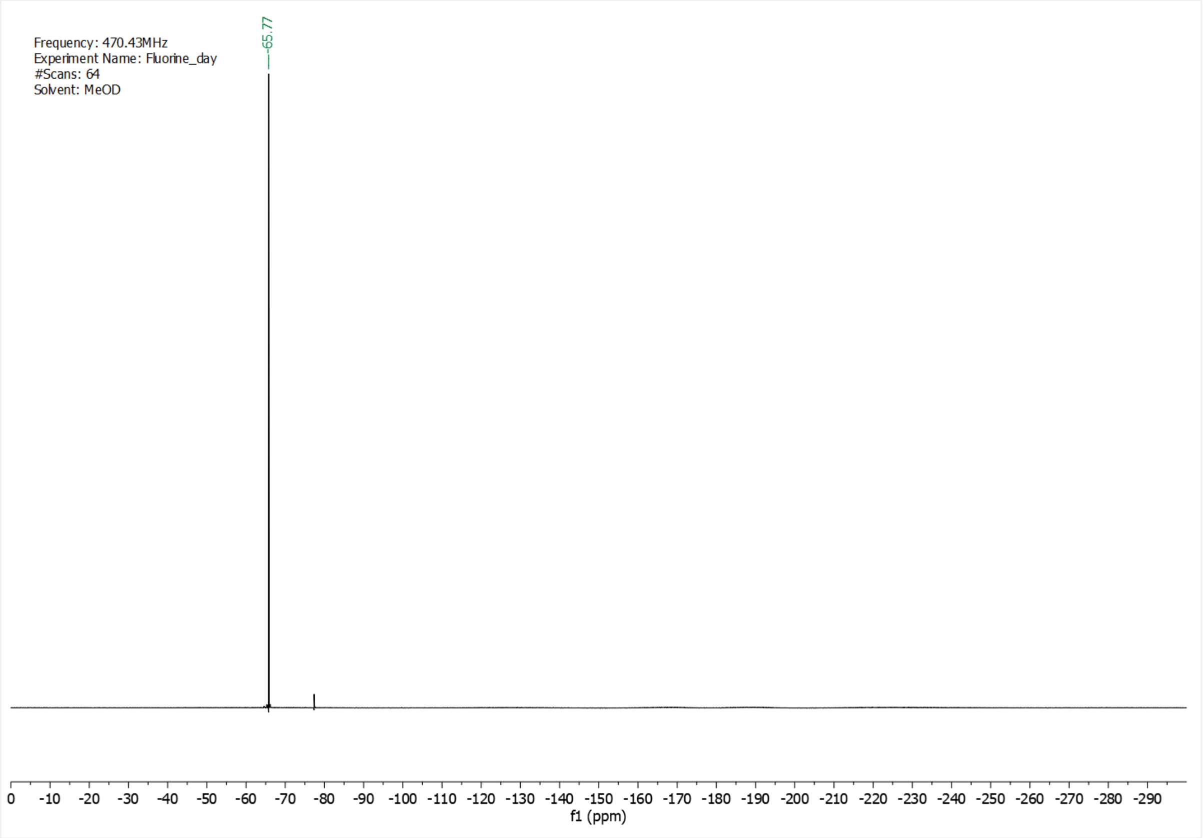
Frequency: 470.43MHz
Experiment Name: Fluorine_day
#Scans: 64
Solvent: MeOD
-65.77
f1 (ppm)

4-Iodo-2,6-bis((4-methylphenyl)sulfonamido)benzoic acid (2v)

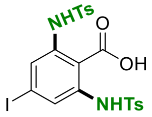

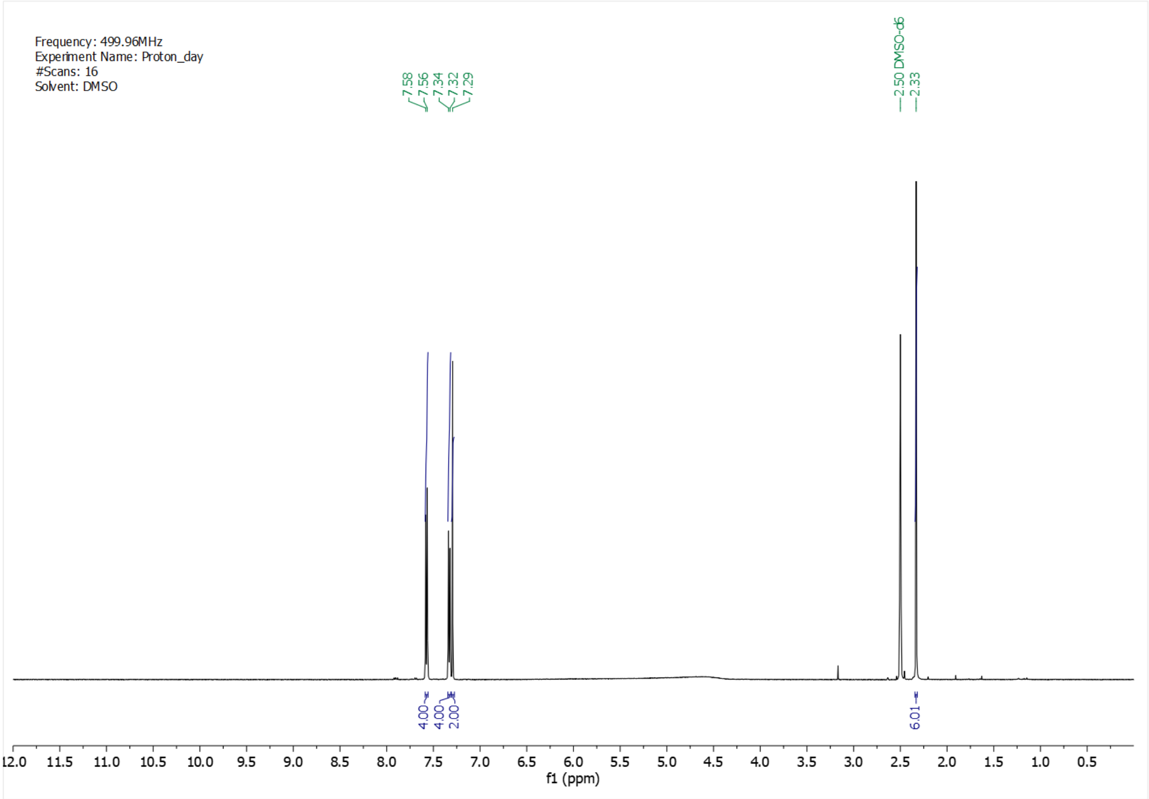

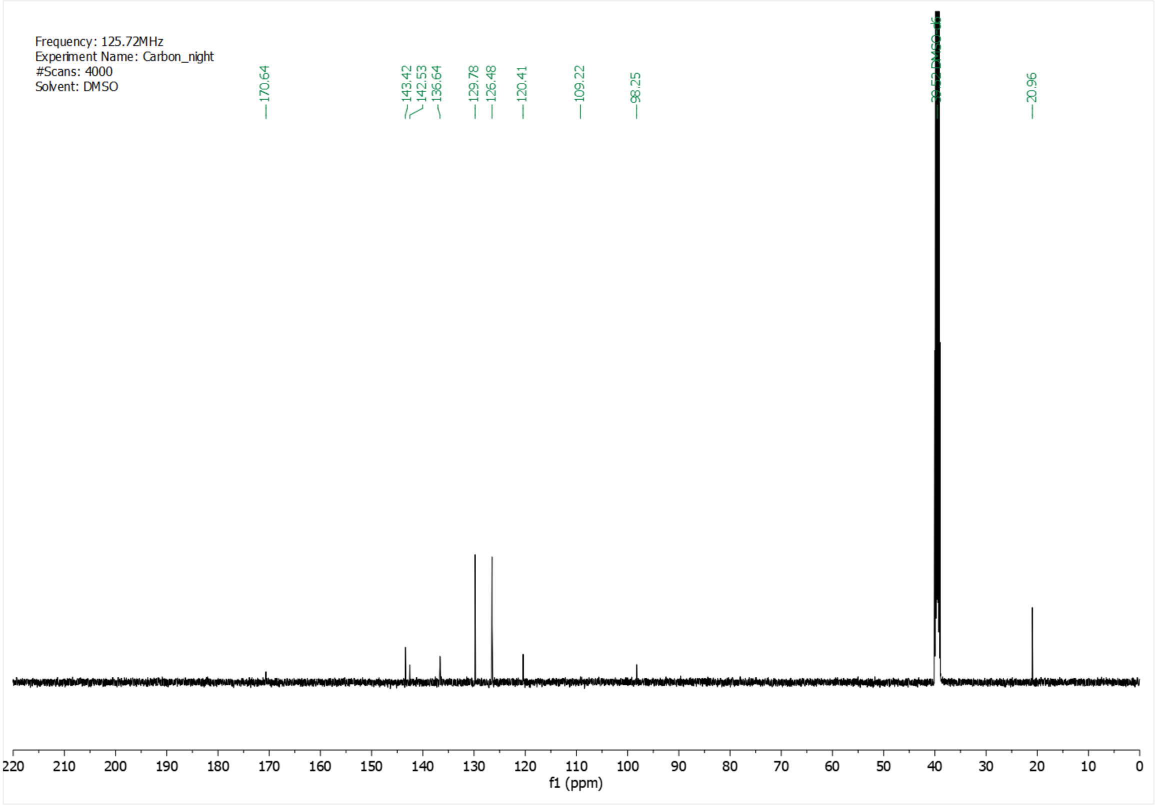

3-Methyl-7-((4-methylphenyl)sulfonamido)-4-oxo-2-phenyl-4H-chromene-8-carboxylic acid (2w)

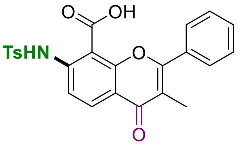

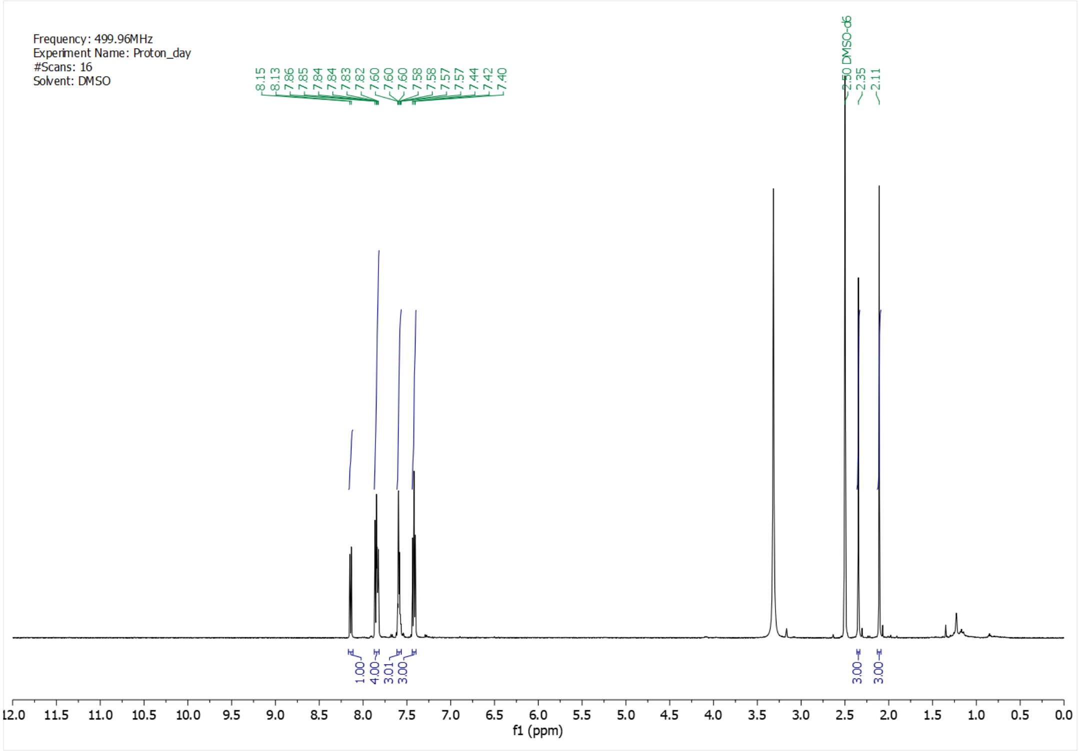

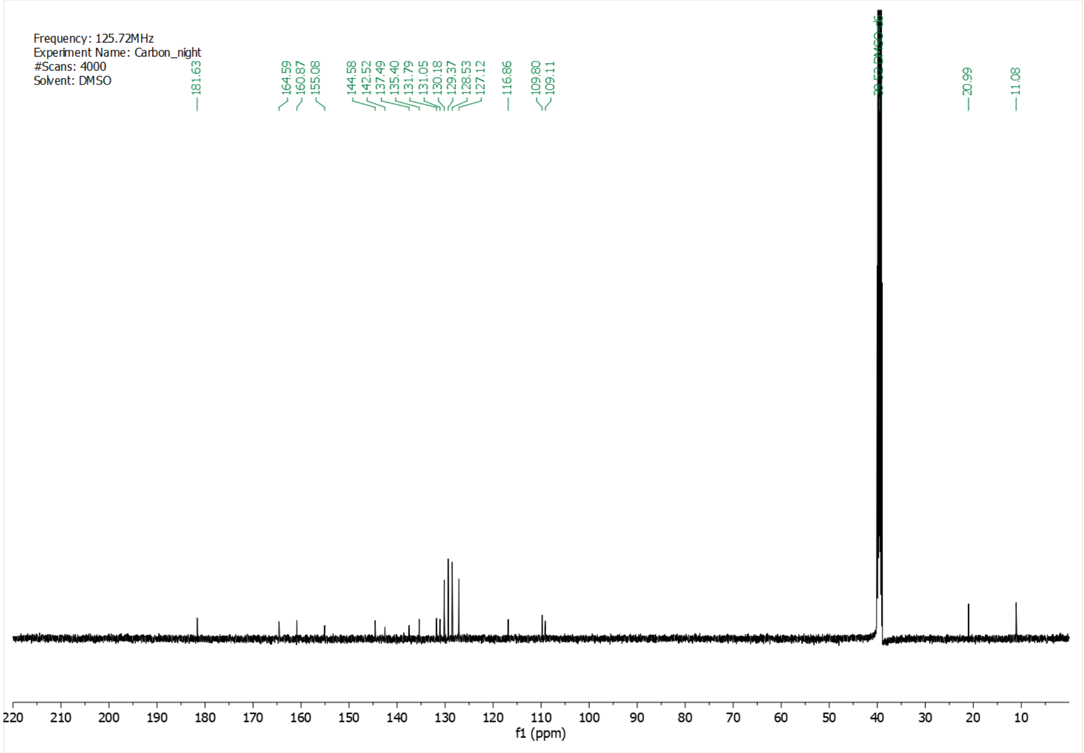

***N*-(3',6'-Dihydroxy-3-oxo-3H-spiro[isobenzofuran-1,9'-xanthen]-4-yl)-4-methylbenzenesulfonamide (2x)**

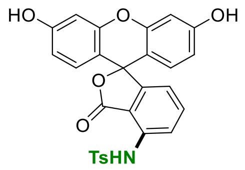

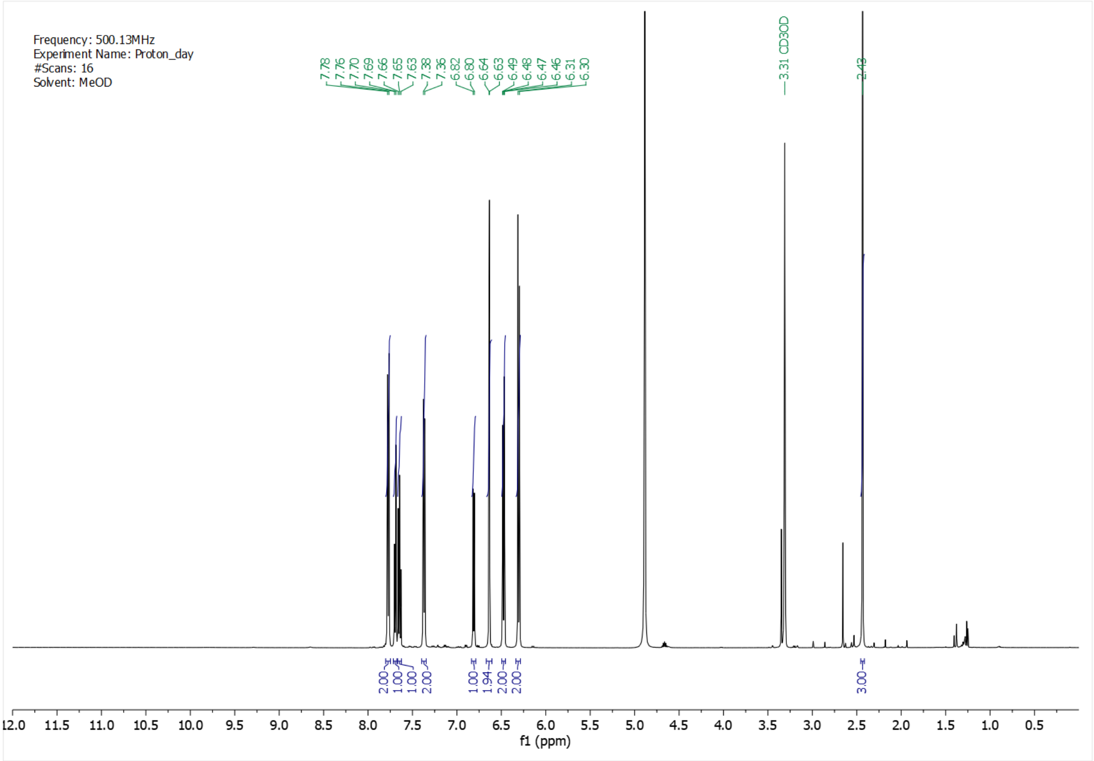

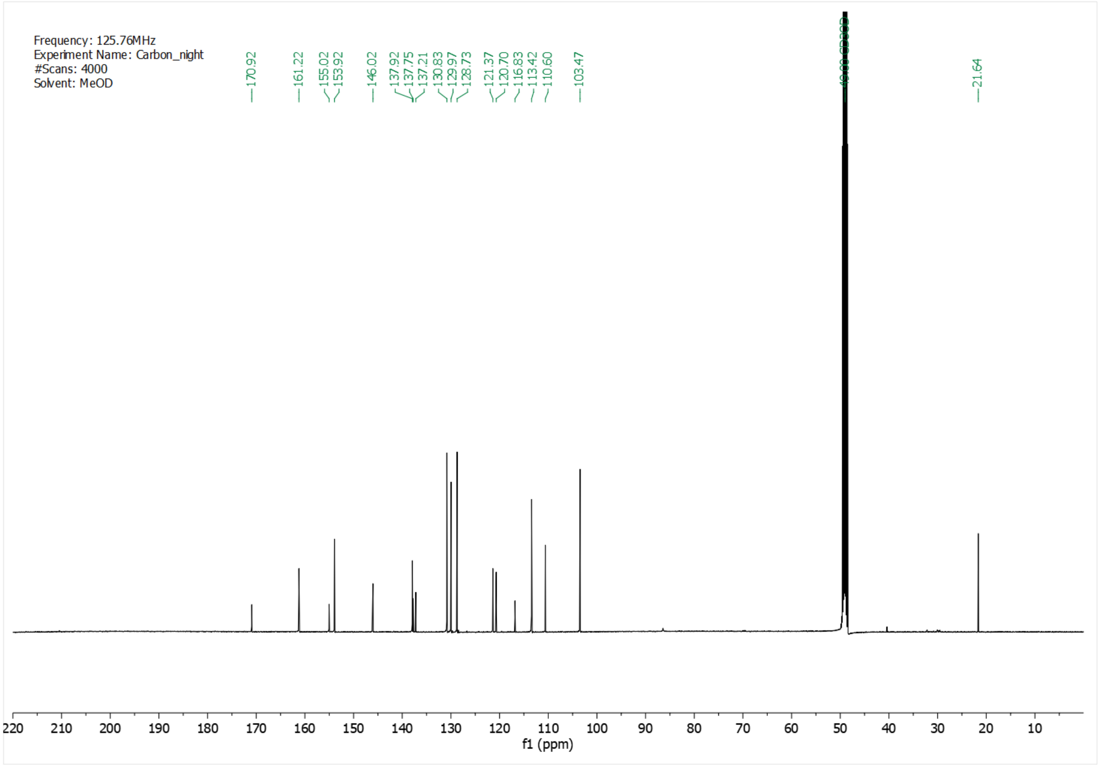

(*E*)-2-(3-(3,4-Dimethoxyphenyl)acrylamido)-6-((4-methylphenyl)sulfonamido)benzoic acid (2y)

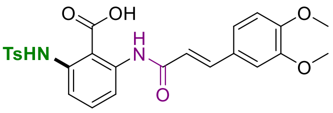

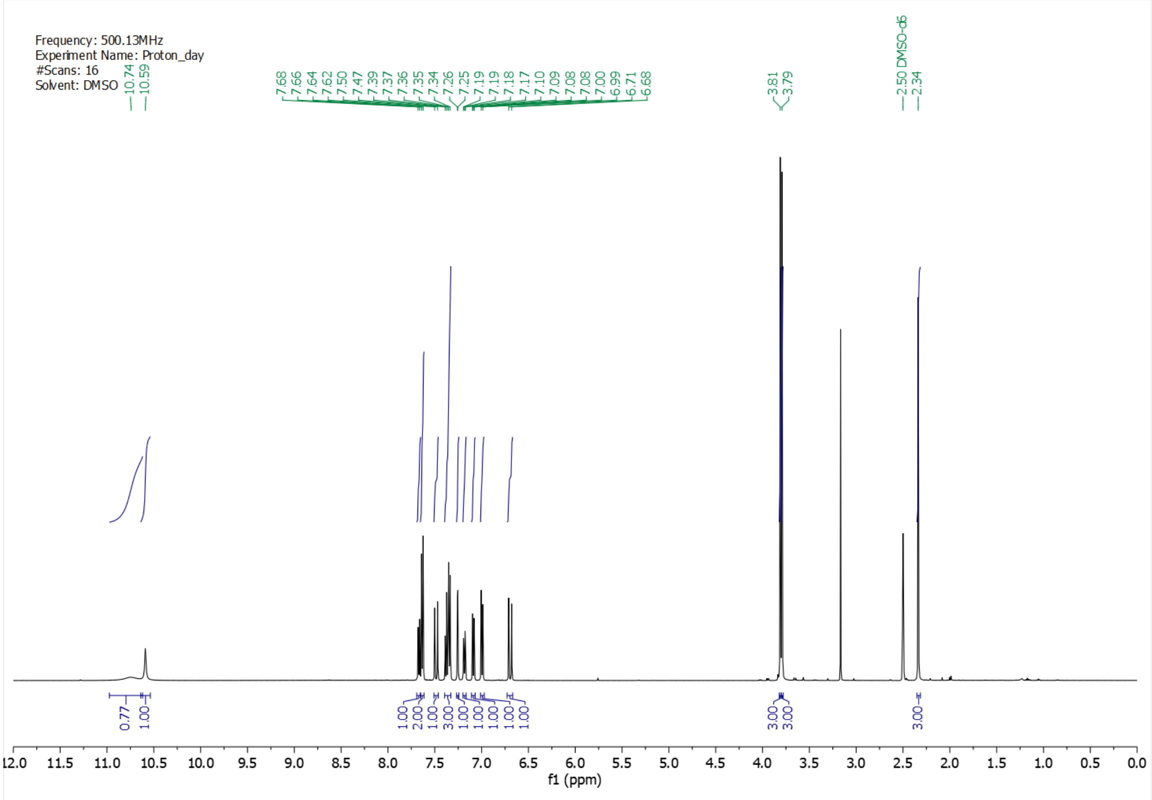

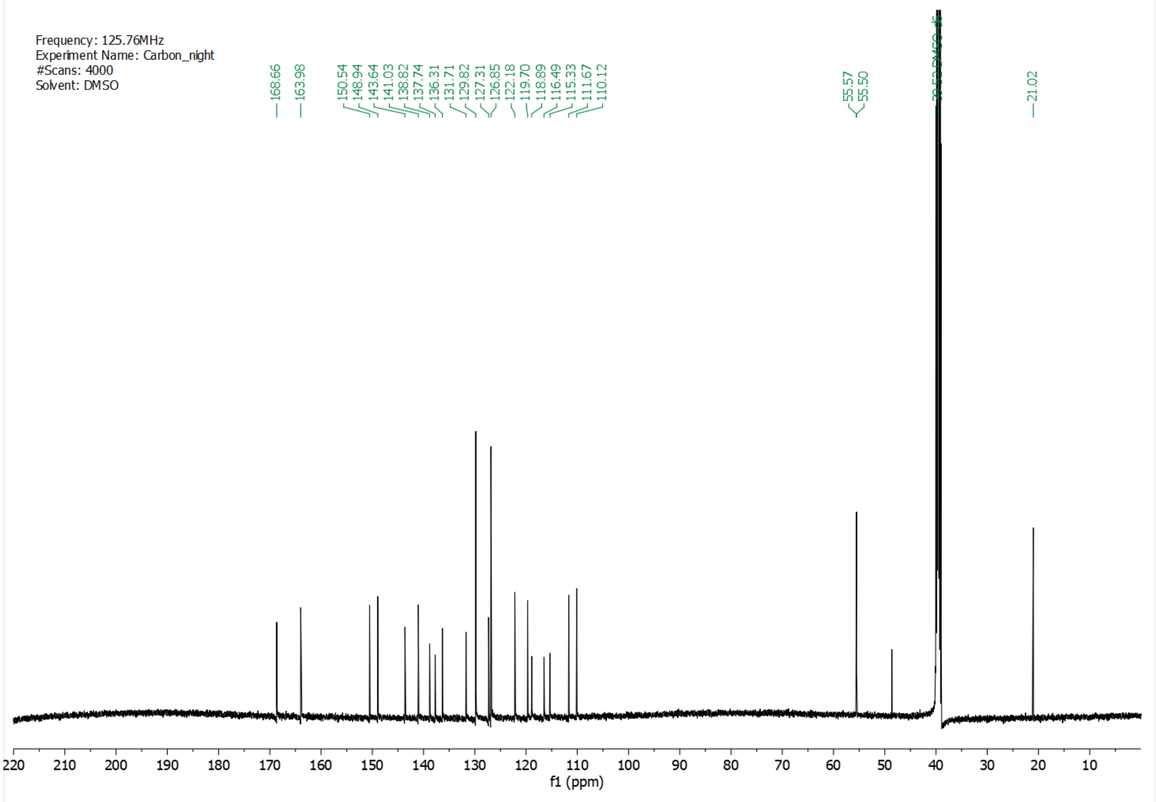

2,6-Bis((4-methylphenyl)sulfonamido)-4-(1-(3,5,5,8,8-pentamethyl-5,6,7,8-tetrahydronaphthalen-2-yl)vinyl)benzoic acid (2z)

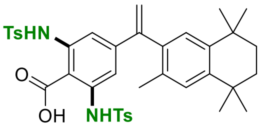

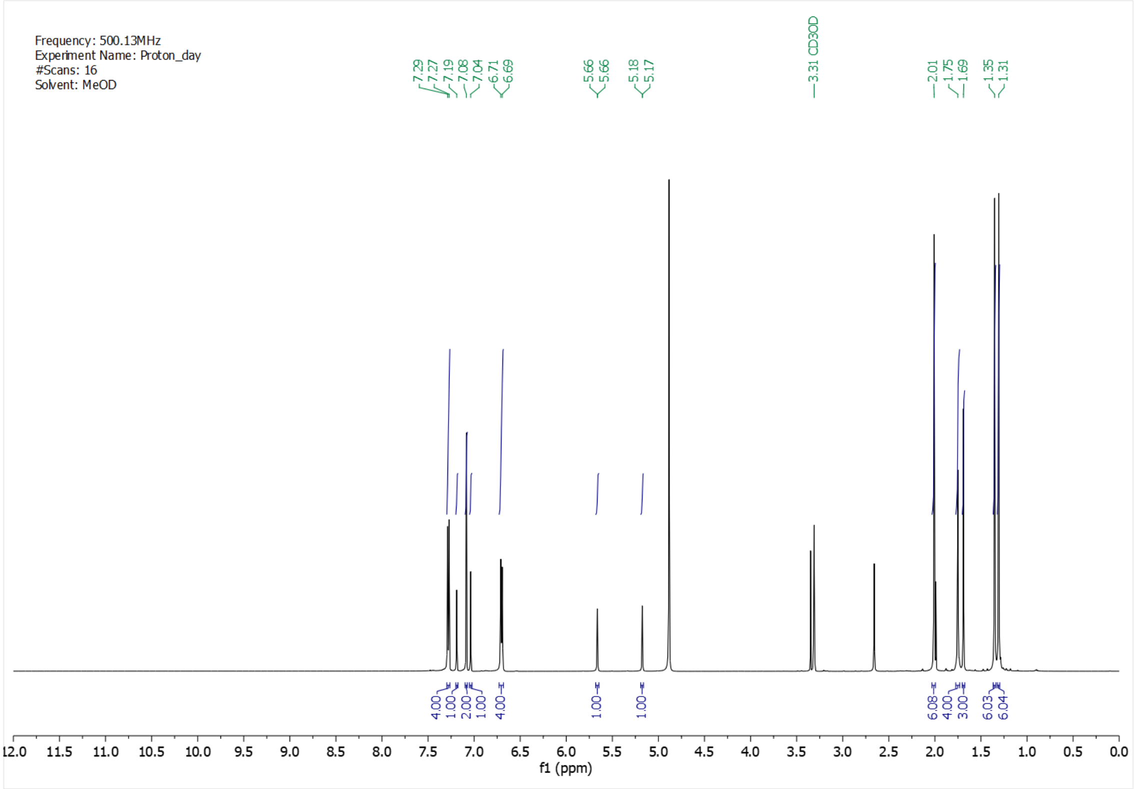

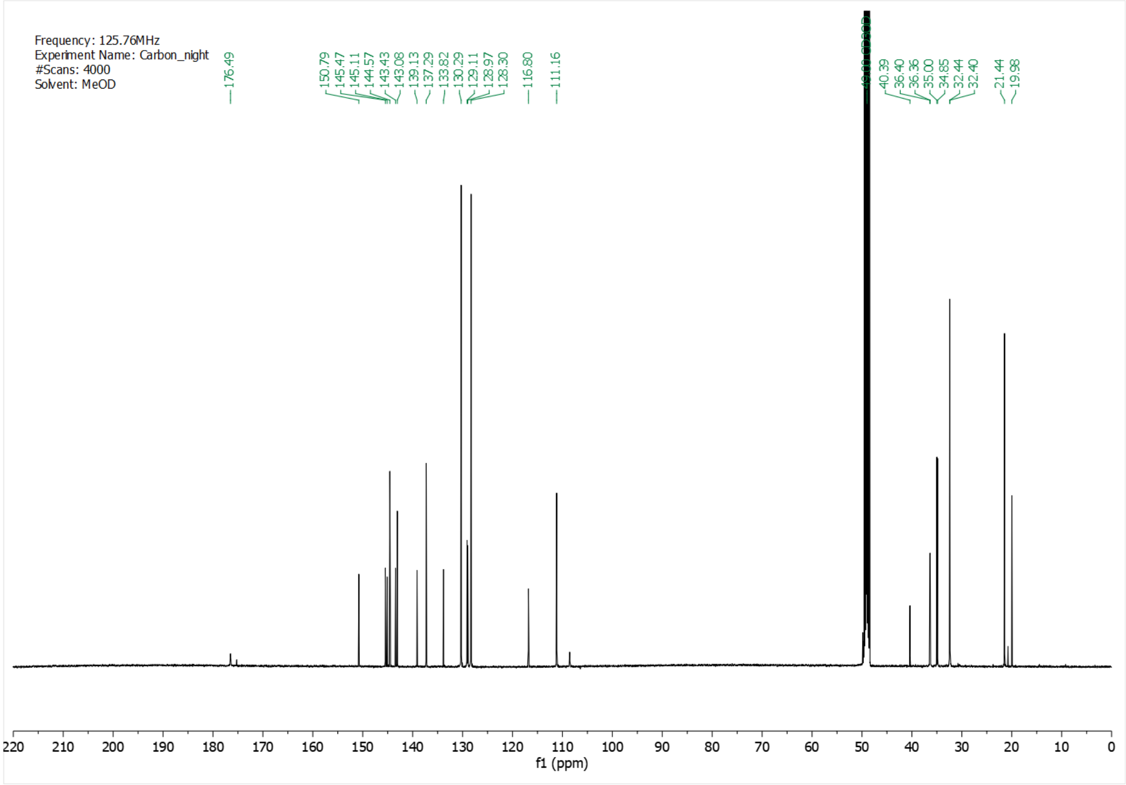

5-(5-(2-Fluorophenyl)-1,2,4-oxadiazol-3-yl)-2-((4-methylphenyl)sulfonamido)benzoic acid (2aa)

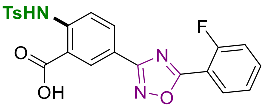

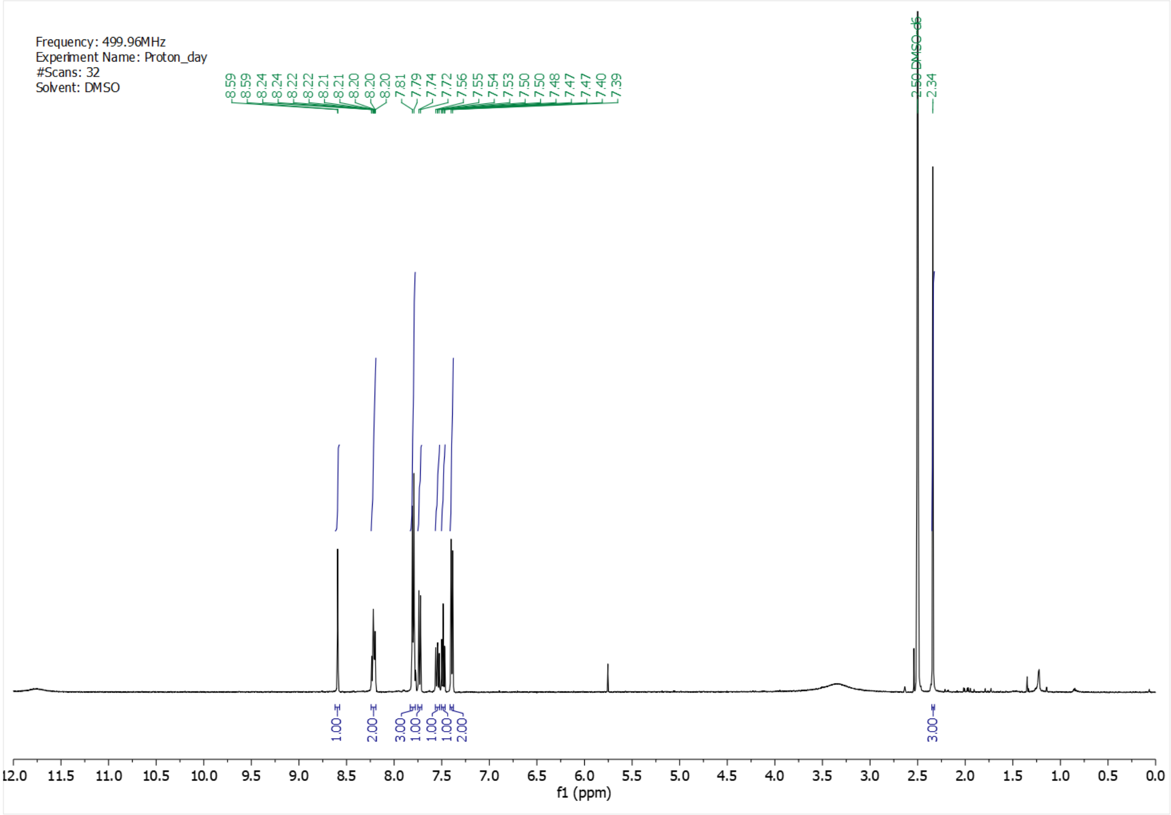

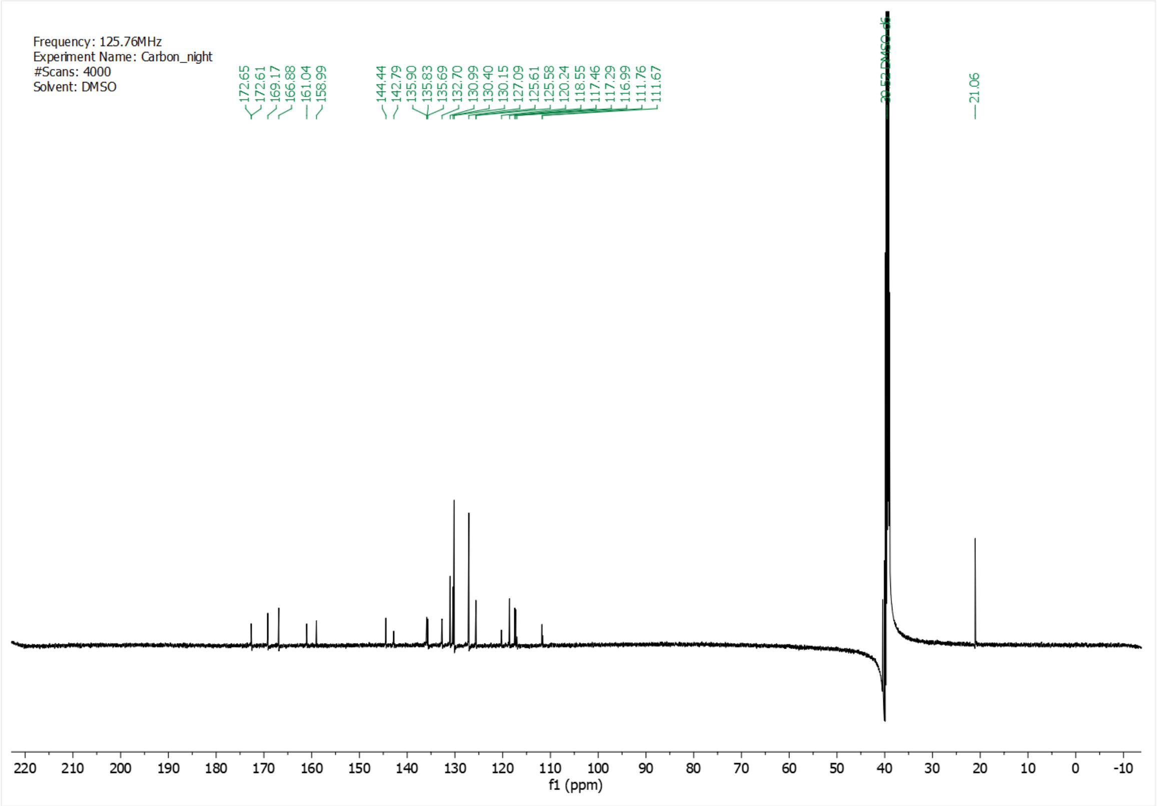

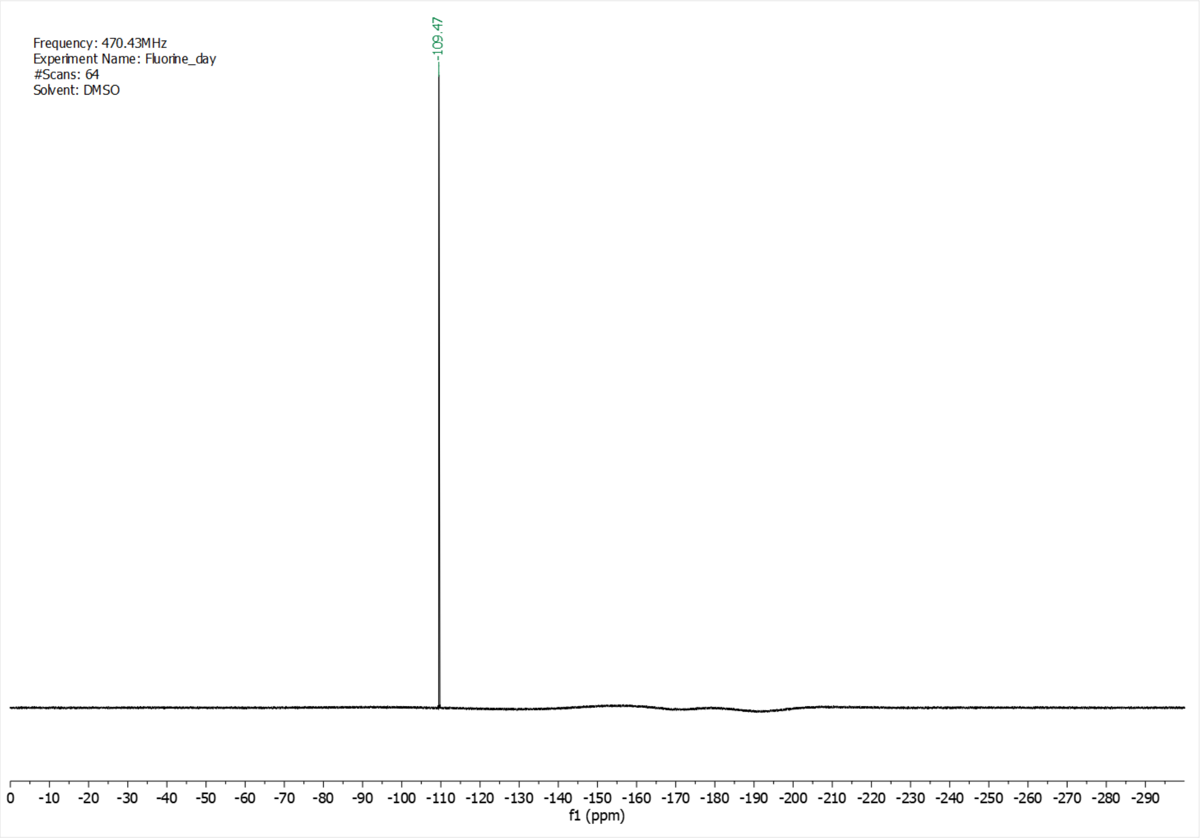
Frequency: 470.43MHz
Experiment Name: Fluorine_day
#Scans: 64
Solvent: DMSO
-109.47
0 -10 -20 -30 -40 -50 -60 -70 -80 -90 -100 -110 -120 -130 -140 -150 -160 -170 -180 -190 -200 -210 -220 -230 -240 -250 -260 -270 -280 -290
f1 (ppm)

5-(6-(1-(2,2-Difluorobenzo[d][1,3]dioxol-5-yl)cyclopropane-1-carboxamido)-3-methylpyridin-2-yl)-2-((4-methylphenyl)sulfonamido)benzoic acid (2ab)

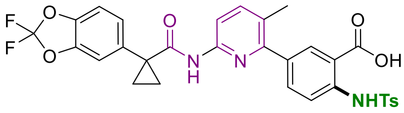

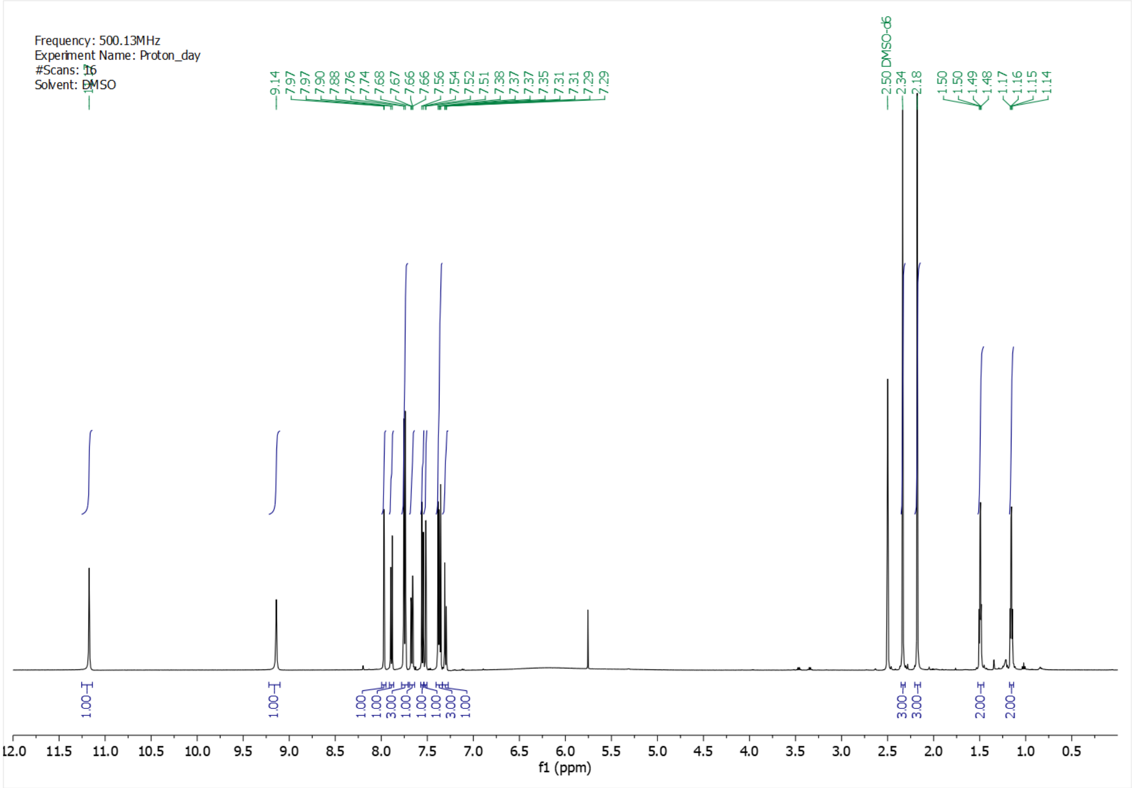

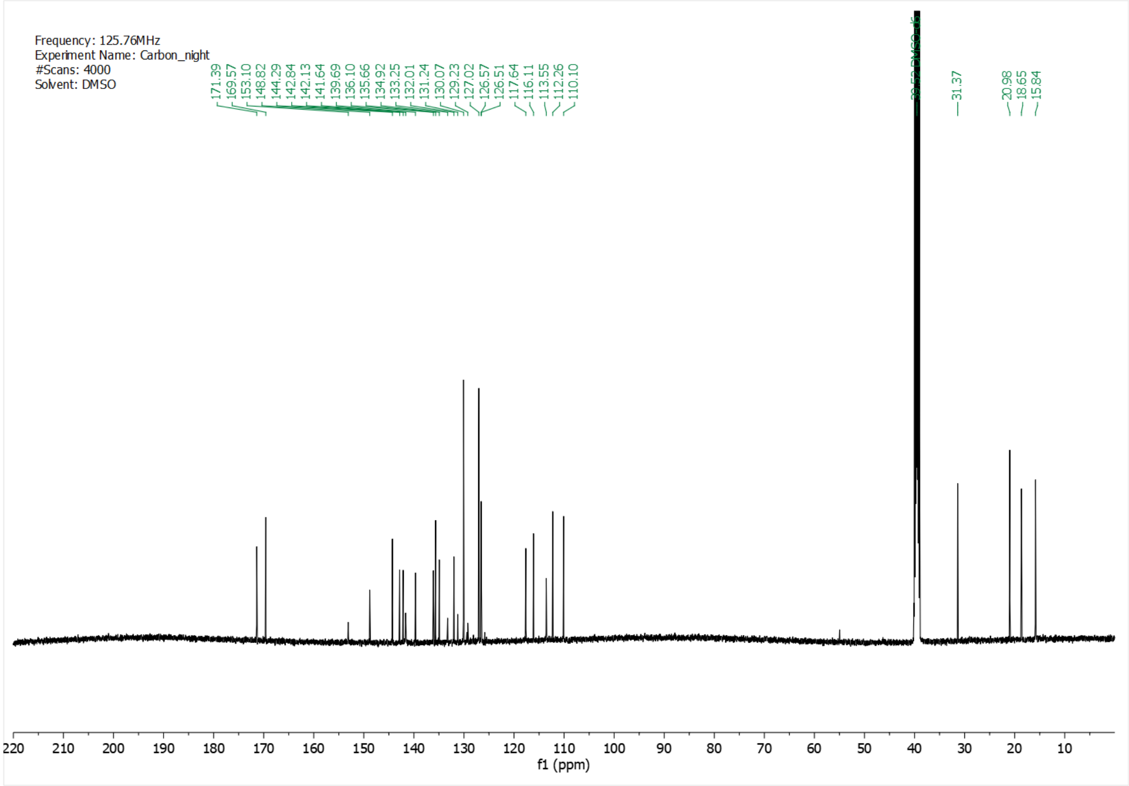

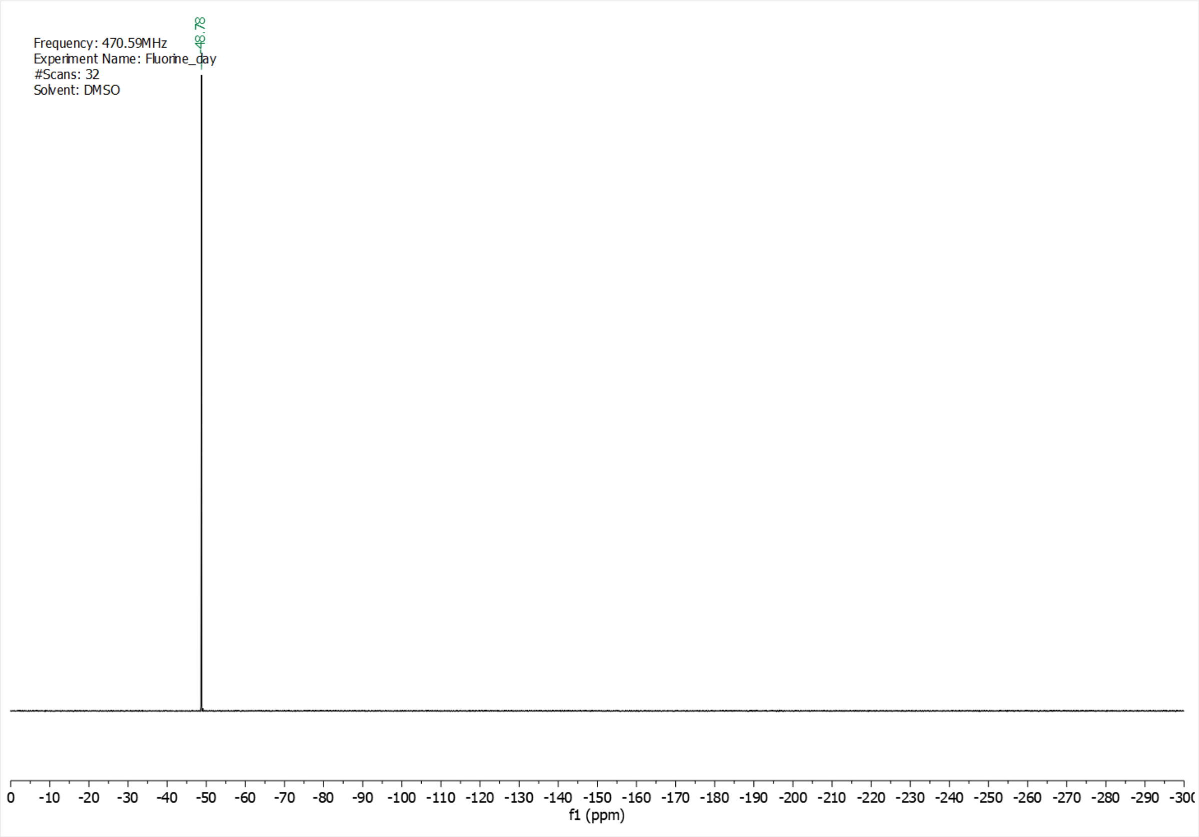
-48.78
Frequency: 470.59MHz
Experiment Name: Fluorine_day
#Scans: 32
Solvent: DMSO
0 -10 -20 -30 -40 -50 -60 -70 -80 -90 -100 -110 -120 -130 -140 -150 -160 -170 -180 -190 -200 -210 -220 -230 -240 -250 -260 -270 -280 -290 -300
f1 (ppm)

(*S*)-2-Ethoxy-4-(2-((3-methyl-1-(2-(piperidin-1-yl)phenyl)butyl)amino)-2-oxoethyl)-6-((4-methylphenyl)sulfonamido)benzoic acid (2ac)

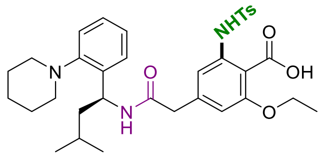

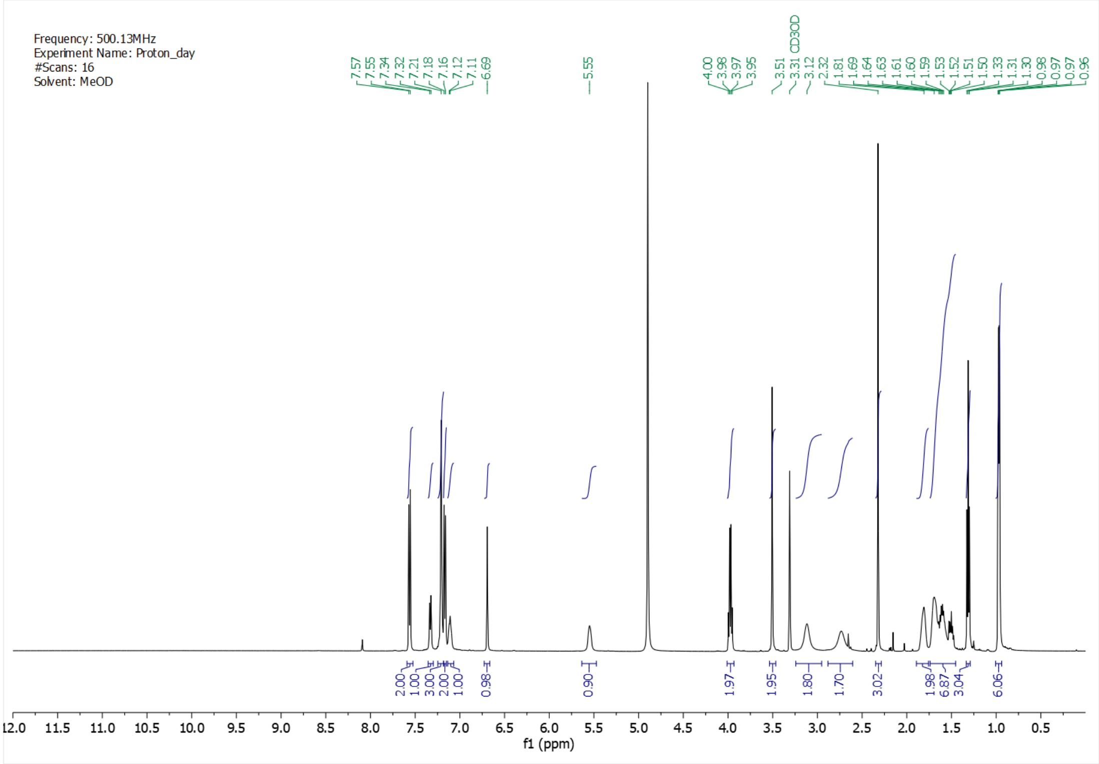

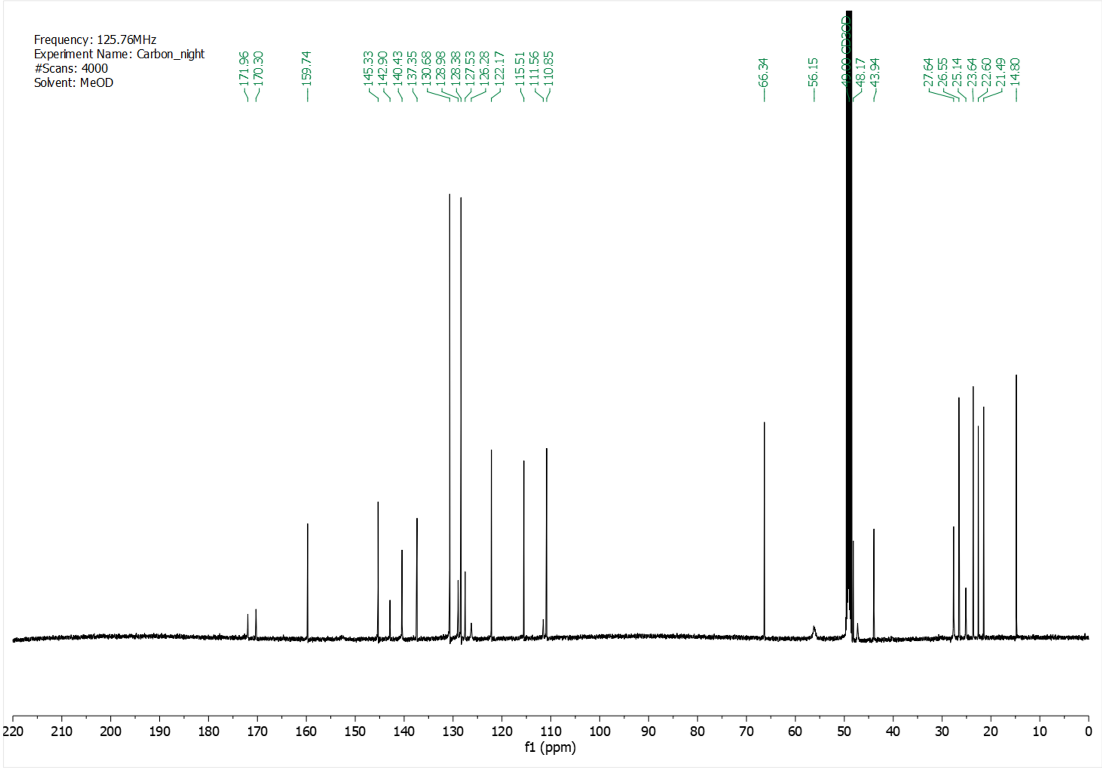

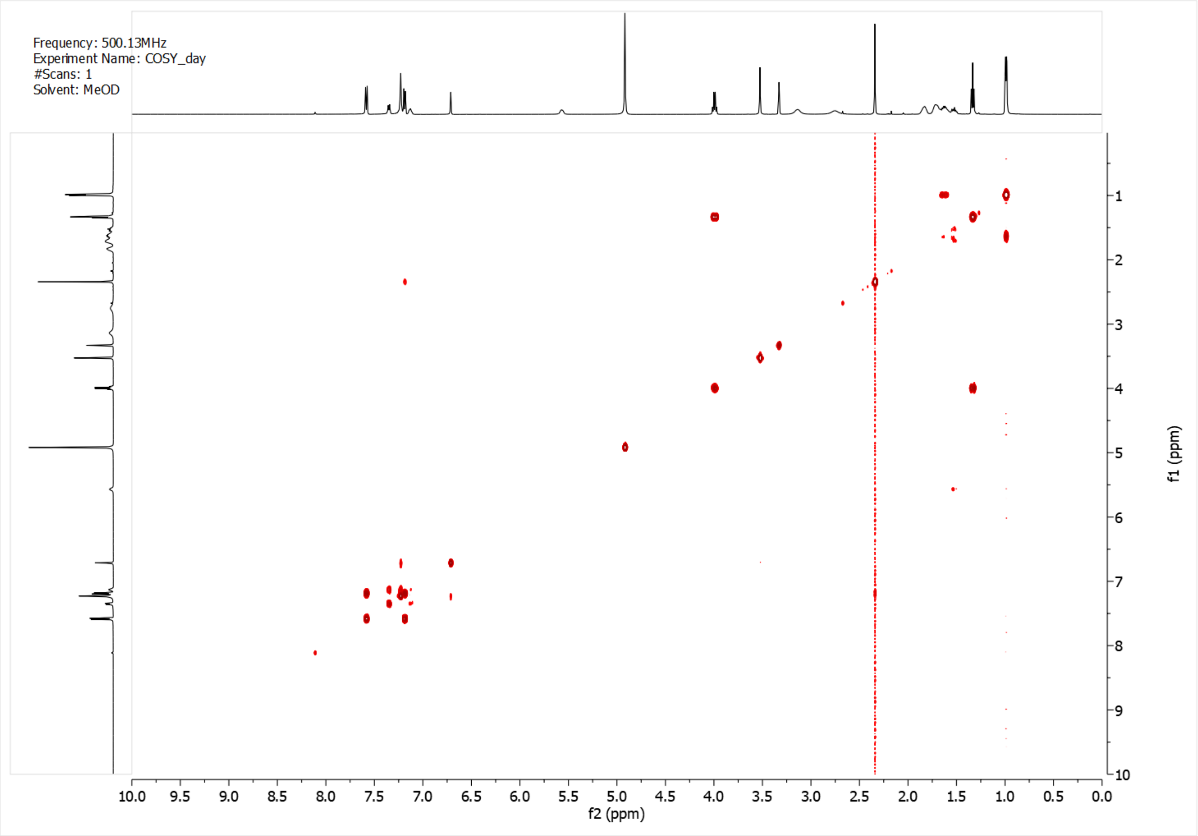
Frequency: 500.13MHz
Experiment Name: COSY_day
#Scans: 1
Solvent: MeOD
f2 (ppm)
f1 (ppm)

(*E*)-3-(3,4-dimethoxyphenyl)-*N*-(3-((4-methylphenyl)sulfonamido)phenyl)acrylamide (2ya)

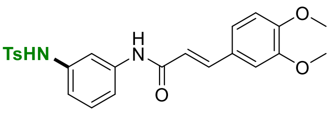

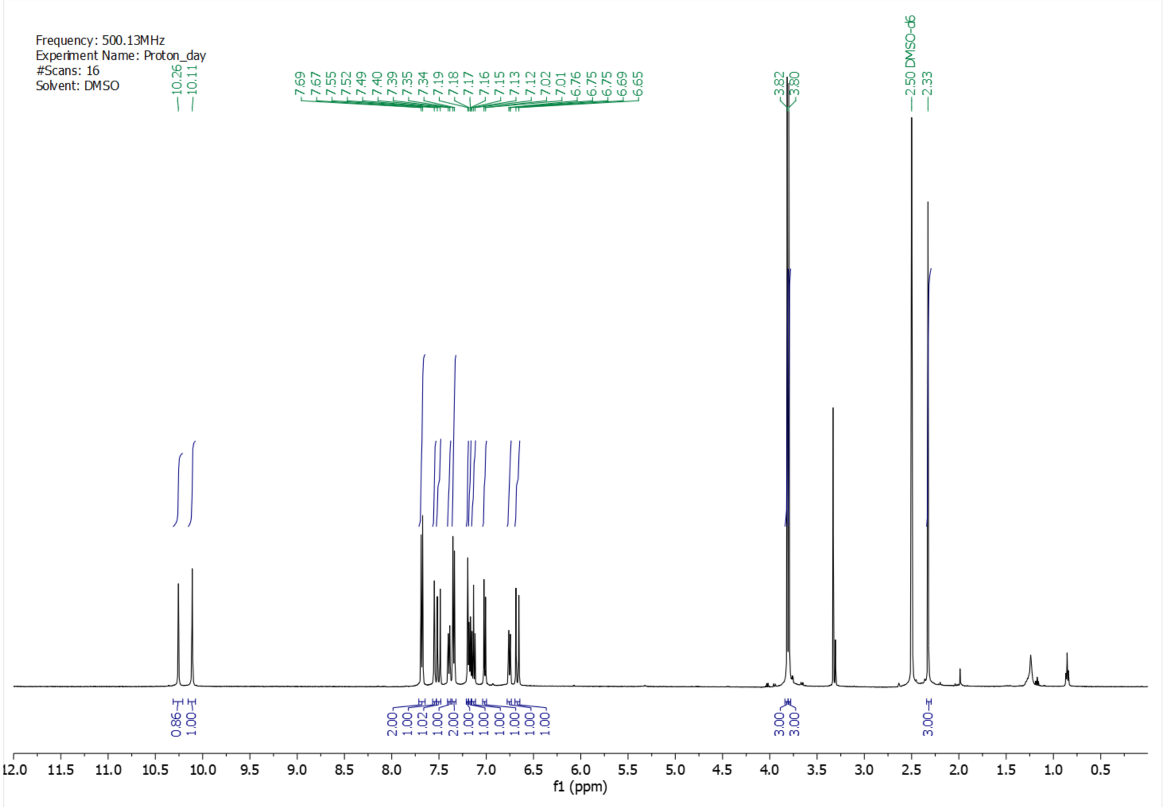

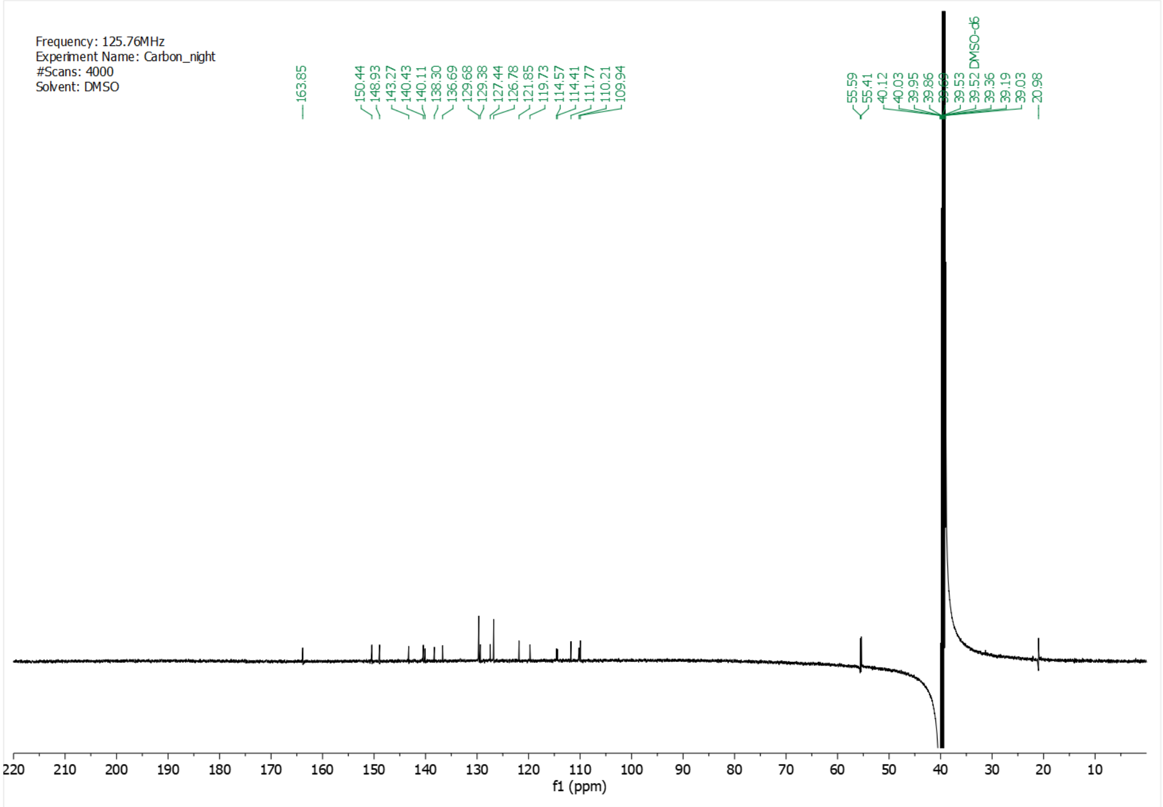

(*E*)-*N*-(2-(3,4-dimethoxystyryl)-4-oxo-4H-benzo[d][1,3]oxazin-5-yl)-4-methylbenzenesulfonamide (2yb)

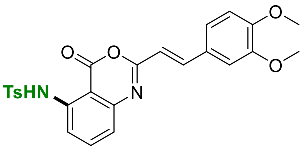

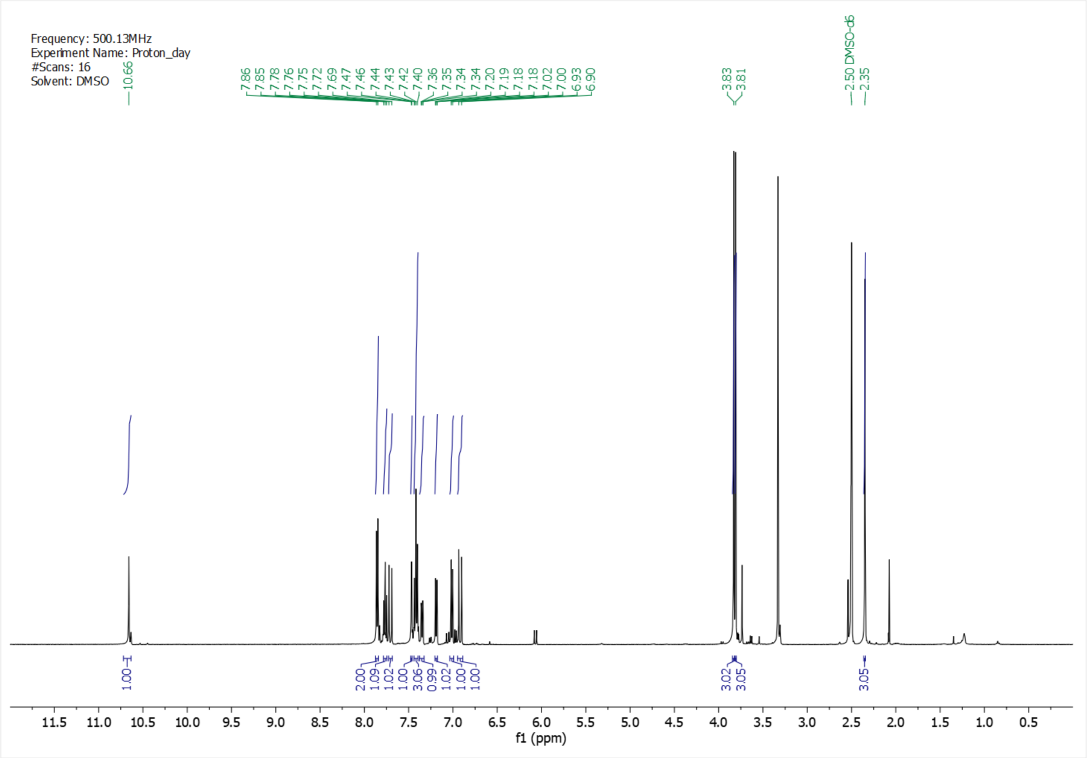

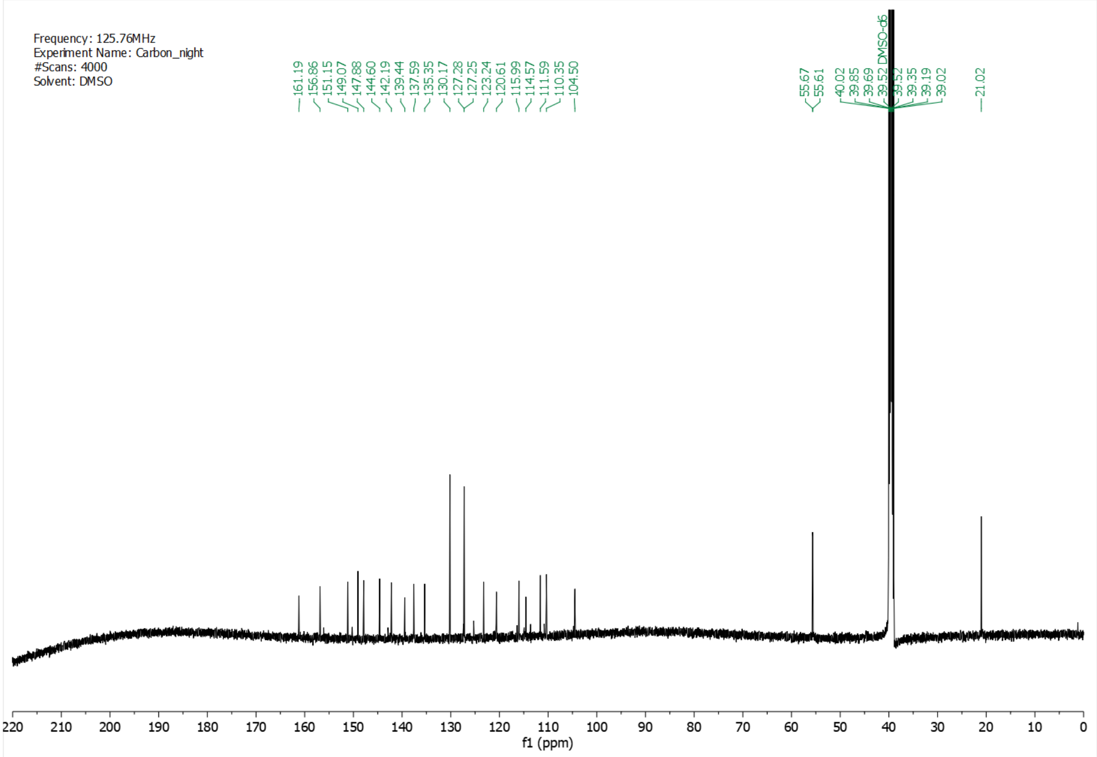

7-Amino-3-methyl-4-oxo-2-phenyl-4H-chromene-8-carboxylic acid (3a)

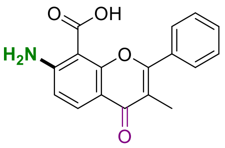

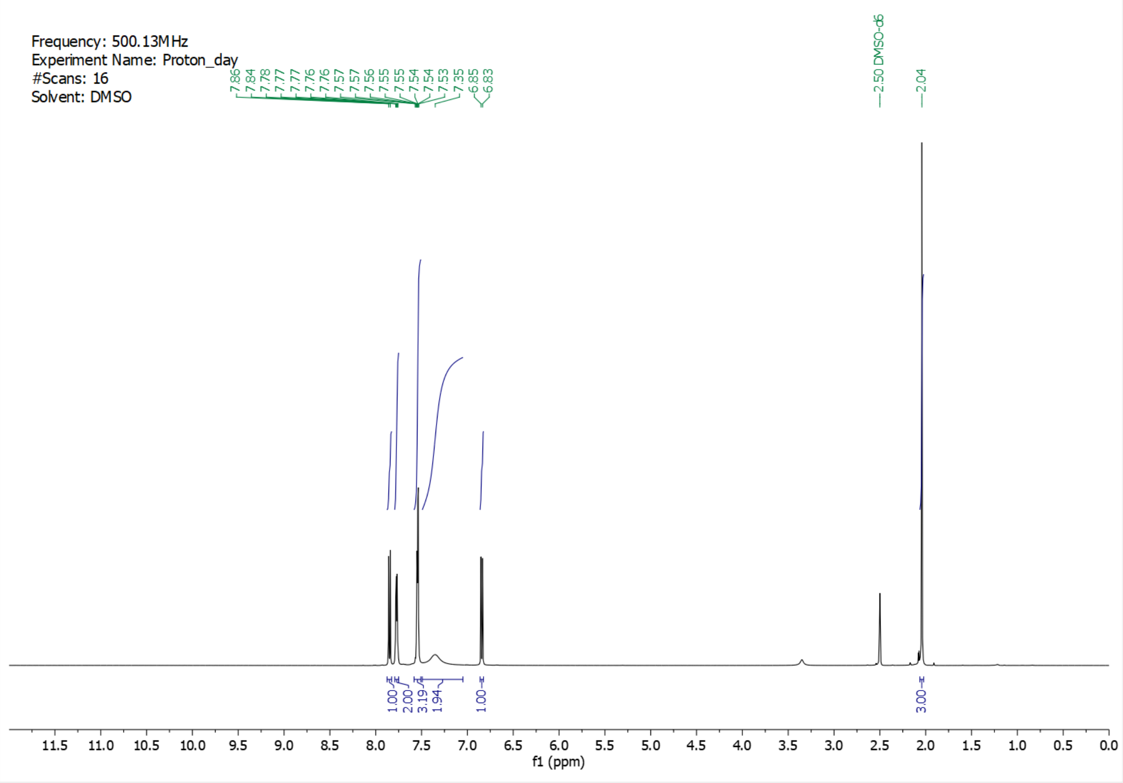

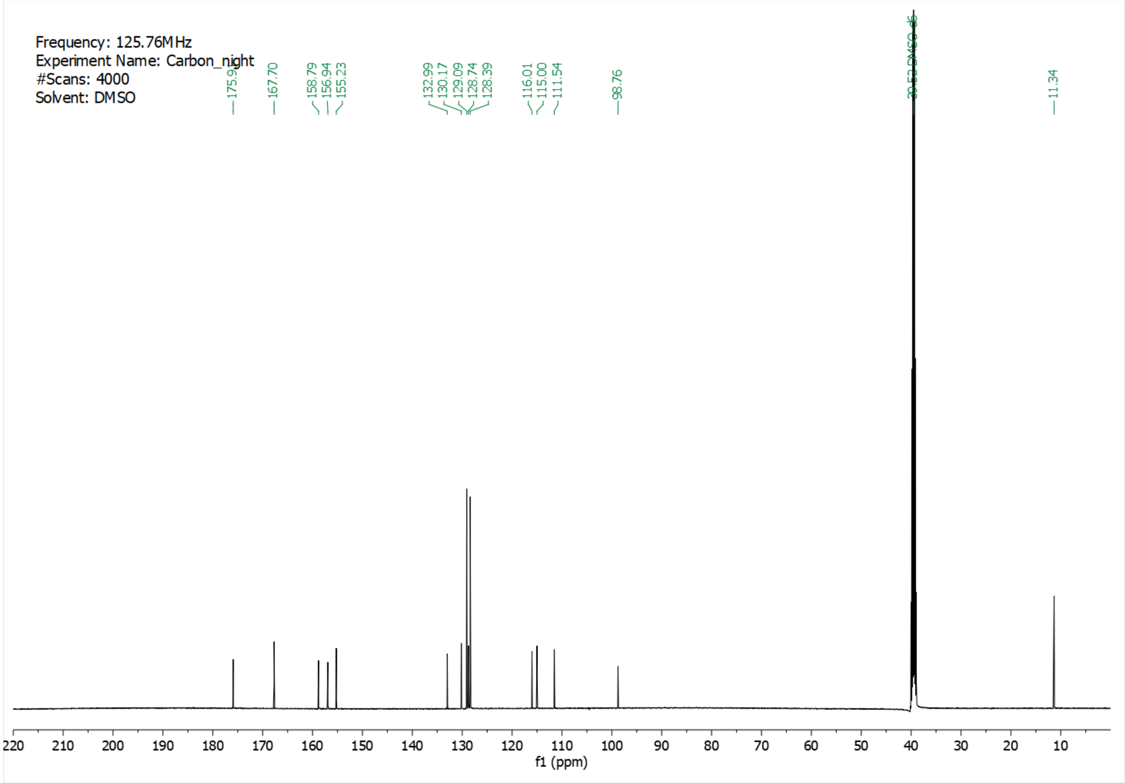

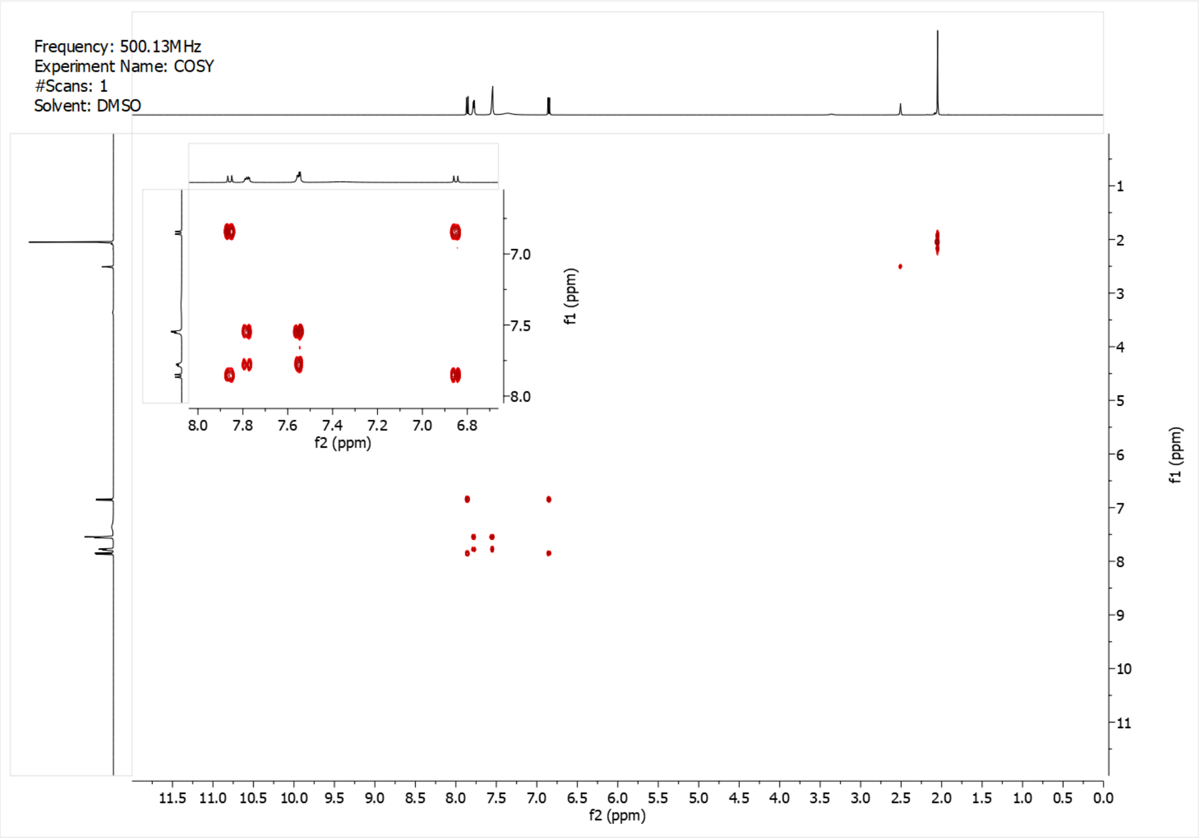

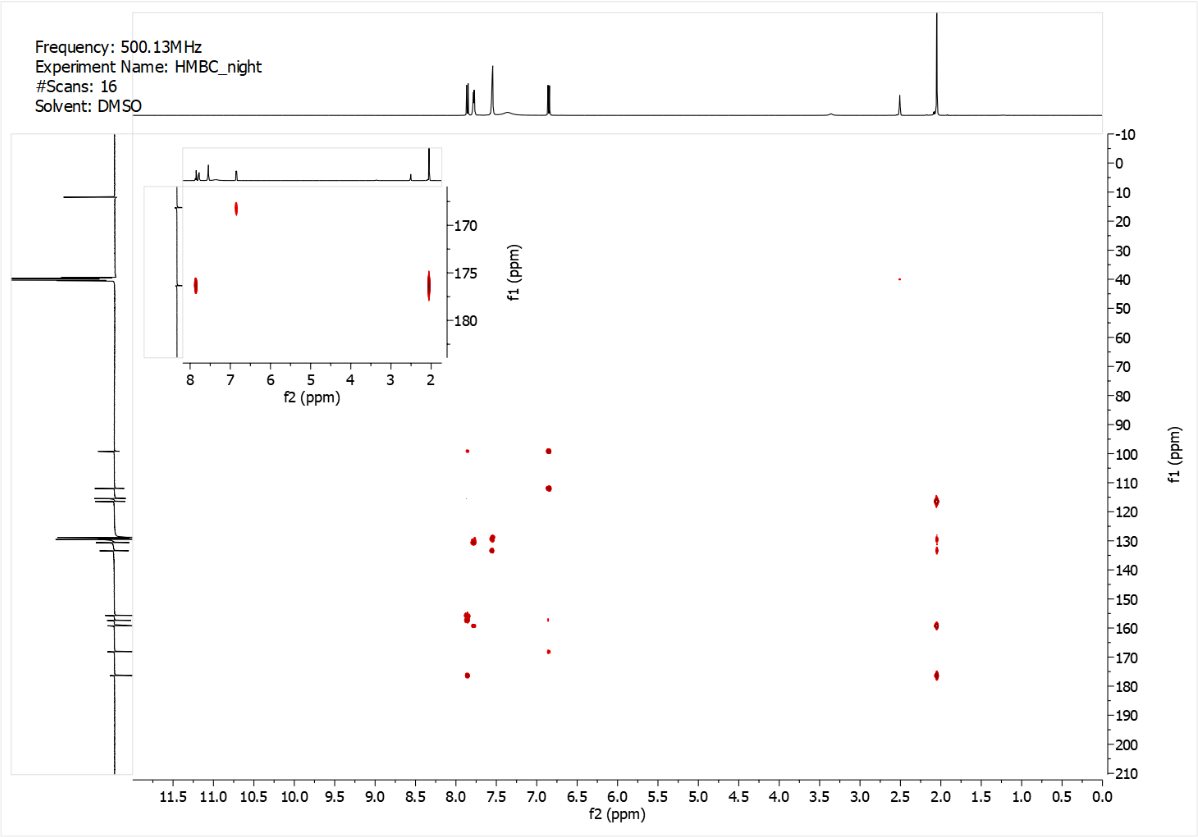

HMBC confirms regiochemistry. Cross-peaks between cyclic ketone carbon and methyl-H, as well as neighboring sp^2-H.

4-Amino-3',6'-dihydroxy-3H-spiro[isobenzofuran-1,9'-xanthen]-3-one (3b)

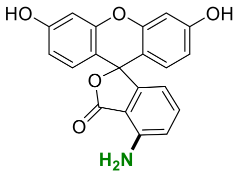

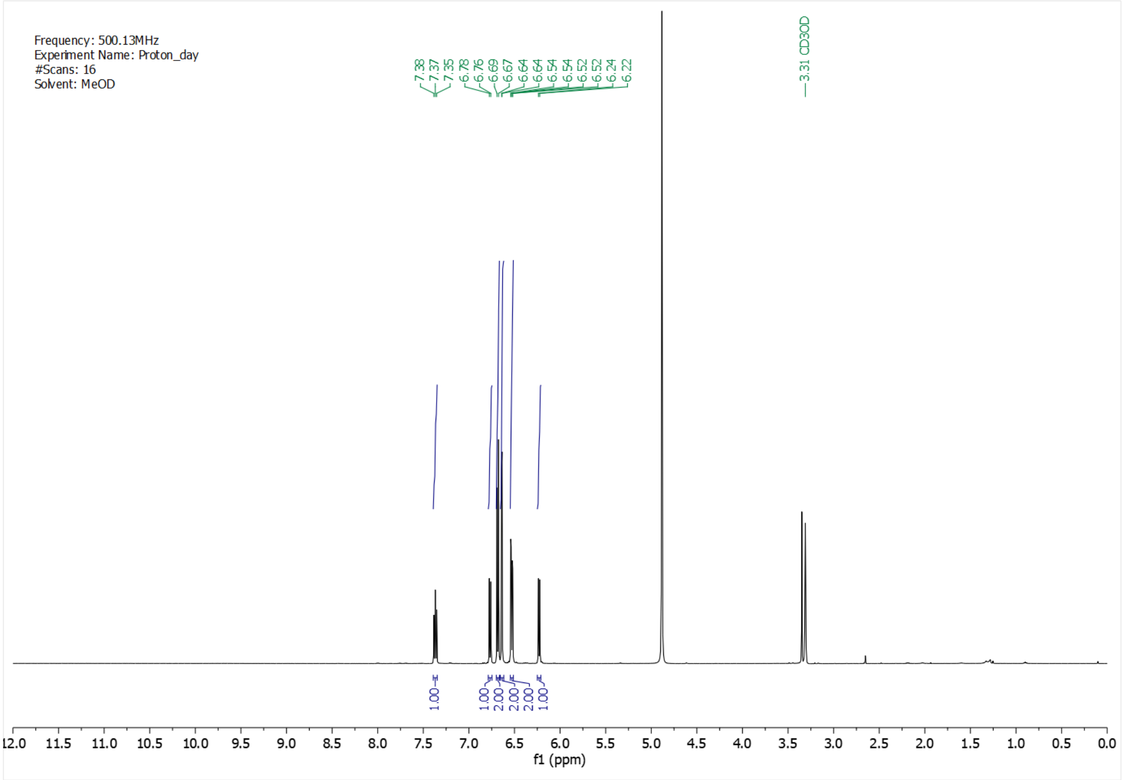

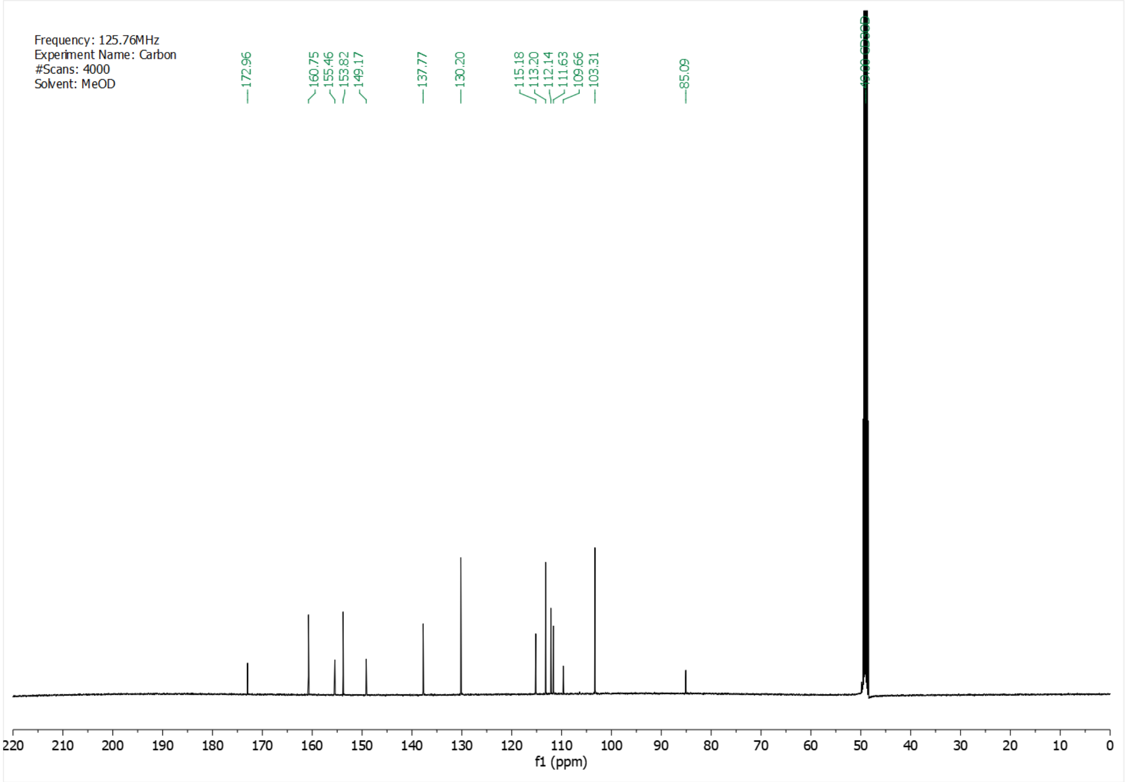

(*E*)-2-Amino-6-(3-(3,4-dimethoxyphenyl)acrylamido)benzoic acid (3c)

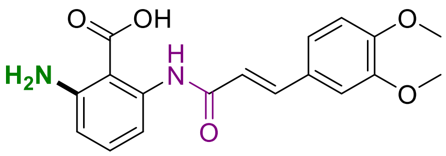

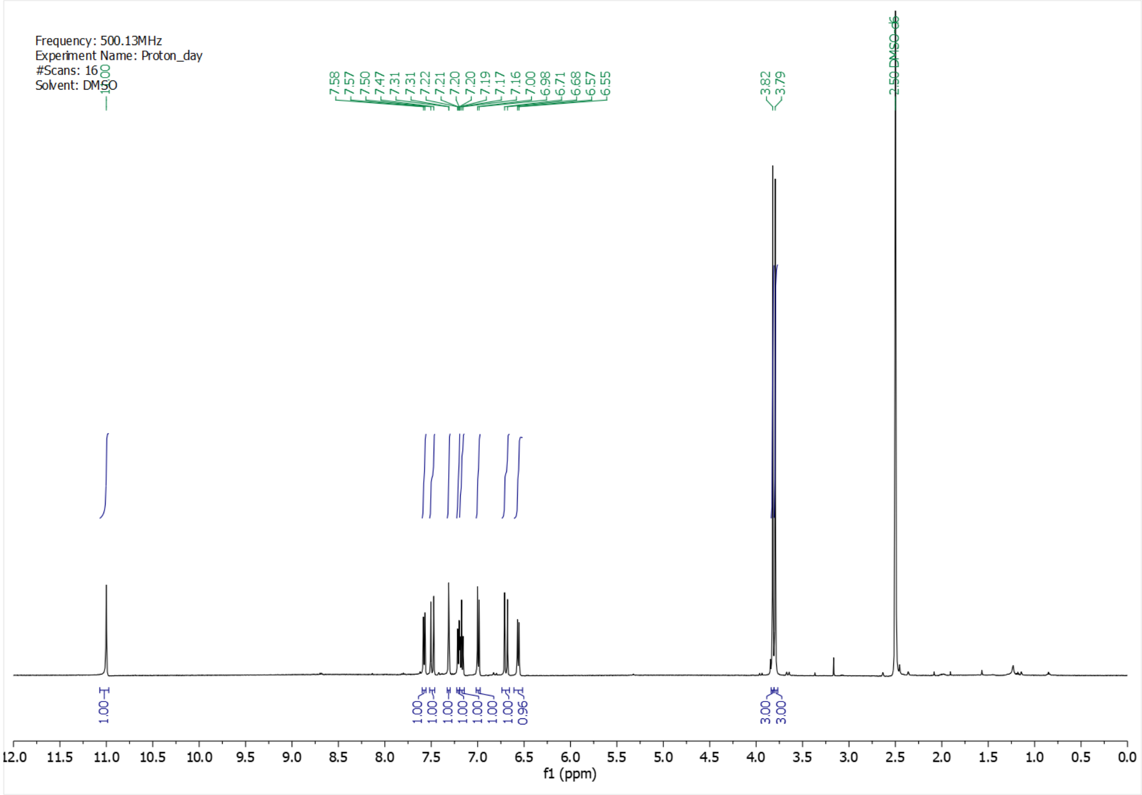

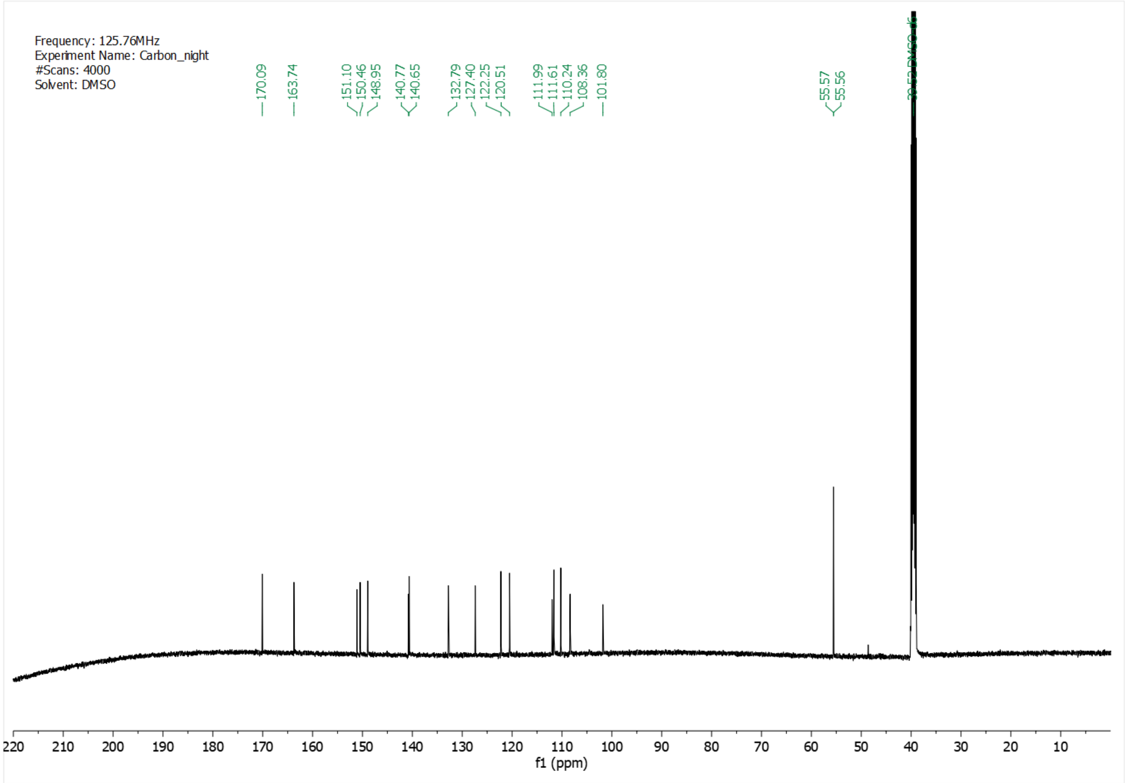

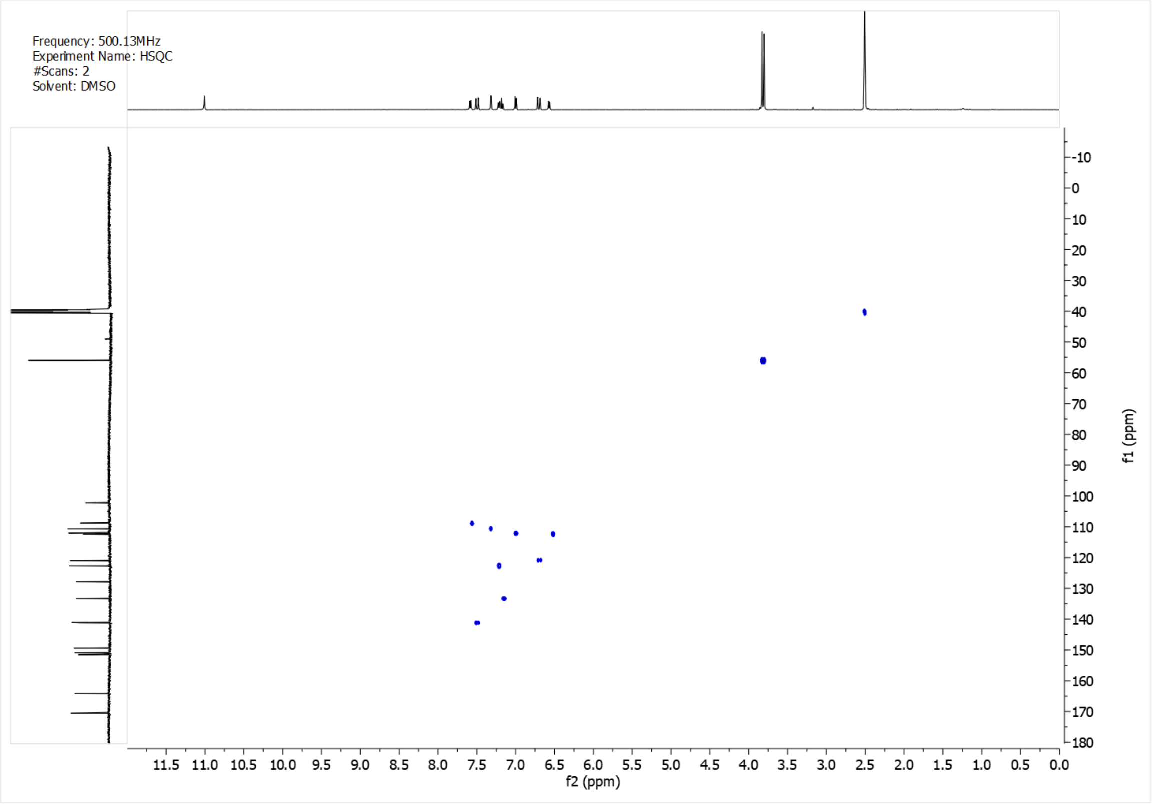

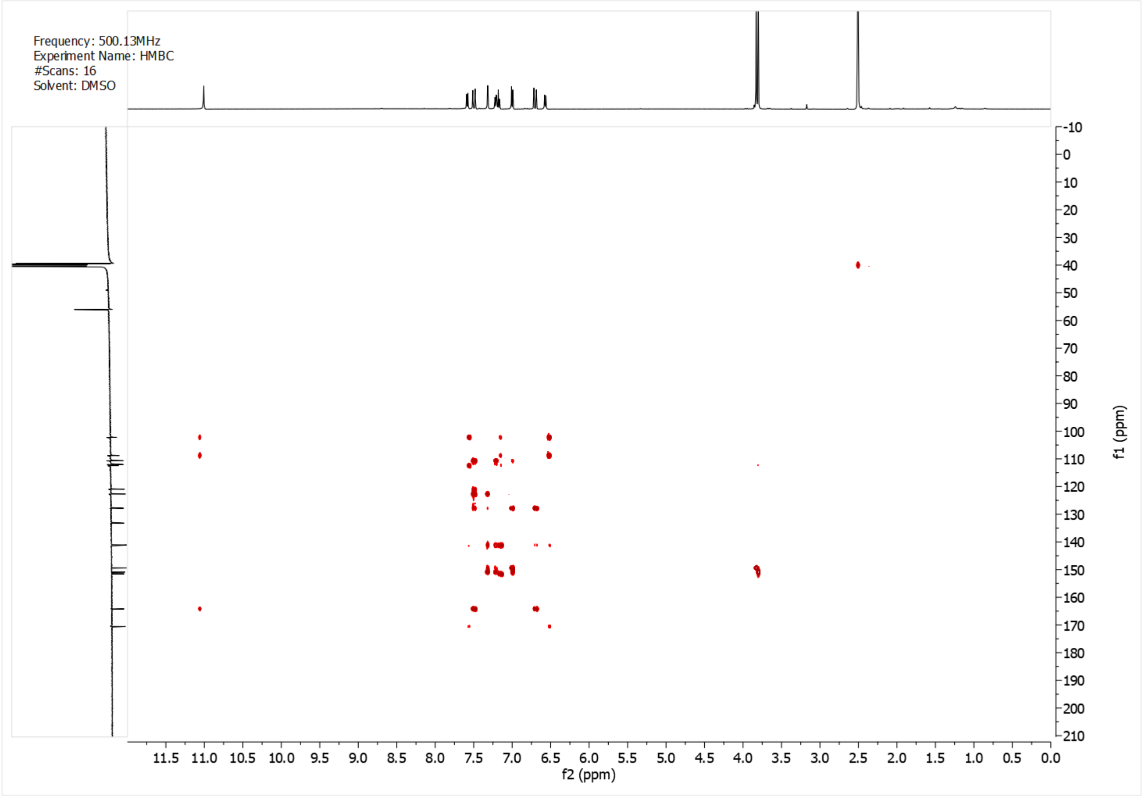

Note: Regiochemistry confirmed by HMBC. Both alkene-H cross-peaks with amide carbon observed. Benzoic acid H-3 and H-5 cross-peaks with carboxylic acid carbon observed.

2-Amino-4-(1-(3,5,5,8,8-pentamethyl-5,6,7,8-tetrahydronaphthalen-2-yl)vinyl)benzoic acid (3d)

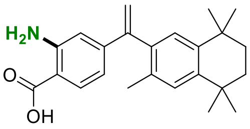

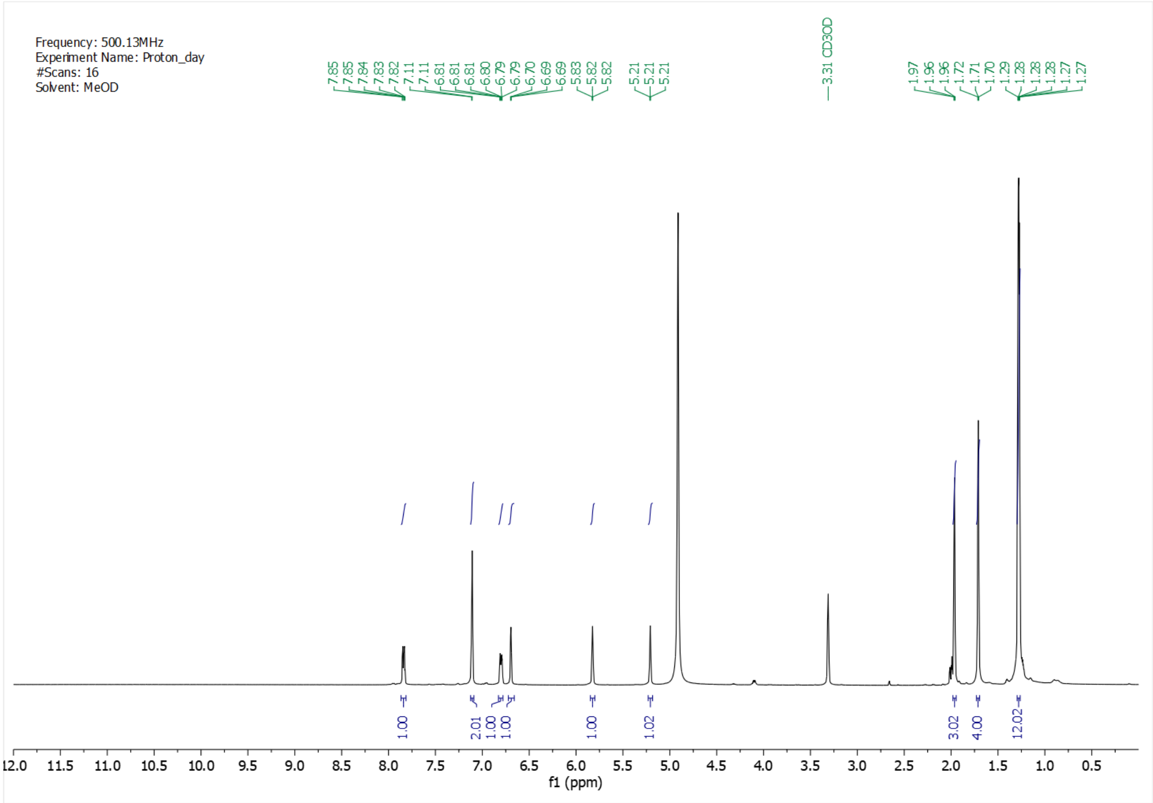

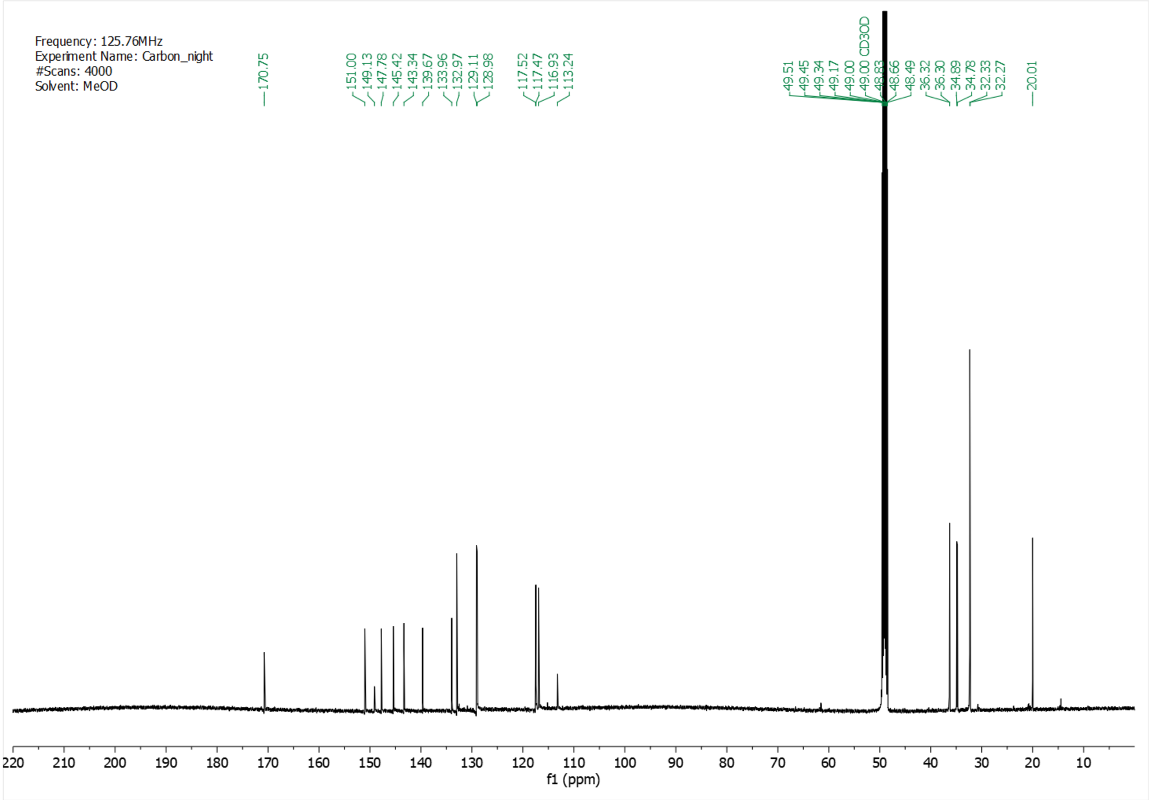

2-Amino-5-(5-(2-fluorophenyl)-1,2,4-oxadiazol-3-yl)benzoic acid (3e)

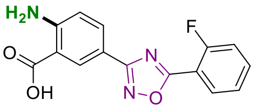

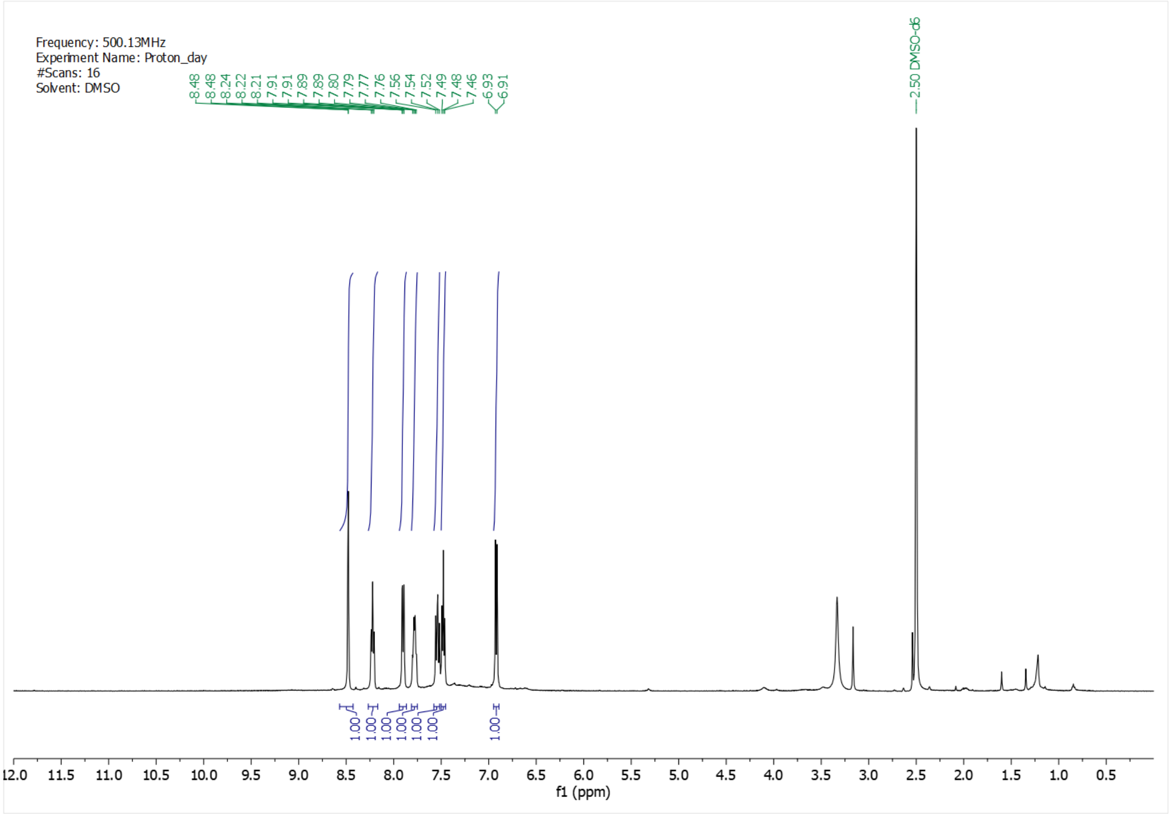

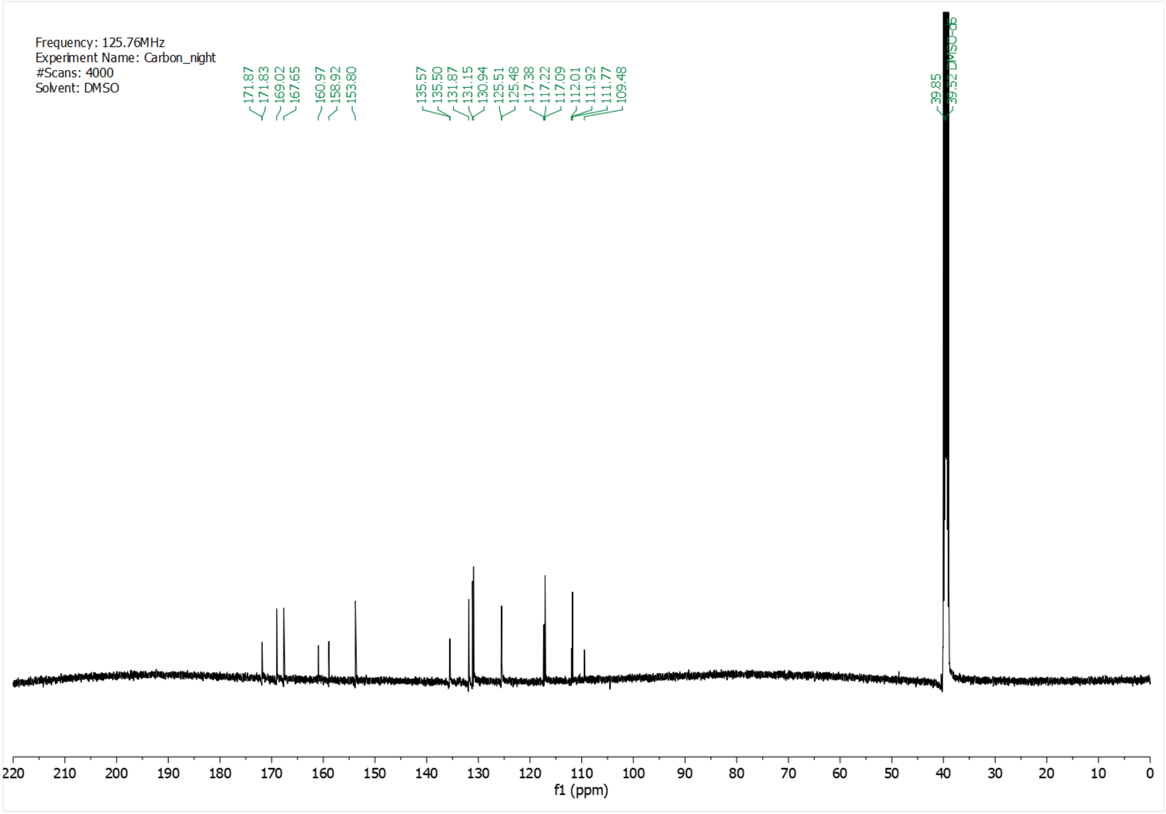

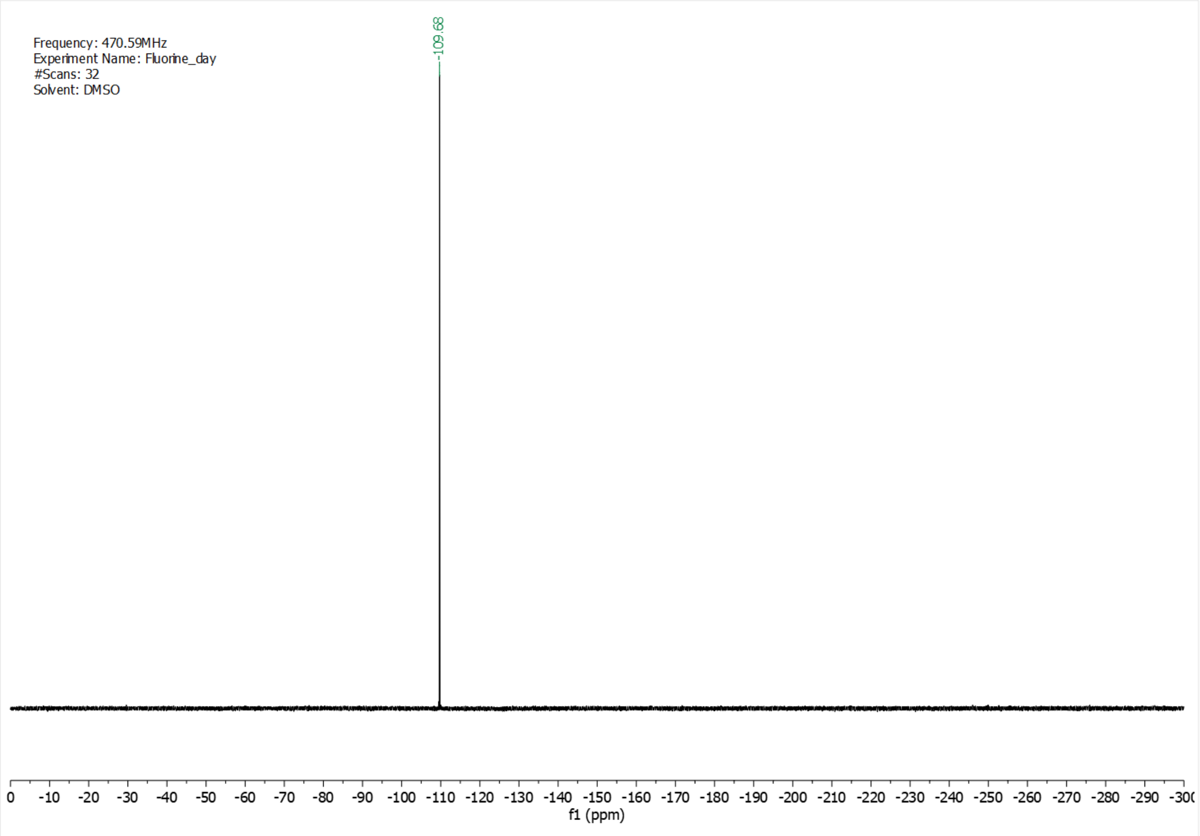
Frequency: 470.59MHz
Experiment Name: Fluorine_day
#Scans: 32
Solvent: DMSO
-109.68
f1 (ppm)

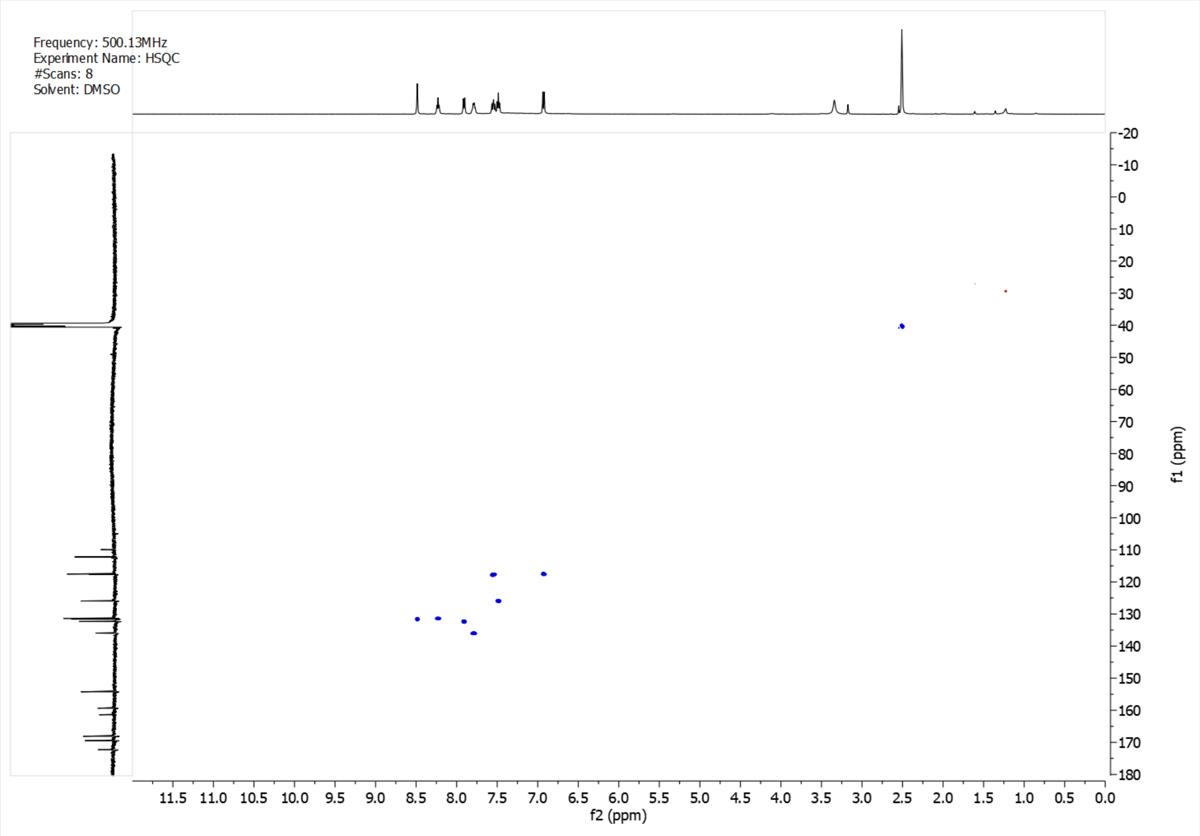
Frequency: 500.13MHz
Experiment Name: HSQC
#Scans: 8
Solvent: DMSO
f2 (ppm)
f1 (ppm)

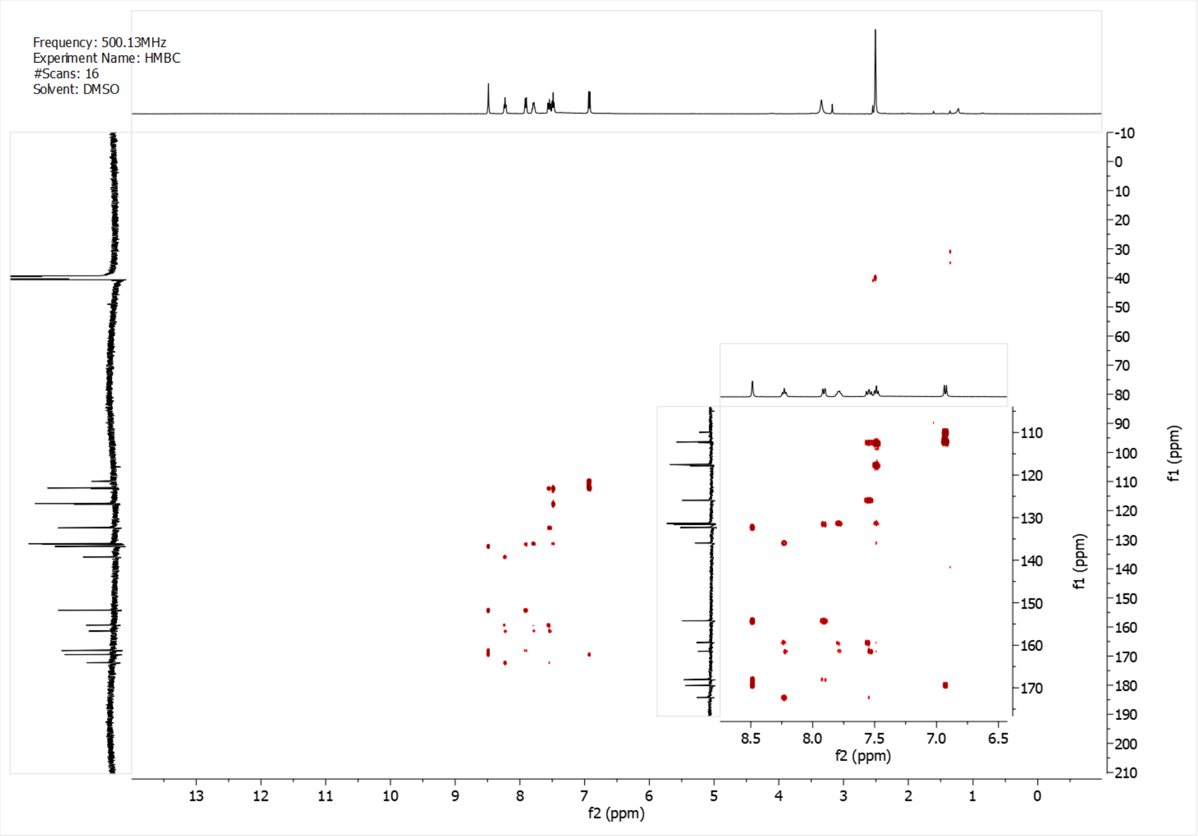

Note: Regiochemistry confirmed by HMBC. On the benzoic acid moiety, cross-peaks between H-3 and H-6 and both carboxylic acid carbonyl and heterocycle carbon on the 5-position observed. Cross-peak between H-4 and heterocycle carbon on the 5-position observed.

2-Amino-5-(6-(1-(2,2-difluorobenzo[d][1,3]dioxol-5-yl)cyclopropane-1-carboxamido)-3-methylpyridin-2-yl)benzoic acid (**3f**)

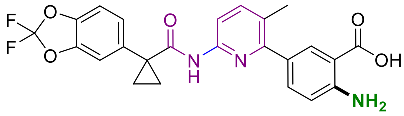

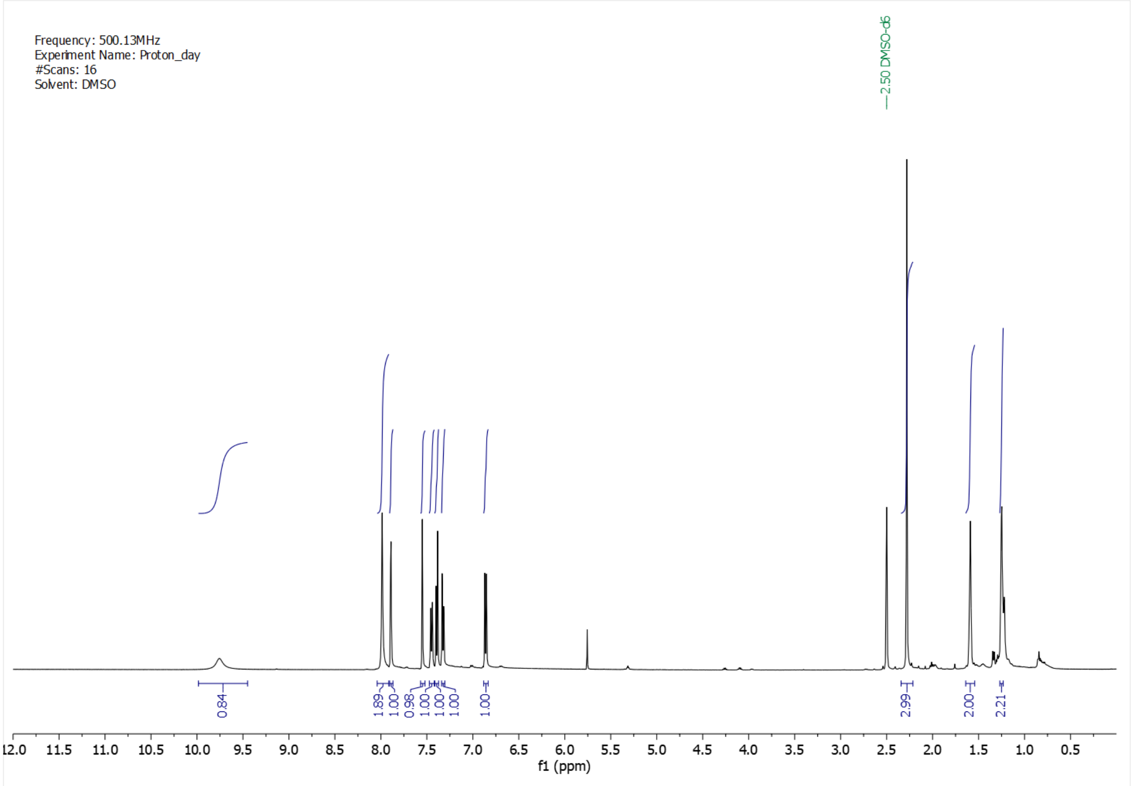
Frequency: 500.13MHz
Experiment Name: Proton_day
#Scans: 16
Solvent: DMSO
2.50 DMSO-d6
f1 (ppm)

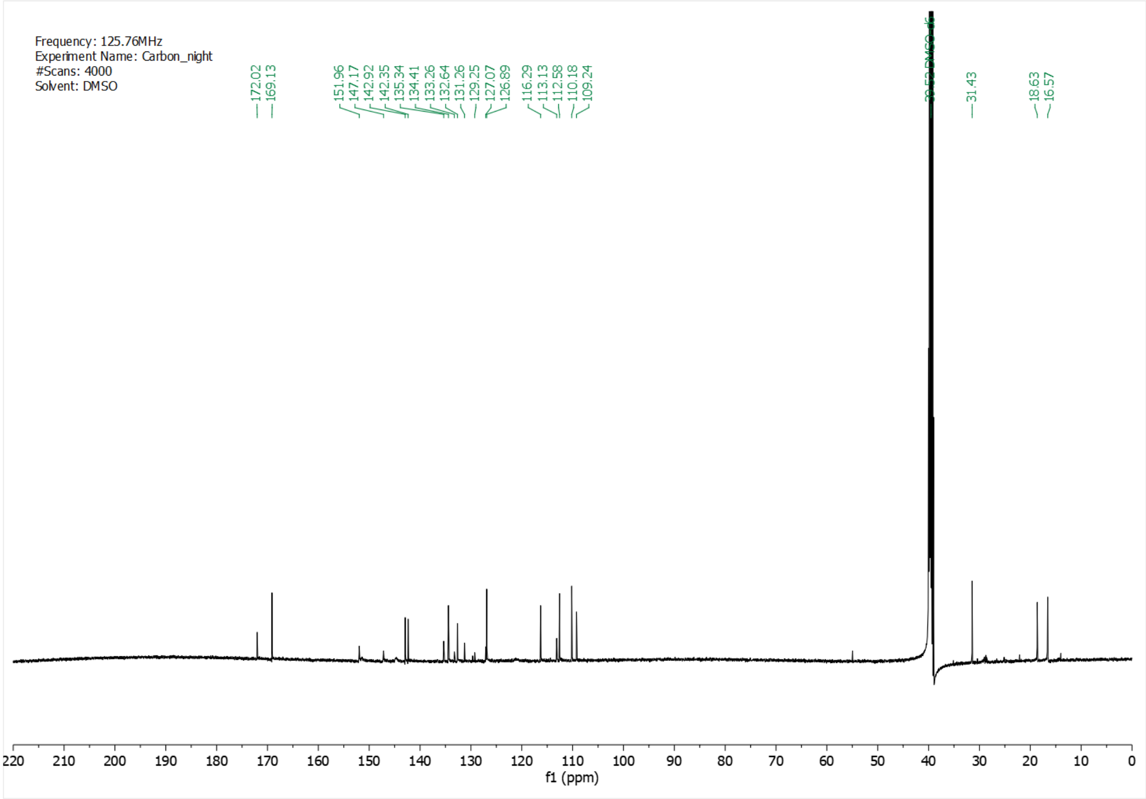
Frequency: 125.76MHz
Experiment Name: Carbon_night
#Scans: 4000
Solvent: DMSO
172.02
169.13
151.96
147.17
142.92
142.35
135.34
134.41
133.26
132.64
131.26
129.25
127.07
126.89
116.29
113.13
112.58
110.18
109.24
31.43
18.63
16.57
f1 (ppm)

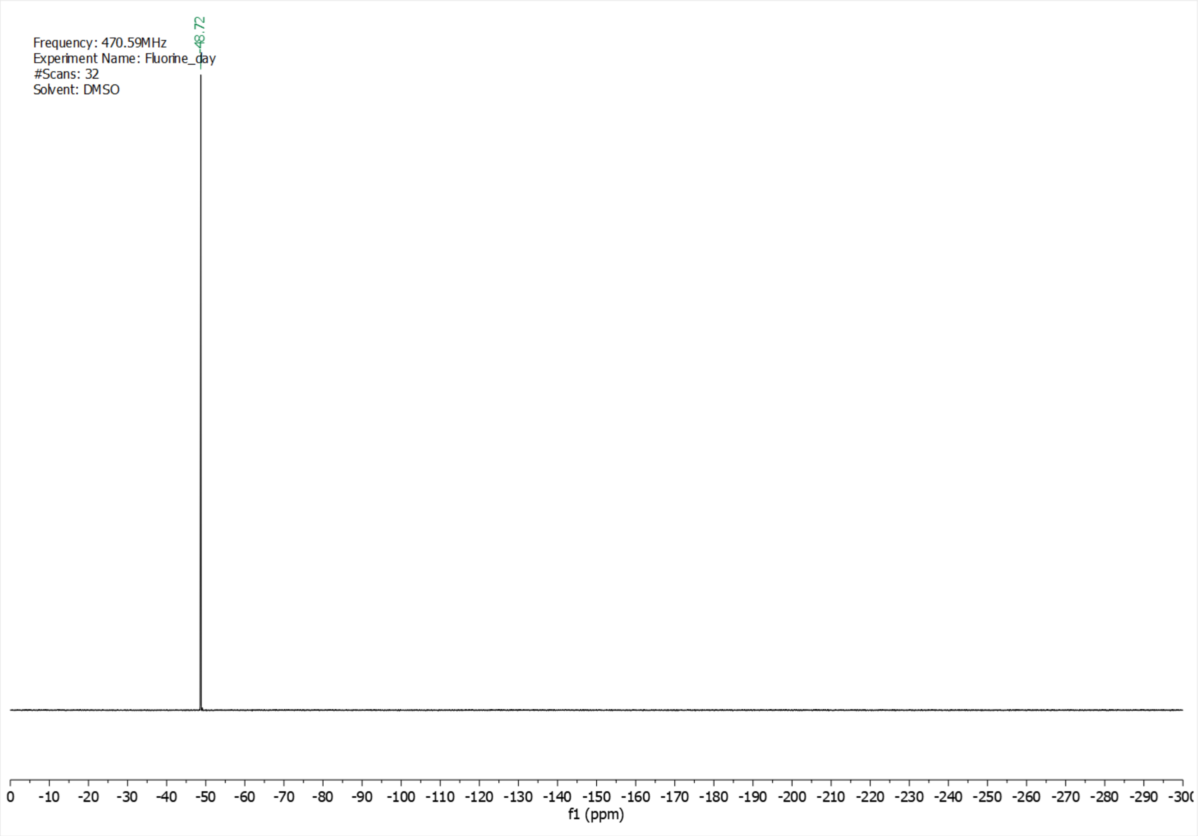
Frequency: 470.59MHz
Experiment Name: Fluorine_day
#Scans: 32
Solvent: DMSO
-48.72
f1 (ppm)

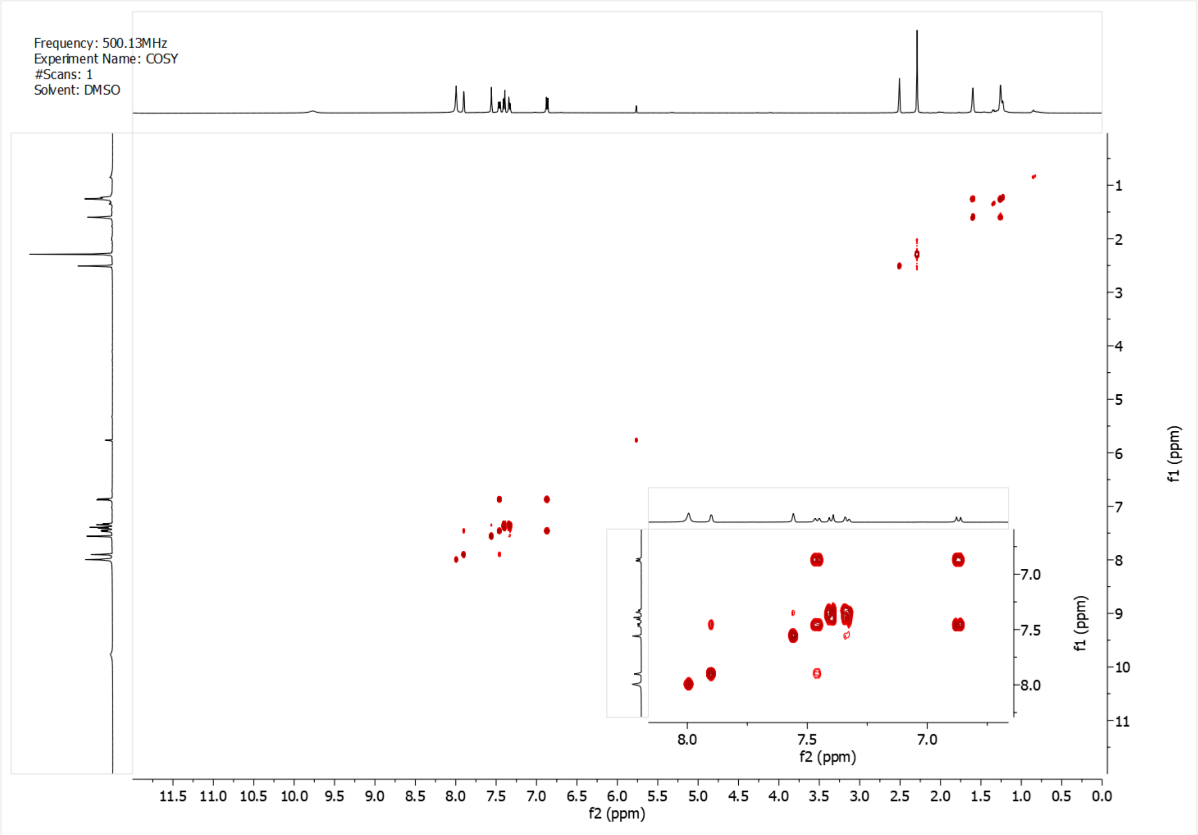
Frequency: 500.13MHz
Experiment Name: COSY
#Scans: 1
Solvent: DMSO
f1 (ppm)
f2 (ppm)

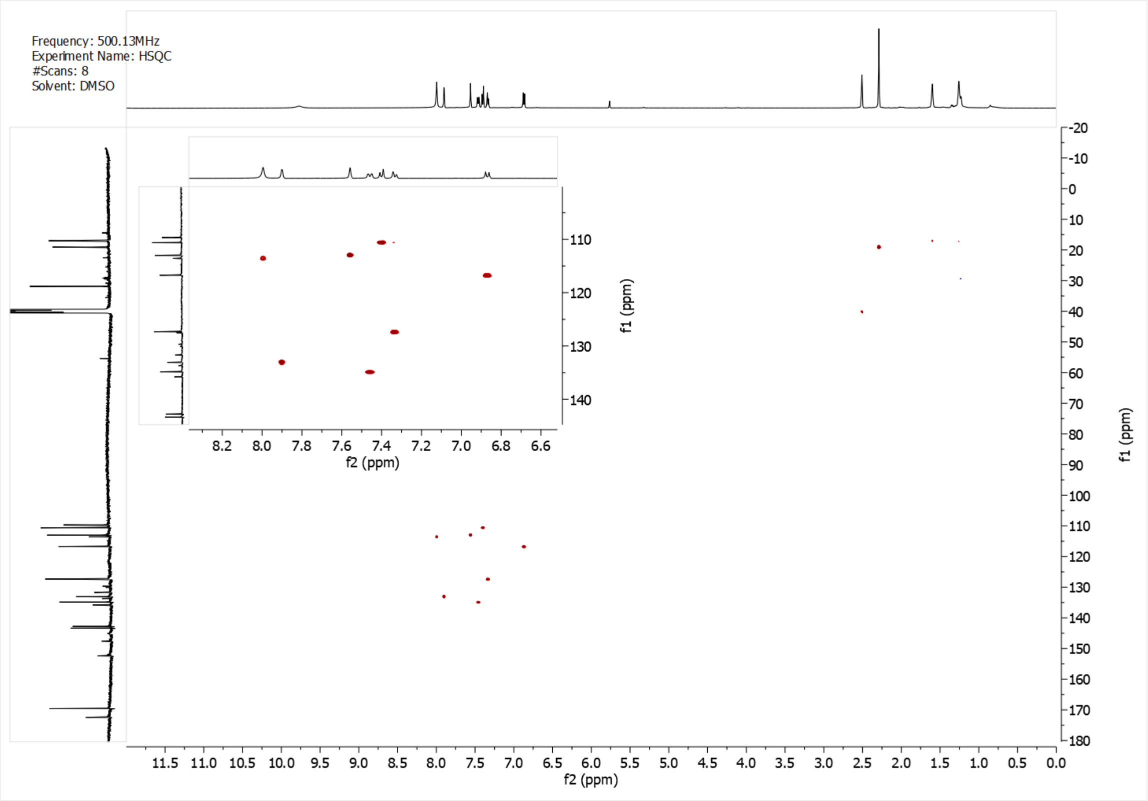

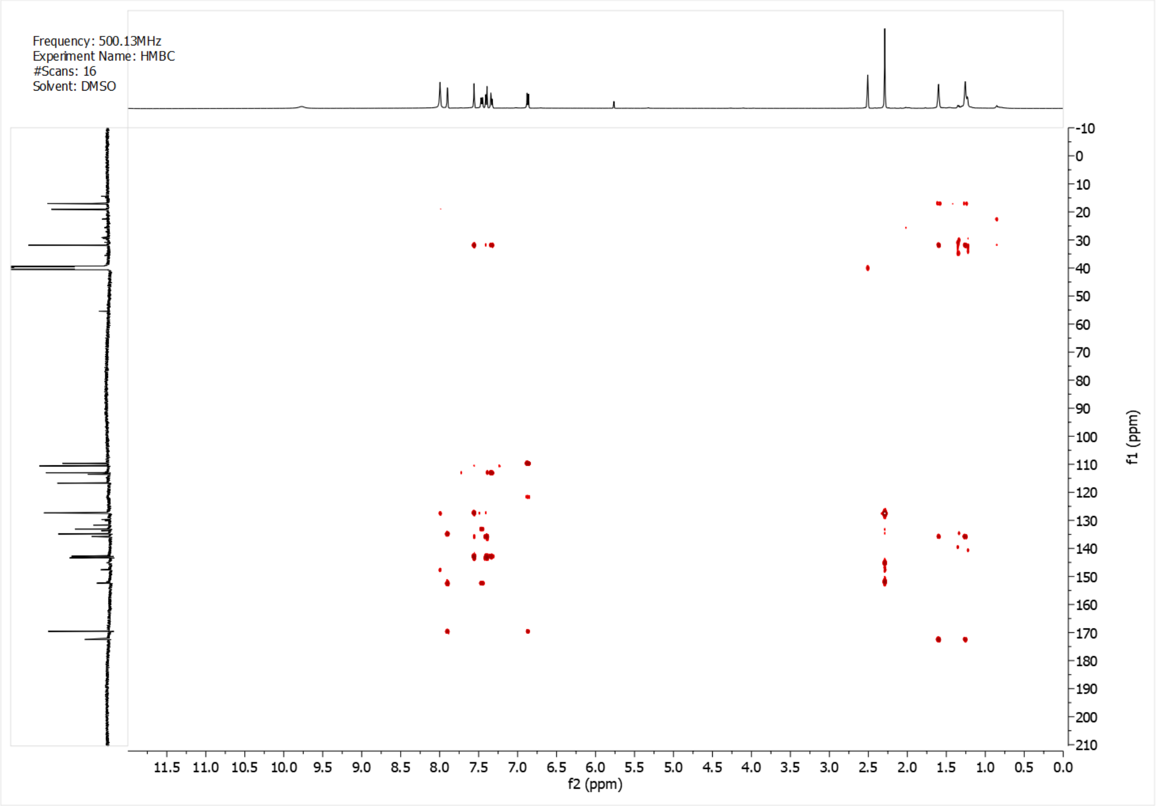

Note: Regiochemistry confirmed by HMBC. On the benzoic acid moiety, cross-peaks between H-6 and both carboxylic acid carbonyl and heterocycle carbon on the 5-position observed. Cross peak between H-3 and carboxylic acid carbon, cross peak with heterocycle carbon only at high magnification (low signal-to-noise ratio). Cross-peak between H-4 and heterocycle carbon on the 5-position observed.

5-acetyl-2-aminobenzoic acid (3g)

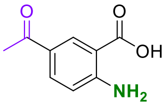

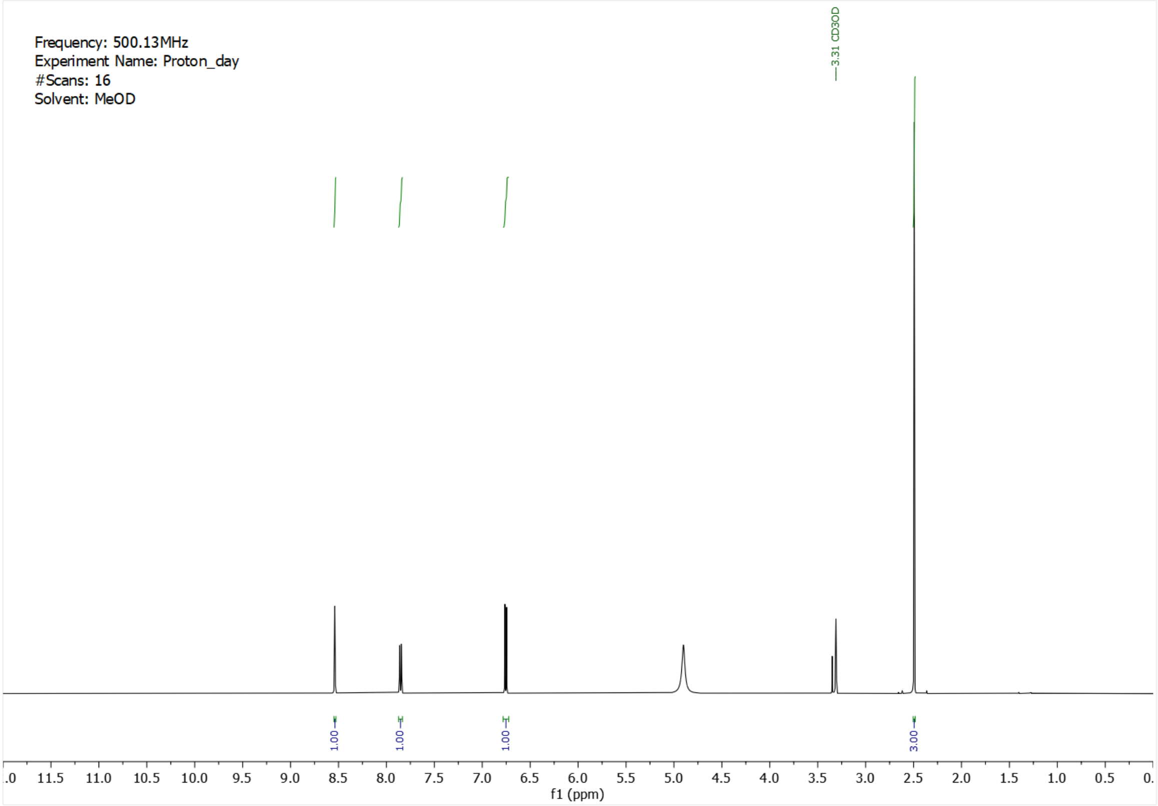

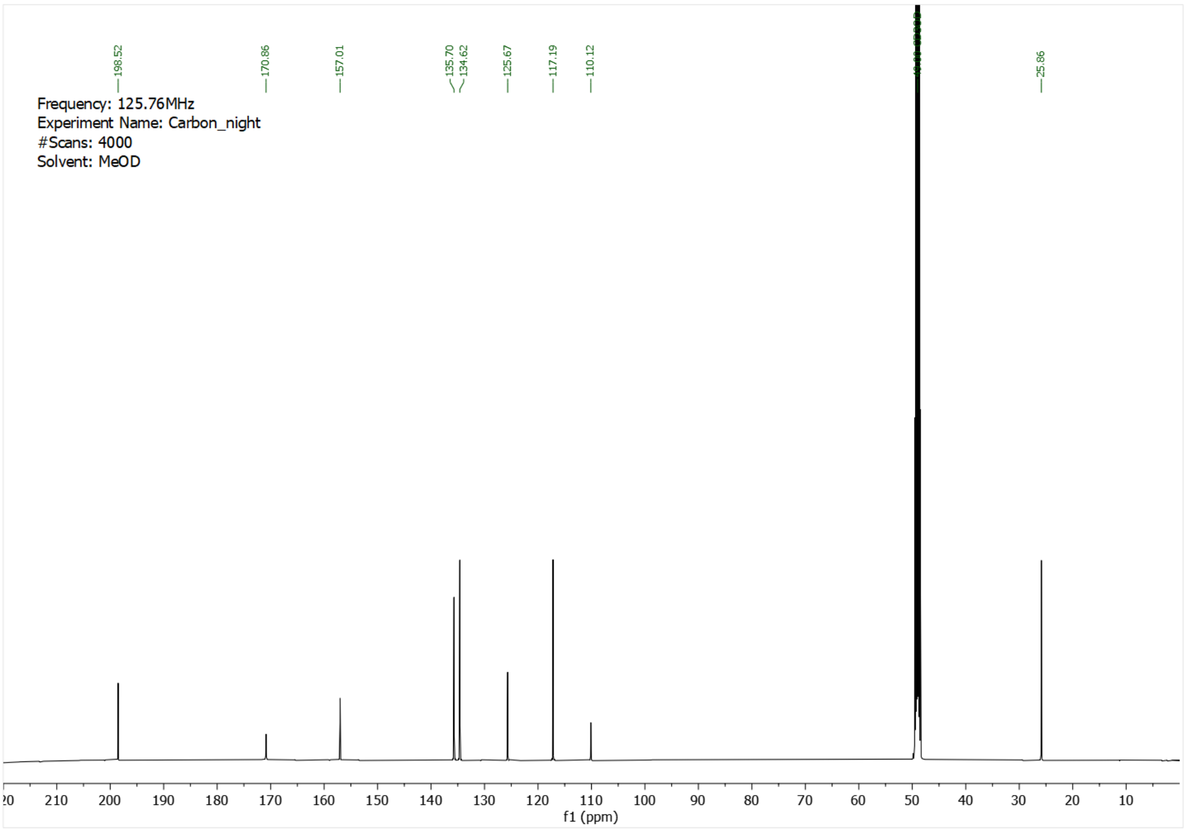

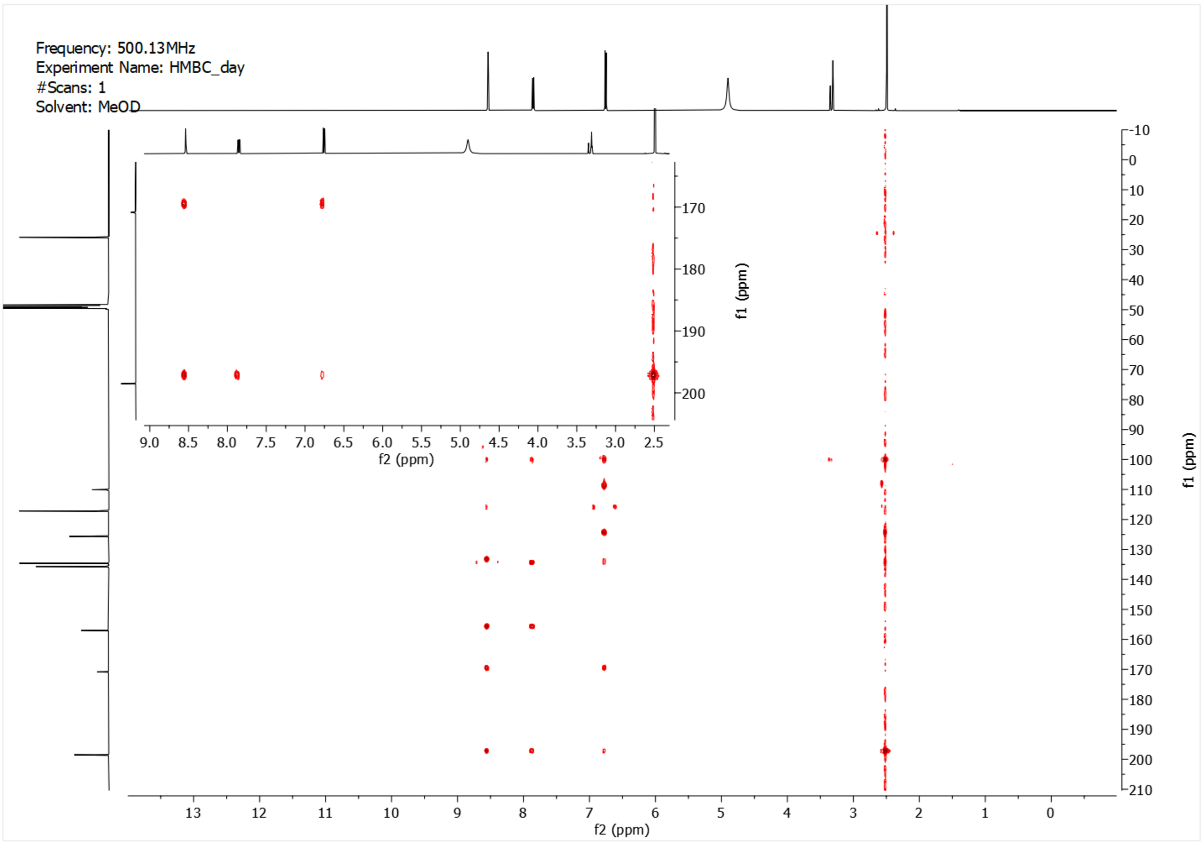

Frequency: 500.13MHz
Experiment Name: HMBC_day
#Scans: 1
Solvent: MeOD
f2 (ppm)
f1 (ppm)

(*E*)-2-(3-(3,4-Dimethoxyphenyl)acrylamido)-6-((2-(3-(2-(2-((2-(2,6-dioxopiperidin-3-yl)-1,3-dioxoisoindolin-4-yl)amino)ethoxy)ethoxy)propanamido)ethyl)sulfonamido)benzoic acid (3yb)

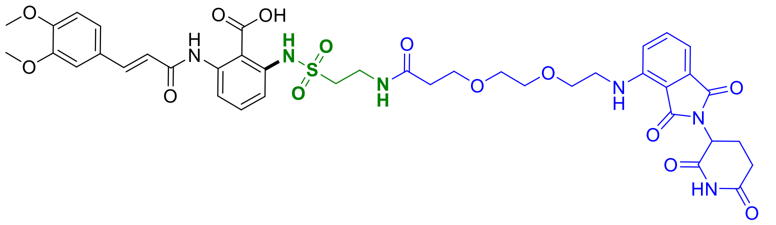

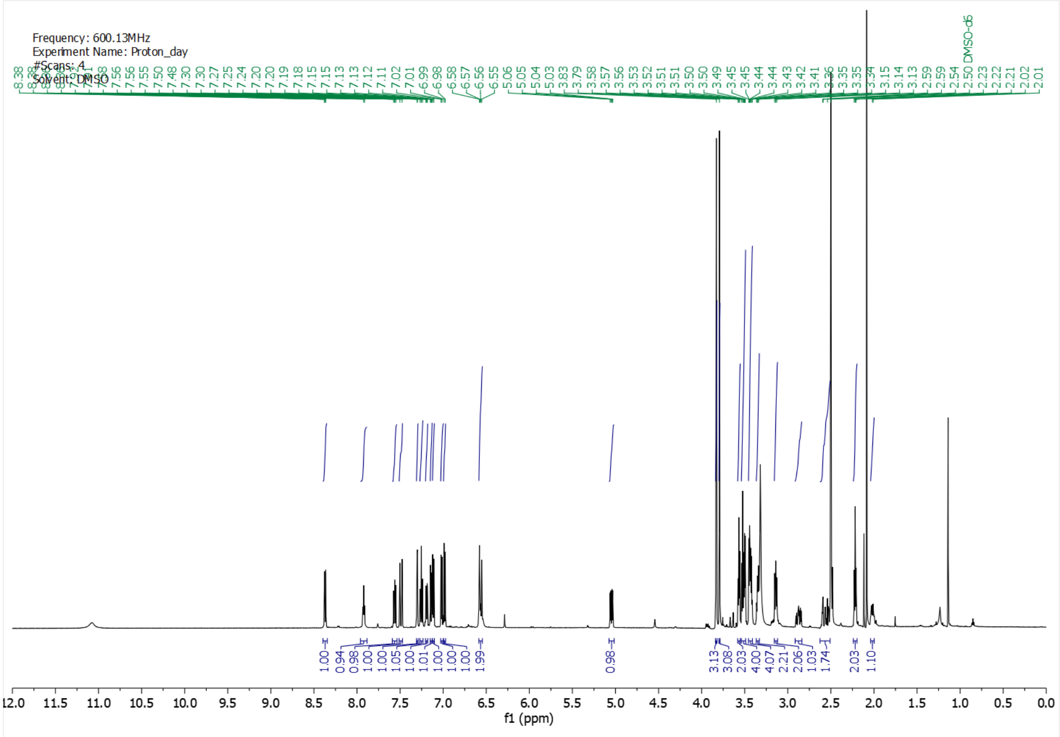

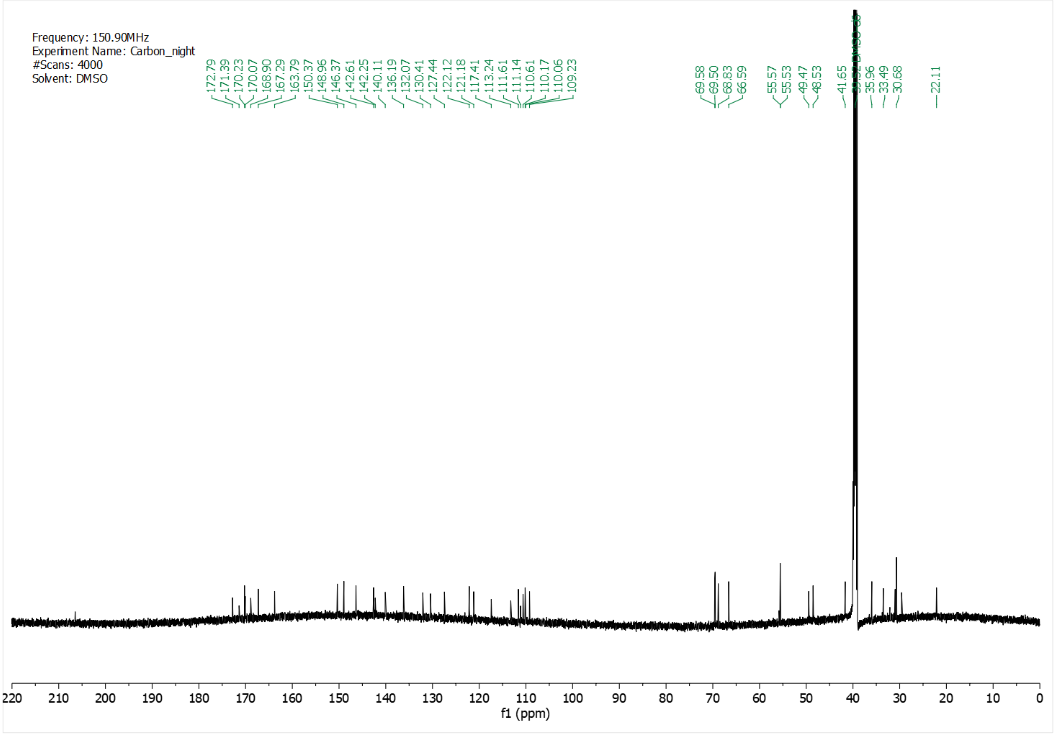

2-((*E*)-3-(3,4-Dimethoxyphenyl)acrylamido)-6-((2-(6-((3aR,4R,6aS)-2-oxohexahydro-1H-thieno[3,4-d]imidazol-4-yl)hexanamido)ethyl)sulfonamido)benzoic acid (3yc)

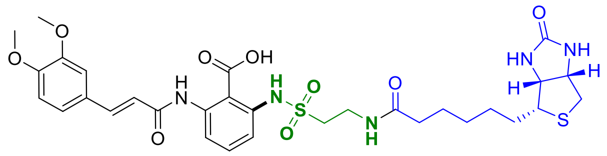

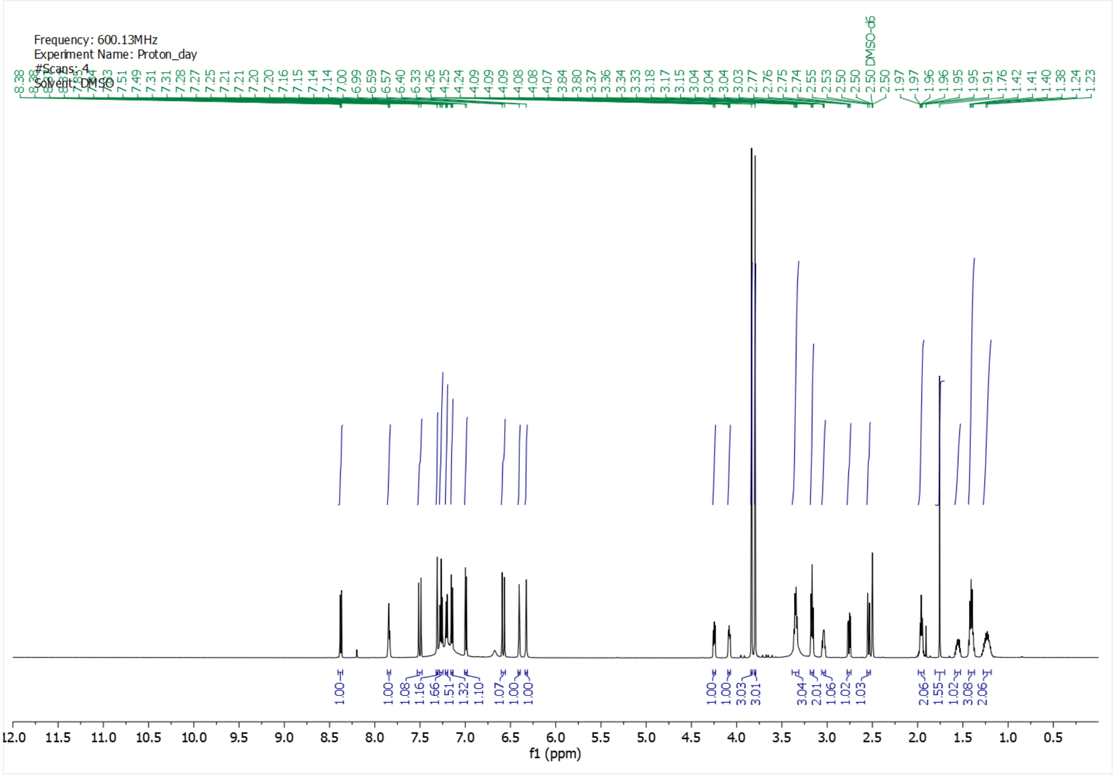

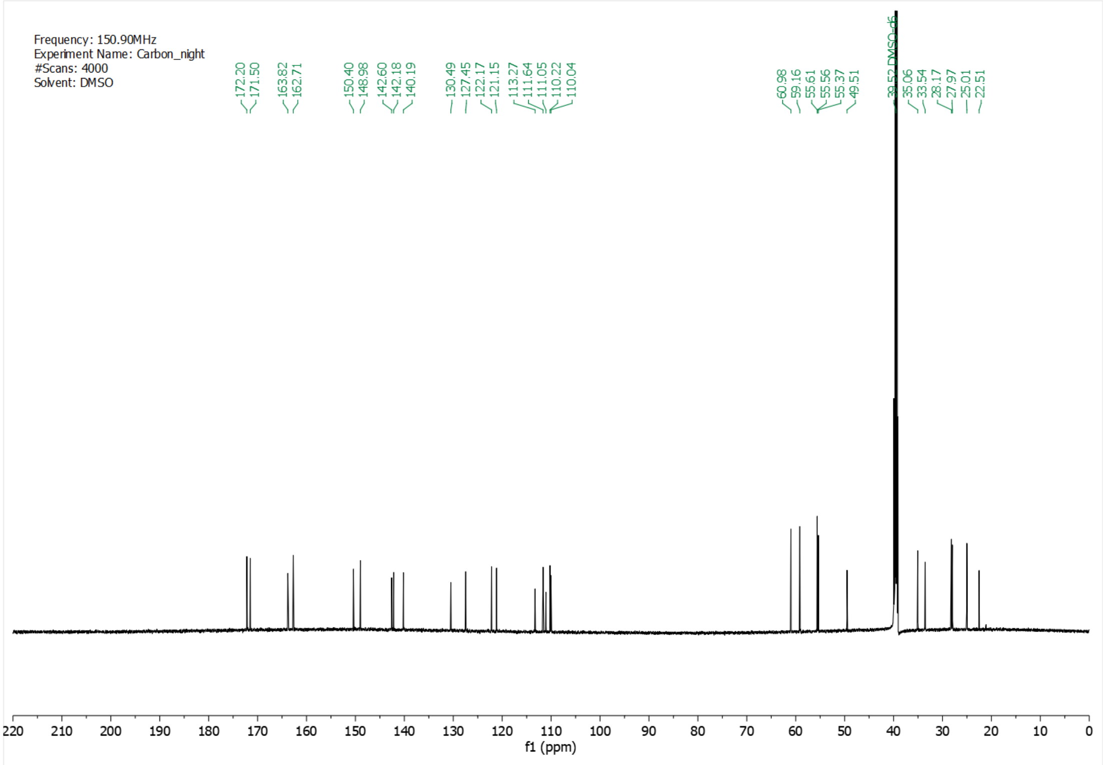

(*E*)-2-((2-(6-(3',6'-Dihydroxy-3-oxo-3H-spiro[isobenzofuran-1,9'-xanthene]-5-carboxamido)hexanamido)ethyl)sulfonamido)-6-(3-(3,4-dimethoxyphenyl)acrylamido)benzoic acid (3yd)

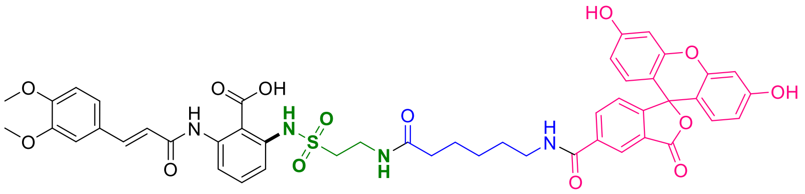

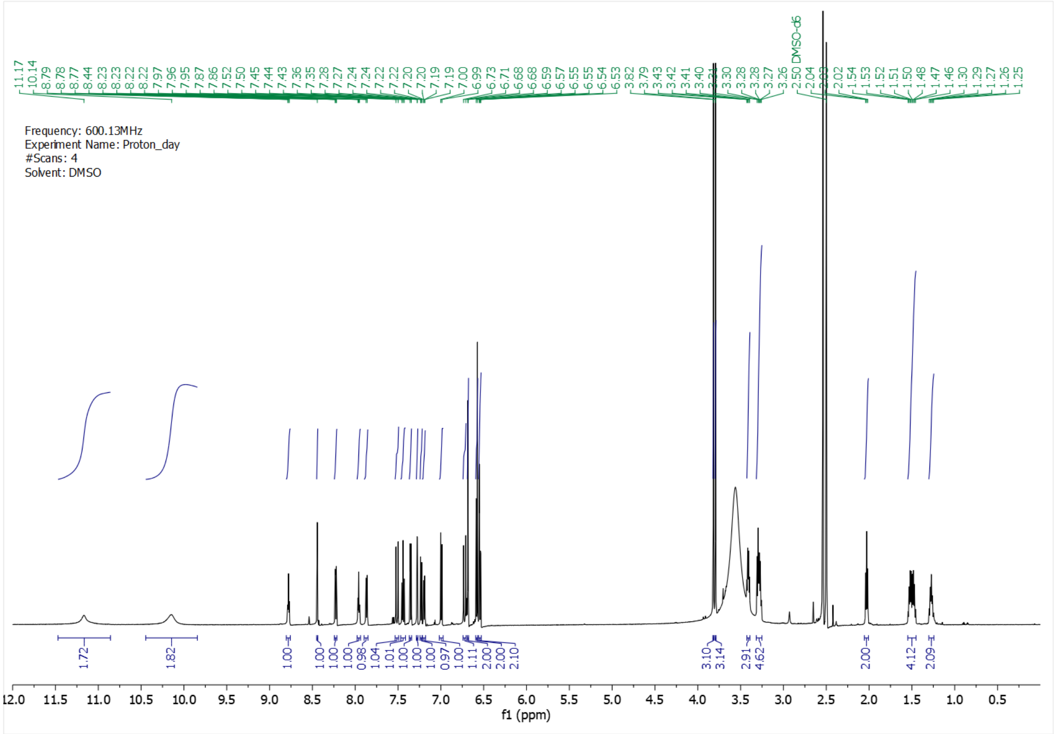

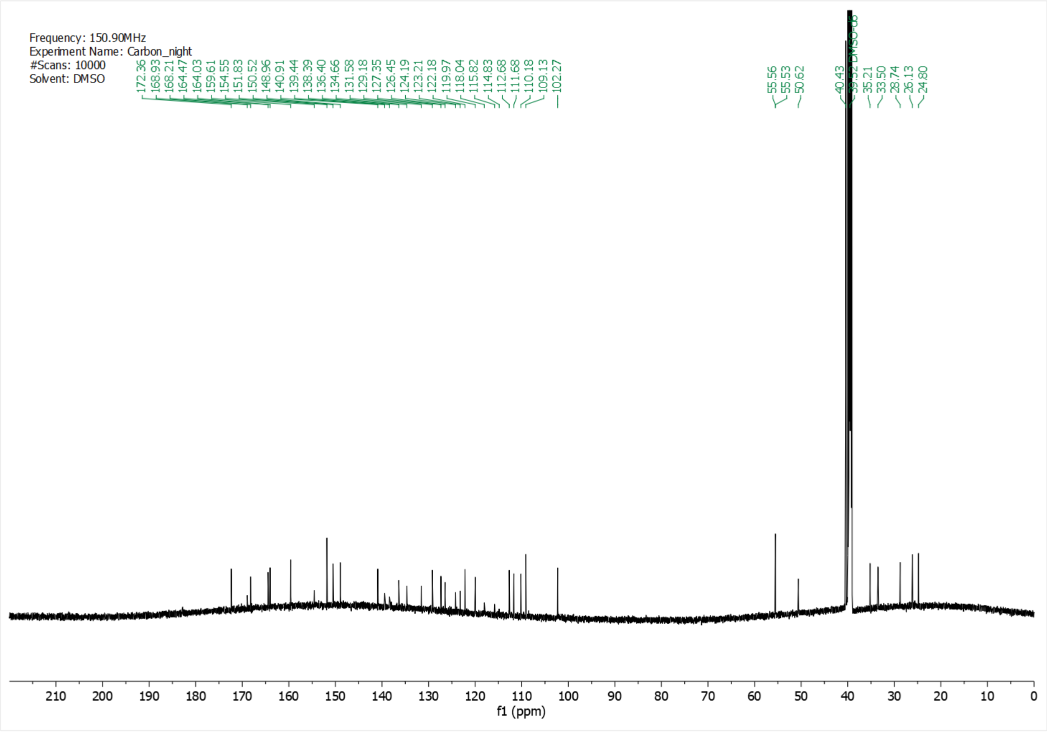

2-((2-(6-(3-(((6*S*,9*S*,12*S*,21*S*,24*R*)-6-((*S*)-1-(L-threonyl-L-alanyl-L-leucyl)pyrrolidine-2-carboxamido)-1-amino-12-(2-amino-2-oxoethyl)-24-carbamoyl-21-(hydroxymethyl)-1-imino-9-isobutyl-7,10,13,16,19,22-hexaoxo-2,8,11,14,17,20,23-heptaazapentacosan-25-yl)thio)-2,5-dioxopyrrolidin-1-yl)hexanamido)ethyl)sulfonamido)-6-((*E*)-3-(3,4-dimethoxyphenyl)acrylamido)benzoic acid (3ya)

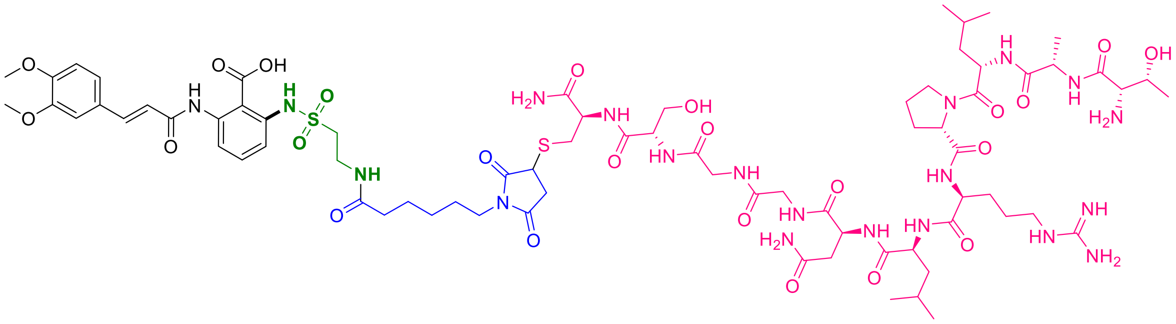

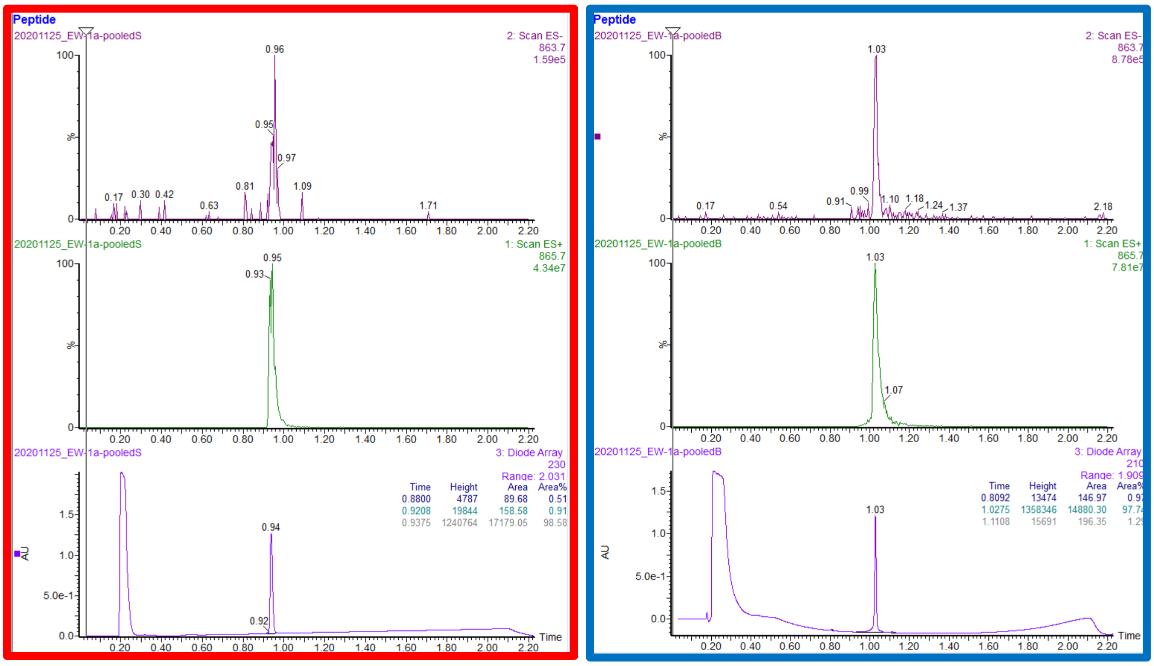

Purity analysis. Red: pH = 3. Blue: pH = 10.

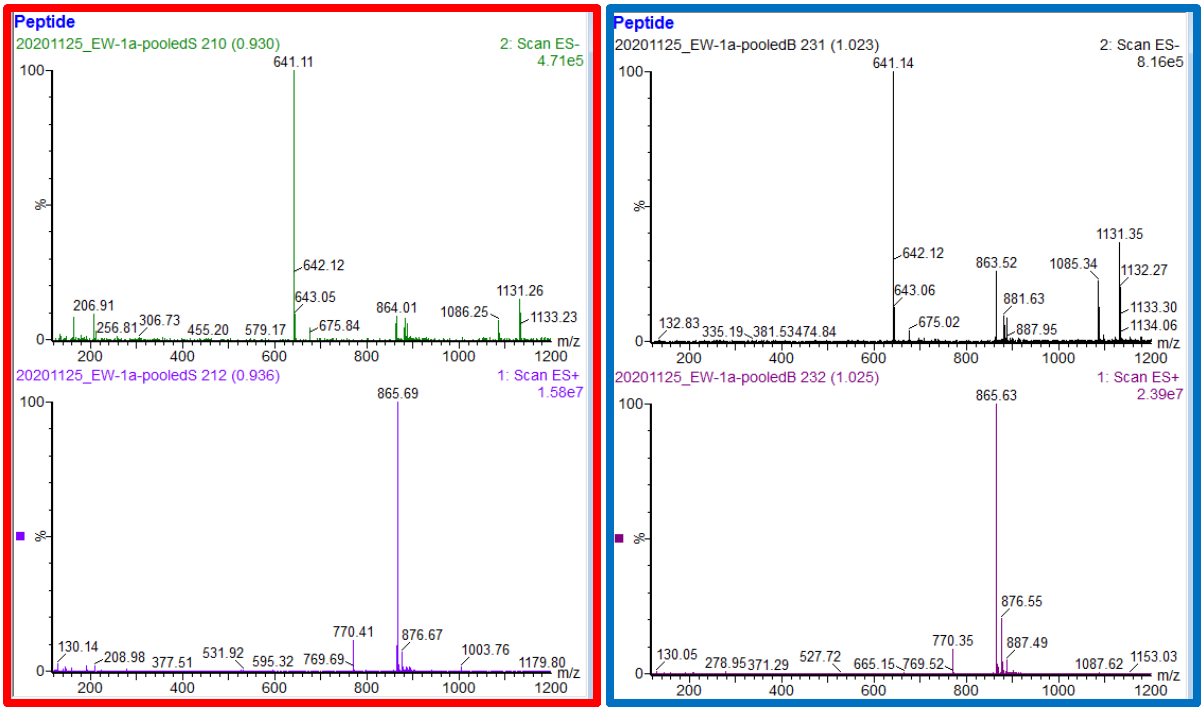

MS analysis. Red: pH = 3. Blue: pH = 10.

Fragments identified:

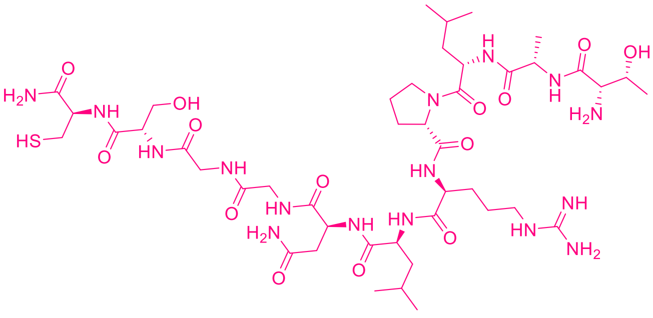

Exact Mass: 1086.5604

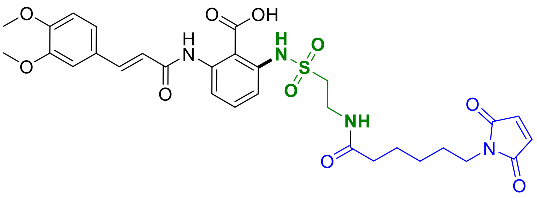

Exact Mass: 642.1996

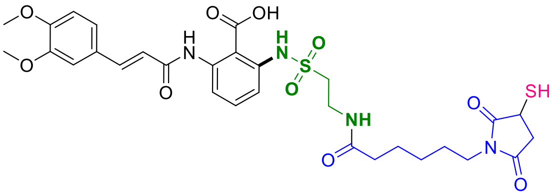

Exact Mass: 676.1873

References

1. S. P. Green, K. M. Wheelhouse, A. D. Payne, J. P. Hallett, P. W. Miller, J. A. Bull, *Org. Process Res. Dev.* **2020**, *24*, 67-84.
2. E. Erbing, A. Sanz-Marco, A. Vázquez-Romero, J. Malmberg, M. J. Johansson, E. Gómez-Bengoa, B. Martín-Matute, *ACS Catal.* **2018**, *8*, 920-925.
3. E. M. Simmons, J. F. Hartwig, *Angew. Chem. Int. Ed.* **2012**, *51*, 3066-3072.
4. A. J. Brouwer, R. M. J. Liskamp, *Synlett*, **2011**, *15*, 2228-2230.
5. A. L. Garreau, H. Zhou, M. C. Young, *Org. Lett.* **2019**, *21*, 7044-7048.
6. D. Lee, S. Chang, *Chem. Eur. J.* **2015**, *21*, 5364-5368.
7. M.-E. Wei, L.-H. Wang, Y.-D. Li, X.-L. Cui, *Chin. Chem. Lett.* **2015**, *26*, 1336-1340.
8. T. Wang, T. Hong, Y. Huang, H. Su, F. Wu, Y. Chen, L. Wei, W. Huang, X. Hua, Y. Xia, J. Xu, J. Gan, B. Yuan, Y. Feng, X. Zhang, C.-G. Yang, X. Zhou, *J. Am. Chem. Soc.* **2015**, *137*, 13736-13739.
